# Supplementary material for: A renewably sourced, circular photopolymer resin for additive manufacturing
Source: Nature. 2024 May 15;629(8014):1069–74. doi: 10.1038/s41586-024-07399-9 (PMC11136657; doi:10.1038/s41586-024-07399-9)
Supplement: Supplementary file 1 — Supplementary Text and Data including Figs. 1–185 and Tables 1–6. See Contents for details. [file 41586_2024_7399_MOESM1_ESM.pdf]

nature portfolio

https://doi.org/10.1038/s41586-024-07399-9

Supplementary information

A renewably sourced, circular photopolymer resin for additive manufacturing

In the format provided by the authors and unedited

Supplementary Materials for

A circular photopolymer resin for additive manufacturing

Thiago O. Machado, Connor J. Stubbs, Viviane Chiaradia, Maher A. Alraddadi, Arianna Brandolese, Joshua C. Worch, Andrew P. Dove.

Corresponding authors: Joshua C. Worch, jworch@vt.edu; Andrew P. Dove, a.dove@bham.ac.uk

This PDF file includes:

Synthetic Procedures
Figs. S1 to S185
Tables S1 to S6

Table of Contents

Synthetic procedures 3
NMR spectra 9
Recycled resins from 2D-photosets 25
Recycled resins from 3D-prints 39
Hydrolysed resins from 3D-printed parts 45
Hydrolysed resins from 2D-photosets 47
Mass Spectrometry 49
FT-IR spectra 58
Size-exclusion chromatography (SEC) data for depolymerization of 2D-photosets 94
TGA thermograms 111
DSC thermograms 124
Mechanical (tensile) properties 133
Recycling and re-curing/printing resins using catalyzed depolymerization (phosphazene:thiophenol) method 156
Recycling and re-curing/printing resins using thermal depolymerization (DMF, 140 °C) method 176
Irradiance of light sources 196
Summary of thermomechanical data for materials produced from all synthesized lipoate formulations 197
Summary of thermomechanical data for commercial 3D printing resins 198

Synthetic procedures

Synthesis of resin component monomers

Synthesis of MenLp1

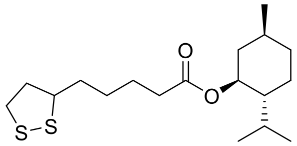

Lipoic acid (10 g, 1 equiv., 48.4 mmol), menthol (6 mL, molar excess), DMAP (5.91 g, 1 equiv., 48.4 mmol) were placed in a 500 mL single neck round-bottom flask. DCM (200 mL) was added to the flask and the mixture was stirred until all reagents were dissolved (ca. 10 min). The reaction mixture was then cooled to 0 °C in an ice-water bath and EDC·HCl (9.28 g, 1 equiv., 48.4 mmol) was added portion-wise over 5 min. After the addition was complete, the reaction was stirred for 15 min at 0 °C, then removed from the ice-bath. The flask was wrapped in aluminum foil to protect from ambient light and stirred overnight at ambient temperature (*ca*. 16 h at 22 °C). The reaction mixture was washed with HCl (3 × 50 mL), $NaHCO_3$ (1 × 50 mL) and brine (1 × 50 mL) before drying over $MgSO_4$. The resultant mixture is transferred to a 250 mL round-bottom flask and concentrated *in vacuo* to afford a yellow oil (yield = 9.72 g, 81%). ^{1}H NMR (400 MHz, $CDCl_3$) δ 4.67 (td, *J* = 10.9, 4.4 Hz, 1H), 3.56 (dq, *J* = 8.3, 6.4 Hz, 1H), 3.24 – 3.00 (m, 2H), 2.40 – 2.49 (m, 1H), 2.29 (t, *J* = 7.4 Hz, 2H), 2.02 – 1.77 (m, overlapping, 3H), 1.75 – 1.56 (m, overlapping, 6H), 1.56 – 1.30 (m overlapping, 4H), 1.11 – 0.77 (m overlapping, 9H), 0.75 (d, *J* = 7.0 Hz, 3H). ^{13}C NMR (101 MHz, $CDCl_3$) δ 173.18, 74.16, 56.47, 47.13, 41.09, 40.32, 38.60, 34.74, 34.60, 34.38, 31.50, 28.88, 26.40, 24.97, 23.53, 22.15, 20.90, 16.43. HRMS (TOF-MS) (*m*/*z*): $[M + Na]^+$ calculated for $C_{18}H_{32}O_2NaS_2$, 367.1741; found, 367.1744. FT-IR: 1716 cm^{-1} (C=O ester).

Synthesis of IsoLp2

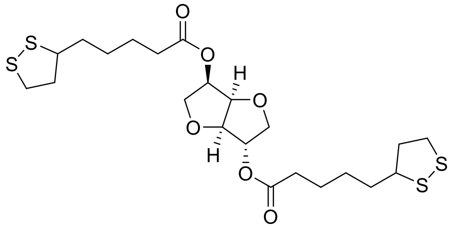

Lipoic acid (25 g, 2.2 equiv., 121 mmol), isosorbide (8 g, 1 equiv., 55 mmol), DMAP (16.13g, 2.4 equiv., 132 mmol) were placed in a 500 mL single neck round-bottom flask. DCM (200 mL) was added to the flask and the mixture was stirred until all reagents were dissolved (ca. 10 min). The reaction mixture was then cooled to 0 °C in an ice-water bath and EDC·HCl (25.3 g, 2.4 equiv., 132 mmol) was added portion-wise over 5 min. After the addition was complete, the reaction was stirred for 15 min at 0 °C, then removed from the ice-bath. The flask was wrapped in aluminum foil to protect from ambient light and stirred overnight at ambient temperature (*ca*. 16 h at 22 °C). The reaction mixture was washed HCl (3 × 50 mL), $NaHCO_3$ (1 × 50 mL) and brine (1 × 50 mL) before drying over $MgSO_4$. The residue was filtered through short pad of basic alumina and silica. The resultant mixture is transferred to a 250 mL round-bottom flask and concentrated *in vacuo* to afford a thick yellow oil that readily solidified upon standing at ambient temperature (yield = 25.2 g, 88%). ^{1}H NMR (400 MHz, $CDCl_3$) δ 5.22 (d, *J* = 3.0 Hz, 1H), 5.17 (q, *J* = 5.6 Hz, 1H), 4.85 (t, *J* = 5.0 Hz, 1H), 4.50 (d, *J* = 4.6 Hz, 1H), 4.04 – 3.91 (m overlapping, 3H), 3.82 (dd, *J* = 9.8, 5.4 Hz, 1H), 3.64 – 3.52 (m, 2H), 3.26 – 3.07 (m, 4H), 2.55 – 2.30 (m overlapping, 6H), 1.98 – 1.85 (m, 2H), 1.78 – 1.61 (m, 8H), 1.56 – 1.37 (m, 4H). ^{13}C NMR (101 MHz, $CDCl_3$) δ 172.94, 172.63, 86.08, 80.87, 78.07, 73.97, 73.58, 70.50, 56.45, 40.37, 38.63, 34.72, 34.03, 33.83, 28.79, 24.68. HRMS (TOF-MS) (*m*/*z*): $[M + Na]^+$ calculated for $C_{22}H_{34}O_6NaS_4$, 545.1136; found, 545.1141. FT-IR: 1725 cm^{-1} (C=O ester).

Synthesis of EtLp1

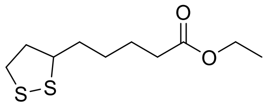

Lipoic acid (20 g, 1 equiv., 97 mmol), ethanol (6 mL, molar excess), DMAP (11.8 g, 1 equiv., 97 mmol) were placed in a 500 mL single neck round-bottom flask. DCM (200 mL) was added to the flask and the mixture was stirred until all reagents were dissolved (*ca.* 10 min). The reaction mixture was then cooled to 0 °C in an ice-water bath and EDC·HCl (18.5 g, 1 equiv., 97 mmol) was added portion-wise over 5 min. After the addition was complete, the reaction was stirred for 15 min at 0 °C, then removed from the ice-bath. The flask was wrapped in aluminum foil to protect from ambient light and stirred overnight at ambient temperature (*ca.* 16 h at 22 °C). The reaction mixture was washed with HCl (3 × 100 mL), $NaHCO_3$ (1 × 100 mL) and brine (1 × 100 mL) before drying over $MgSO_4$. The resultant mixture is transferred to a 250 mL round-bottom flask and concentrated *in vacuo* to afford a yellow oil (yield = 17.5 g, 78%). ^{1}H NMR (400 MHz, $CDCl_3$) δ 4.12 (q, *J* = 7.2 Hz, 2H), 3.57 (dq, *J* = 8.4, 6.4 Hz, 1H), 3.23 – 3.06 (m, 2H), 2.45 (ddd, *J* = 13.1, 6.5, 5.4 Hz, 1H), 2.31 (t, *J* = 7.4 Hz, 2H), 1.91 (dq, *J* = 12.9, 6.9 Hz, 1H), 1.79 – 1.57 (m, 3H), 1.55 – 1.39 (m, 2H), 1.25 (t, *J* = 7.1 Hz, 3H). ^{13}C NMR (101 MHz, $CDCl_3$) δ 173.67, 60.43, 56.49, 40.36, 38.62, 34.74, 34.26, 28.90, 24.83, 14.39. HRMS (TOF-MS) (*m/z*): $[M]^+$ calculated for $C_{10}H_{18}O_2S_2$, 234.0748; found, 234.0748. FT-IR: 1724 cm^{-1} (C=O ester).

Synthesis of GlyLp3

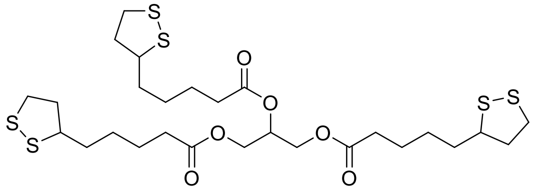

Lipoic acid (48.4 g, 3 equiv., 235 mmol), glycerol (7.2 g, 1 equiv., 78 mmol), DMAP (28.7 g, 3 equiv., 235 mmol) were placed in a 1 L single neck round-bottom flask. DCM (400 mL) was added to the flask and the mixture was stirred until all reagents were dissolved (ca. 10 min). The reaction mixture was then cooled to 0 °C in an ice-water bath and EDC·HCl (44.9 g, 3 equiv., 235 mmol) was added portion-wise over 5 min. After the addition was complete, the reaction was stirred for 15 min at 0 °C, then removed from the ice-bath. The flask was wrapped in aluminum foil to protect from ambient light and stirred overnight at ambient temperature (*ca.* 16 h at 22 °C). The reaction mixture was washed with HCl (3 × 200 mL), $NaHCO_3$ (1 × 200 mL) and brine (1 × 200 mL) before drying over $MgSO_4$. The resultant mixture is transferred to a 500 mL round-bottom flask and concentrated *in vacuo* to afford a yellow oil (yield = 41.3 g, 81%). ^{1}H NMR (400 MHz, $CDCl_3$) δ 5.26 (q, *J* = 4.8 Hz, 1H) 4.31 (dd, *J* = 11.9, 4.2 Hz, 2H), 4.14 (dd, *J* = 11.9, 5.9 Hz, 2H), 3.57 (dq, *J* = 8.9, 6.3 Hz, 3H), 3.23 – 2.95 (m, 6H), 2.47 (m, 3H), 2.31 – 2.38 (m, 6H), 1.96 – 1.82 (m, 3H), 1.75 – 1.55 (m, 12H), 1.57 – 1.32 (m, 6H). ^{13}C NMR (101 MHz, $CDCl_3$) δ 173.06, 69.14, 62.30, 56.49, 56.47, 53.57, 40.41, 40.39, 38.65, 34.74, 34.09, 33.93, 28.86, 28.83, 24.74, 24.71. HRMS (TOF-MS) (*m/z*): $[M]^+$ calculated for $C_{27}H_{44}O_6S_6$, 656.1462; found, 656.1467. FT-IR: 1730 cm^{-1} (C=O ester).

Synthesis of GuaLp1

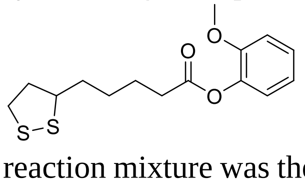

Lipoic acid (10 g, 1 equiv., 48.4 mmol), guaicol (6 mL, molar excess), DMAP (5.91 g, 1 equiv., 48.4 mmol) were placed in a 500 mL single neck round-bottom flask. DCM (200 mL) was added to the flask and the mixture was stirred until all reagents were dissolved (ca. 10 min). The reaction mixture was then cooled to 0 °C in an ice-water bath and EDC·HCl (9.28 g, 1 equiv., 48.4 mmol) was added portion-wise over 5 min. After the addition was complete, the reaction was stirred for 15 min at 0 °C, then removed from the ice-bath. The flask was wrapped in aluminum

foil to protect from ambient light and stirred overnight at ambient temperature (*ca*. 16 h at 22 °C). The reaction mixture was washed HCl (3 × 50 mL), $NaHCO_3$ (1 × 50 mL) and brine (1 × 50 mL) before drying over $MgSO_4$. The resultant mixture is transferred to a 250 mL round-bottom flask and concentrated *in vacuo* to afford a yellow oil (yield = 12.4 g, 82%). ^{1}H NMR (400 MHz, $CDCl_3$) δ 7.20 (ddd, *J* = 8.2, 7.4, 1.7 Hz, 1H), 7.02 (dd, *J* = 7.9, 1.8 Hz, 1H), 6.99 – 6.90 (m, 2H), 3.83 (s, 3H), 3.65 – 3.56 (m, 1H), 3.25 – 3.07 (m, 2H), 2.60 (t, *J* = 7.3 Hz, 2H), 2.48 (dtd, *J* = 13.0, 6.6, 5.4 Hz, 1H), 2.00 – 1.90 (m, 1H), 1.90 – 1.70 (m, 4H), 1.70 – 1.48 (m, 2H). ^{13}C NMR (101 MHz, $CDCl_3$) δ 171.67, 151.25, 139.91, 126.97, 122.93, 120.90, 112.53, 56.52, 55.99, 40.38, 38.64, 34.77, 33.93, 28.80, 24.90. HRMS (TOF-MS) (*m*/*z*): $[M + Na]^+$ calculated for $C_{15}H_{20}O_3NaS_2$, 335.0752; found, 335.0762. FT-IR: 1751 cm^{-1} (C=O ester).

Synthesis of SteaLp₁

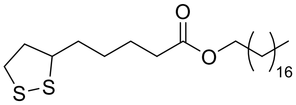

Lipoic acid (10 g, 1 equiv., 48.4 mmol), stearyl alcohol (6 mL, molar excess), DMAP (5.91 g, 1 equiv., 48.4 mmol) were placed in a 500 mL single neck round-bottom flask. DCM (200 mL) was added to the flask and the mixture was stirred until all reagents were dissolved (ca. 10 min). The reaction mixture was then cooled to 0 °C in an ice-water bath and EDC·HCl (9.28 g, 1 equiv., 48.4 mmol) was added portion-wise over 5 min. After the addition was complete, the reaction was stirred for 15 min at 0 °C, then removed from the ice-bath. The flask was wrapped in aluminum foil to protect from ambient light and stirred overnight at ambient temperature (*ca*. 16 h at 22 °C). The reaction mixture was washed with HCl (3 × 50 mL), $NaHCO_3$ (1 × 50 mL) and brine (1 × 50 mL) before drying over $MgSO_4$. The resultant mixture is transferred to a 250 mL round-bottom flask and concentrated *in vacuo* to afford a yellow oil (yield = 19.3 g, 87%). ^{1}H NMR (400 MHz, $CDCl_3$) δ 4.06 (t, *J* = 6.8 Hz, 2H), 3.57 (dq, *J* = 8.4, 6.4 Hz, 1H), 3.23 – 3.06 (m, 2H), 2.40 – 2.49 (m, 1H), 2.31 (t, *J* = 7.4 Hz, 2H), 1.91 (dq, *J* = 12.8, 7.0 Hz, 1H), 1.77 – 1.56 (m overlapping, 6H), 1.56 – 1.39 (m, 2H), 1.36 – 1.24 (m overlapping, 30H), 0.92 – 0.84 (t, *J* = 6.5 Hz, 3H). . ^{13}C NMR (101 MHz, $CDCl_3$) δ 173.76, 64.70, 56.50, 40.36, 38.63, 34.77, 34.28, 32.07, 29.85, 29.81, 29.74, 29.68, 29.51, 29.41, 28.93, 28.80, 26.09, 24.88, 22.84, 14.27. HRMS (TOF-MS) (*m*/*z*): $[M + Na]^+$ calculated for $C_{26}H_{50}O_2NaS_2$, 481.3150; found, 481.3146. FT-IR: 1722 cm^{-1} (C=O ester).

<u>Green (alternative) synthesis of monomers</u>

Green synthesis of IsoLp₂

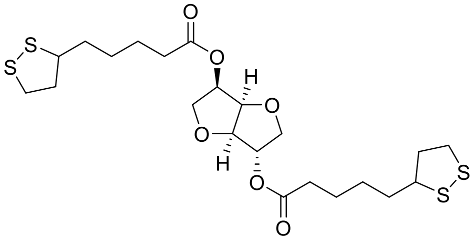

Lipoic acid (3.0 g, 2.2 equiv., 14.5 mmol), isosorbide (0.96 g, 1 equiv., 6.6 mmol) and *p*-toluenesulfonic acid (PTSA) (0.039 g, 0.03 equiv., 0.20 mmol) were placed in a two-neck round-bottom flask and connected to a condenser under N_2 atmosphere. The flask was wrapped in aluminum foil to protect from ambient light and stirred for 7 h at 140 °C. After 7 h, a solution containing BHT (2.2 wt% in methyl THF) was added dropwise to the reaction mixture, which was then poured into a round-bottom flask containing cold methyl THF (−78 °C). The crude was purified using a silica/basic alumina plug and concentrated *in vacuo* to afford a yellow solid (yield = 2.27 g, 66%). ^{1}H NMR (400 MHz, $CDCl_3$) δ 5.22 (d, *J* = 3.0 Hz, 1H), 5.17 (q, *J* = 5.6 Hz, 1H), 4.85 (t, *J* = 5.0 Hz, 1H), 4.50 (d, *J* = 4.6 Hz, 1H), 4.04 – 3.91 (m overlapping, 3H), 3.82 (dd, *J* = 9.8, 5.4 Hz, 1H), 3.64 –

3.52 (m, 2H), 3.26 – 3.07 (m, 4H), 2.55 – 2.30 (m overlapping, 6H), 1.98 – 1.85 (m, 2H), 1.78 – 1.61 (m, 8H), 1.56 – 1.37 (m, 4H).

Green synthesis of MenLp$_1$

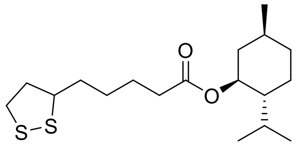

Lipoic acid (3.0 g, 1.2 equiv., 14.5 mmol), menthol (1.89 g, 1 equiv., 12.1 mmol) and *p*-toluenesulfonic acid (PTSA) (0.048 g, 0.02 equiv., 0.25 mmol) were placed in a two-neck round-bottom flask and connected to a condenser under N_2 atmosphere. The flask was wrapped in aluminum foil to protect from ambient light and stirred for a certain time at 100 or 140 °C. After the pre-determined time, the reaction mixture was poured into a round-bottom flask containing cold MeTHF (−78 °C), purified using a silica/basic alumina plug and concentrated *in vacuo* to afford a yellow oil (yield = 2.93 g, 70%). ^{1}H NMR (400 MHz, $CDCl_3$) δ 4.67 (td, *J* = 10.9, 4.4 Hz, 1H), 3.56 (dq, *J* = 8.3, 6.4 Hz, 1H), 3.24 – 3.00 (m, 2H), 2.40 – 2.49 (m, 1H), 2.29 (t, *J* = 7.4 Hz, 2H), 2.02 – 1.77 (m, overlapping, 3H), 1.75 – 1.56 (m, overlapping, 6H), 1.56 – 1.30 (m, overlapping, 4H), 1.11 – 0.77 (m, overlapping, 9H), 0.75 (d, *J* = 7.0 Hz, 3H).

Green synthesis of MenLp$_1$:IsoLp$_2$ (30:70) resin in one-pot.

Lipoic acid (4.0 g, 1 equiv., 19.6 mmol), isosorbide (0.96 g, 0.33 equiv., 6.6 mmol), menthol (0.66 g, 0.21 equiv., 4.2 mmol) and *p*-toluenesulfonic acid (PTSA) (0.056 g, 0.014 equiv., 0.29 mmol) were placed in a two-neck round-bottom flask and connected to a condenser under N_2 atmosphere. The flask was wrapped in aluminum foil to protect from ambient light and stirred for 6 h at 140 °C. After 6 h, a solution containing pyrogallol (2.2 wt.% in MeTHF) was added dropwise to the reaction mixture, which was then poured into a round-bottom flask containing cold methyl THF (−78 °C). The crude was purified using a silica/basic alumina plug and concentrated *in vacuo* to afford the resin mixture as a yellow oil (yield = 2.9 g, 59%).

<u>2D-photocuring resin preparation</u>

Resins were prepared by adding 70 wt.% of crosslinker (IsoLp$_2$ or GlyLp$_3$), 30 wt.% of linear diluent (R-Lp$_1$) and 1 wt.% ethyl (2,4,6-trimethylbenzoyl) phenylphosphinate (TPOL) into a 50 mL reagent bottle with screw cap equipped with a magnetic stir bar. The resin was stirred for 1 h at ambient temperature to reach homogeneity before curing.

<u>3D-printing resin preparation</u>

Resins were prepared by adding 70 wt.% of crosslinker (IsoLp$_2$ or GlyLp$_3$) and 30 wt.% of linear diluent (MenLp$_1$ or EtLp$_1$) into a 50 mL reagent bottle with screw cap equipped with a magnetic stir bar. The resin was stirred for 1 h to reach homogeneity.

Recycled resins were prepared by adding the recovered resin mixture into a 50 mL reagent bottle with screw cap equipped with a magnetic stir bar and 2 wt.% of phenylbis(2,4,6-trimethylbenzoyl)phosphine oxide (BAPO) was added to the mixture. The recycled resin was stirred for 1 h to ensure BAPO dissolution and provide a homogeneous mixture.

<u>Depolymerization experiments (catalyzed method with phosphazene:thiophenol)</u>

Post-cured 2D-photosets (*ca.* 0.2 g) were cut up and ground with mortar and pestle and then added to a 20 mL scintillation vial. MeTHF was added to the vial to yield a suspension with disulfide concentration of 0.25 M (1 equiv.). The suspension was stirred for 15 min. and then a solution containing thiophenol (0.1 equiv.) and P$_1$-t-Bu (0.1 equiv.) was added with a syringe. The

reaction mixture was placed in an oil bath at 80 °C and the depolymerization was carried out for 3 h. The reaction was quenched with TFA (0.2 equiv.) and the system was stirred for 15 min at 80 °C after TFA addition. The solvent was removed under reduced pressure to afford the crude recycled product.

3D-printed objects (*ca*. 15-20 g) were ground with mortar and pestle and then added to a 250 mL single neck round-bottom flask. MeTHF was added to the flask to yield a suspension with disulfide concentration of 0.5 M (1 equiv.). The suspension was stirred for 15 min. and then a solution containing thiophenol (0.01 equiv.) and P_1-t-Bu (0.01 equiv.) was added with a syringe. The reaction mixture was placed in an oil bath at 80 °C and stirred for 3 h. The reaction mixture was quenched with TFA (0.1 equiv.), which was added to the round-bottom flask with a syringe and the system was stirred for 15 min at 80 °C after TFA addition. The crude product was diluted in MeTHF (*ca*. 100 mL) and filtered through a pad of basic alumina. The solvent was removed under reduced pressure to afford the recycled resin.

<u>Depolymerization experiments (catalyzed method with DBU:DTT)</u>

3D-printed objects (*ca*. 15-20 g) were ground with mortar and pestle and then added to a 250 mL single neck round-bottom flask. MeTHF was added to the flask to yield a suspension with disulfide concentration of 0.5 M (1 equiv.). The suspension was stirred for 15 min. and then a solution containing DBU (0.1 equiv.) and another containing DTT (0.01 equiv.) were added with a syringe. The reaction mixture was placed in an oil bath at 80 °C and stirred for 3 h. The reaction mixture was quenched with TFA (0.2 equiv.), which was added to the round-bottom flask with a syringe and the system was stirred for 15 min at 80 °C after TFA addition. The crude product was diluted in MeTHF (*ca*. 100 mL) and filtered through a pad of basic alumina. The solvent was removed under reduced pressure to afford the recycled resin.

<u>Depolymerization experiments (thermal method with DMF and no catalyst)</u>

Post-cured 2D-photosets (*ca.* 0.3 g) were cut up and ground with mortar and pestle and then added to a 25 mL 3-neck round-bottom flask equipped with a condenser. DMF was added to the flask to yield a suspension with disulfide concentration of 0.1 M (1 equiv.). The flask was purged with N_2 and then the reaction mixture was placed in an oil bath and refluxed for 3 h to yield a yellow solution. The hot solution was poured into a beaker containing *ca.* 50 mL cold toluene (cooled in a dry-ice bath) and partially concentrated on a rotary evaporator. The solution was then washed with LiCl (3x10 mL) to remove residual DMF. The remaining organic phase was concentrated under vacuum to afford the recycled resin.

Post-cured 2D-photosets (*ca.* 15-20 g) were ground with mortar and pestle and then added to a 250 mL 3-neck round-bottom flask equipped with a condenser. DMF was added to the flask to yield a suspension with disulfide concentration of 0.1 M (1 equiv.). The flask was purged with N_2 and then the reaction mixture was placed in an oil bath and refluxed for 3 h to yield a yellow solution. The hot solution was poured into a beaker containing *ca.* 250 mL cold toluene (cooled in a dry-ice bath) and partially concentrated on a rotary evaporator. The solution was then washed with LiCl (3x50 mL) to remove residual DMF. The remaining organic phase was concentrated under vacuum to afford the recycled resin.

<u>One-pot hydrolytic depolymerization experiments</u>

3D-printed objects (0.15 g) or post-cured 2D-photosets (0.15 g) were ground with mortar and pestle and then added to a 20 mL Schlenk flask. NaOH (10 wt%) was added to the flask followed

by 5 ml of DMF and 1 mL of H_2O. The reaction mixture was placed in an oil bath and heated at reflux for 3 h. The reaction mixture was rapidly cooled in a dry-ice toluene bath and the solvents were removed under vacuum to afford the crude recycled resin.

One-pot hydrolytic depolymerization with set-up to collect volatile components

3D-printed objects (150 mg) were ground with mortar and pestle and then added to a 20 mL two-neck round bottom flask connected with a Dean-Stark apparatus. NaOH (10 wt%) was added to the flask followed by 5 ml of DMF and 1 mL of H_2O. The reaction mixture was placed in an oil bath and heated at reflux for 3 h. The reaction mixture was rapidly cooled in a dry-ice toluene bath and the solvents were removed under vacuum to afford the crude recycled resin. The fraction collected in the Dean-Stark trap was collected as a crude sample without further purification.

NMR spectra

<u>Lipoates</u>

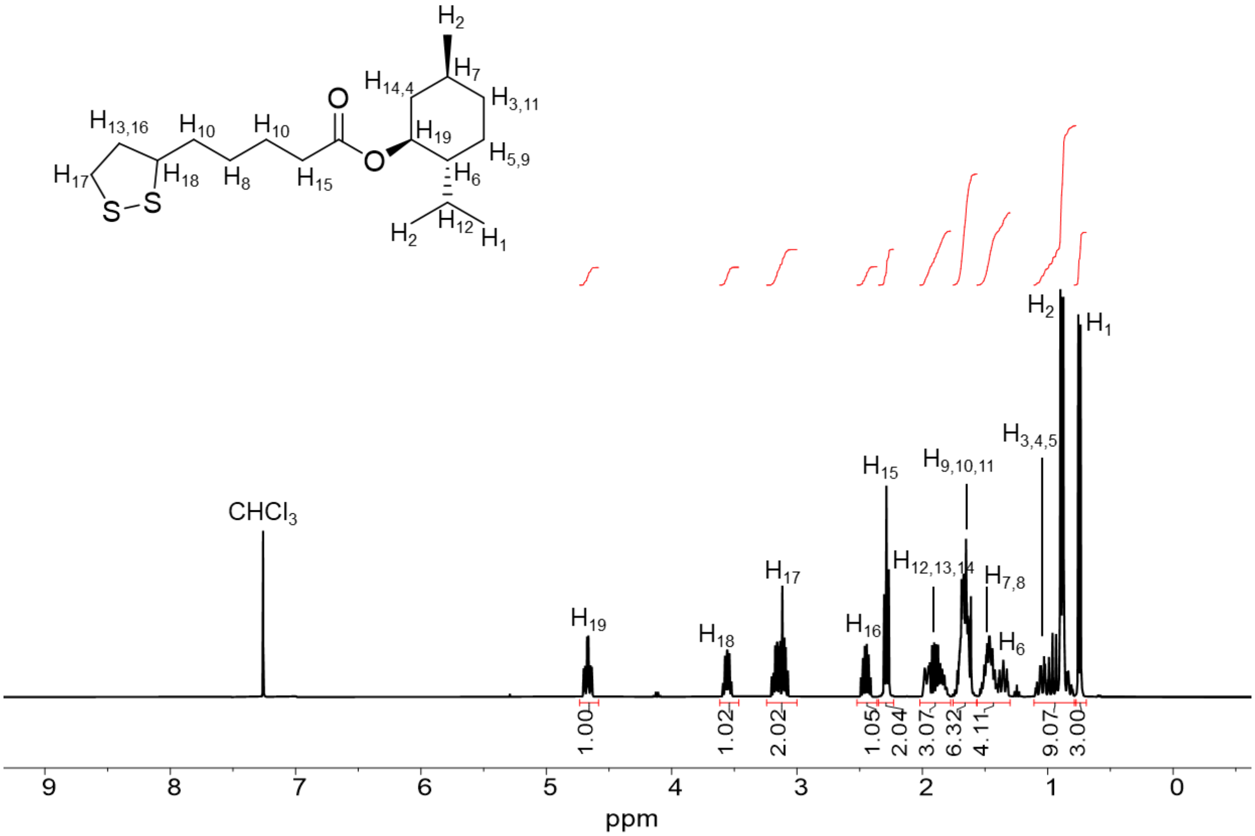

Fig. S1.

MenLp1 ^{1}H NMR Spectrum – 400 MHz, 298 K, $CDCl_3$.

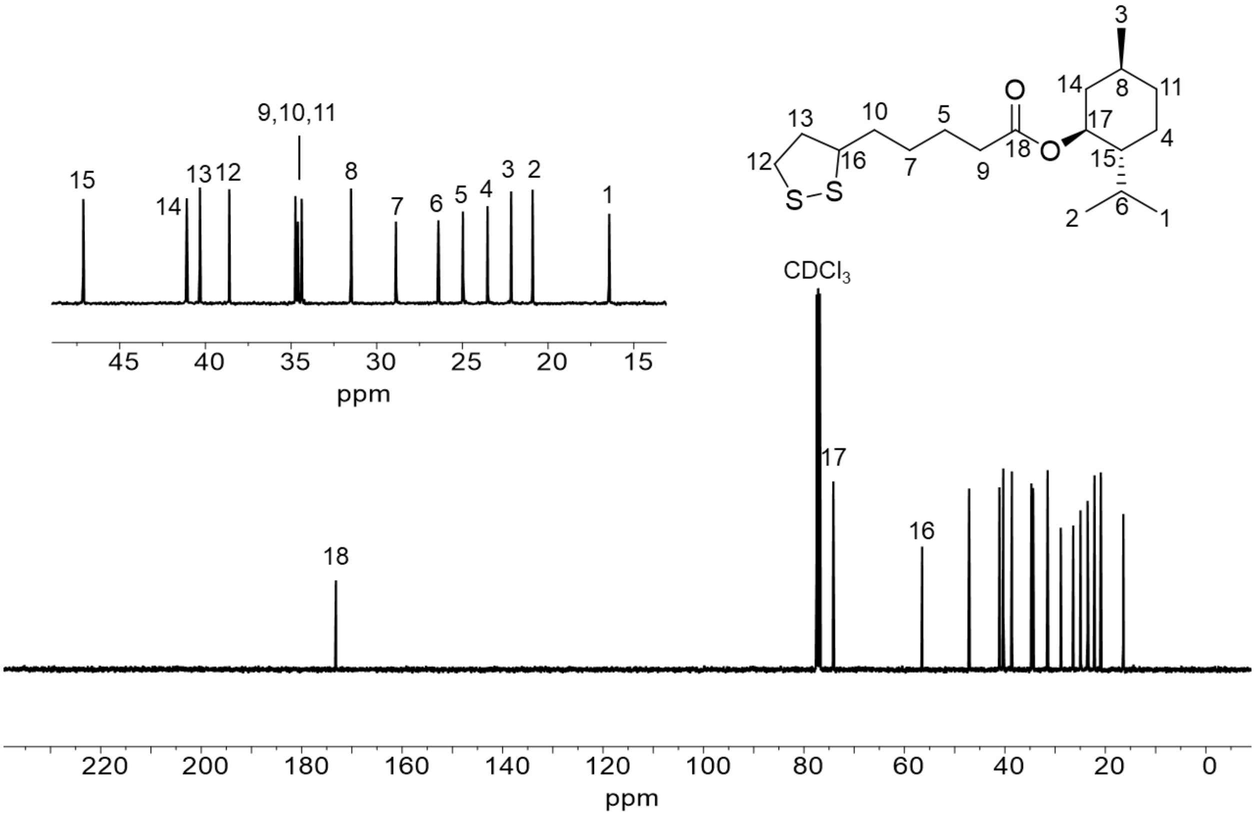

Fig. S2.

MenLp1 ^{13}C NMR Spectrum – 101 MHz, 298 K, $CDCl_3$.

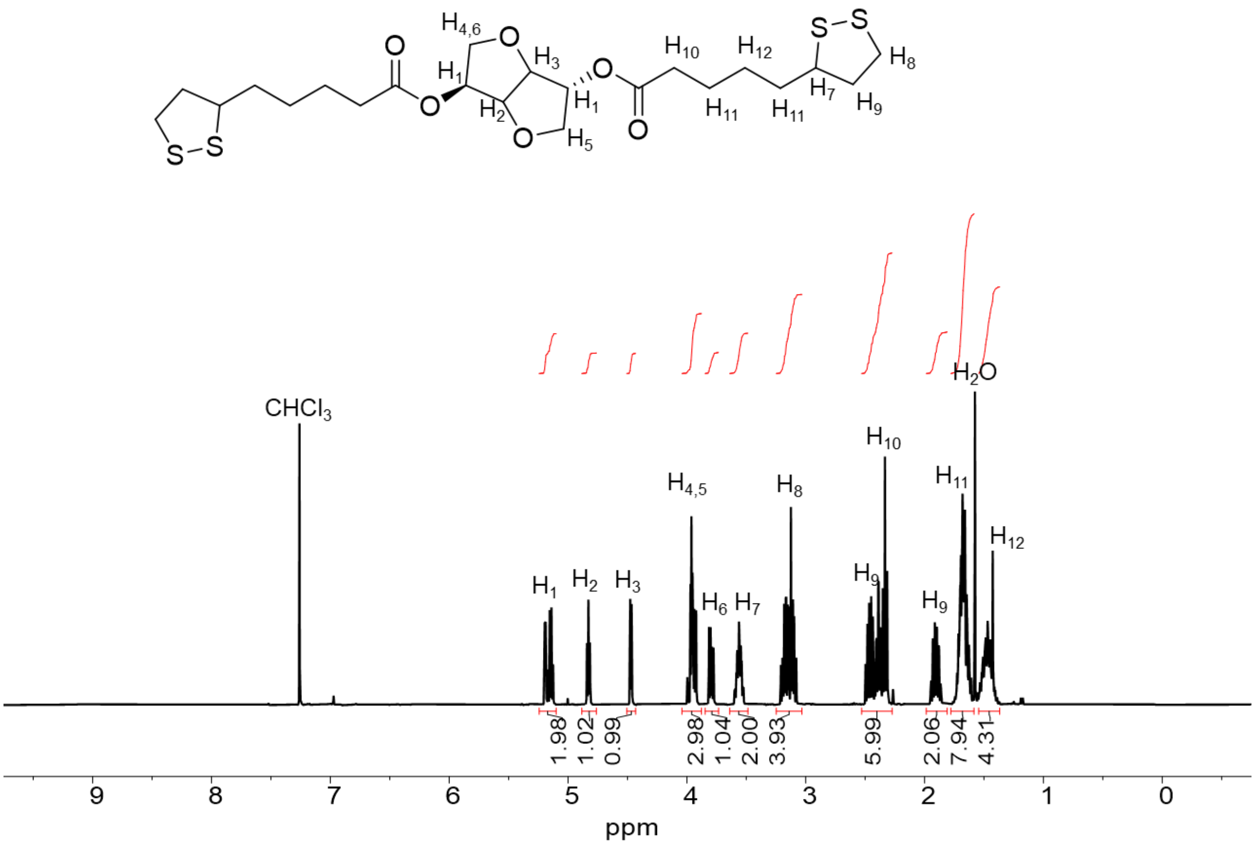

Fig. S3.

IsoLp2 ^{1}H NMR Spectrum – 400 MHz, 298 K, $CDCl_3$.

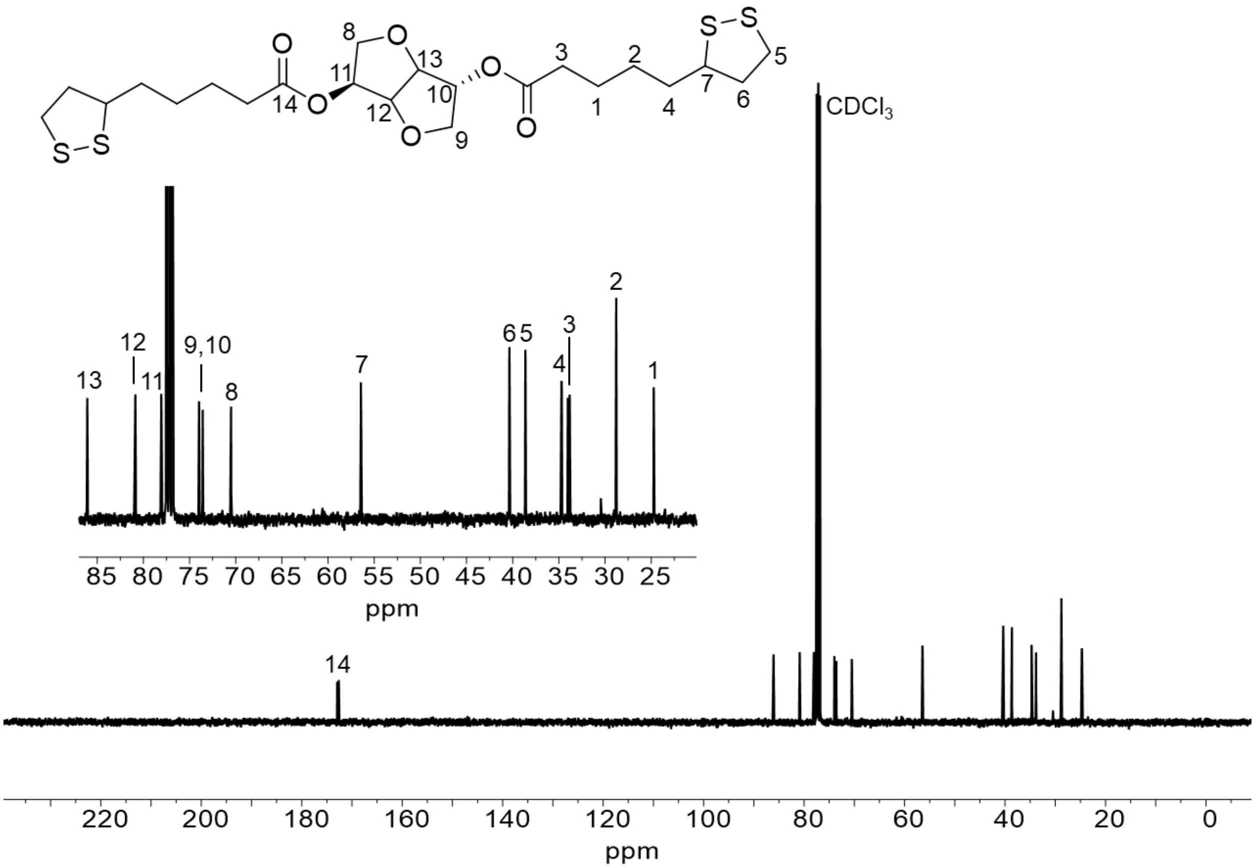

Fig. S4.
IsoLp2 ^{13}C NMR Spectrum – 101 MHz, 298 K, $CDCl_3$.

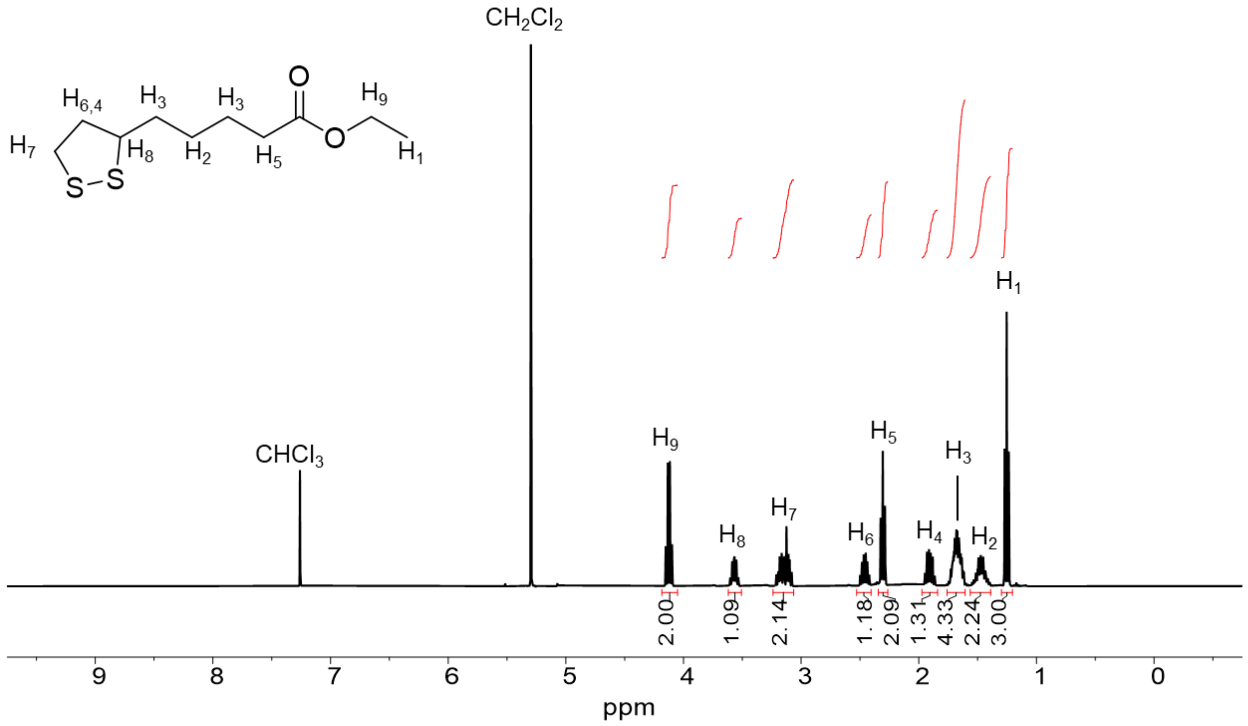

Fig. S5.

EtLp1 ^{1}H NMR Spectrum – 400 MHz, 298 K, $CDCl_3$.

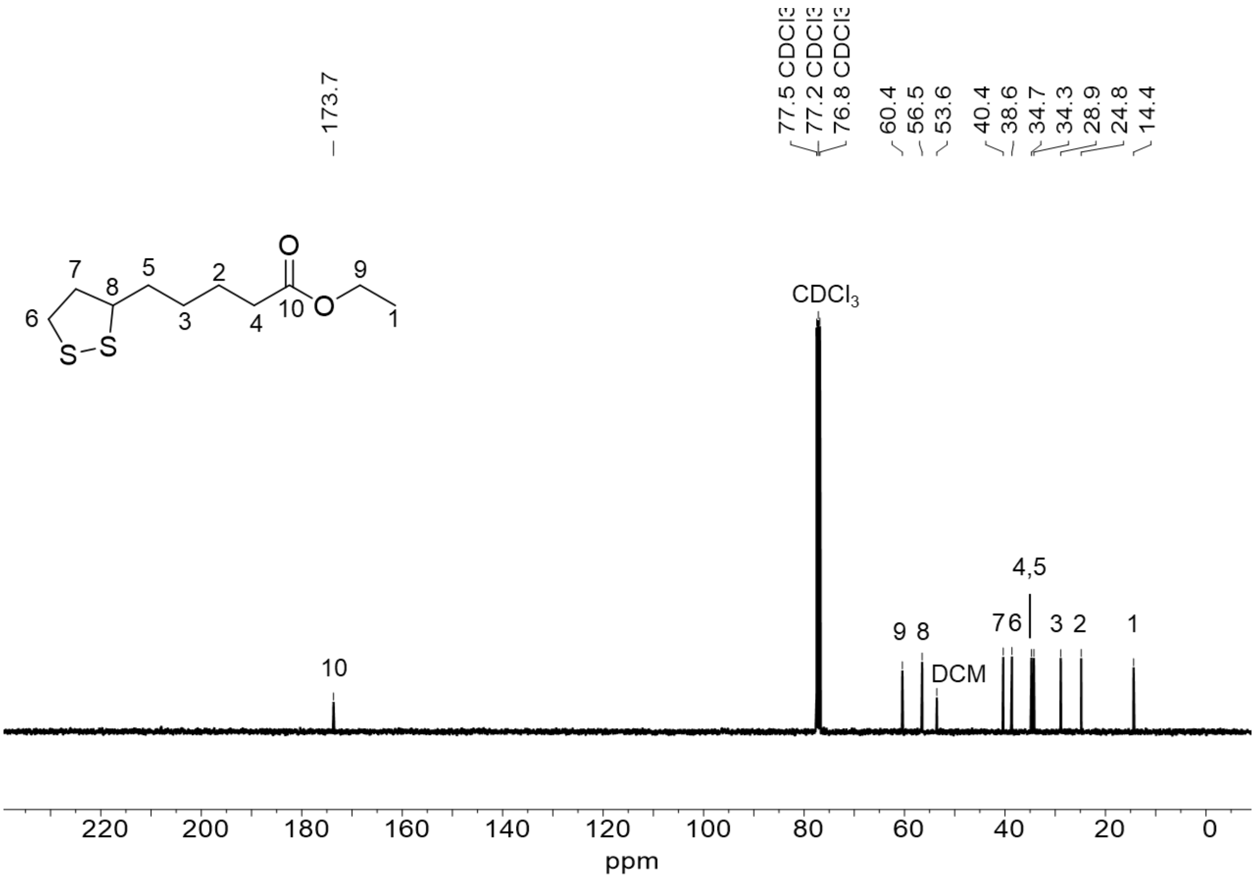

Fig. S6.

EtLp1 ^{13}C NMR Spectrum – 101 MHz, 298 K, $CDCl_3$.

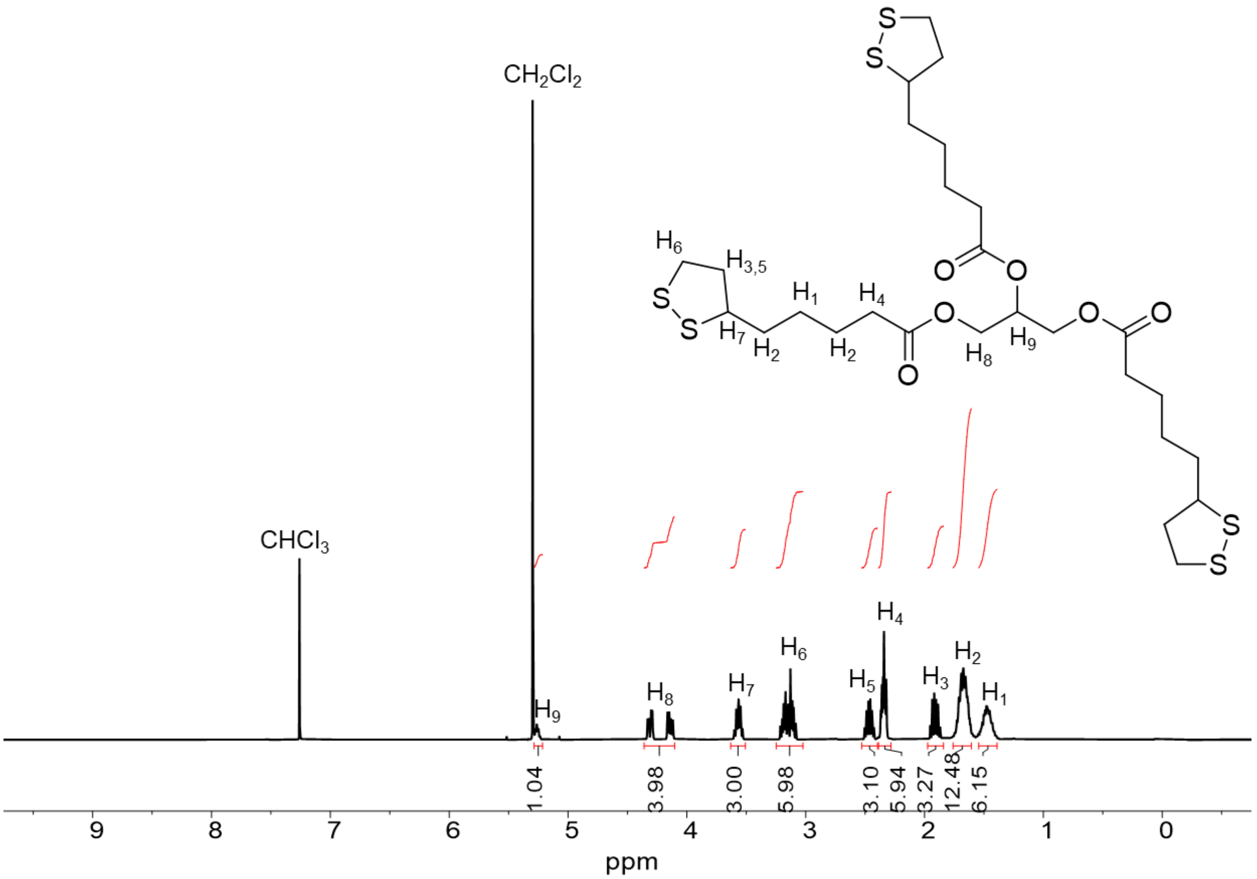

Fig. S7.

GlyLp3 ^{1}H NMR Spectrum – 400 MHz, 298 K, $CDCl_3$.

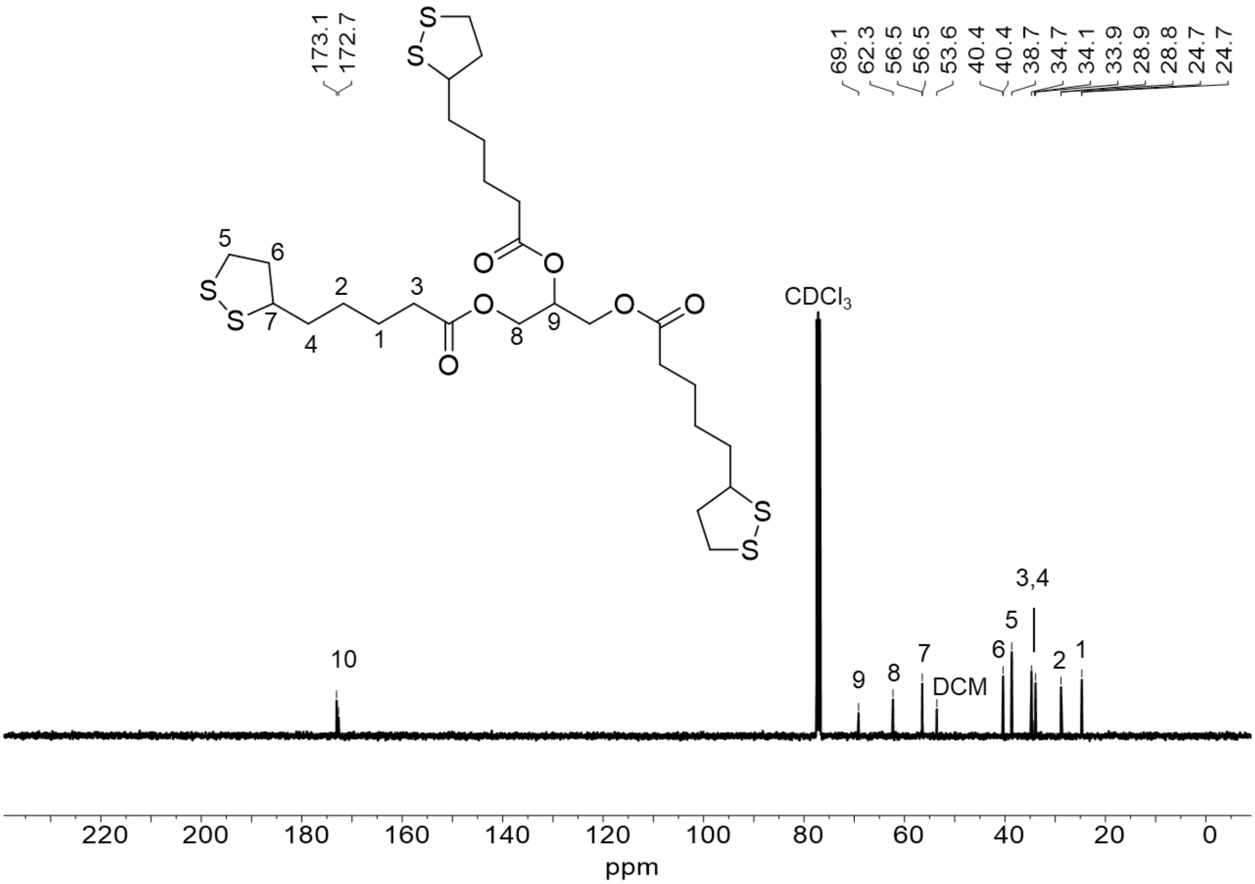

Fig. S8.

GlyLp3 ^{13}C NMR Spectrum – 101 MHz, 298 K, $CDCl_3$.

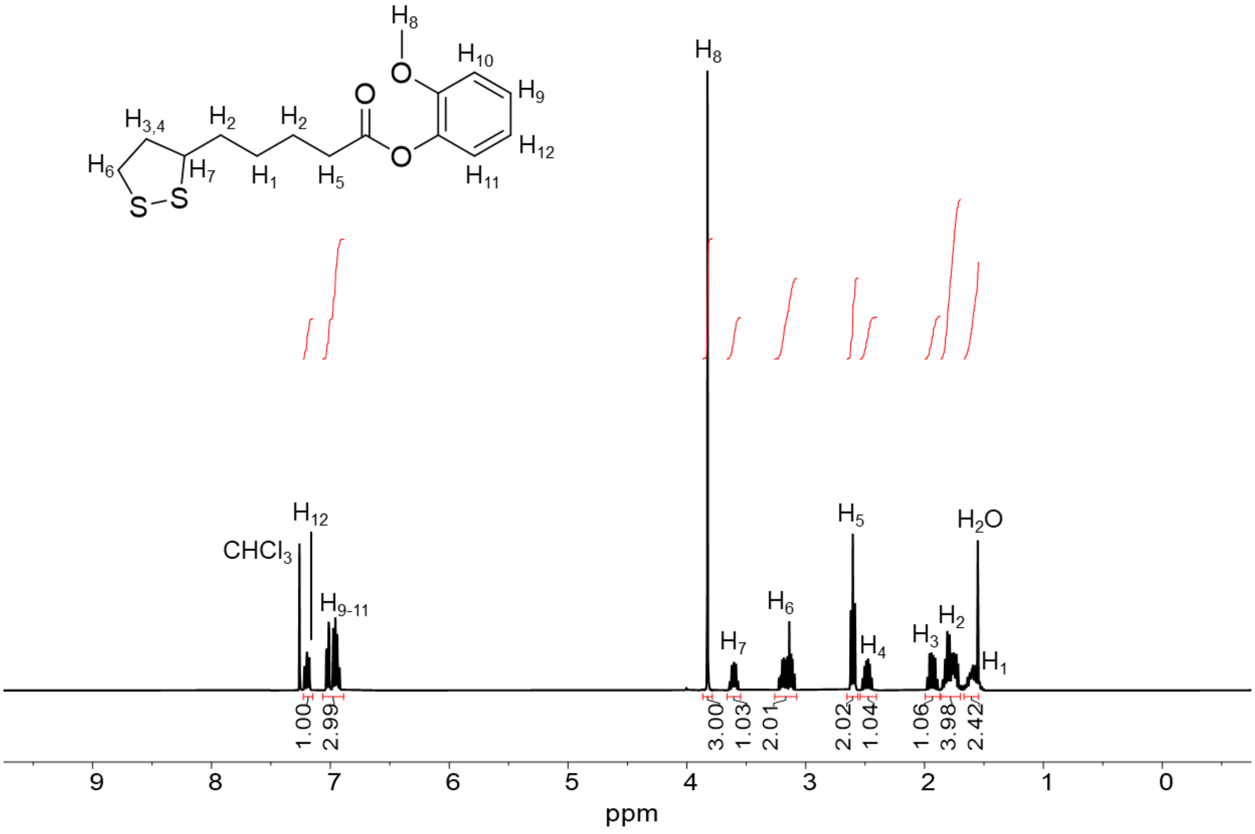

Fig. S9.

GuaLp1 ^{1}H NMR Spectrum – 400 MHz, 298 K, $CDCl_3$.

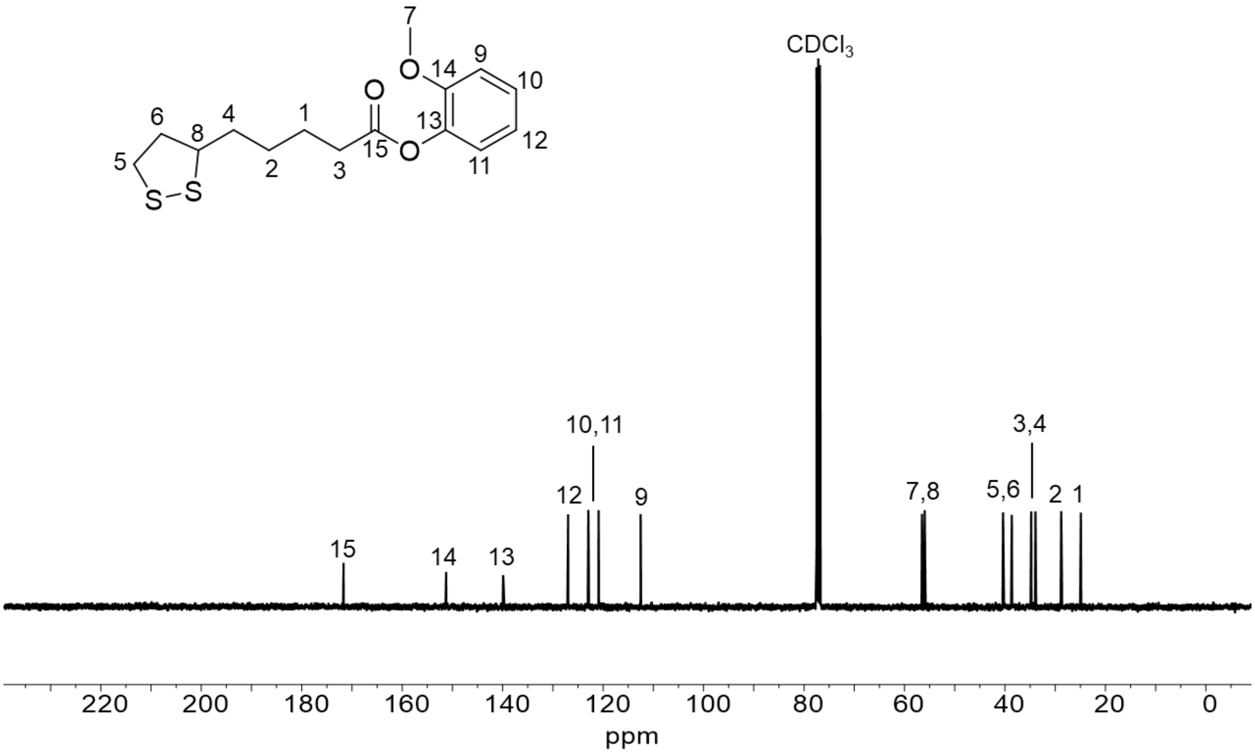

Fig. S10.

GuaLp1 ^{13}C NMR Spectrum – 101 MHz, 298 K, $CDCl_3$.

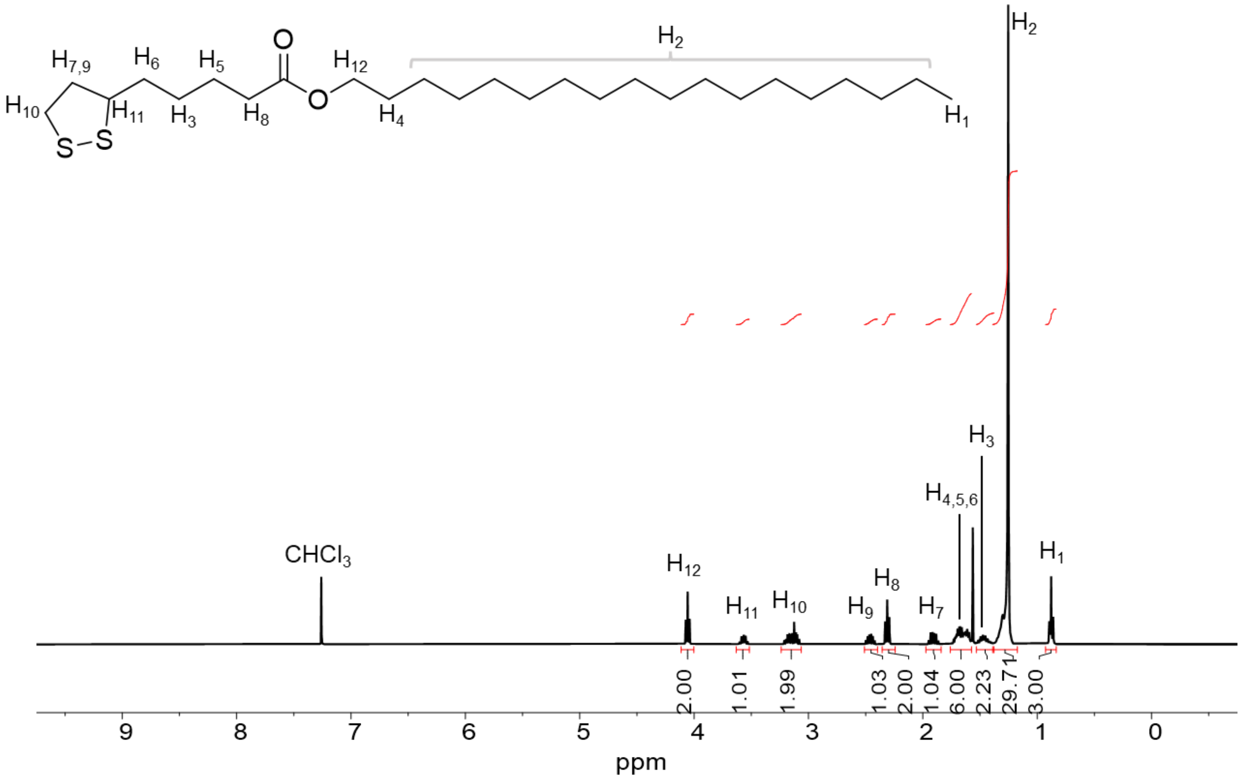

Fig. S11.

StearLp1 ^{1}H NMR Spectrum – 400 MHz, 298 K, $CDCl_3$.

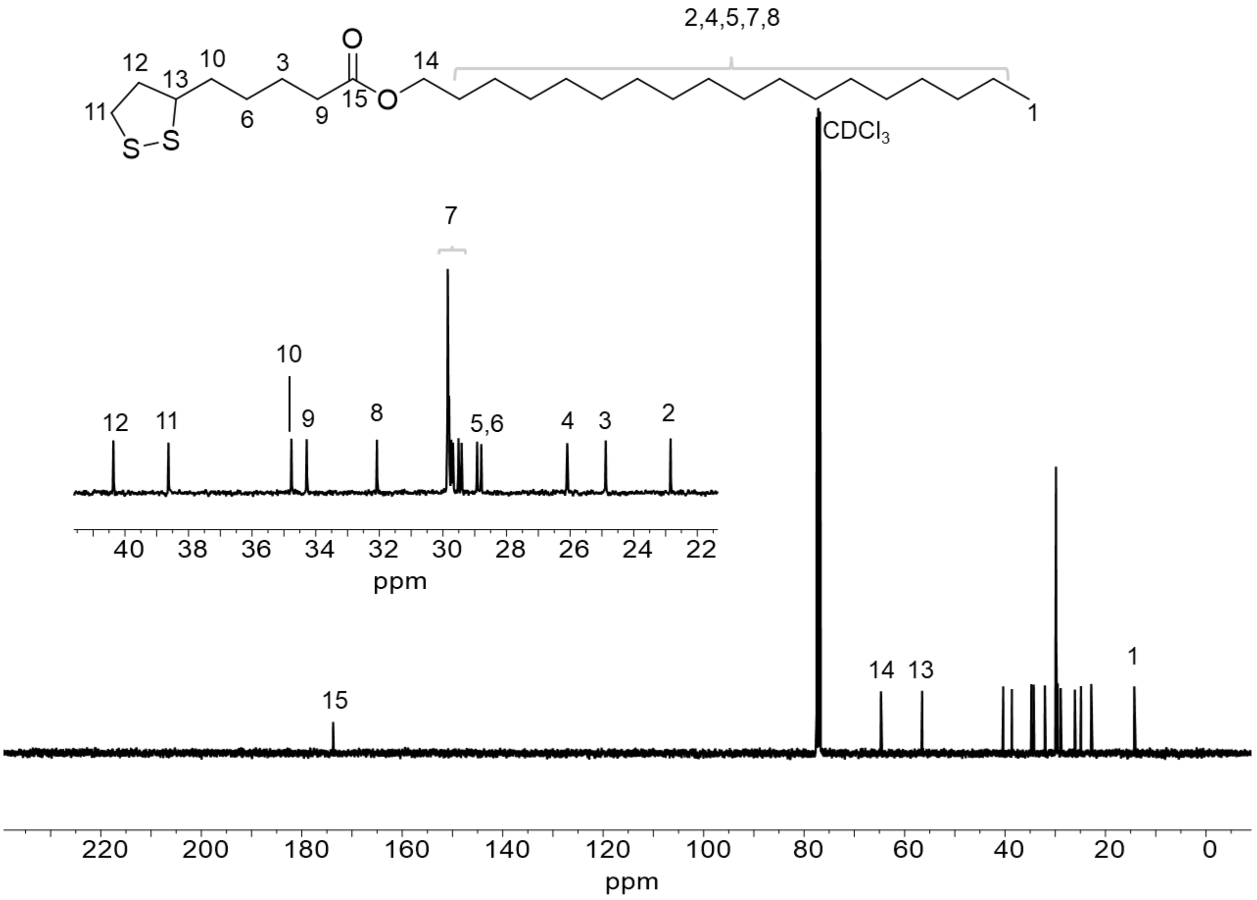

Fig. S12.

StearLp1 ^{13}C NMR Spectrum – 101 MHz, 298 K, $CDCl_3$.

Lipoates from green synthetic method

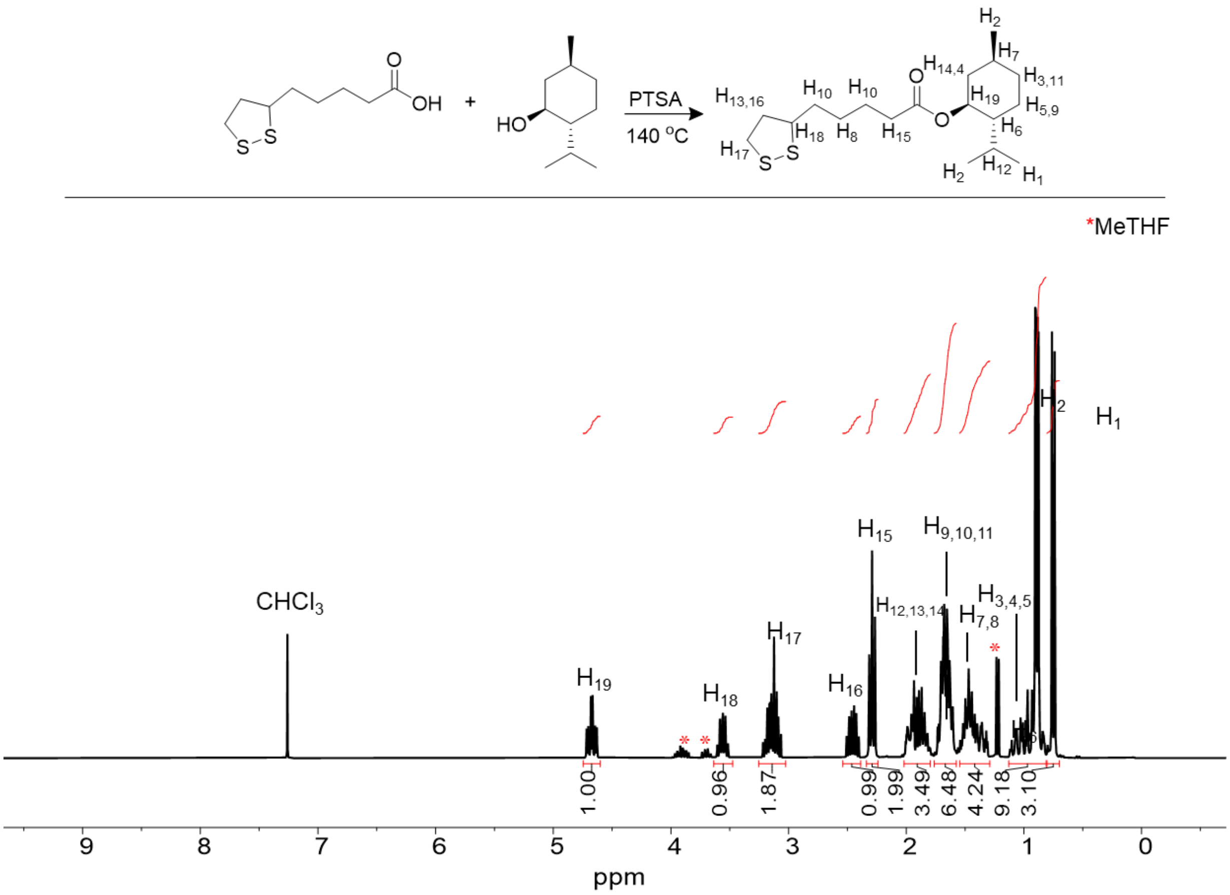

Fig. S13.

MenLp1 ^{1}H NMR Spectrum – 400 MHz, 298 K, $CDCl_3$ – obtained from green synthetic route.

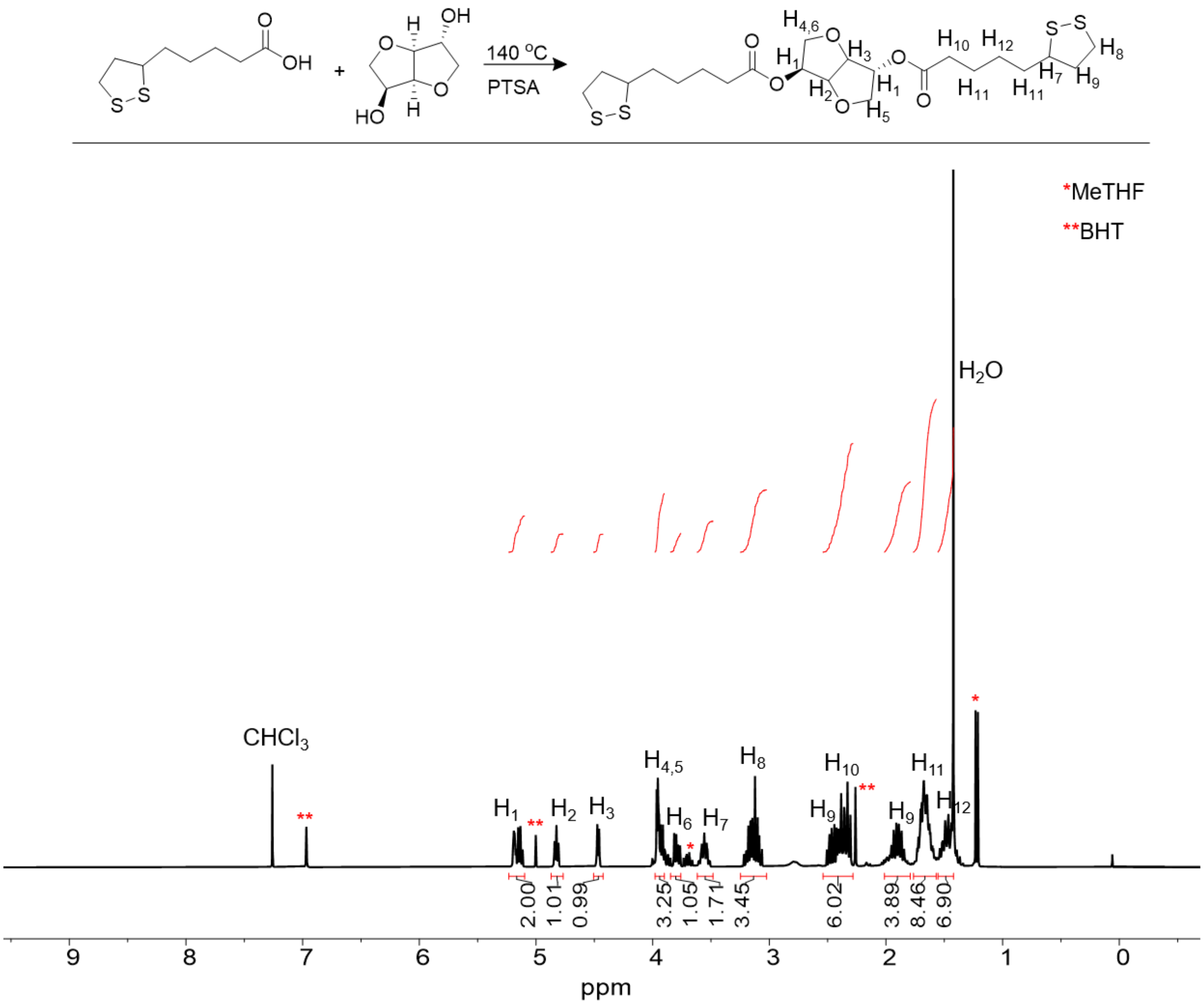

Fig. S14.

IsoLp2 ^{1}H NMR Spectrum – 400 MHz, 298 K, $CDCl_3$ – obtained from green synthetic route.

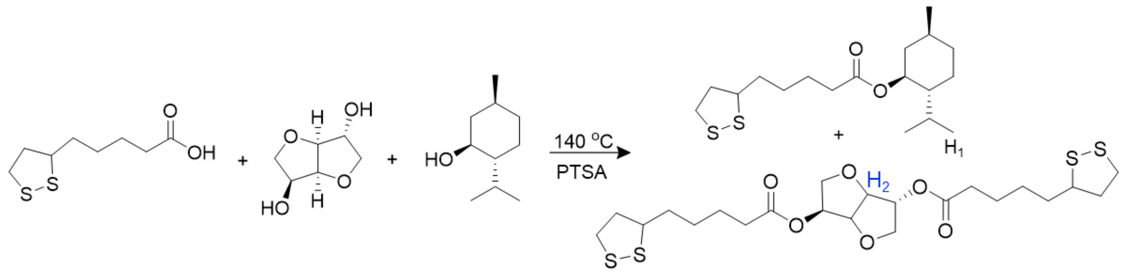

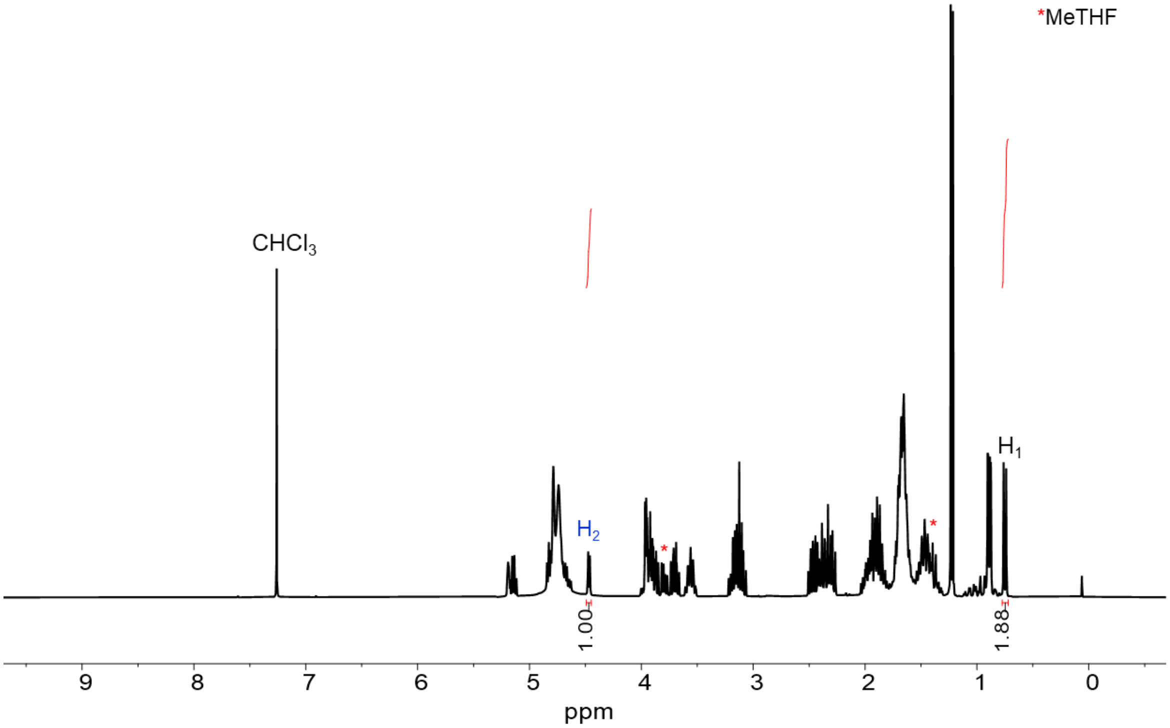

Fig. S15.

MenLp1:IsoLp2 (30:70 wt% or 40:60 mol%) ^{1}H NMR Spectrum – 400 MHz, 298 K, $CDCl_3$ – obtained from green synthetic route. *Reaction mixture yielded MenLp1-IsoLp2 (37:63 mol%) from relative integrals of H_1 (IsoLp2) and H_2 (MenLp1).

Table S1.

Summary of experimental screening conditions for green synthesis of lipoates.

Entry	Alcohol	Alcohol:LA mol equiv	PTSA (wt.%)	Temp. (°C)	Time (h)	Conversion (%)	Yield (%)
1	Menthol	1:1.2	-	140	15	66	33
2	Menthol	1:1.2	-	100	15	16	NR
3	Menthol	1:1.2	1	140	5	100	70
4[a]	Isosorbide	1:2.2	1	140	7	100	66
5[b]	Menthol/ Isosorbide	1/0.21:0.33	1	140	6	100	59

All reactions were cooled down by pouring the reaction mixture directly into cold (−78 °C) MeTHF, followed by purification via silica/basic alumina plug. [a]BHT or [b]pyrogallol was added into the reaction mixture before cooling down to inhibit cross-linking reactions of the resin during reaction work-up.

Recycled resins from 2D-photosets

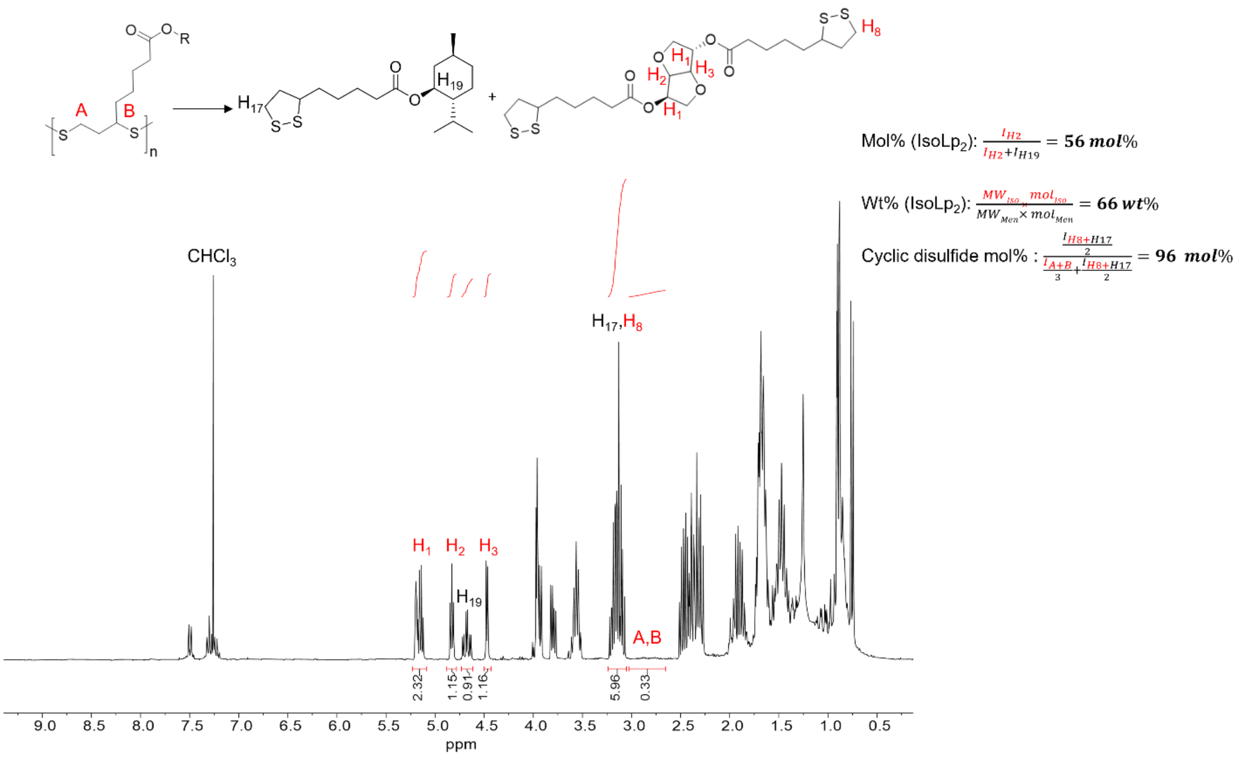

Fig. S16.

Catalyzed depolymerization (phosphazene:thiophenol method) of MenLp1:IsoLp2 (30:70 wt%) 2D-photoset ^{1}H NMR Spectrum – 400 MHz, 298 K, $CDCl_3$.

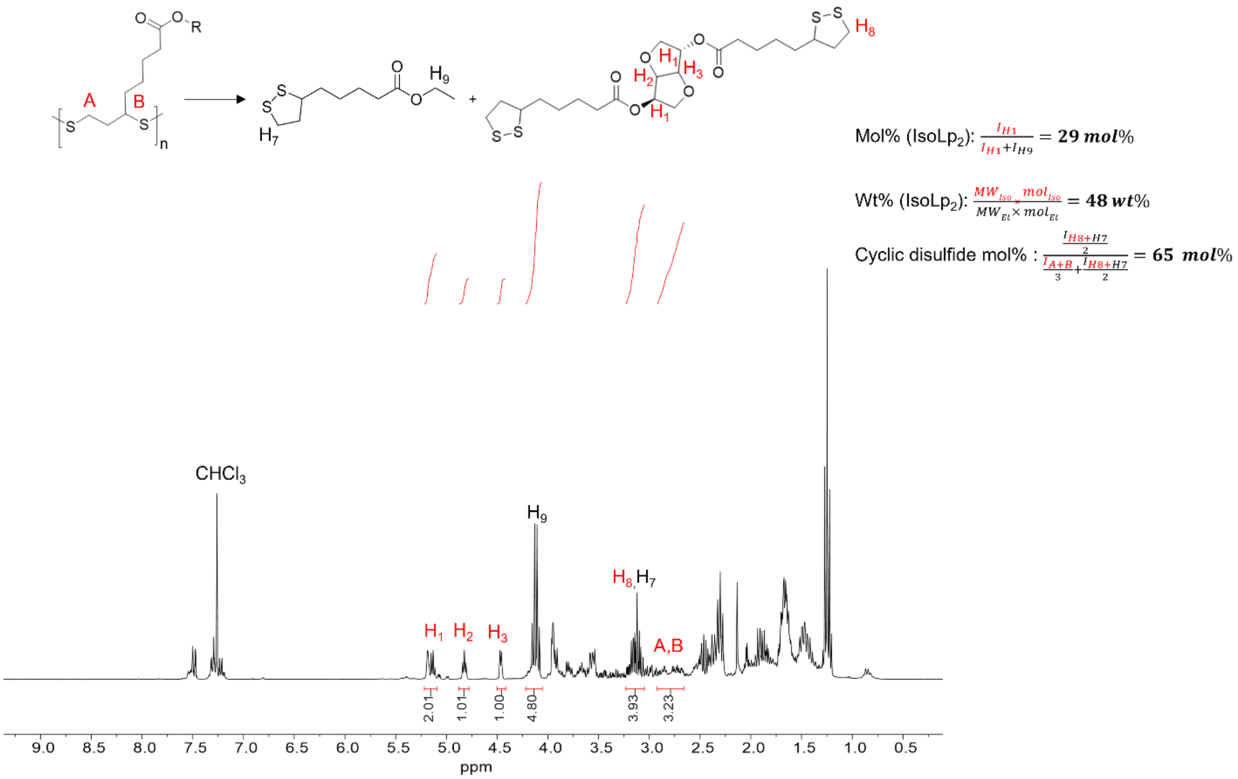

Fig. S17.

Catalyzed depolymerization (phosphazene:thiophenol method) of EtLp1:IsoLp2 (30:70 wt%) 2D-photoset ^{1}H NMR Spectrum – 400 MHz, 298 K, $CDCl_3$.

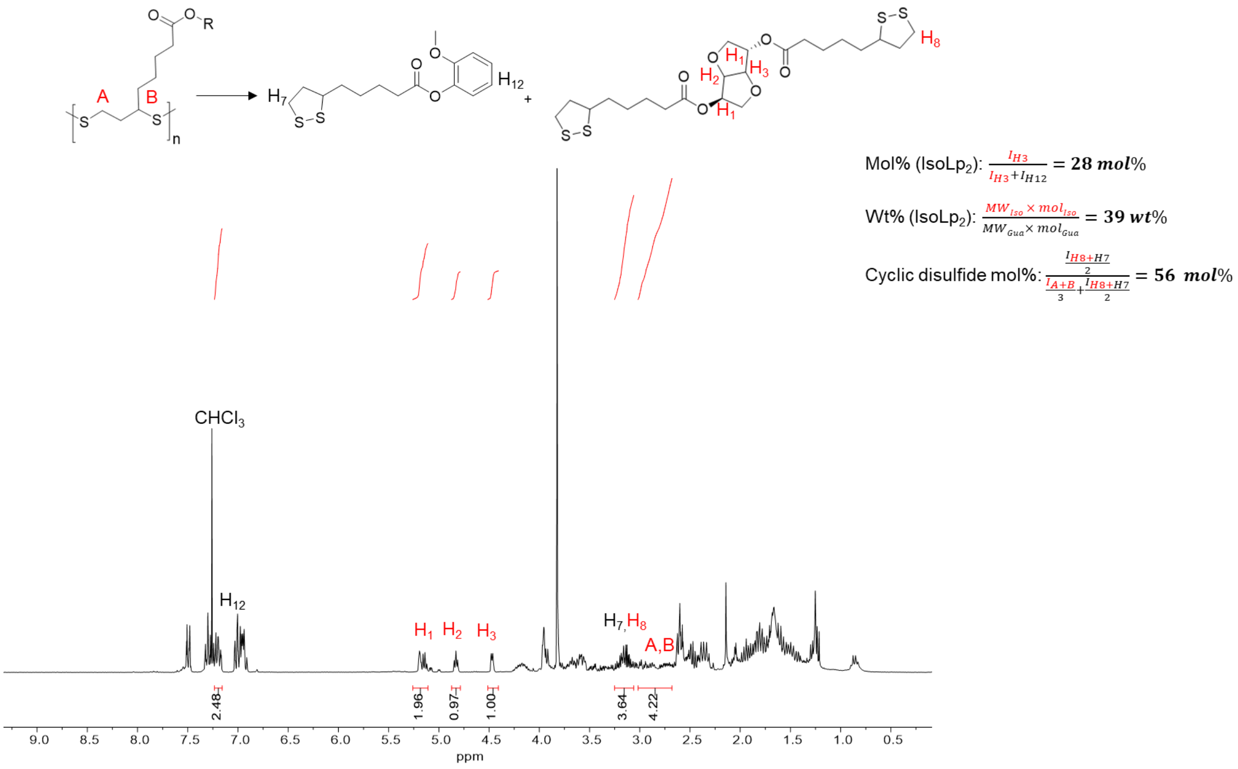

Fig. S18.

Catalyzed depolymerization (phosphazene:thiophenol method) of GuaLp1:IsoLp2 (30:70 wt%) 2D-photoset ^{1}H NMR Spectrum – 400 MHz, 298 K, $CDCl_3$.

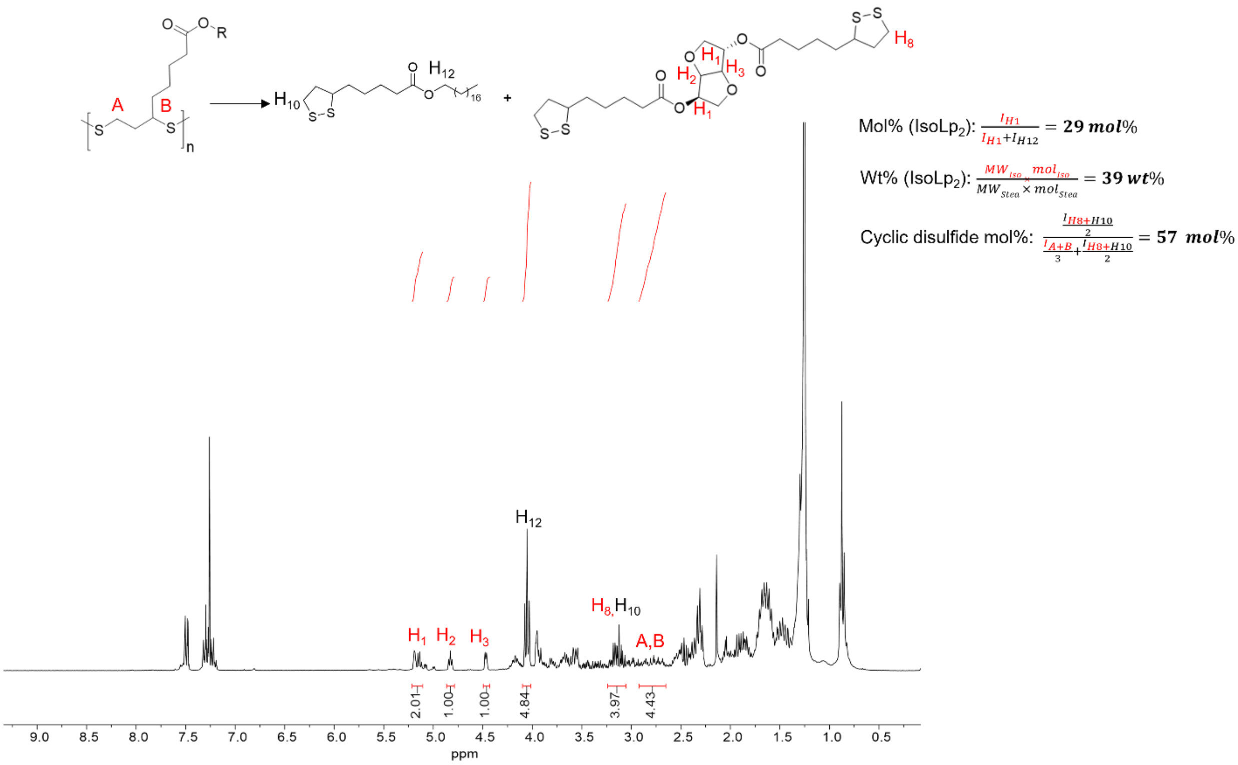

Fig. S19.

Catalyzed depolymerization (phosphazene:thiophenol method) of SteaLp1:IsoLp2 (30:70 wt%) 2D-photoset ^{1}H NMR Spectrum – 400 MHz, 298 K, $CDCl_3$.

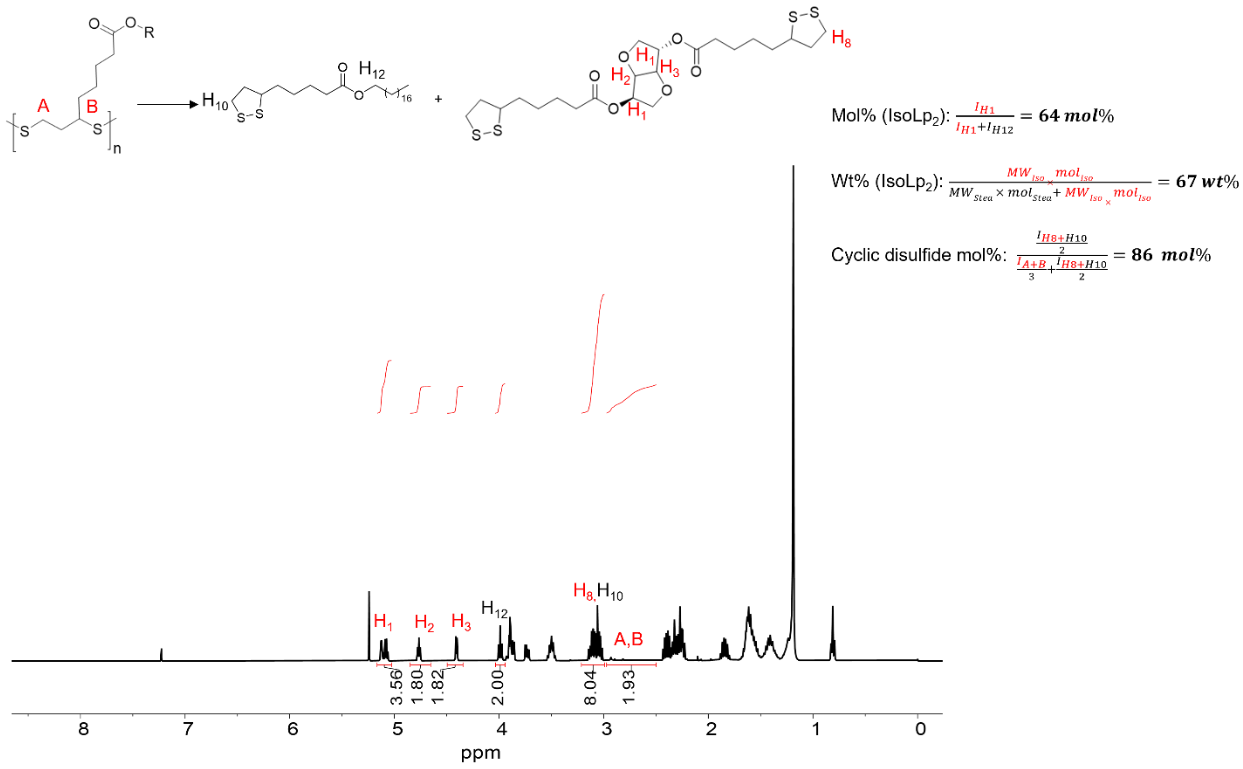

Fig. S20.
Thermal depolymerization (DMF method) of SteaLp1:IsoLp2 (30:70 wt%) 2D-photoset ^{1}H NMR Spectrum – 400 MHz, 298 K, $CDCl_3$.

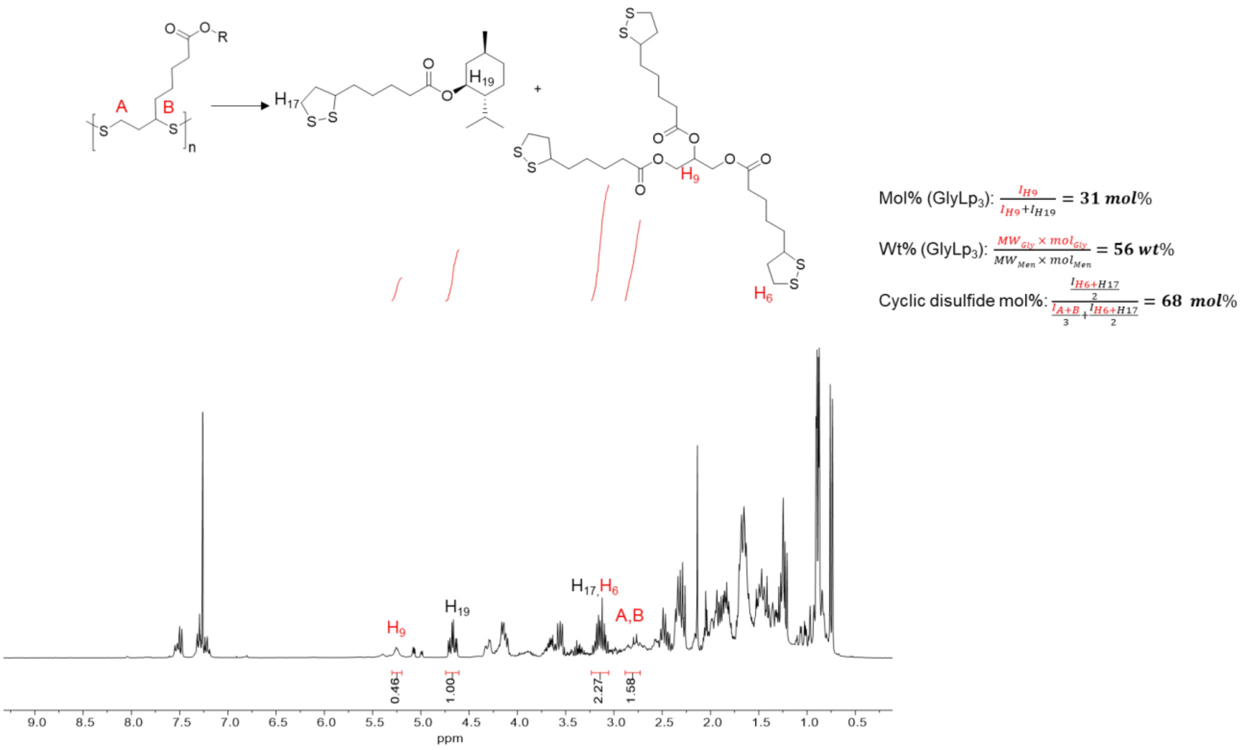

Fig. S21.

Catalyzed depolymerization (phosphazene:thiophenol method) of $MenLp_1$:$GlyLp_3$ (30:70 wt%) 2D-photoset ^{1}H NMR Spectrum – 400 MHz, 298 K, $CDCl_3$.

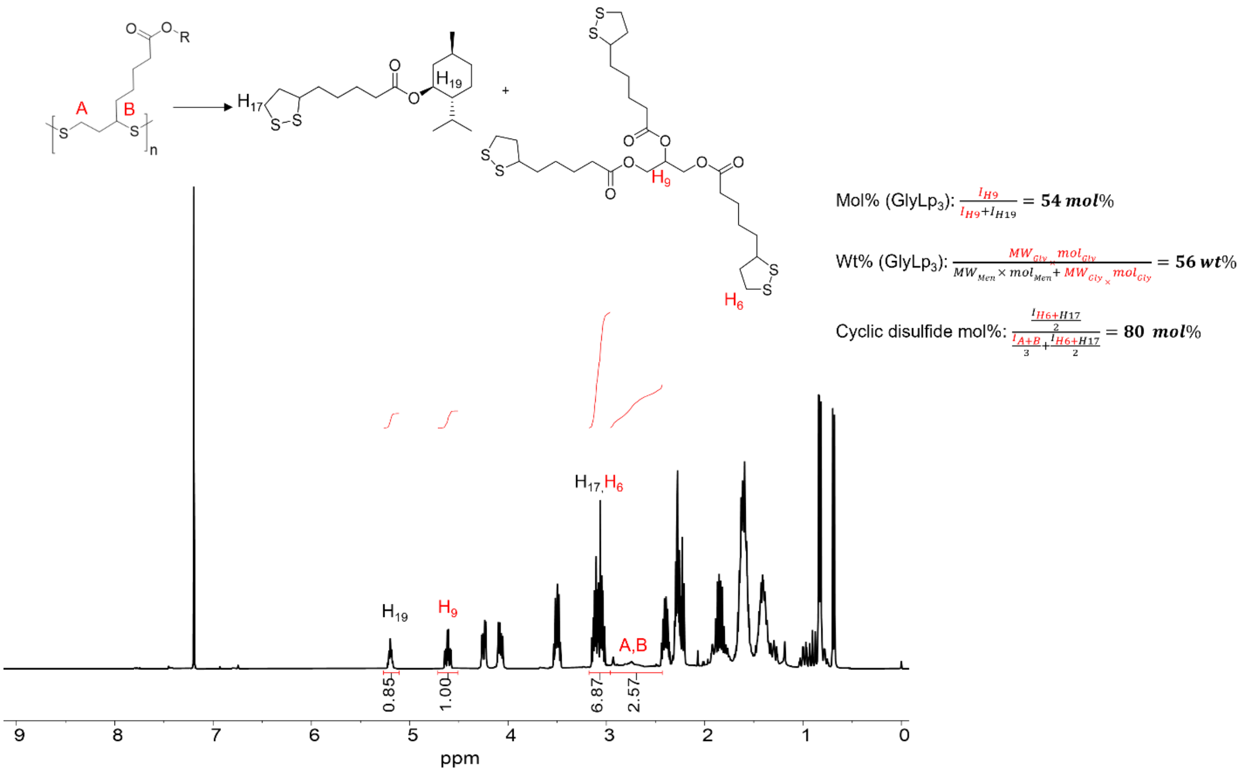

Fig. S22.
Thermal depolymerization (DMF method) of MenLp1:GlyLp3 (33:67 wt%) 2D-photoset ^{1}H NMR Spectrum – 400 MHz, 298 K, $CDCl_3$.

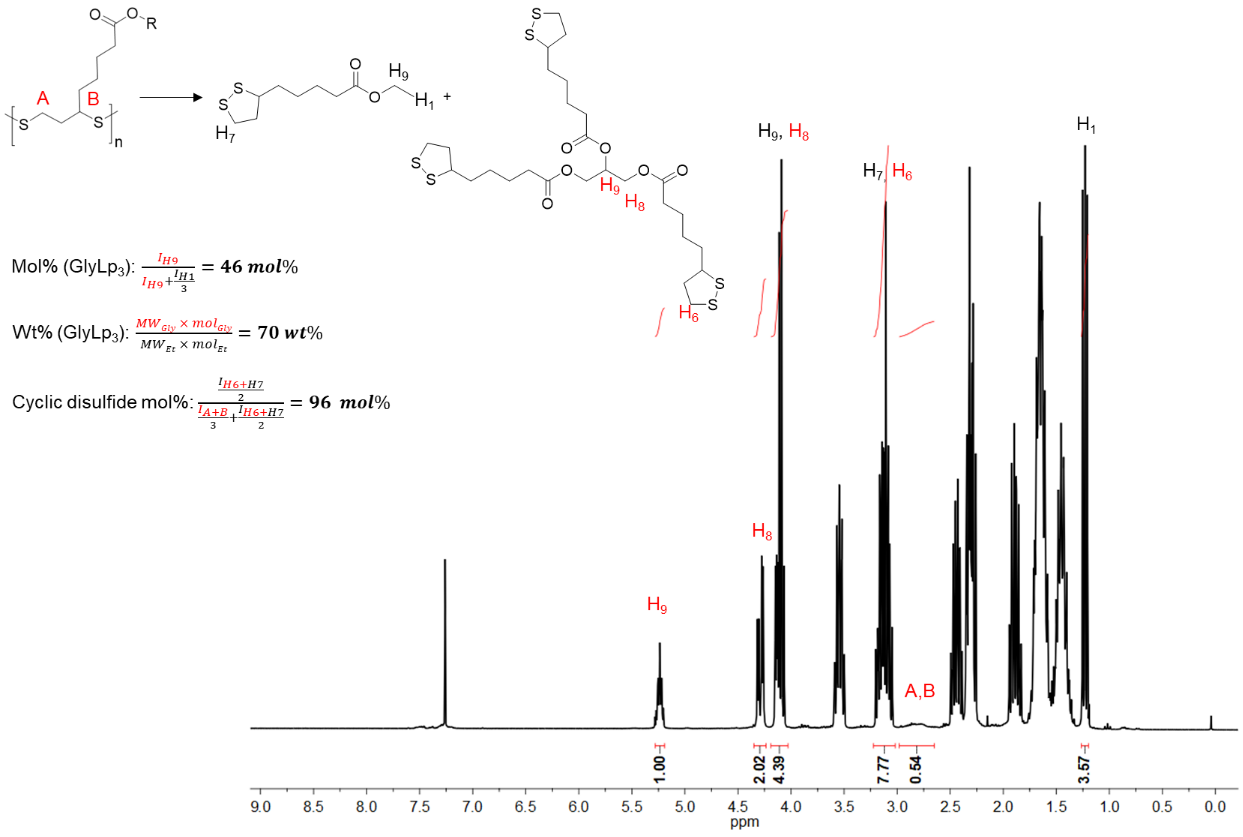

Fig. S23.

Catalyzed depolymerization (phosphazene:thiophenol method) of $EtLp_1$:$GlyLp_3$ (30:70 wt%) 2D-photoset ^1H NMR Spectrum – 400 MHz, 298 K, $CDCl_3$.

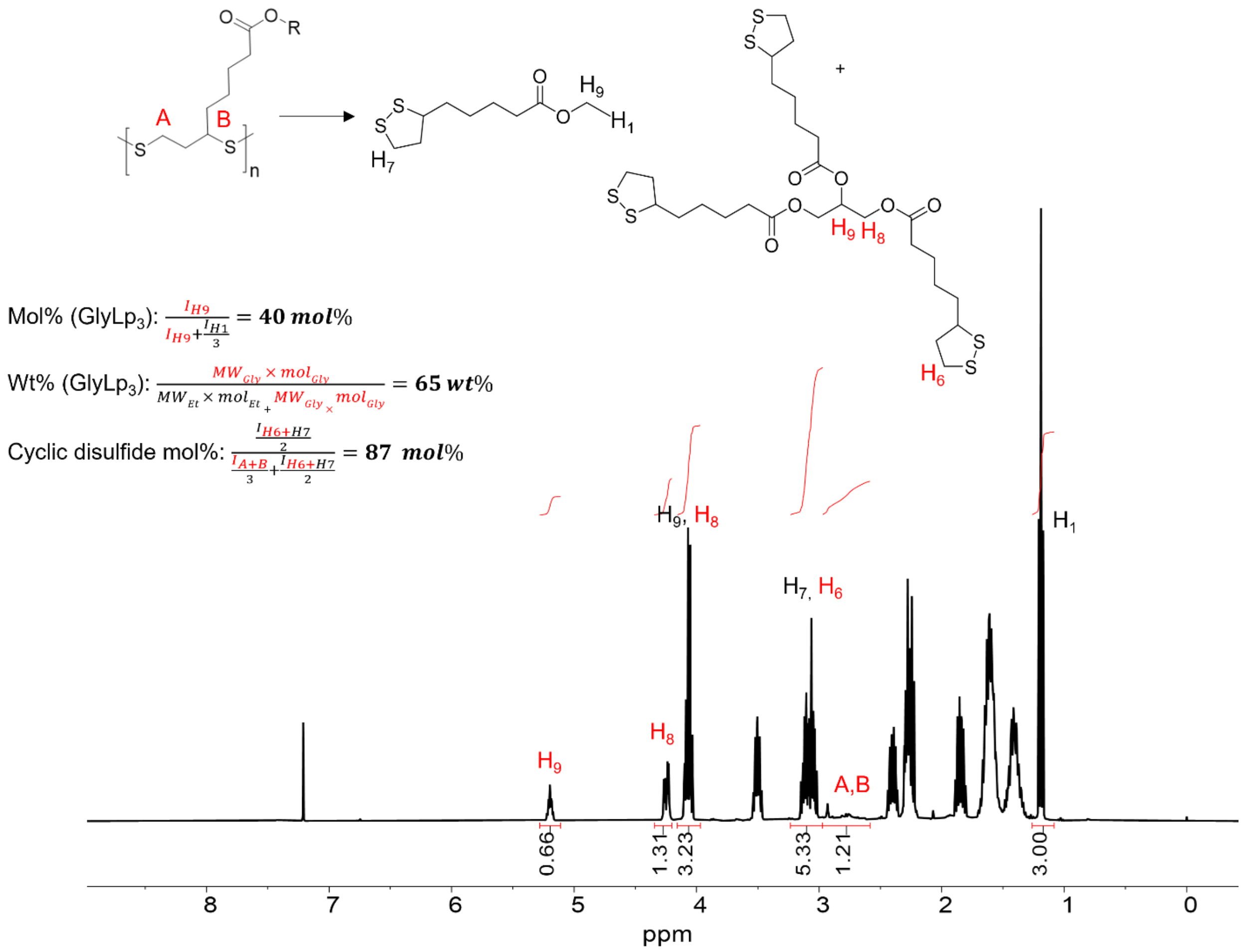

Fig. S24.
Thermal depolymerization (DMF method) of $EtLp_1{:}GlyLp_3$ (34:66 wt%) 2D-photoset ^1H NMR Spectrum – 400 MHz, 298 K, $CDCl_3$.

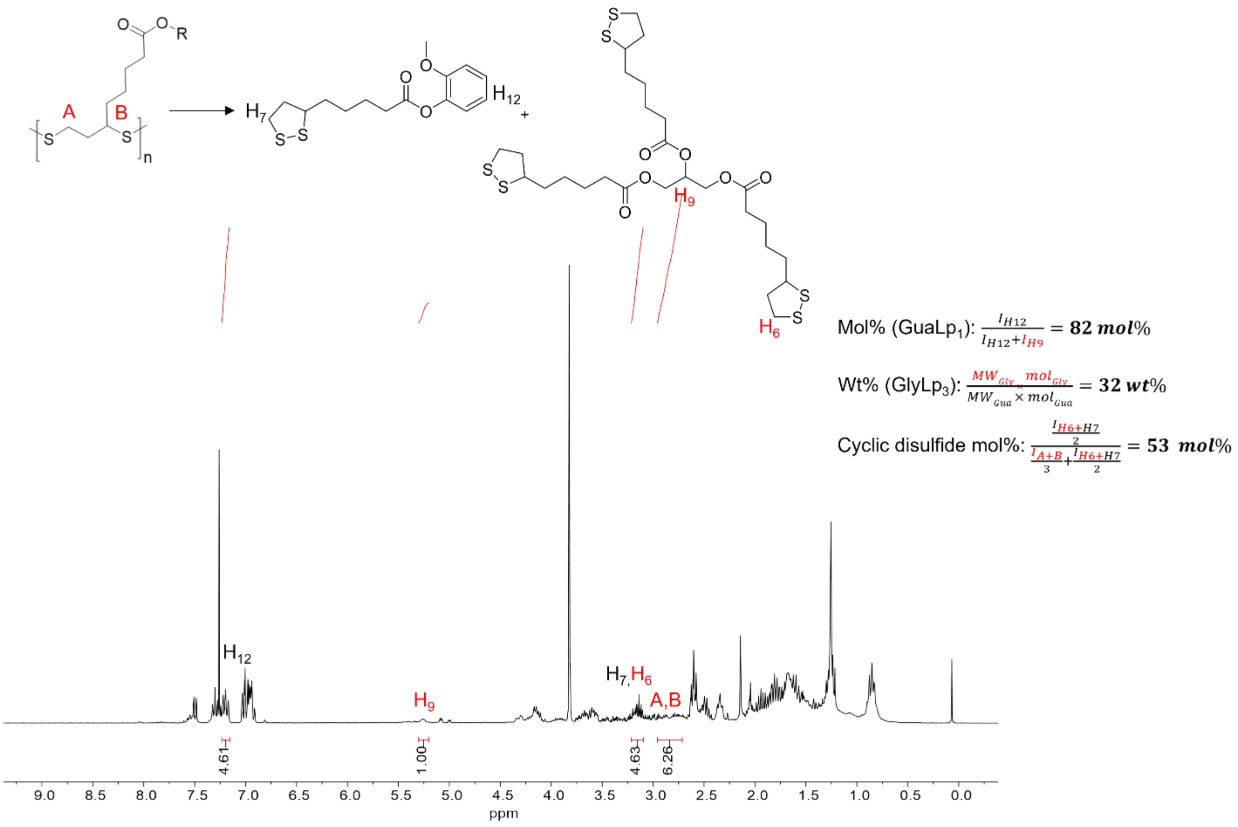

Fig. S25.

Catalyzed depolymerization (phosphazene:thiophenol method) of $GuaLp_1$:$GlyLp_3$ (30:70 wt%) 2D-photoset ^1H NMR Spectrum – 400 MHz, 298 K, $CDCl_3$.

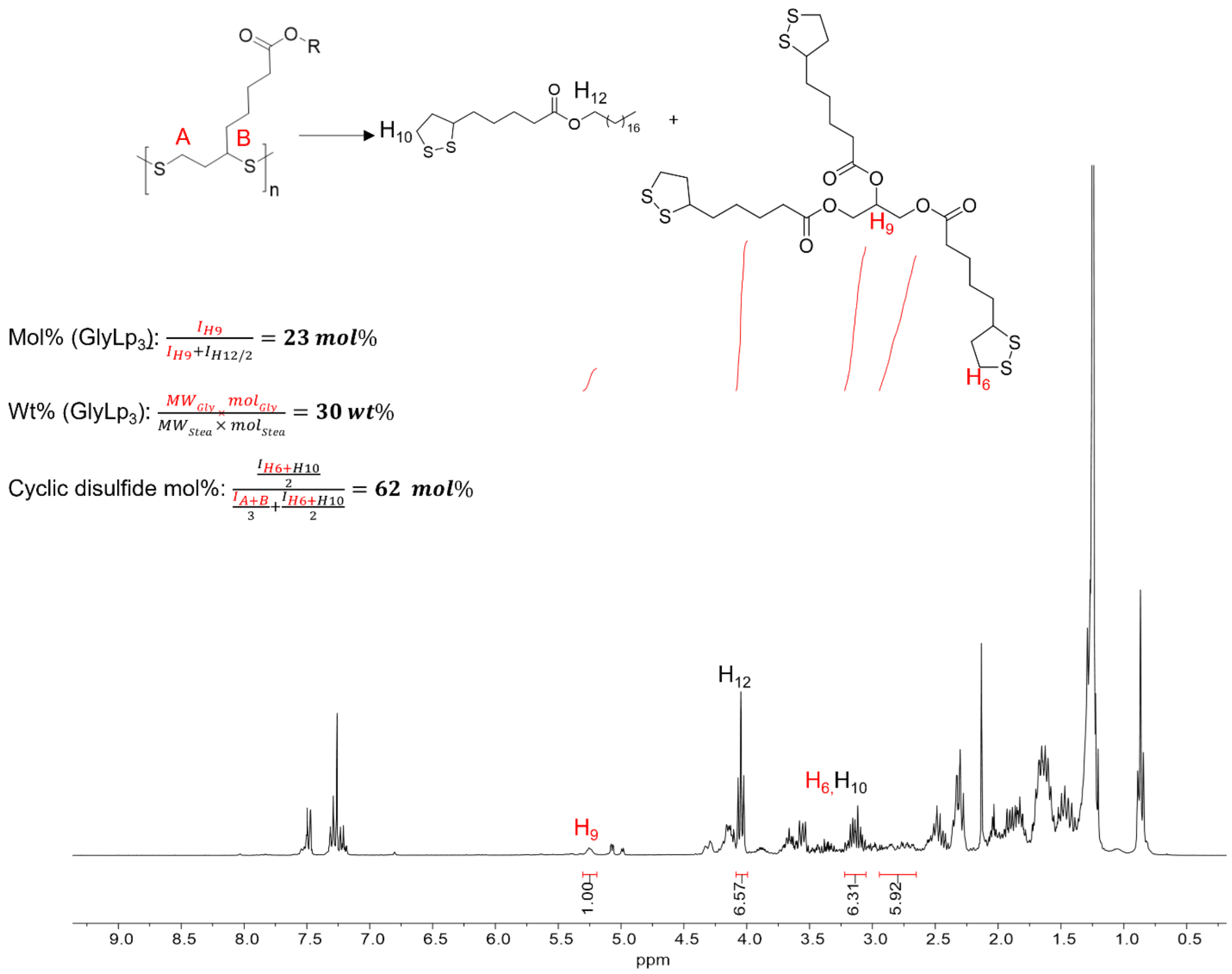

Fig. S26.

Catalyzed depolymerization (phosphazene:thiophenol method) of SteaLp$_1$:GlyLp$_3$ (30:70 wt%) 2D-photoset ^{1}H NMR Spectrum – 400 MHz, 298 K, $CDCl_3$.

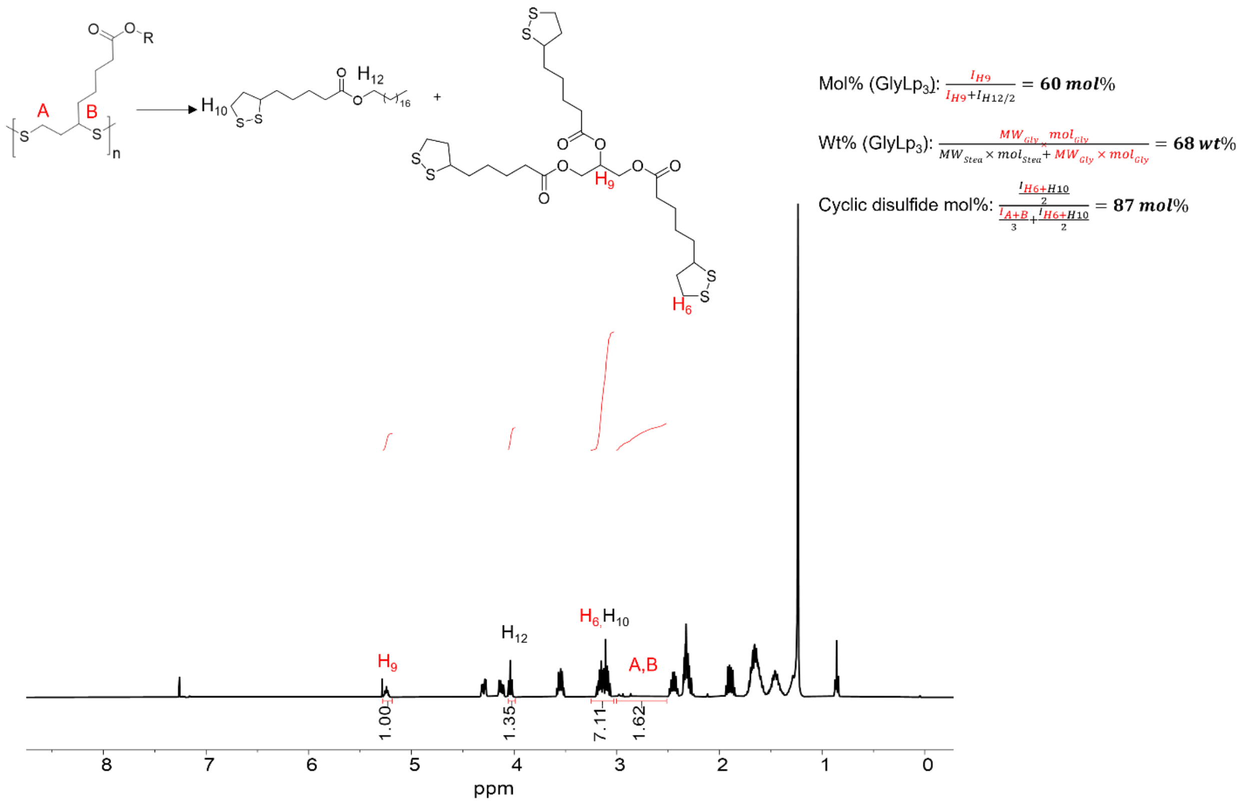

Fig. S27.
Thermal depolymerization (DMF method) of SteaLp1:GlyLp3 (32:68 wt%) 2D-photoset ^{1}H NMR Spectrum – 400 MHz, 298 K, $CDCl_3$.

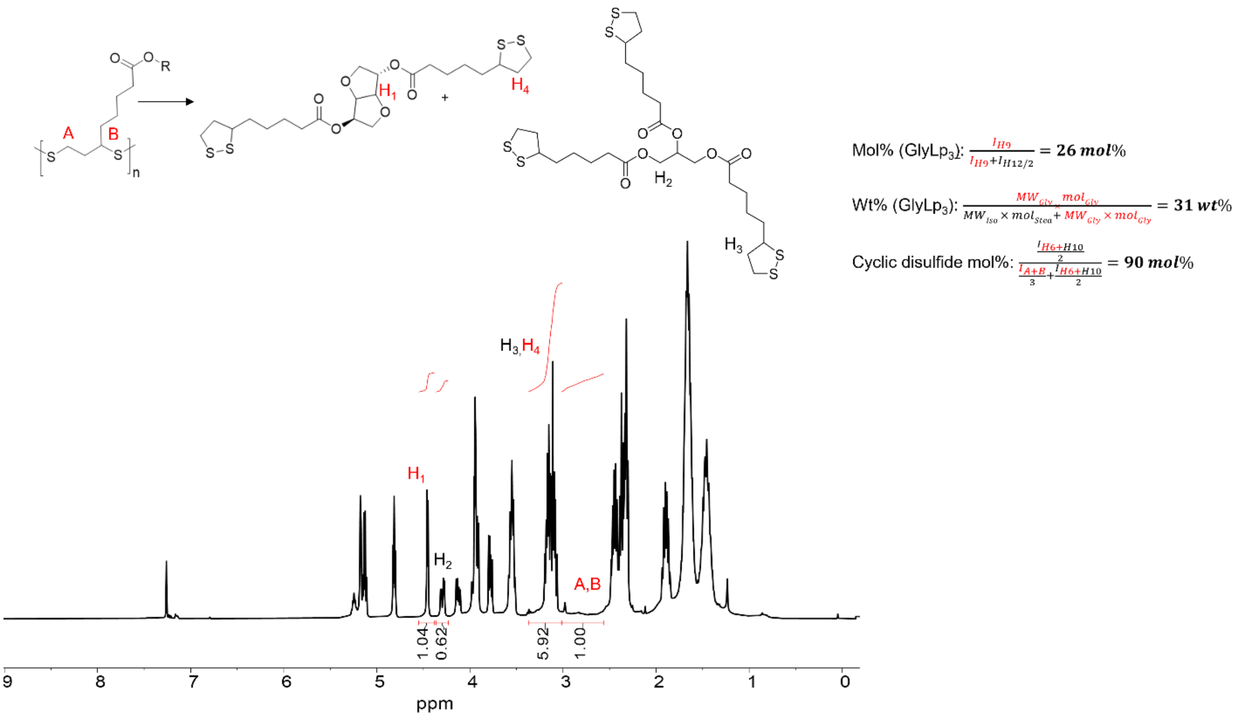

Fig. S28.
Thermal depolymerization (DMF method) of IsoLp2:GlyLp3 (70:30 wt%) 2D-photoset ^{1}H NMR Spectrum – 400 MHz, 298 K, $CDCl_3$.

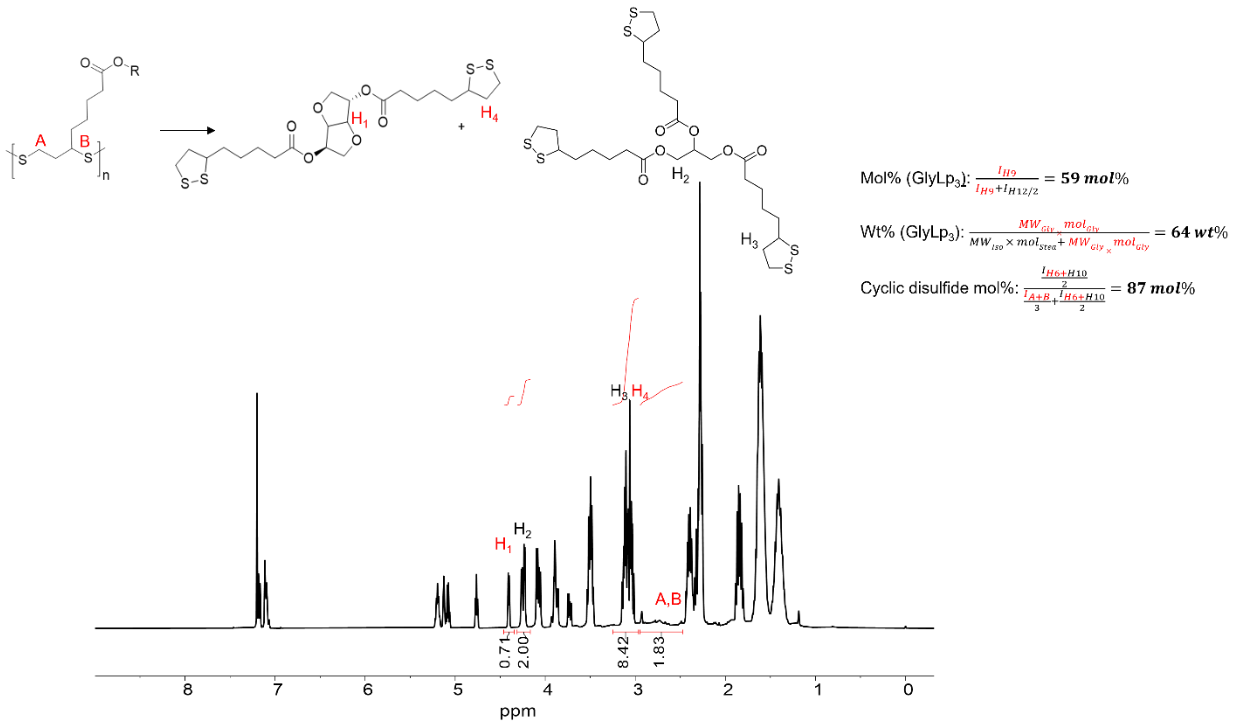

Fig. S29.

Thermal depolymerization (DMF method) of IsoLp2:GlyLp3 (28:72 wt%) 2D-photoset ^{1}H NMR Spectrum – 400 MHz, 298 K, $CDCl_3$.

Recycled resins from 3D-prints

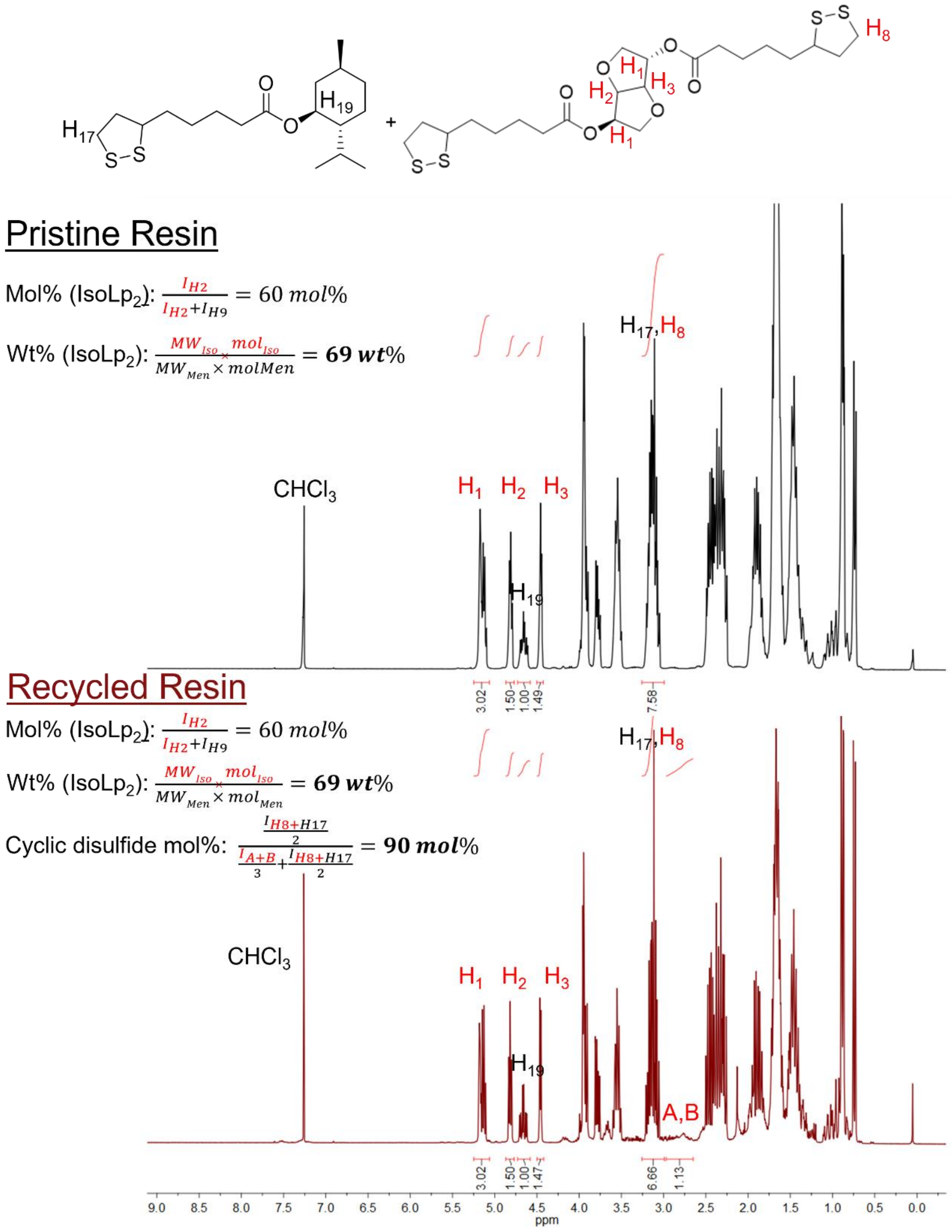

Fig. S30.

Catalyzed depolymerization (phosphazene:thiophenol method) of $MenLp_1$:$IsoLp_2$ (31:69 wt%) 3D-printed parts ^1H NMR Spectrum – 400 MHz, 298 K, $CDCl_3$.

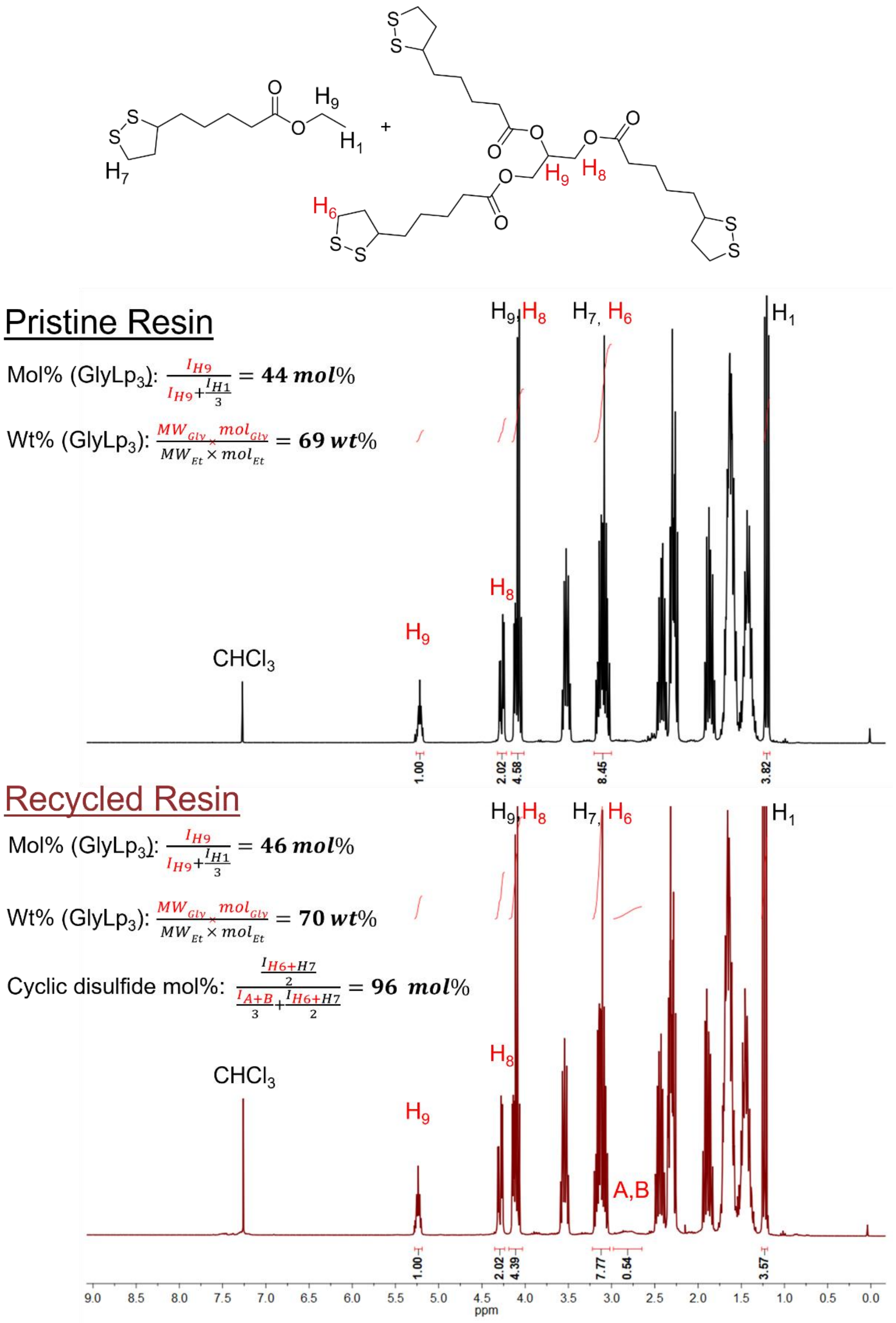

Fig. S31.

Catalyzed depolymerization (phosphazene:thiophenol method) of $EtLp_1$:$GlyLp_3$ (31:69 wt%) 3D-printed parts ^{1}H NMR Spectrum – 400 MHz, 298 K, $CDCl_3$.

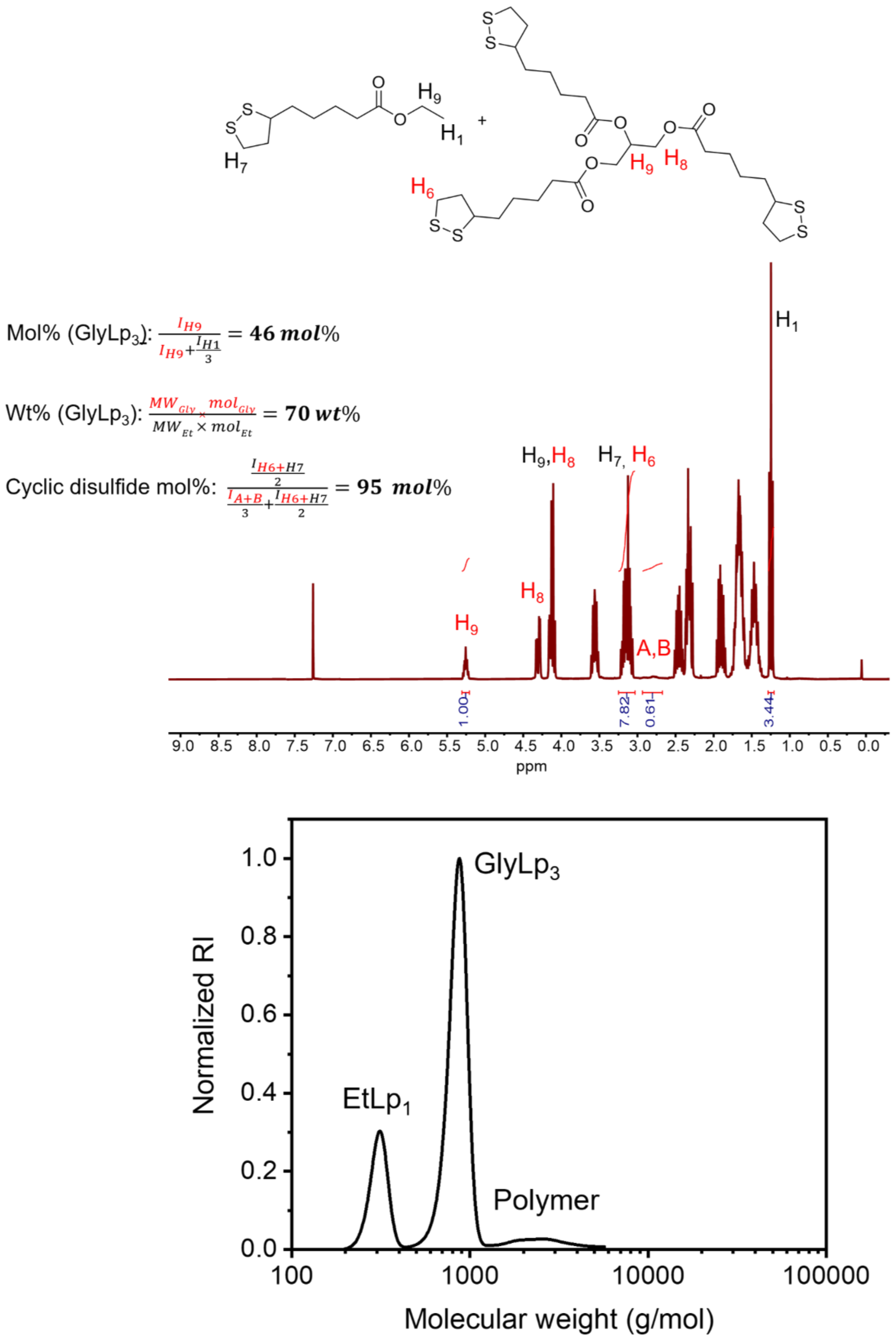

Fig. S32.

Catalyzed depolymerization (DBU:DTT method) of $EtLp_1$:$GlyLp_3$ (31:69 wt%) 3D-printed parts to yield recycled resin. ^1H NMR Spectrum – 400 MHz, 298 K, $CDCl_3$ (Top); SEC chromatogram ($CHCl_3$, 0.5 % w/w NEt_3) determined against polystyrene (PS) standards (Bottom).

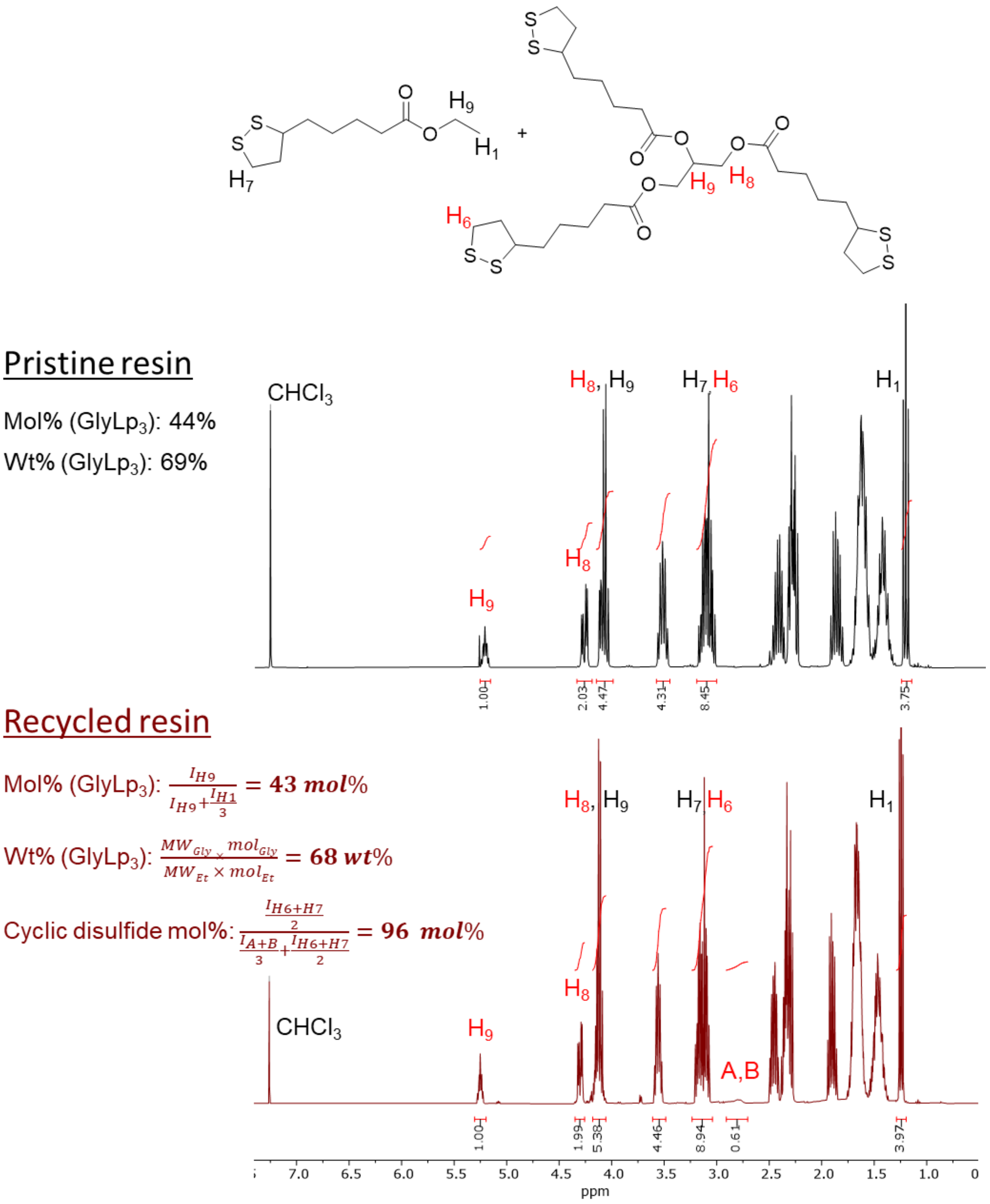

Fig. S33.
Thermal depolymerization (DMF, 140 °C) method of $EtLp_1$:$GlyLp_3$ (31:69 wt%) 3D-printed parts *(***1st recycle** compared to pristine*)* ^{1}H NMR Spectrum – 400 MHz, 298 K, $CDCl_3$.

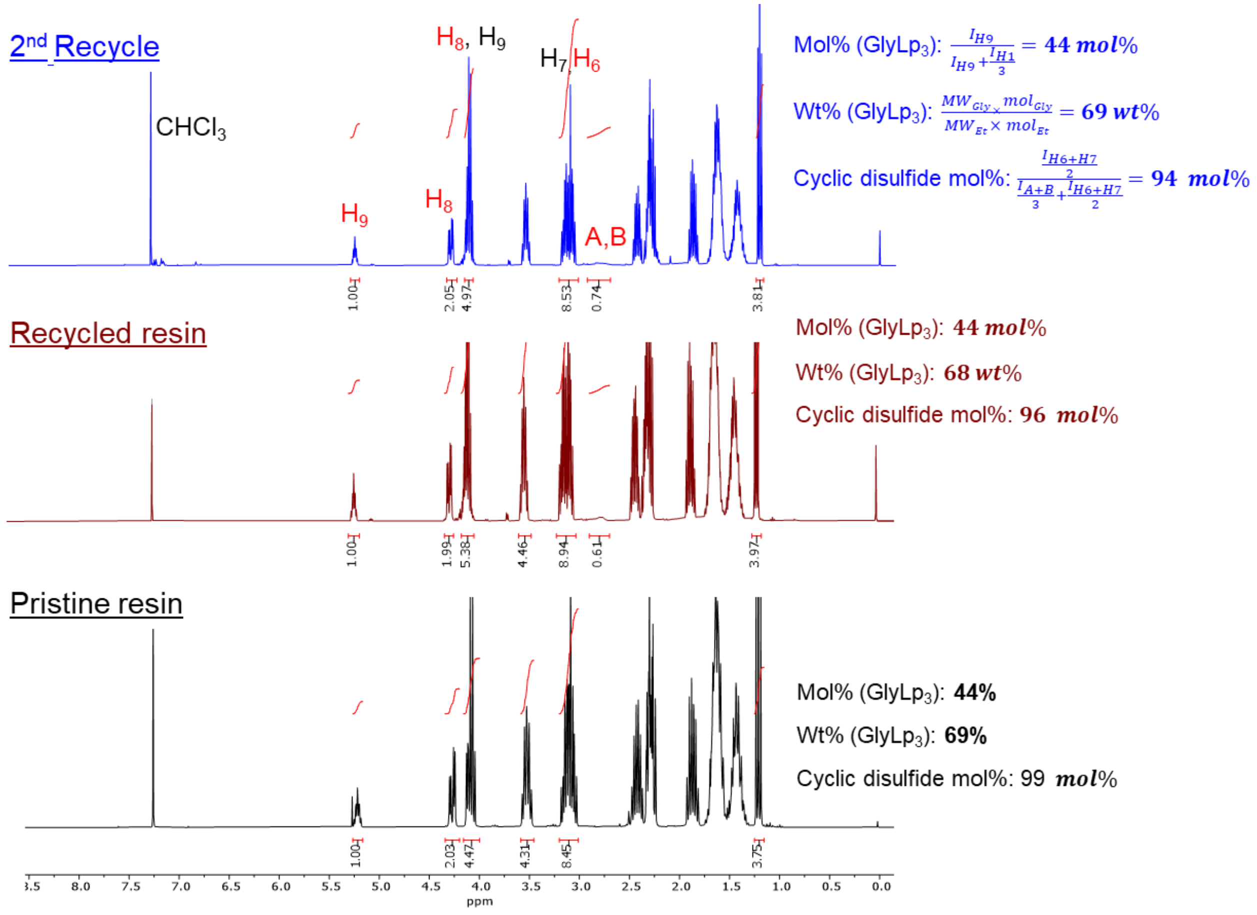

Fig. S34.
Thermal depolymerization (DMF, 140 °C) method of $EtLp_1$:$GlyLp_3$ (31:69 wt%) 3D-printed parts **(2nd recycle** compared to 1st recycle and pristine) ^{1}H NMR Spectrum – 400 MHz, 298 K, $CDCl_3$.

Table. S2.

Summary of catalyzed depolymerization (phosphazene:thiophenol) experiments for 2D-photosets and 3D-printed parts.

Resin (30:70 wt.%)	Recycled wt.% ratio	Recycled disulfide content (mol%)	Yield of recycled resin (%)
	**2D-photosets**		
$MenLp_1$:$IsoLp_2$	34:66	96	96
$EtLp_1$:$IsoLp_2$	52:48	65	50
$GuaLp_1$:$IsoLp_2$	61:39	56	29
$SteaLp_1$:$IsoLp_2$	61:39	57	24
$MenLp_1$:$GlyLp_3$	44:56	68	59
$EtLp_1$:$GlyLp_3$	30:70	96	85
$GuaLp_1$:$GlyLp_3$	68:32	53	42
$SteaLp_1$:$GlyLp_3$	70:30	62	38
	**3D-prints**		
**Resin (31:69 wt.%)**			
$MenLp_1$:$IsoLp_2$	31:69	90	98
$EtLp_1$:$GlyLp_3$	30:70	96	97

Reagents & conditions: Thiophenol (0.1 equiv.), P_1-t-Bu (0.1 equiv.), 0.25 M MeTHF (1 equiv.), 80 °C, 3 h.

Table. S3.

Summary of un-catalyzed thermal depolymerization (DMF, 140 ° C) experiments for 2D-photosets and 3D-printed parts.

Resin (wt.% ratio)	Recycled wt.% ratio	Recycled disulfide content (mol%)	Yield of recycled resin (%)
	**2D-photosets**		
$SteaLp_1$:$IsoLp_2$ (30:70)	33:67	86	[a]99
$MenLp_1$:$GlyLp_3$ (33:67)	44:56	80	80
$EtLp_1$:$GlyLp_3$ (34:66)	35:65	87	88
$SteaLp_1$:$GlyLp_3$ (32:68)	32:68	87	83
$IsoLp_2$:$GlyLp_3$ (70:30)	69:31	90	76
$IsoLp_2$:$GlyLp_3$ (28:72)	36:64	87	83
	**3D-prints**		
**Resin (31:69 wt.%) $EtLp_1$:$GlyLp_3$**			
1st recycle	32:68	96	91
2nd recycle	31:69	94	95

Reagents & conditions: DMF (10 equiv.), 140 °C, 2 h. [a]DCM still present in NMR spectrum.

Hydrolysed resins from 3D-printed parts

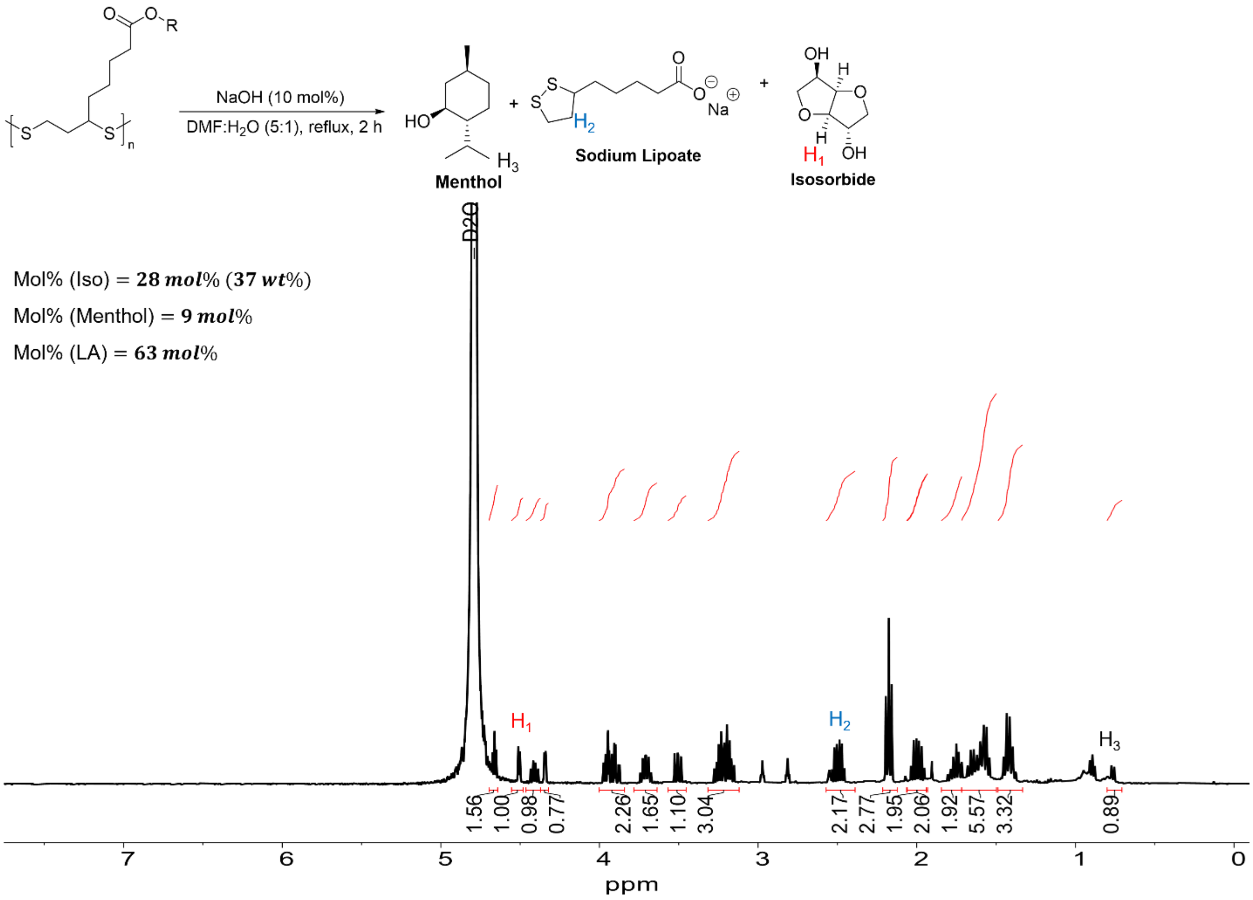

Fig. S35.

Depolymerization and hydrolysis of MenLp1:IsoLp2 (30:70 wt%, entry 13, Table S4) 3D printed parts ^{1}H NMR Spectrum – 400 MHz, 298 K, D_2O.

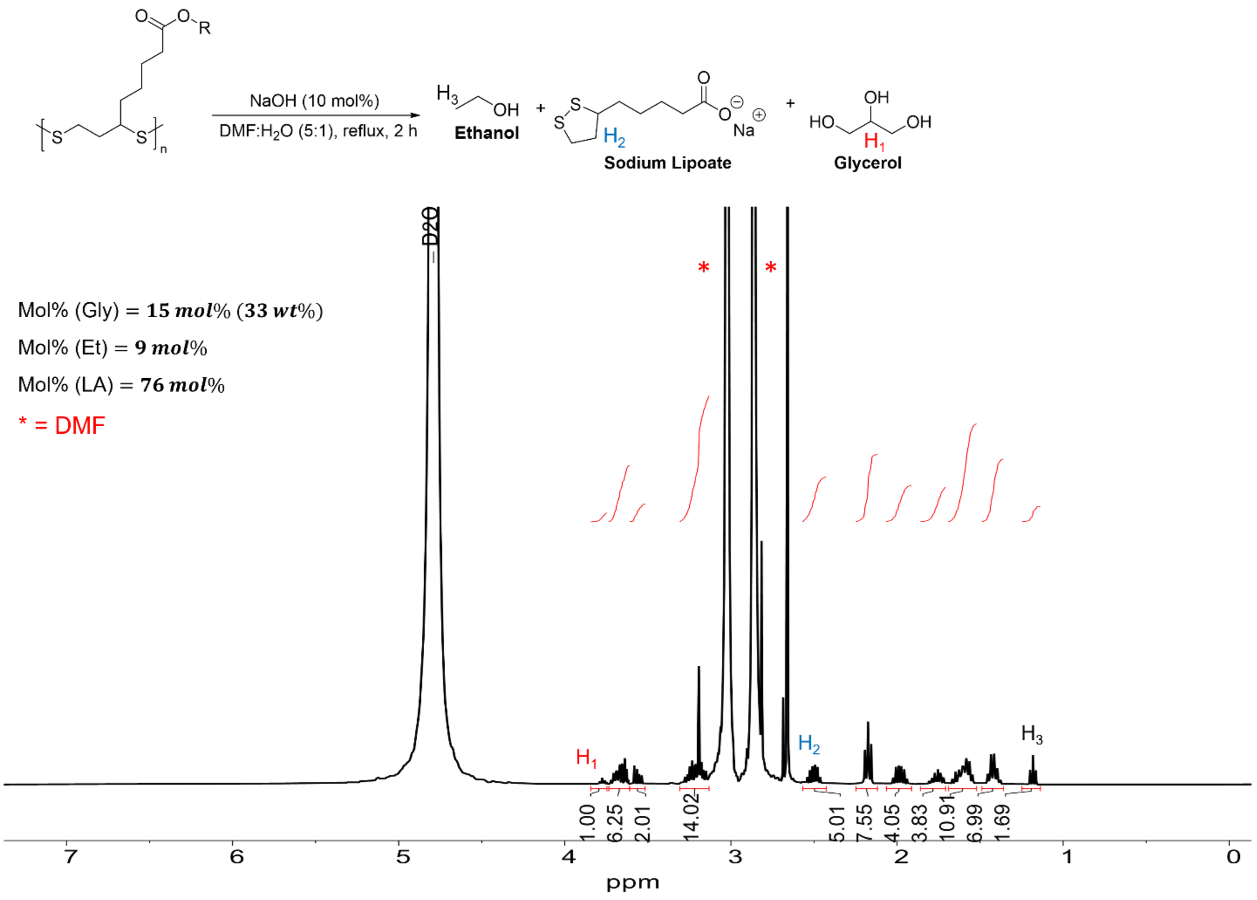

Fig. S36.

Depolymerization and hydrolysis of $EtLp_1$:$GlyLp_3$ (31:69 wt%, entry 3, Table S4) 3D printed parts ^{1}H NMR Spectrum – 400 MHz, 298 K, D_2O before removing solvents under vacuum.

Hydrolysed resins from 2D photoset

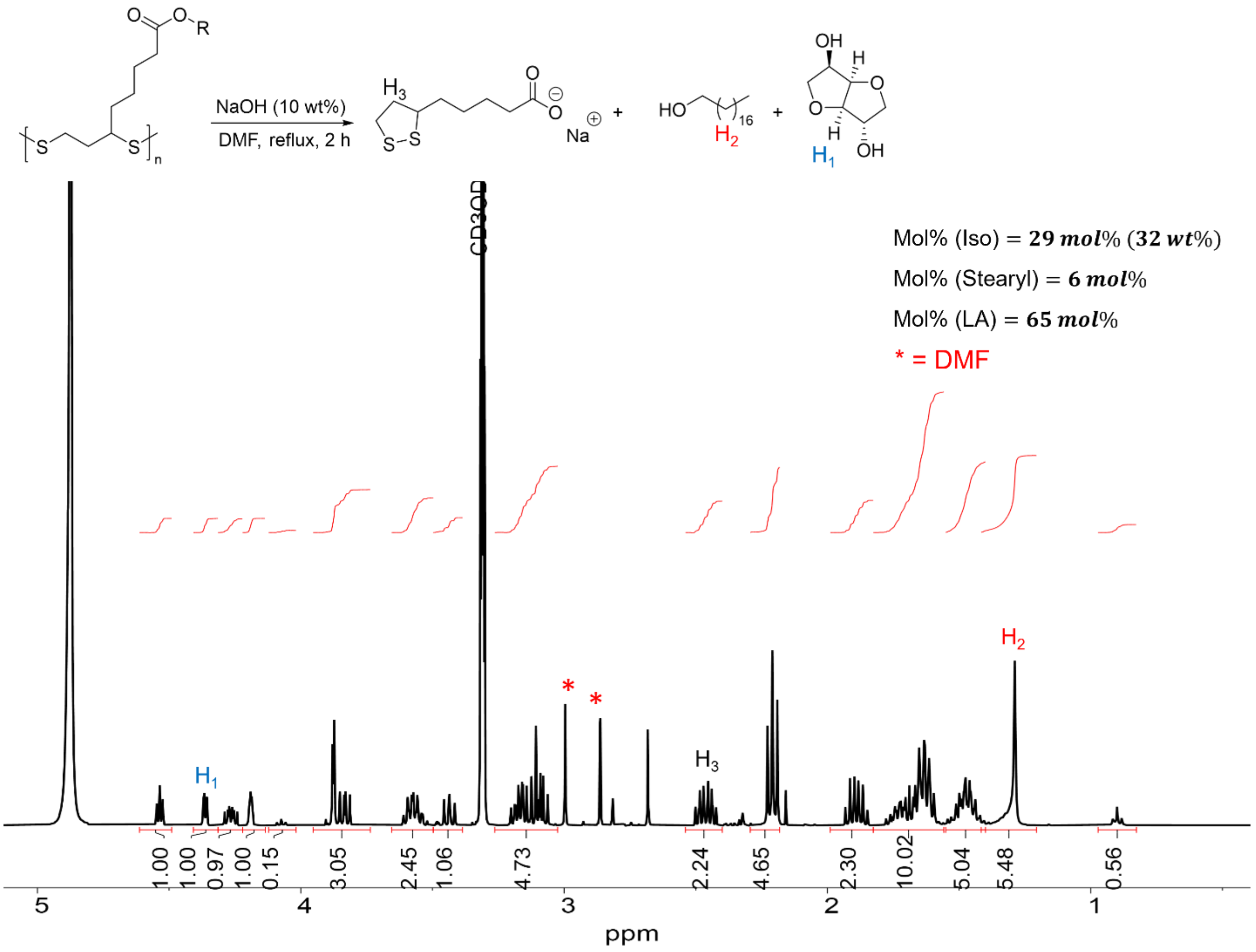

Fig. S37.
Depolymerization of SteaLp1:IsoLp2 (30:70 wt%, entry 14, Table S4) 2D photoset ^{1}H NMR Spectrum – 400 MHz, 298 K, CD_3OD.

Table. S4.

Summary of one-pot depolymerization and hydrolysis experiments for 2D photosets and 3D-printed parts.

Entry	Resin (31:69 wt.%)	Solvent system	Catalyst	NMR Yield of hydrolysed Compounds (%)[a]
		**3D printed**		
1	$EtLp_1$:$GlyLp_3$	DMF:H_2O (5:1)	NaOH (10 mol%)	< 5%
2	$EtLp_1$:$GlyLp_3$	DMF:H_2O (5:1)	TBD (10 mol%)	< 5%
3	$EtLp_1$:$GlyLp_3$	DMF:H_2O (5:1)	NaOH (10 wt%)	>99%
4	$EtLp_1$:$GlyLp_3$	DMF:H_2O (5:1)	KOH (10 wt%)	>99%
5	[b]$EtLp_1$:$GlyLp_3$	d_7-DMF:H_2O (5:1)	NaOH (10 wt%)	n.d.
6	[b,c]$EtLp_1$:$GlyLp_3$	d_7-DMF:H_2O (5:1)	NaOH (10 wt%)	/
7	$EtLp_1$:$GlyLp_3$	H_2O	Novozym® 435 (10 wt%)	/
8	$EtLp_1$:$GlyLp_3$	THF:H_2O (5:1)	Novozym® 435 (10 wt%)	/
9	$EtLp_1$:$GlyLp_3$	THF:H_2O (5:1)	/	/
10	$EtLp_1$:$GlyLp_3$	Me-THF/H_2O (5:1)	Novozym® 435 (10 wt%)	/
11	$EtLp_1$:$GlyLp_3$	Me-THF/H_2O (5:1)	NaOH (10 wt%)	>99%
12	[d]$MenLp_1$:$IsoLp_2$	DMF:H_2O (5:1)	NaOH (10 wt%)	>99%
13	[d]$MenLp_1$:$IsoLp_2$	d_7-DMF:H_2O (5:1)	NaOH (10 wt%)	>99%
		**2D photosets**		
14	[d]$SteaLp_1$:$IsoLp_2$	DMF:H_2O (5:1)	NaOH (10 wt%)	>99%
15	[d]$SteaLp_1$:$IsoLp_2$	d_7-DMF:H_2O (5:1)	NaOH (10 wt%)	>99%

Reaction conditions: 150 mg grounded polymer, catalyst (stated amount); 5 mL DMF + 1 mL H_2O, reflux, 3 h. [a]Detected by ^{1}H NMR and calculated with respect to the presence of esters groups; [b]NMR scale experiment in d_7-DMF; [c] after 3 h the NMR tube was directly cooled down in cold toluene, but the monomers re-polymerized; [d]resin ratio = 30:70 wt%.

Mass Spectrometry

2D-photoset recycling

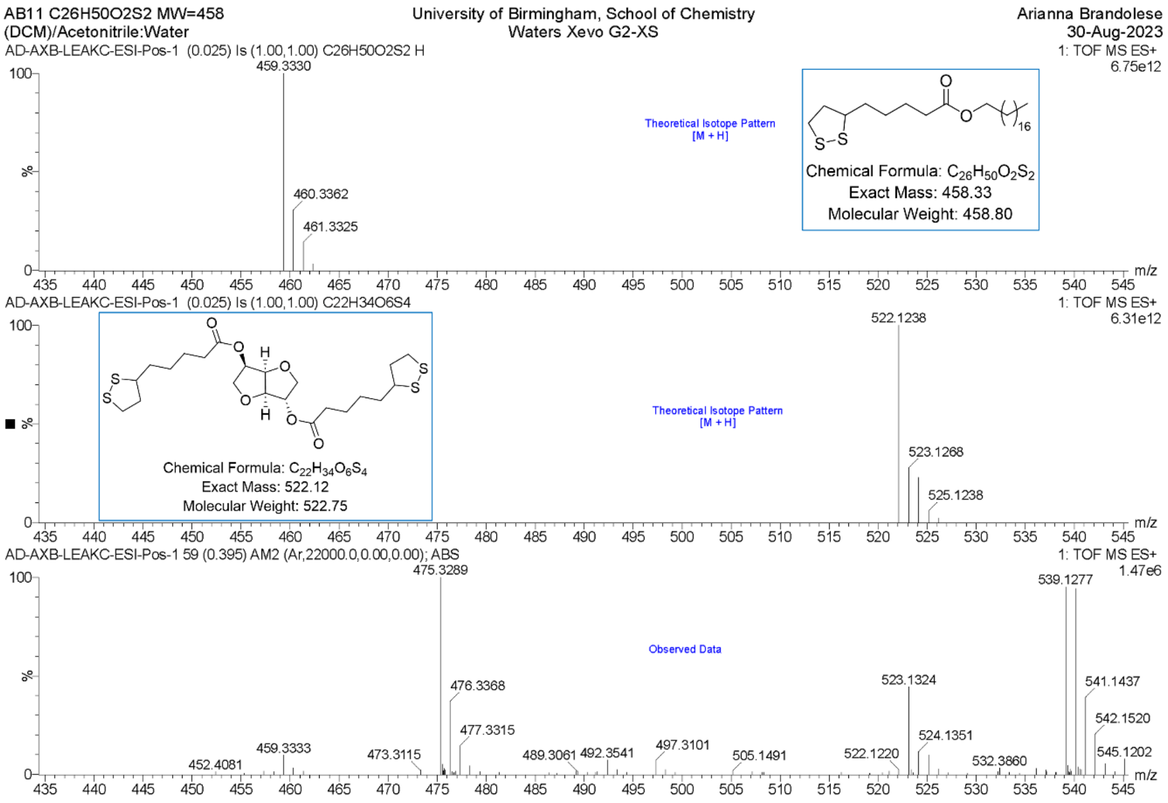

Fig. S38.

Mass spectrum (ES-H+) of thermal depolymerization (DMF, 140 °C) for SteaLp1:IsoLp2 (30:70 wt%). Theoretical mass and isotope pattern for each component are included in plot.

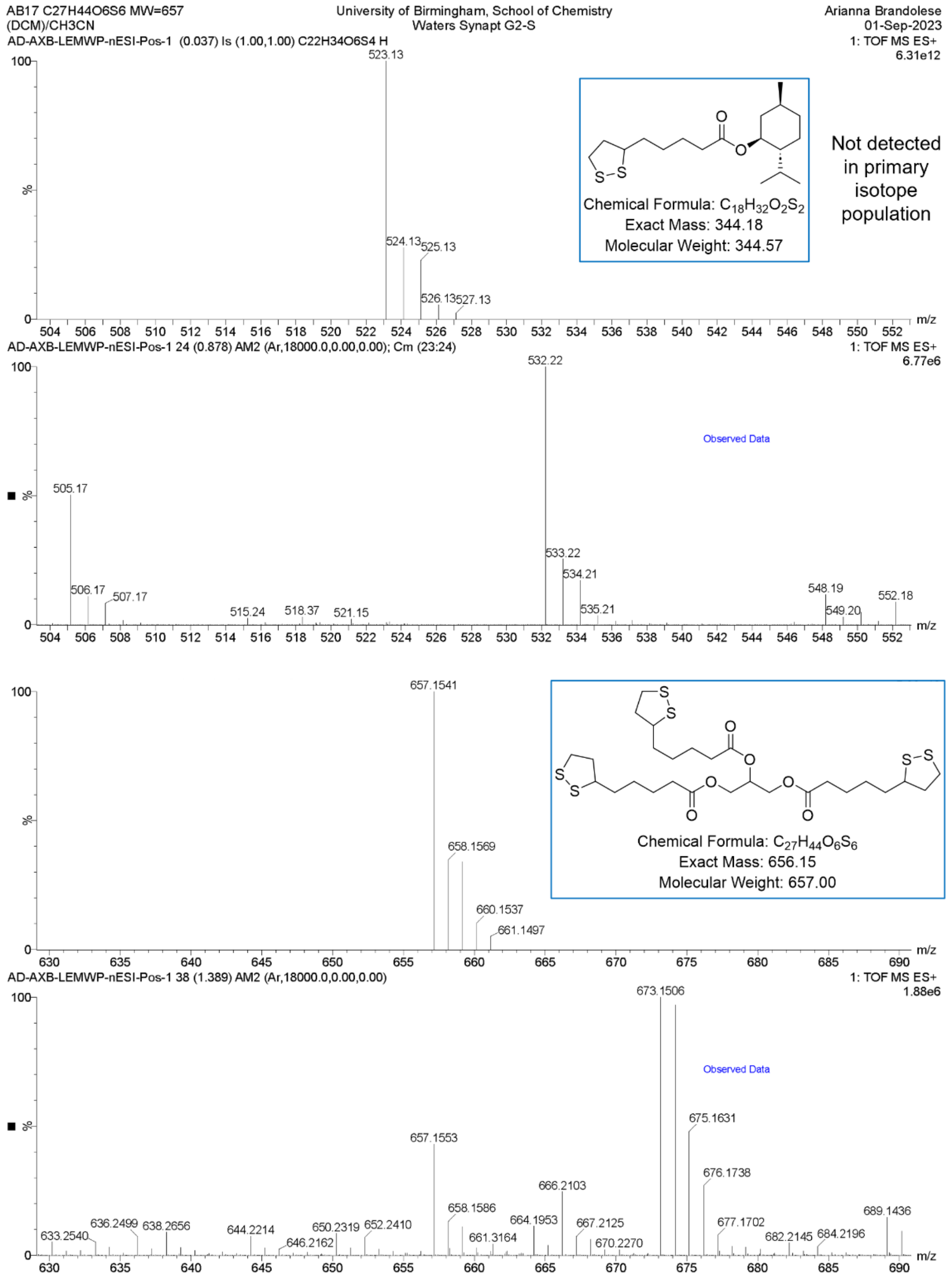

Fig. S39.

Mass spectrum (ES-H+) of thermal depolymerization (DMF, 140 °C) for MenLp1:GlyLp3 (33:67 wt%). Theoretical mass and isotope pattern for each component are included in plot.

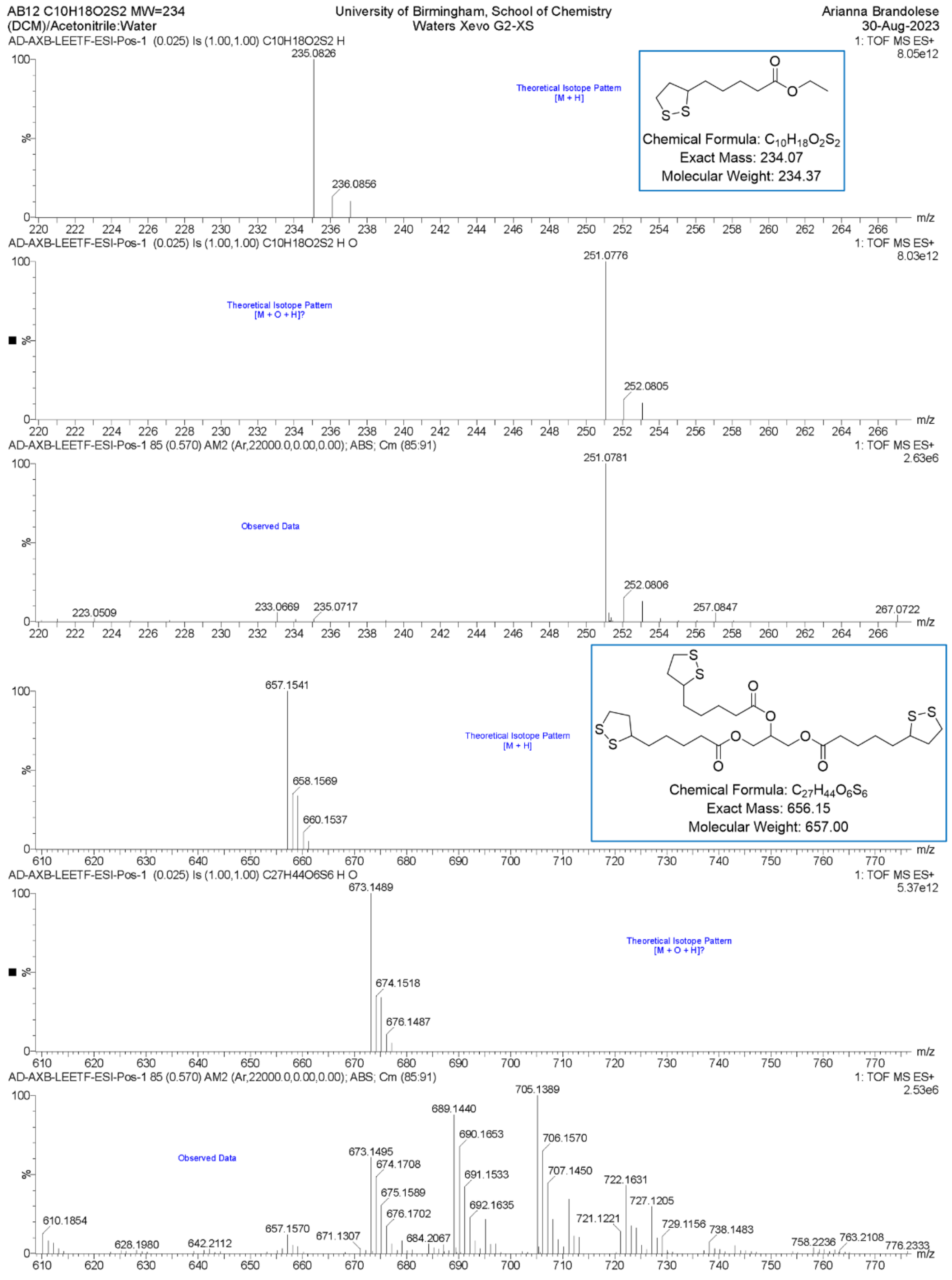

Fig. S40.

Mass spectrum (ES-H+) of thermal depolymerization (DMF, 140 °C) for EtLp1:GlyLp3 (34:66 wt%). Theoretical mass and isotope pattern for each component are included in plot.

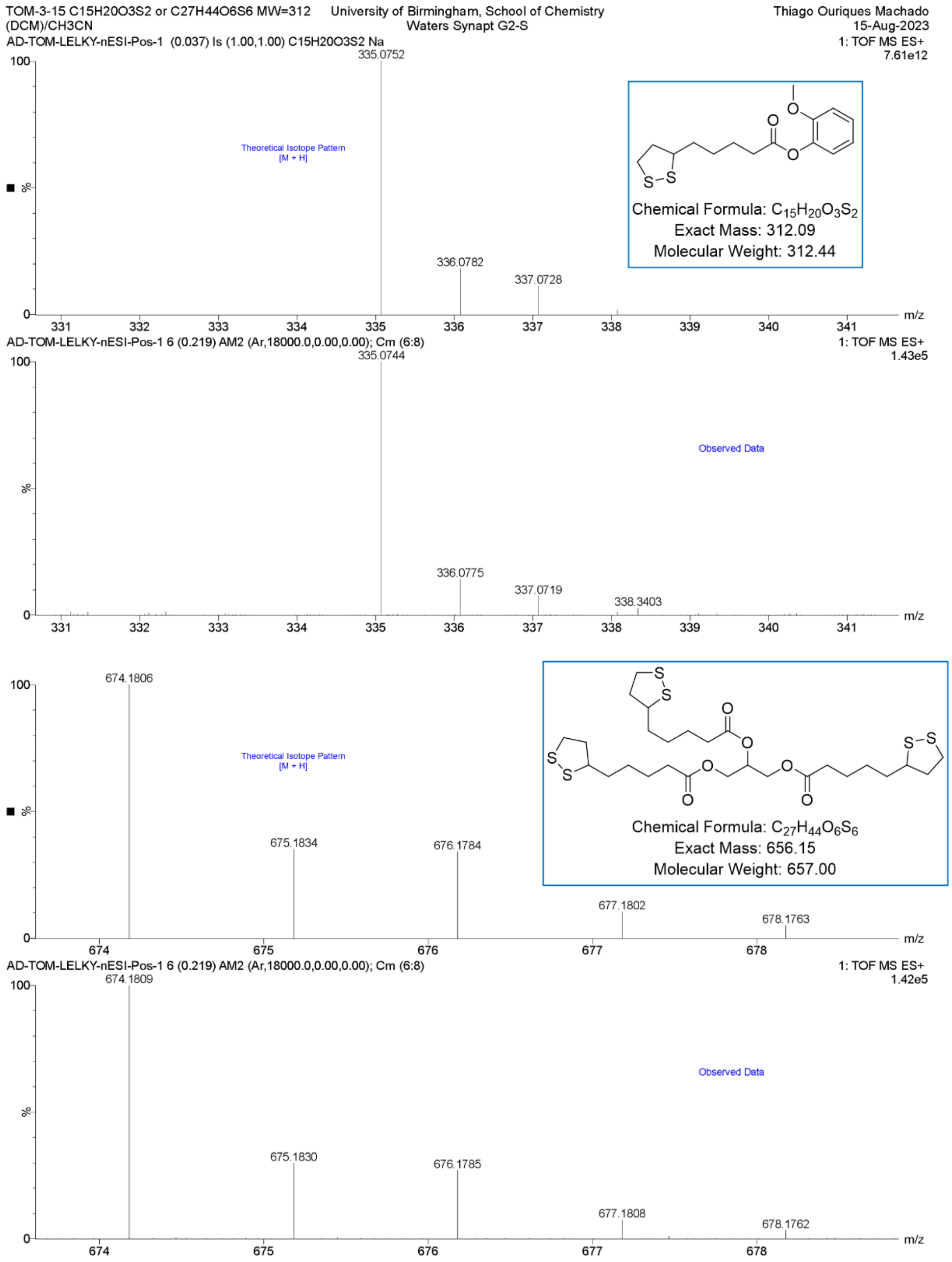

Fig. S41.

Mass spectrum (ES-H+) of thermal depolymerization (DMF, 140 °C) for GuaLp1:GlyLp3 (30:70 wt%). Theoretical mass and isotope pattern for each component are included in plot.

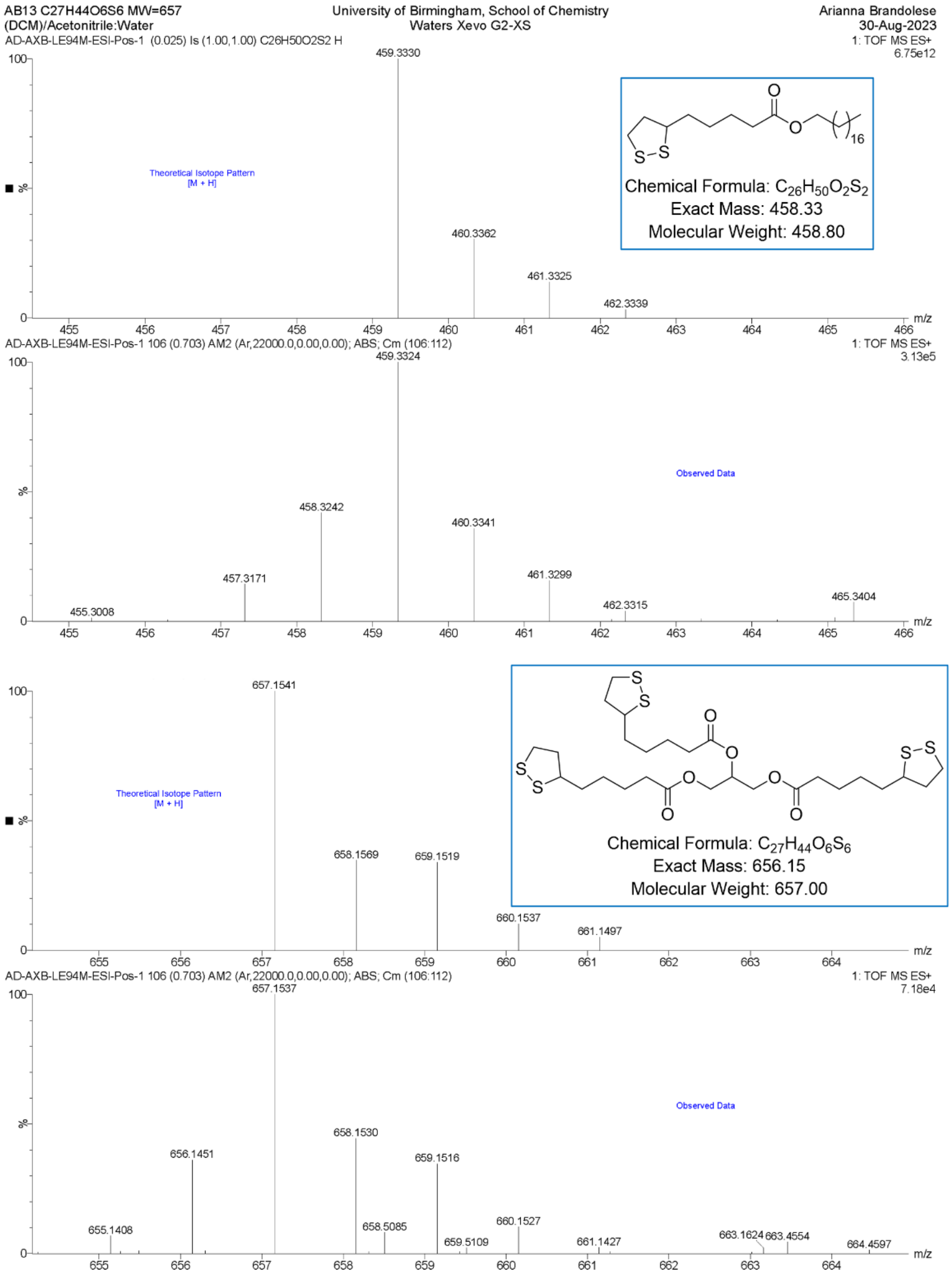

Fig. S42.

Mass spectrum (ES-H+) of thermal depolymerization (DMF, 140 °C) for SteaLp1:GlyLp3 (32:68 wt%). Theoretical mass and isotope pattern for each component are included in plot.

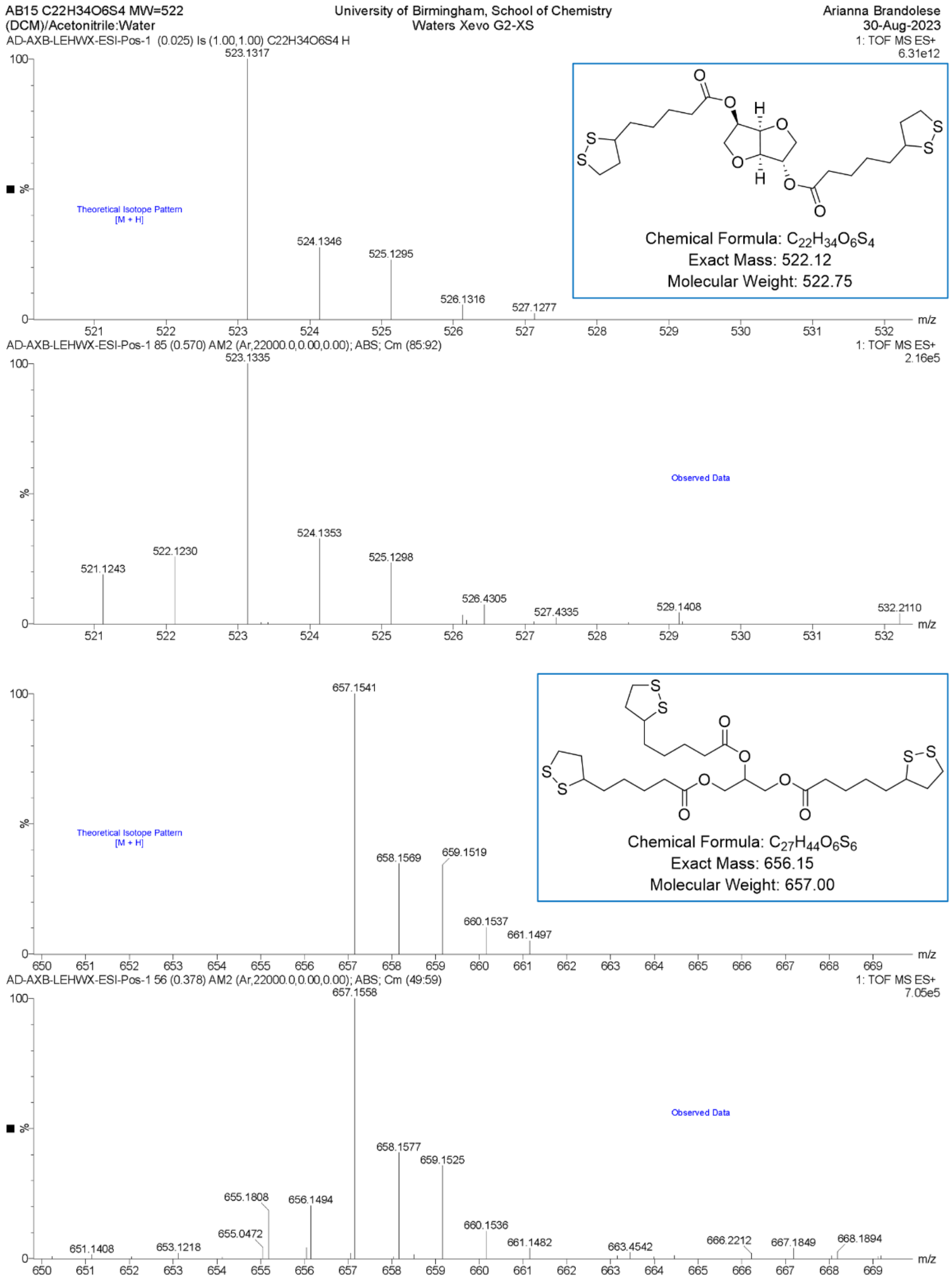

Fig. S43.

Mass spectrum (ES-H+) of thermal depolymerization (DMF, 140 °C) for IsoLp2:GlyLp3 (70:30 wt%). Theoretical mass and isotope pattern for each component are included in plot.

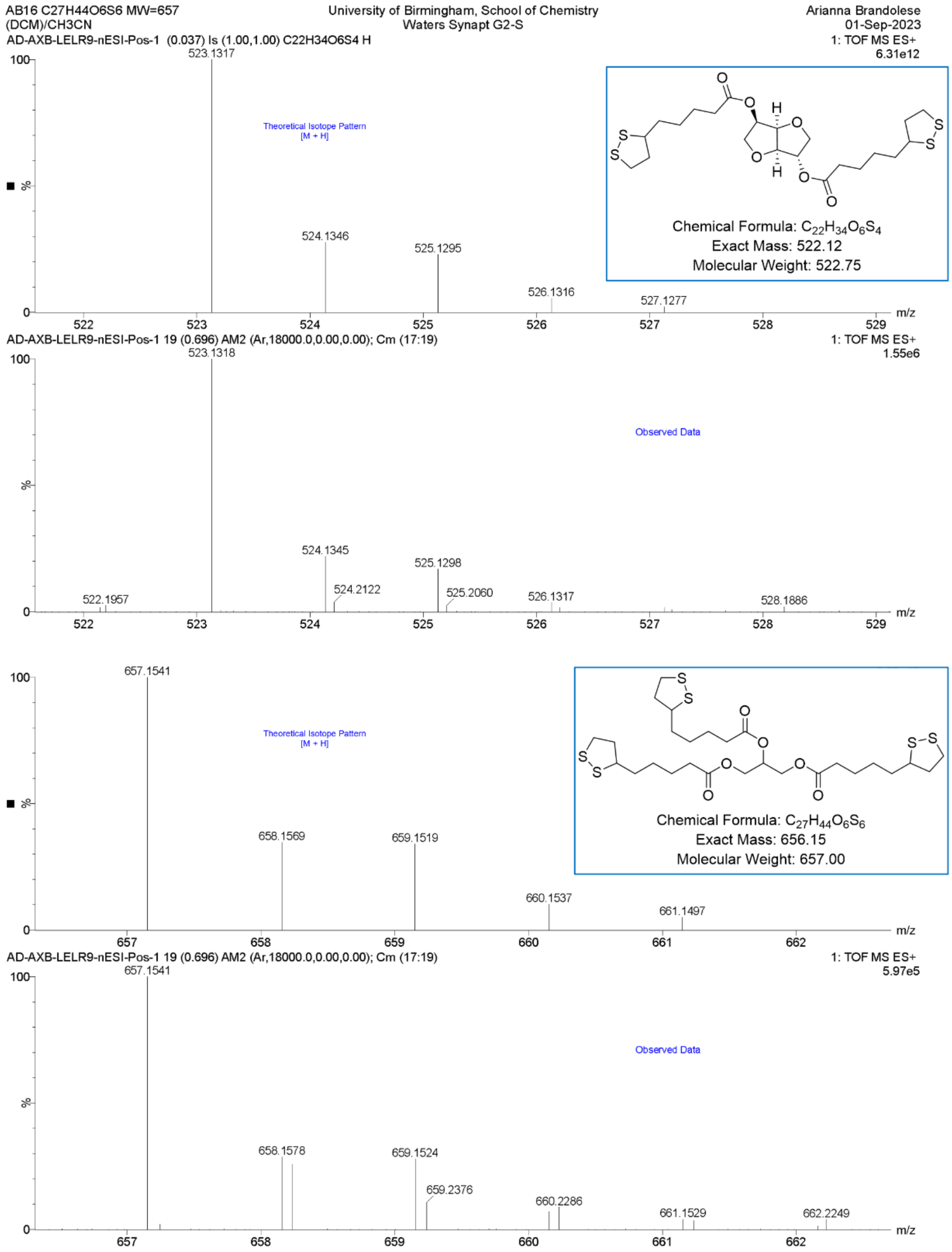

Fig. S44.

Mass spectrum (ES-H+) of thermal depolymerization (DMF, 140 °C) for IsoLp2:GlyLp3 (28:72 wt%). Theoretical mass and isotope pattern for each component are included in plot.

3D-printed parts recycling

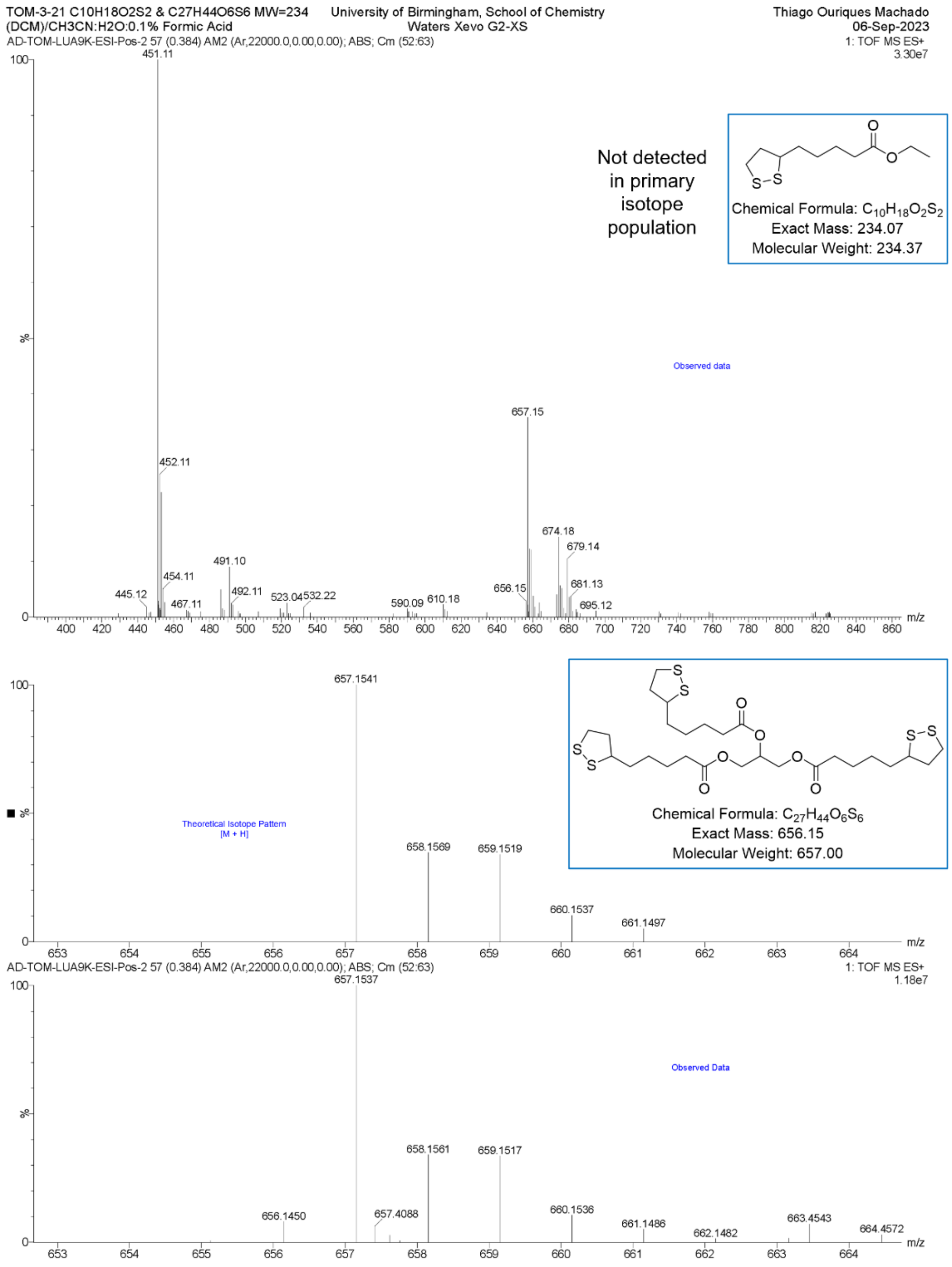

Fig. S45.

Mass spectrum (ES-H+) of thermal depolymerization (DMF, 140 °C) for **1st recycle** of EtLp1:GlyLp3 (31:69 wt%). Theoretical mass and isotope pattern for each component are included in plot.

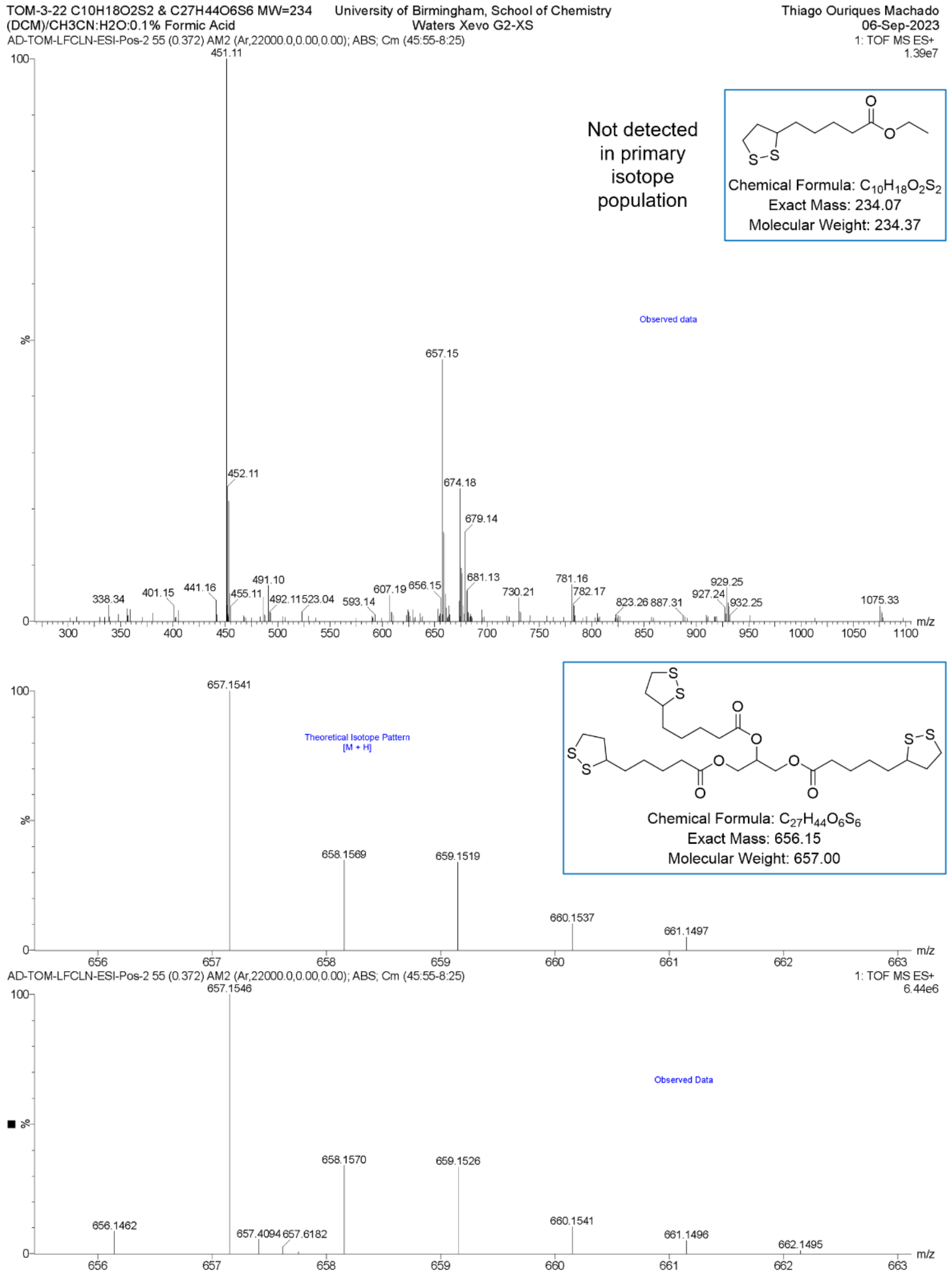

Fig. S46.

Mass spectrum (ES-H+) of thermal depolymerization (DMF, 140 °C) for **2nd recycle** of EtLp$_1$:GlyLp$_3$ (31:69 wt%). Theoretical mass and isotope pattern for each component are included in plot.

FT-IR spectra

Monomers

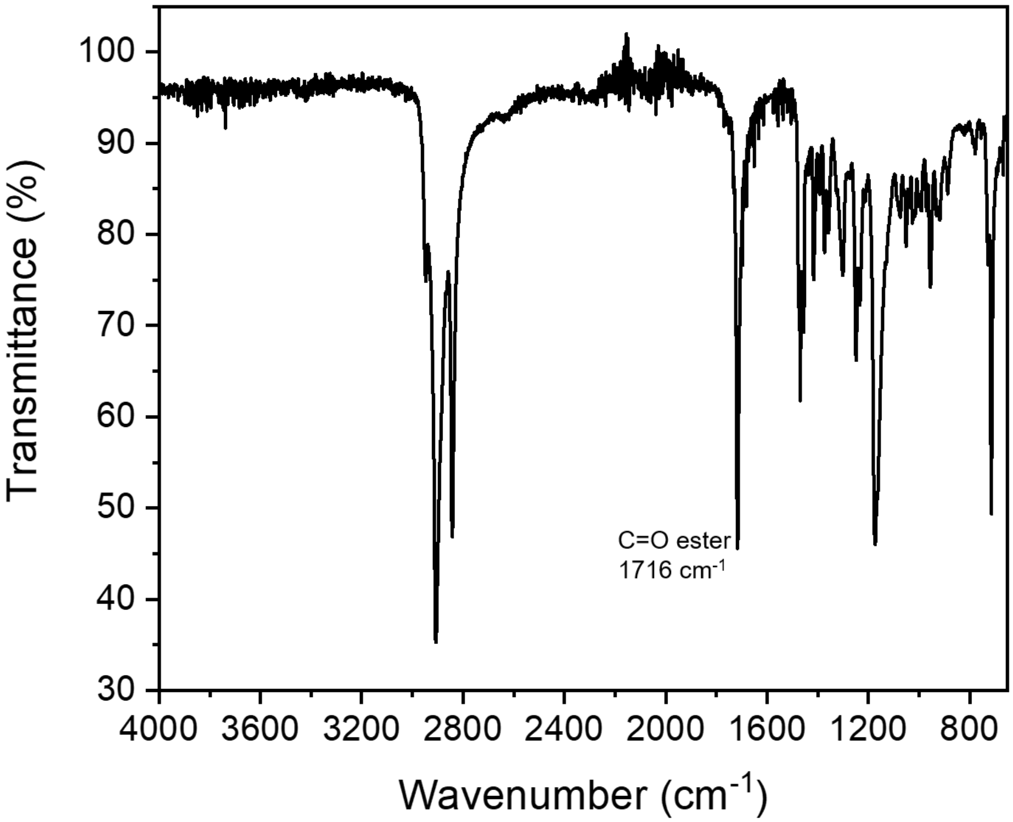

Fig. S47.

FT-IR spectra of $MenLp_1$.

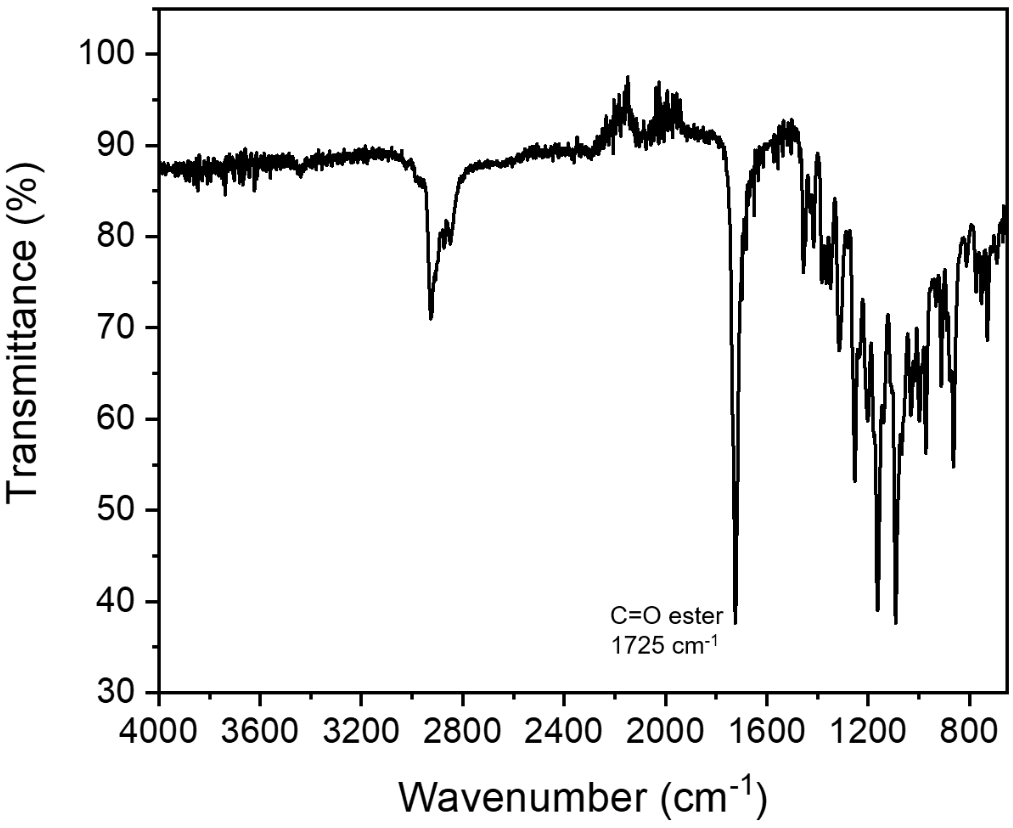

Fig. S48.
FT-IR spectra of $IsoLp_2$.

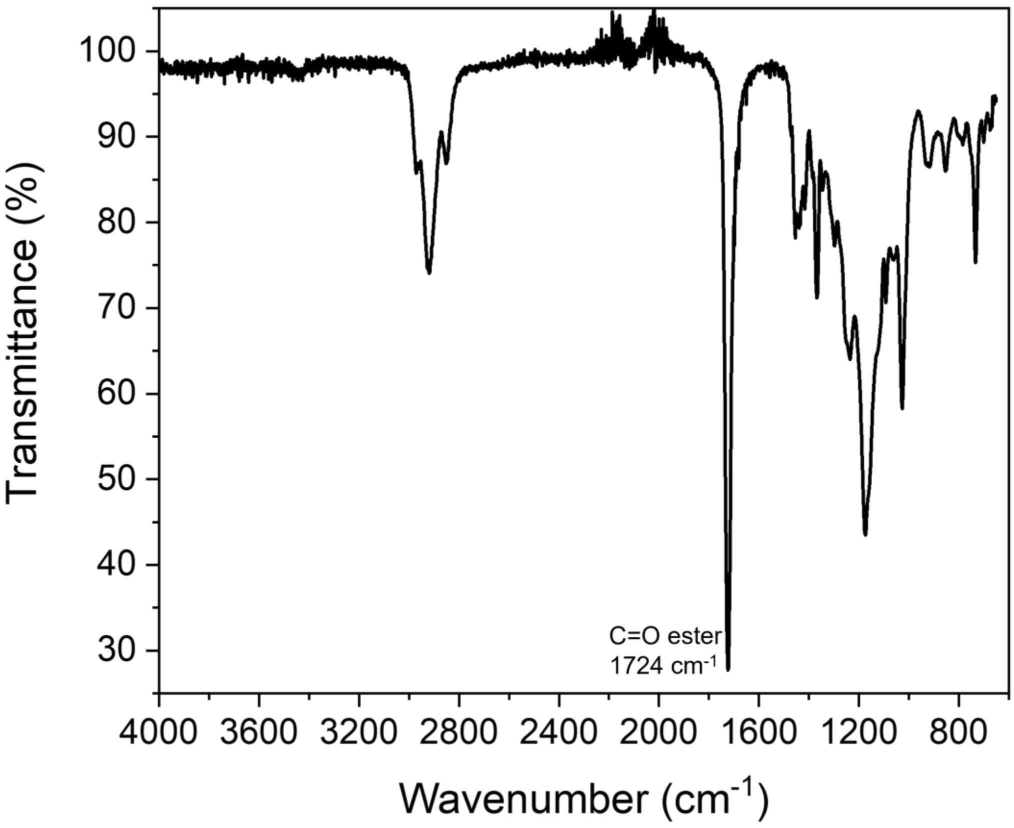

Fig. S49.

FT-IR spectra of $EtLp_1$.

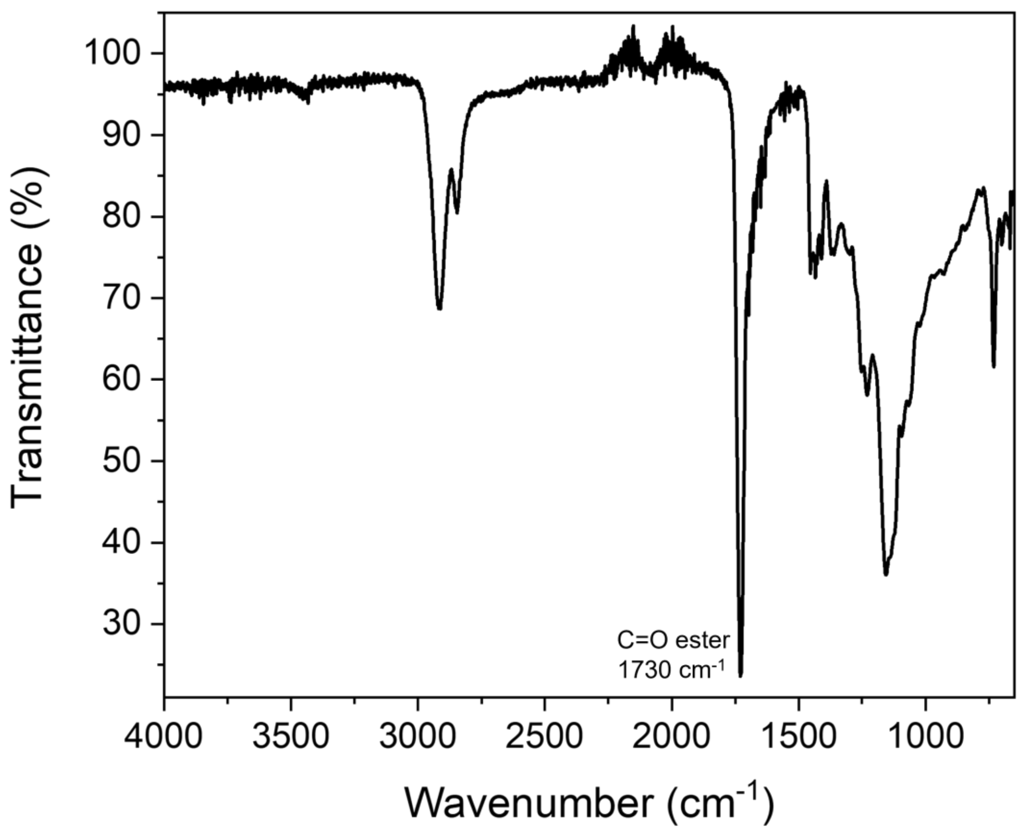

Fig. 50.
FT-IR spectra of $GlyLp_3$.

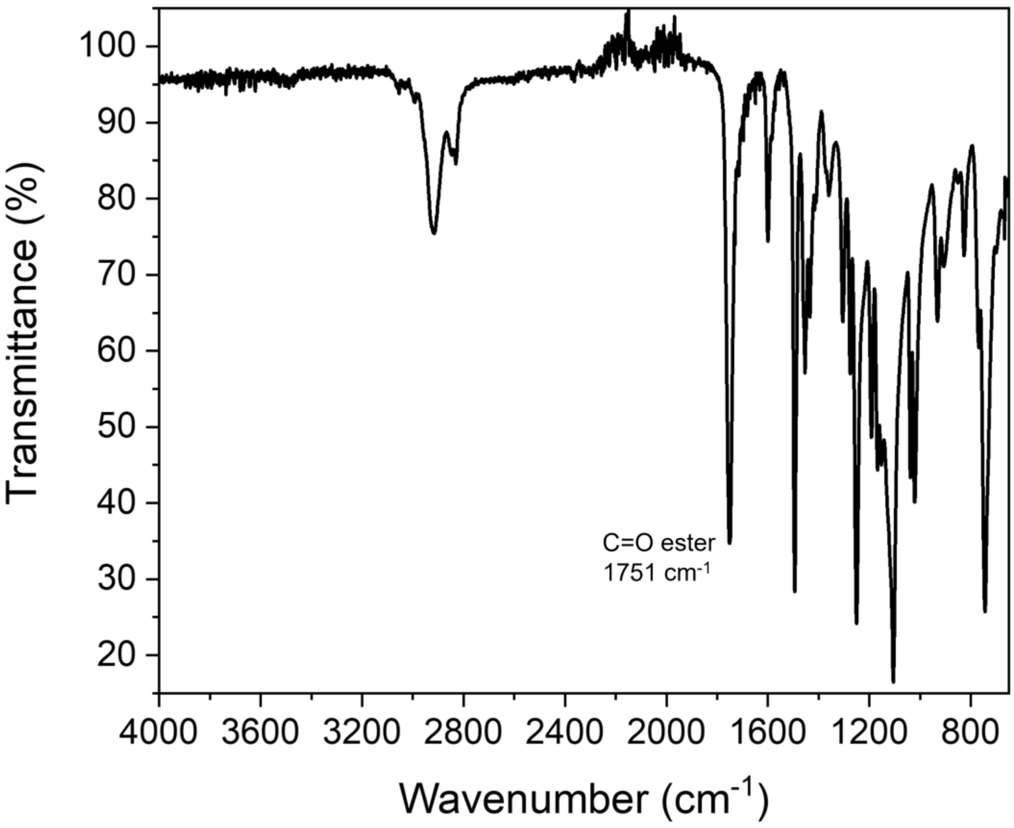

Fig. S51.

FT-IR spectra of $GuaLp_1$.

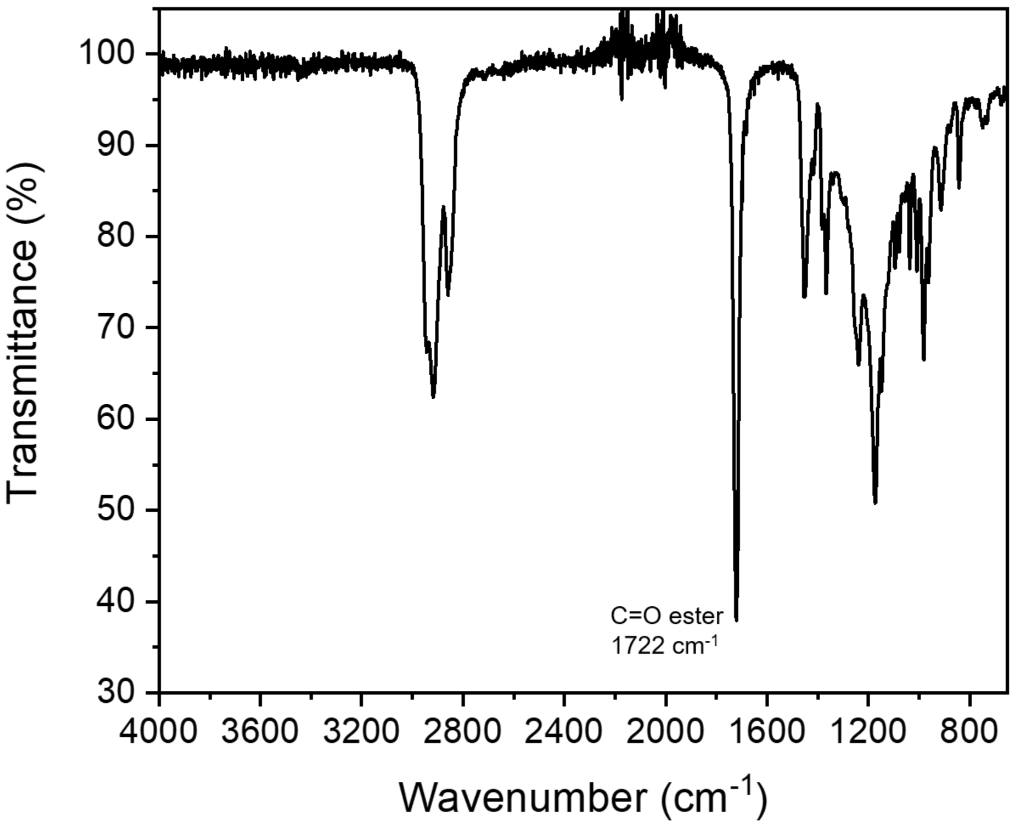

Fig. S52.
FT-IR spectra of $SteaLp_1$.

2D-photosets – post-curing

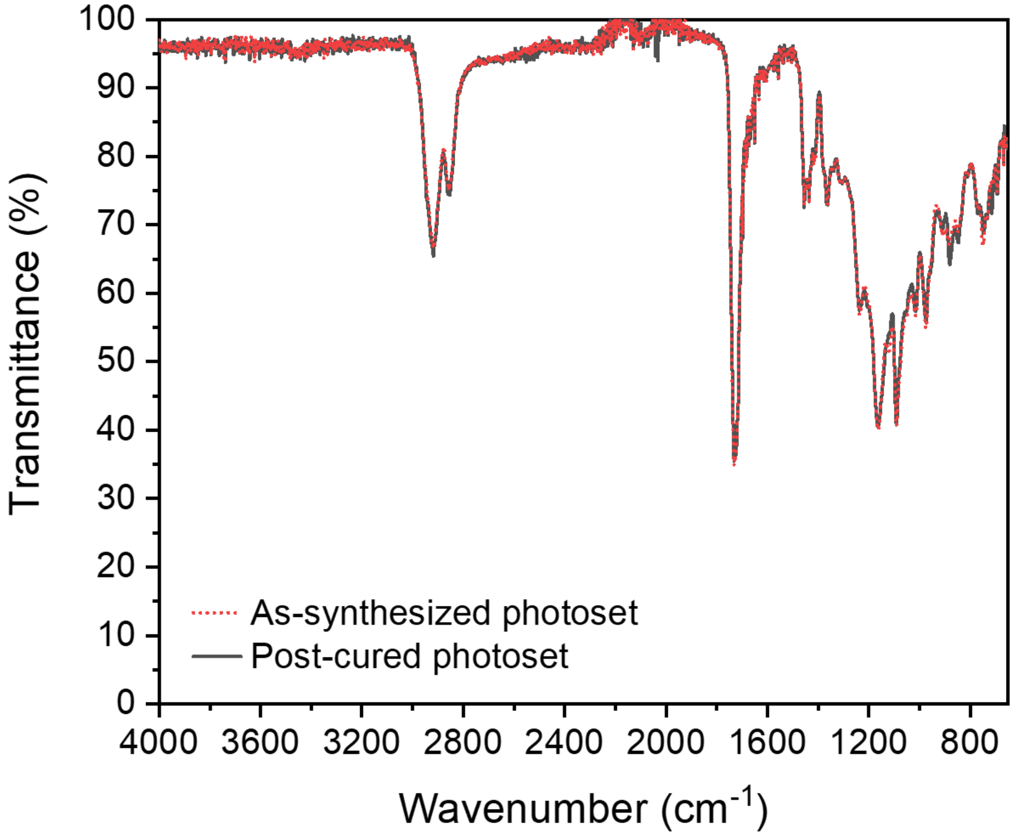

Fig. S53.

FT-IR spectra of $MenLp_1$:$IsoLp_2$ (30:70 wt.%) (as-synthesized & post-cured).

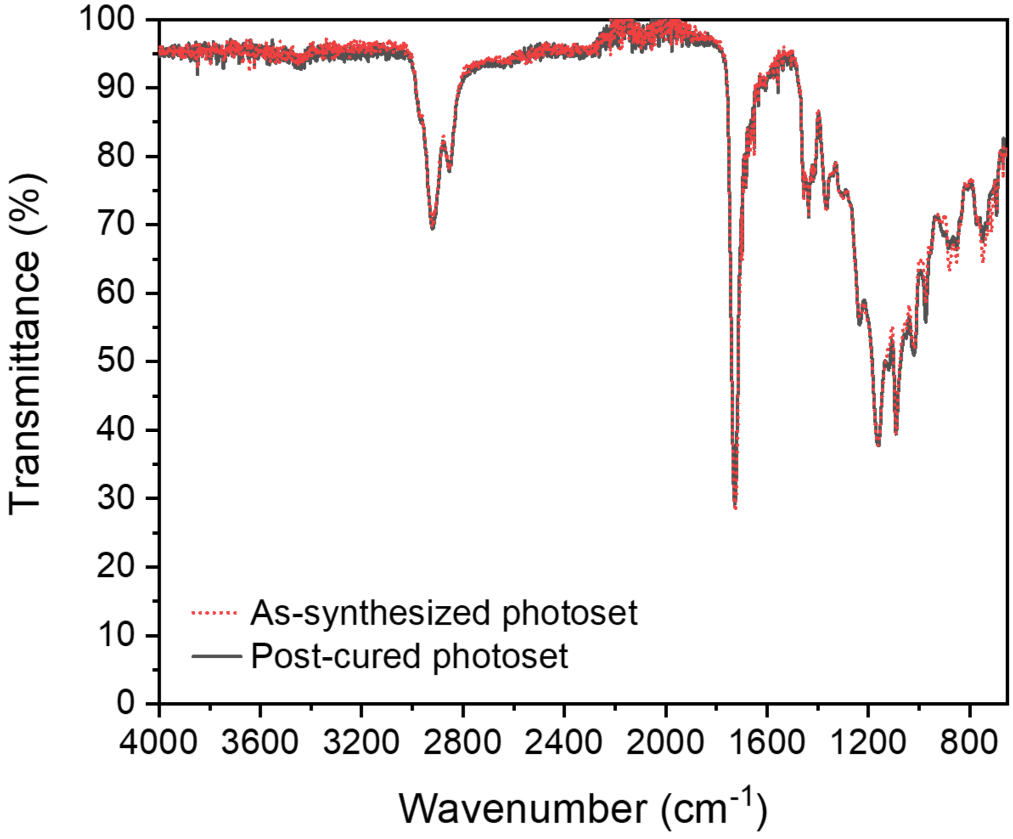

Fig. S54.

FT-IR spectra of $EtLp_1$:$IsoLp_2$ (30:70 wt.%) (as-synthesized & post-cured).

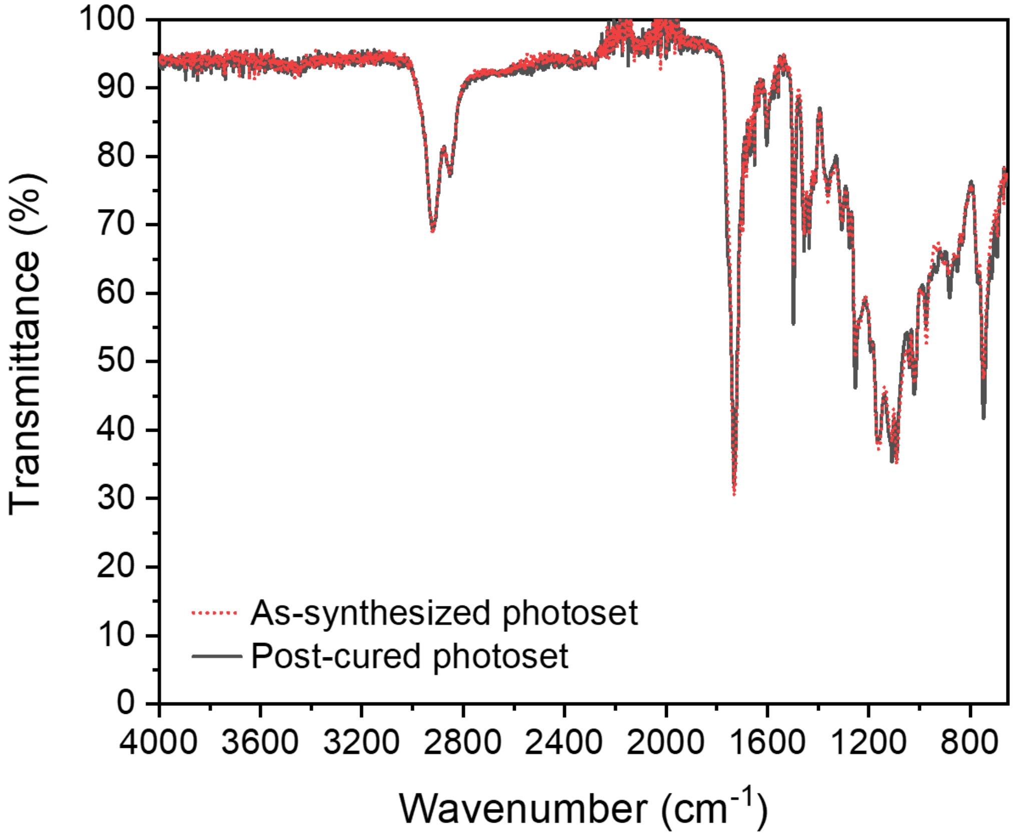

Fig. S55.
FT-IR spectra of $GuaLp_1$:$IsoLp_2$ (30:70 wt.%) (as-synthesized & post-cured).

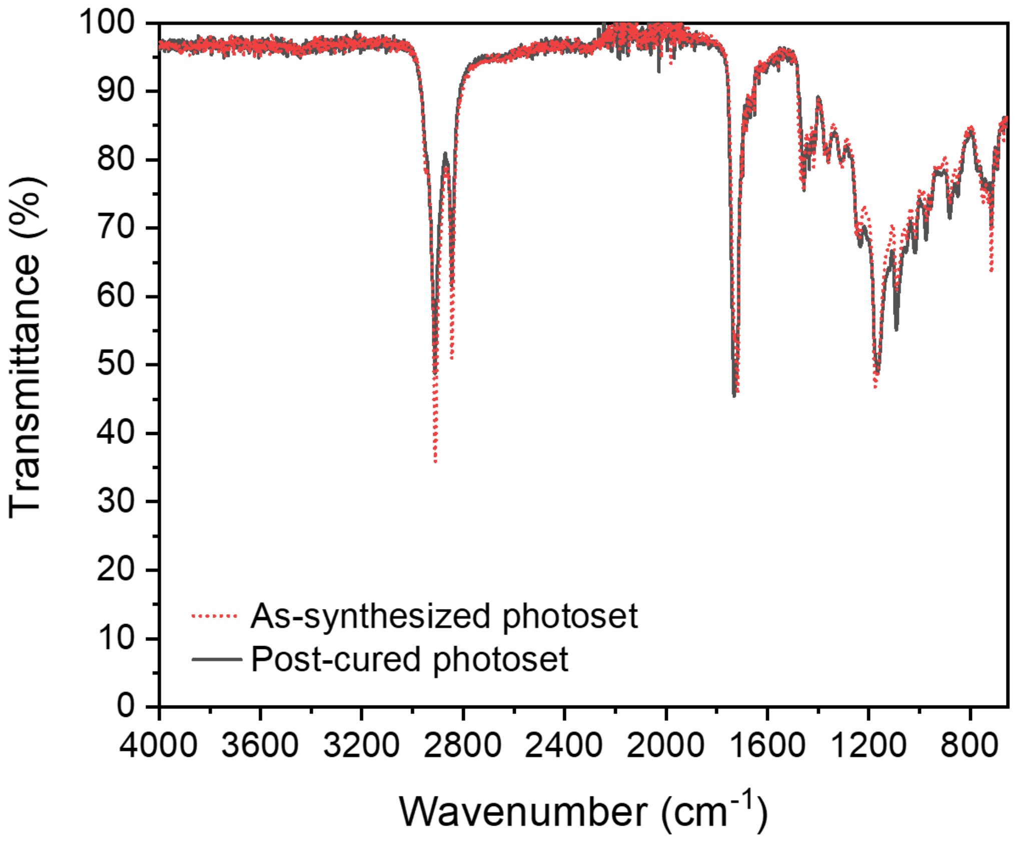

Fig. S56.

FT-IR spectra of SteaLp1:IsoLp2 (30:70 wt.%) (as-synthesized & post-cured).

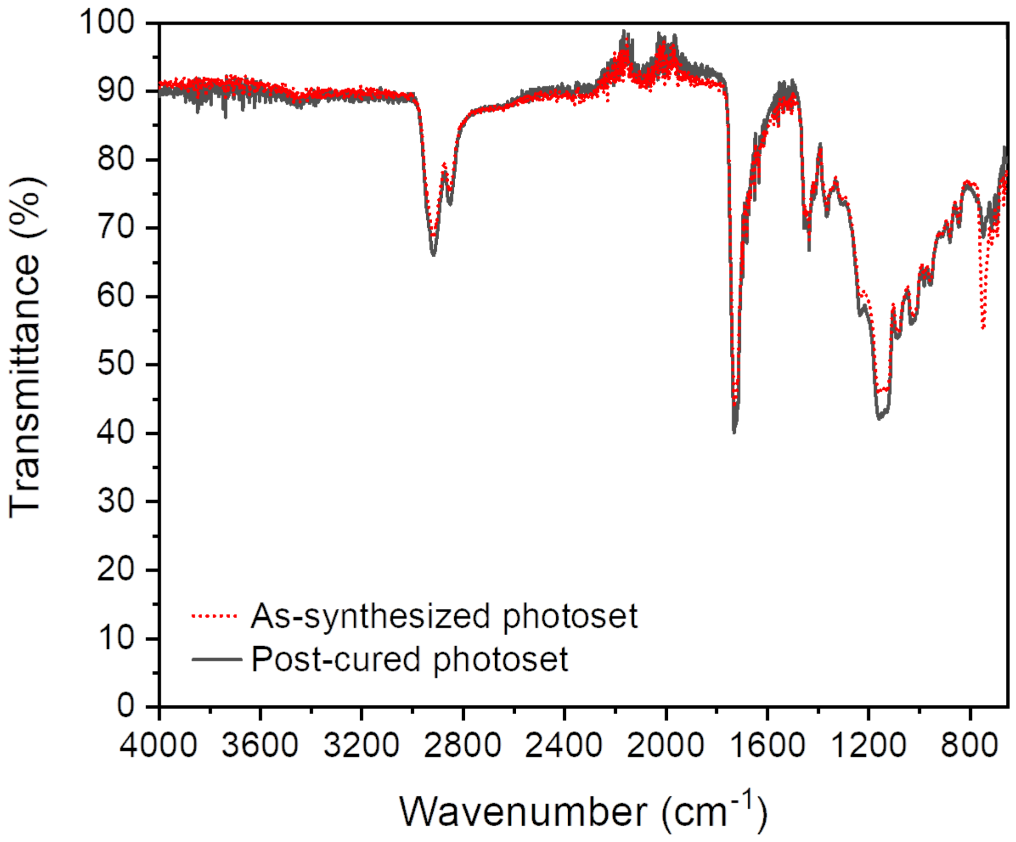

Fig. S57.

FT-IR spectra of MenLp1:GlyLp3 (30:70 wt.%) (as-synthesized & post-cured).

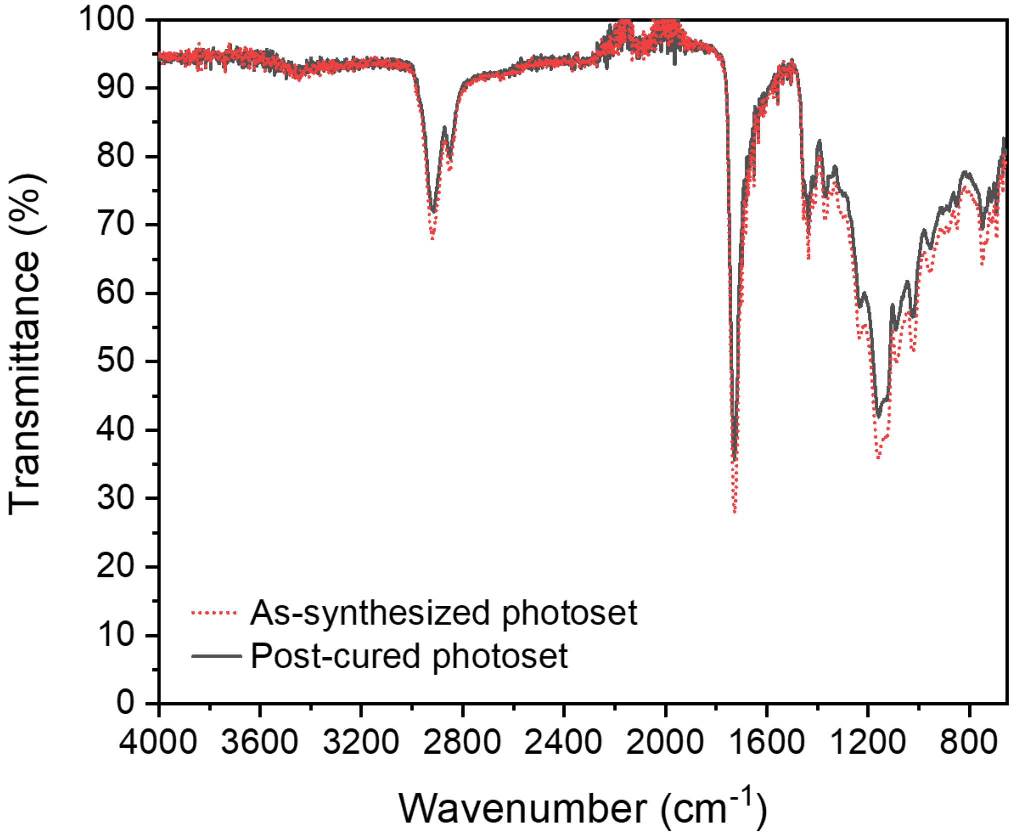

Fig. S58.
FT-IR spectra of $EtLp_1$:$GlyLp_3$ (30:70 wt.%) (as-synthesized & post-cured).

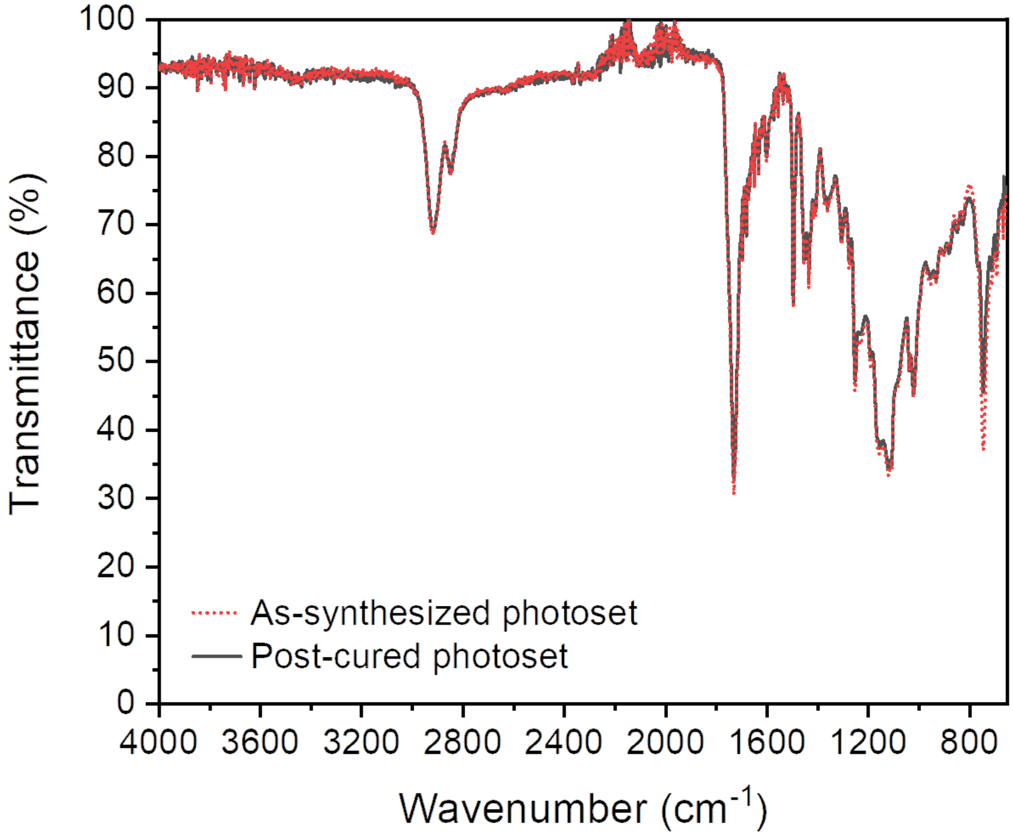

Fig. S59.
FT-IR spectra of $GuaLp_1$:$GlyLp_3$ (30:70 wt.%) (as-synthesized & post-cured).

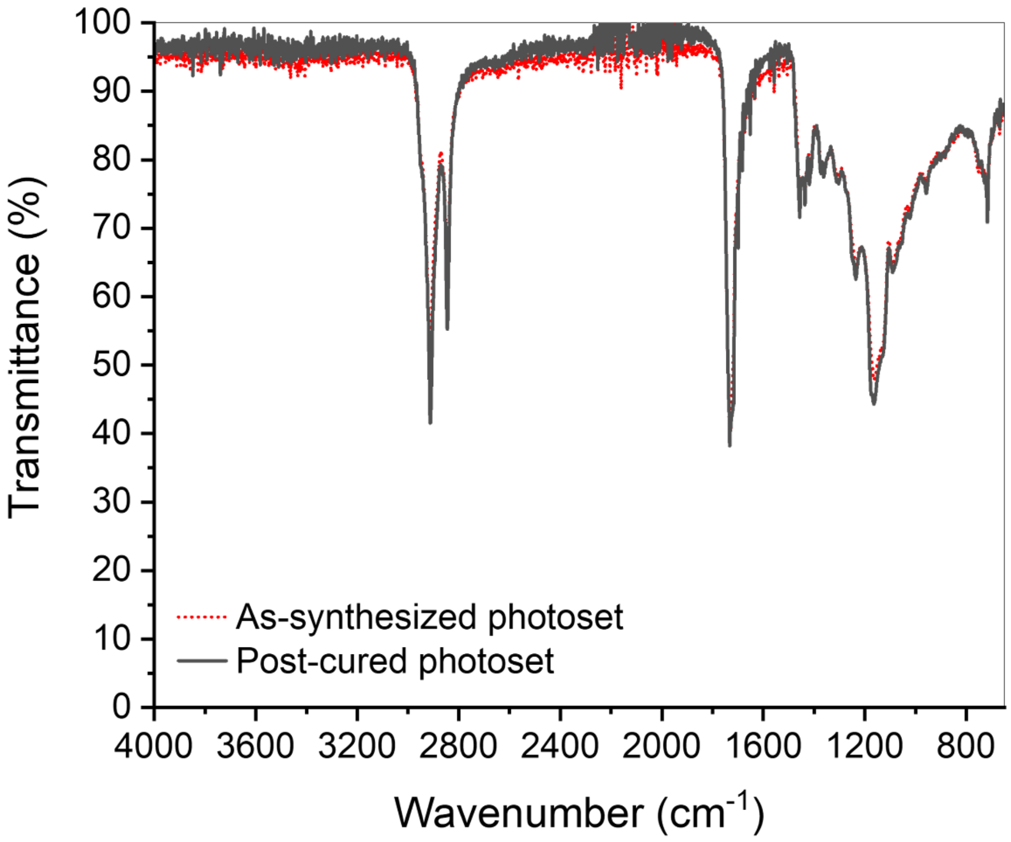

Fig. S60.
FT-IR spectra of $SteaLp_1$:$GlyLp_3$ (30:70 wt.%) (as-synthesized & post-cured).

2D-photoset recycling

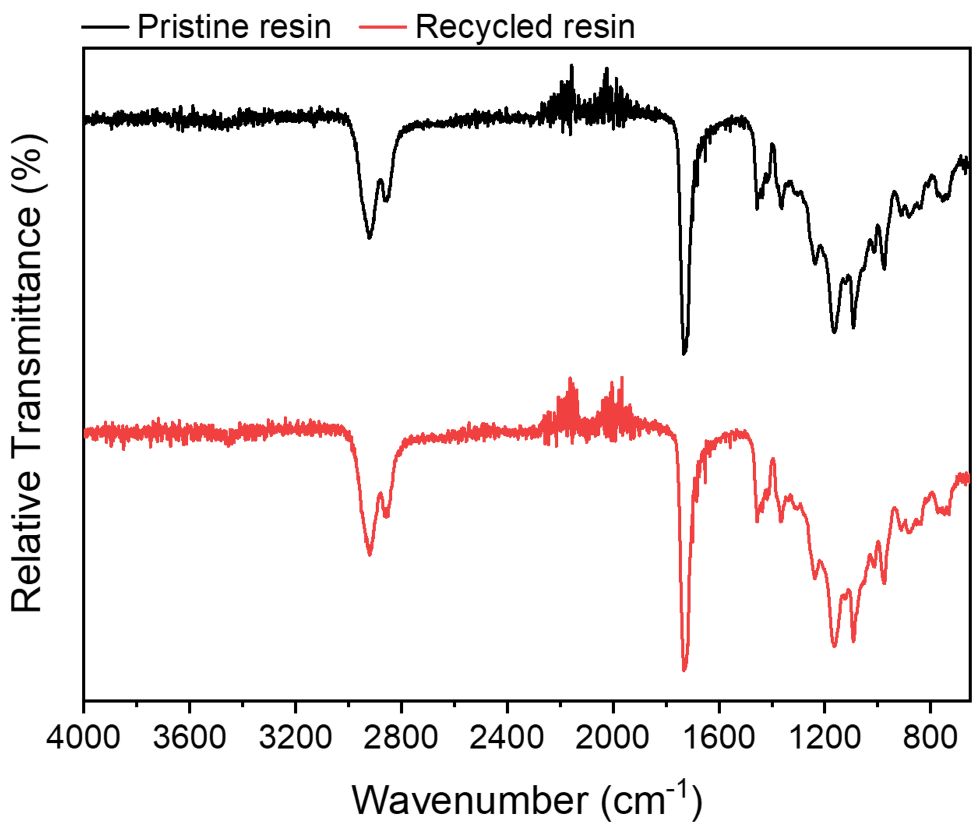

Fig. S61.

FT-IR spectra of MenLp1:IsoLp2 (30:70 wt.%). Pristine & recycled using catalyzed depolymerization (phosphazene:thiophenol) method.

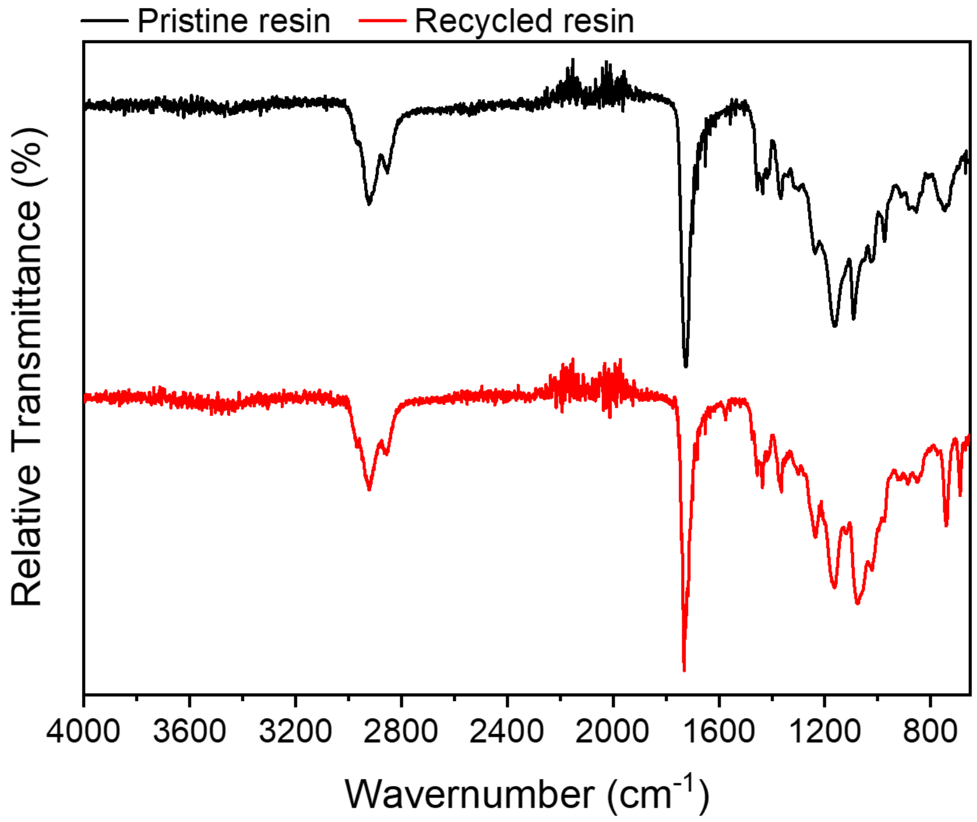

Fig. S62.

FT-IR spectra of $EtLp_1$:$IsoLp_2$ (30:70 wt.%). Pristine & recycled using catalyzed depolymerization (phosphazene:thiophenol) method.

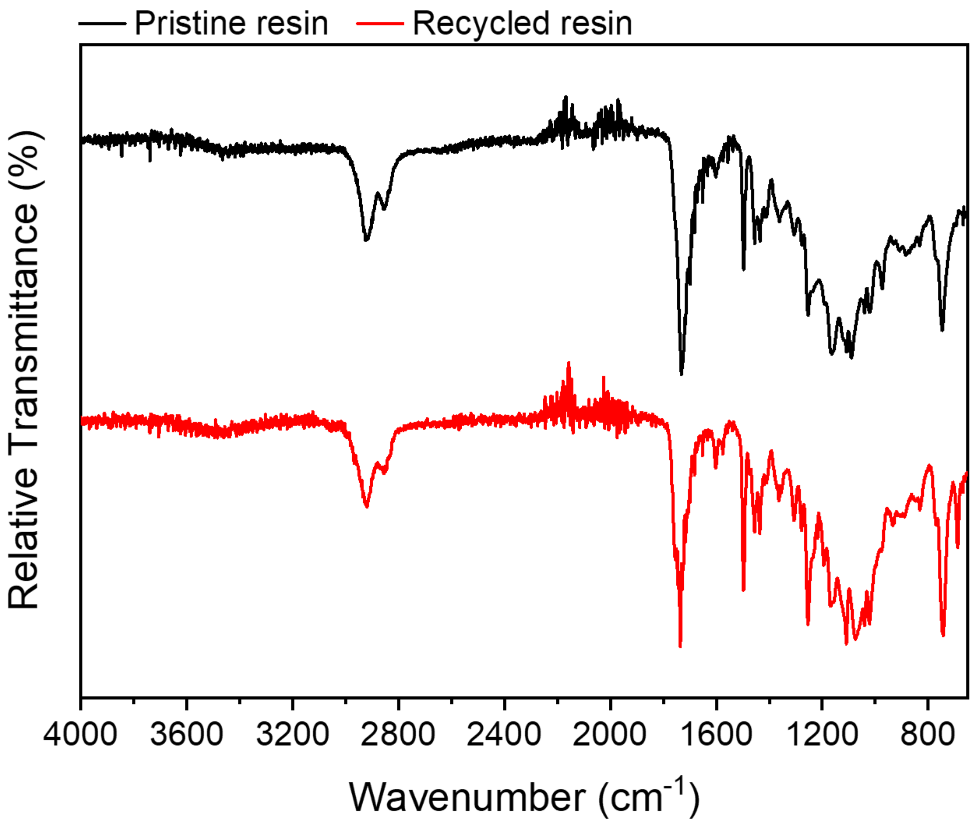

Fig. S63.

FT-IR spectra of $GuaLp_1$:$IsoLp_2$ (30:70 wt.%). Pristine & recycled using catalyzed depolymerization (phosphazene:thiophenol) method.

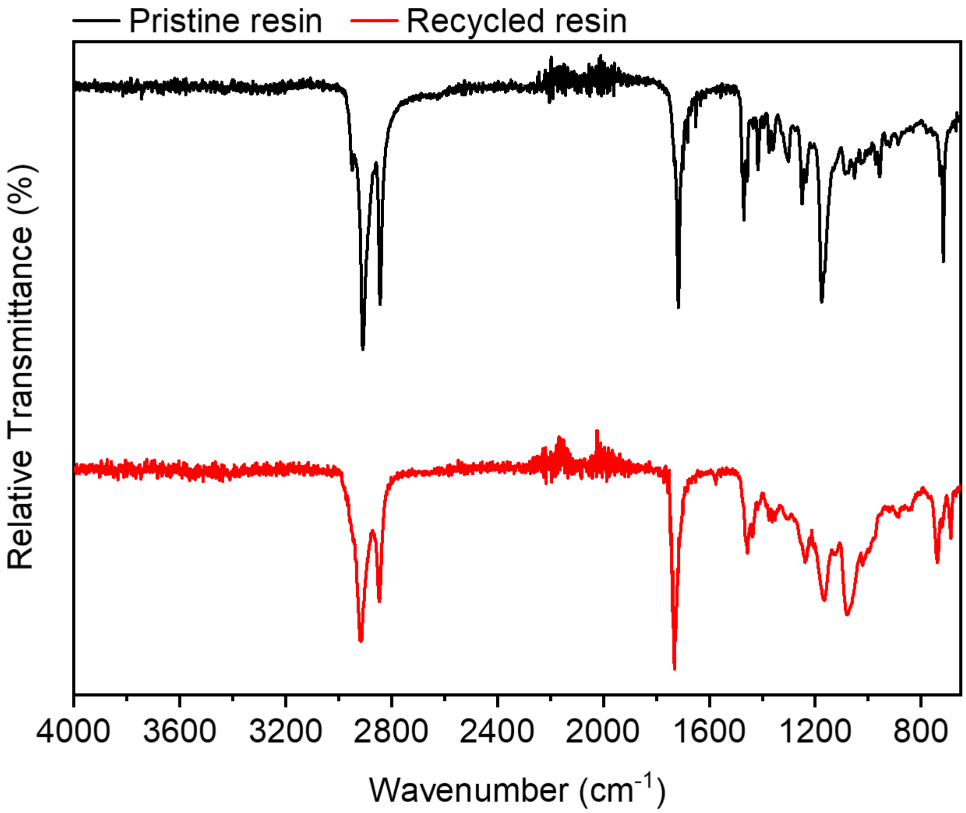

Fig. S64.

FT-IR spectra of SteaLp1:IsoLp2 (30:70 wt.%). Pristine & recycled using catalyzed depolymerization (phosphazene:thiophenol) method.

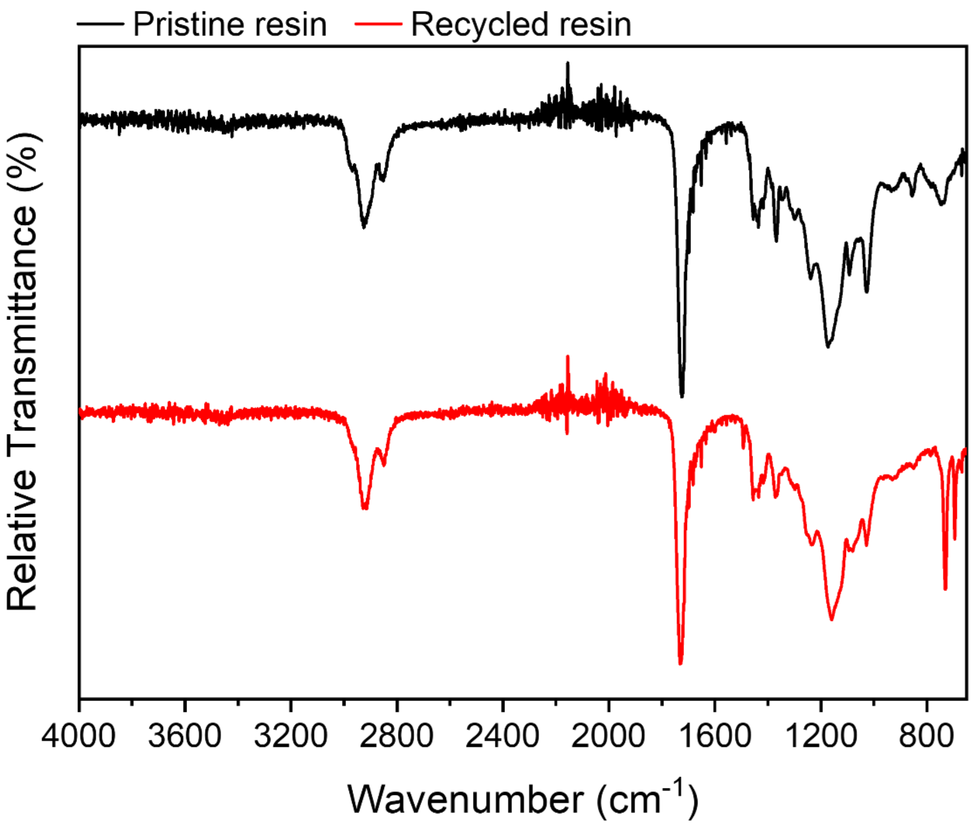

Fig. S65.
FT-IR spectra of SteaLp1:IsoLp2 (30:70 wt.%). Pristine and recycled using thermal depolymerization (DMF, 140 °C) method.

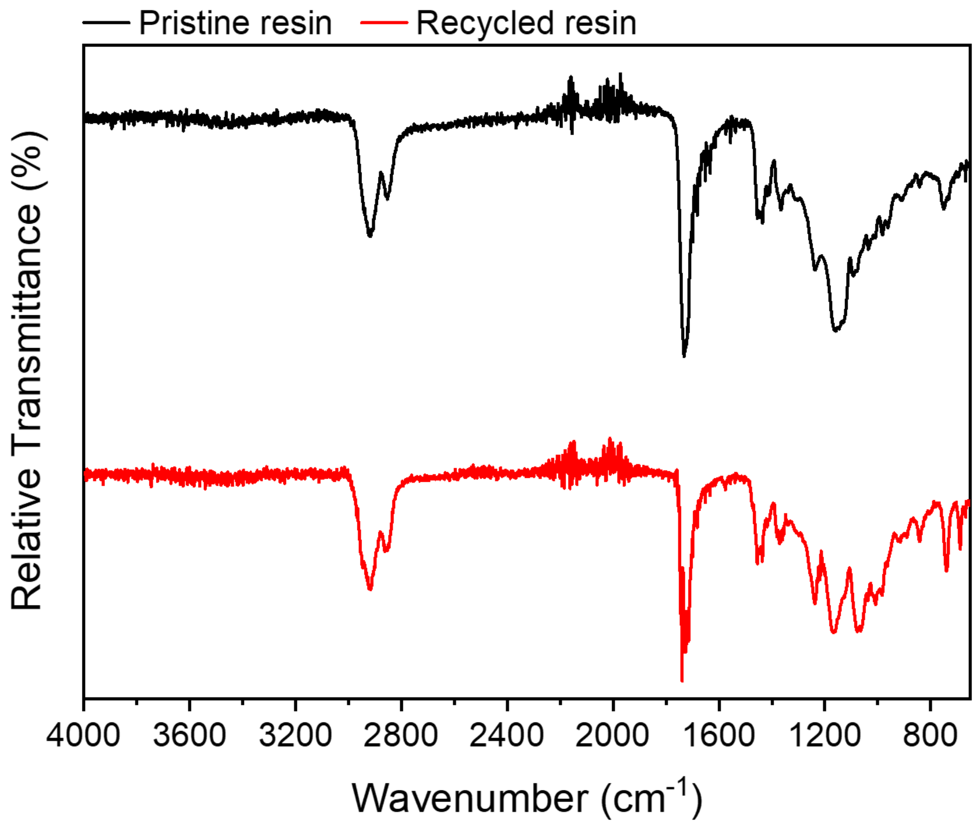

Fig. S66.

FT-IR spectra of MenLp1:GlyLp3 (30:70 wt.%). Pristine & recycled using catalyzed depolymerization (phosphazene:thiophenol) method.

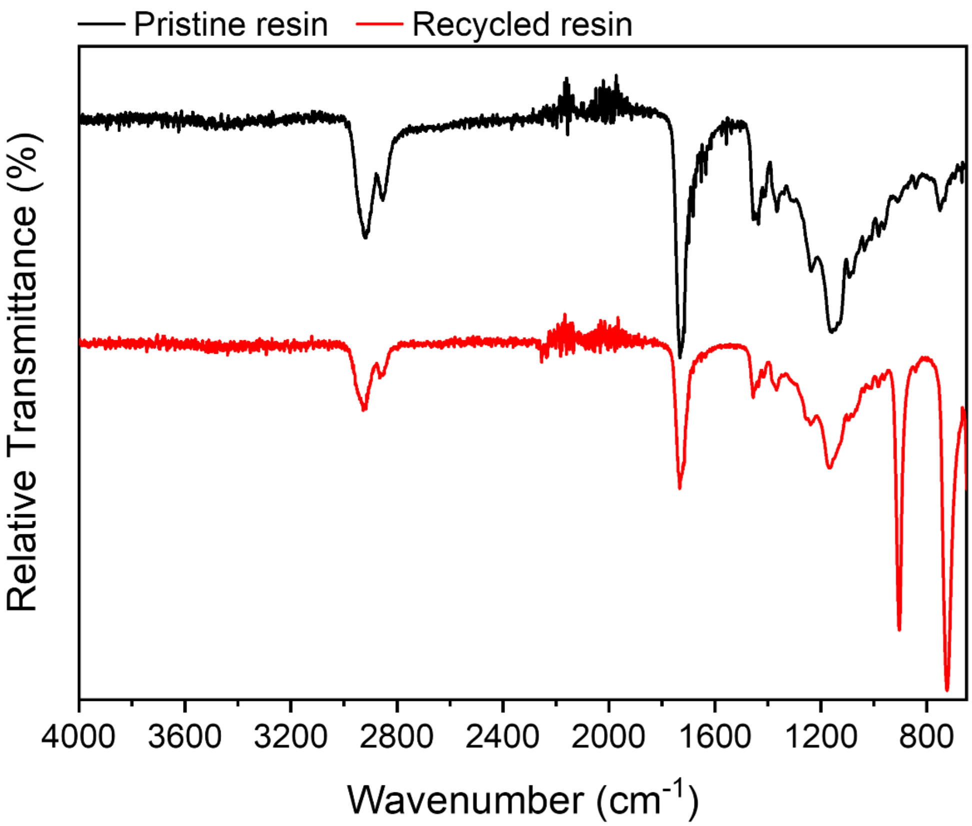

Fig. S67.
FT-IR spectra of MenLp1:GlyLp3 (33:67 wt.%). Pristine & recycled using thermal depolymerization (DMF, 140 °C) method.

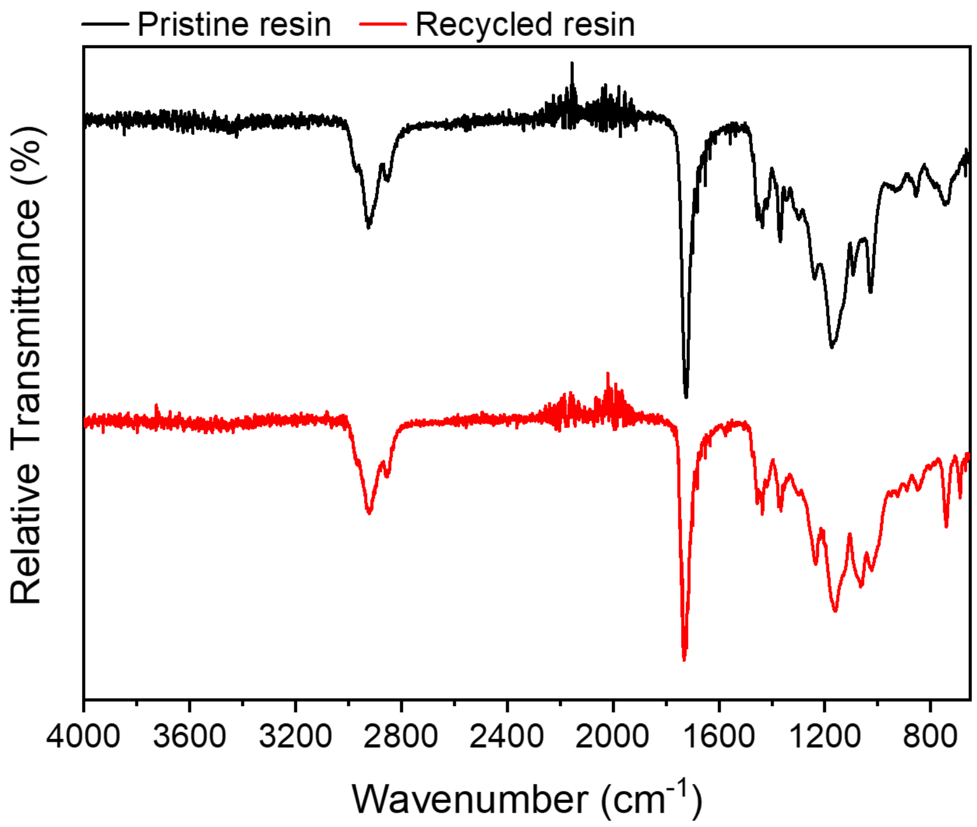

Fig. S68.

FT-IR spectra of $EtLp_1$:$GlyLp_3$ (30:70 wt.%) Pristine & recycled using catalyzed depolymerization (phosphazene:thiophenol) method.

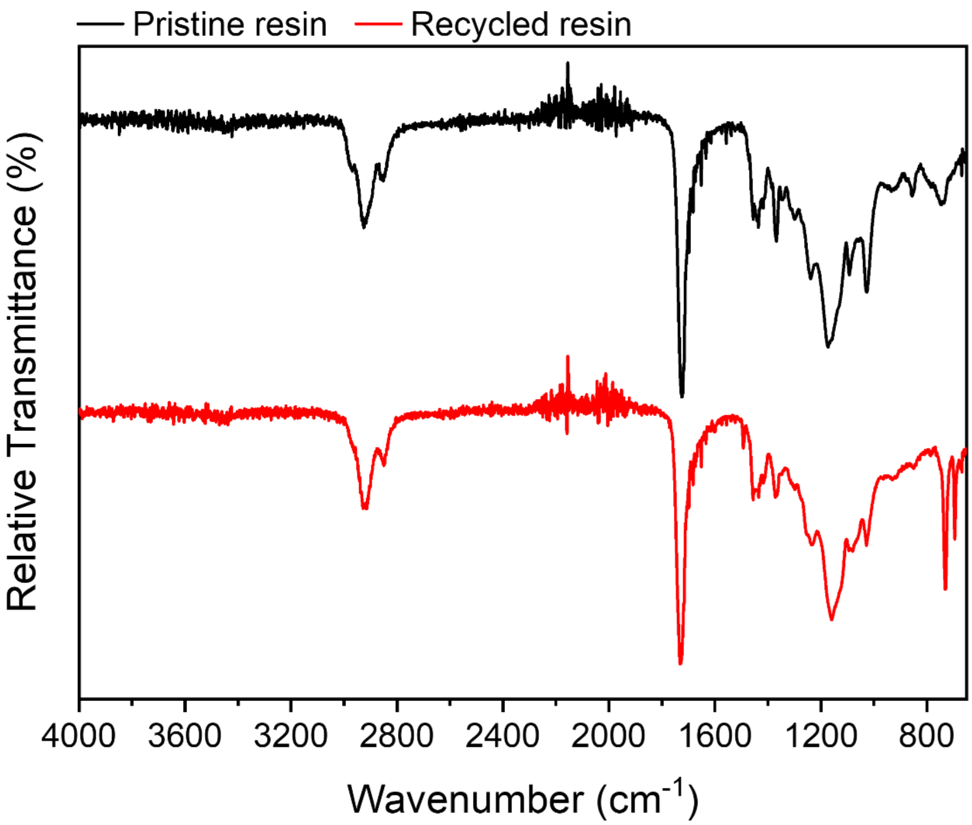

Fig. S69.
FT-IR spectra of EtLp1:GlyLp3 (34:66 wt.%). Pristine & recycled using thermal depolymerization (DMF, 140 °C) method.

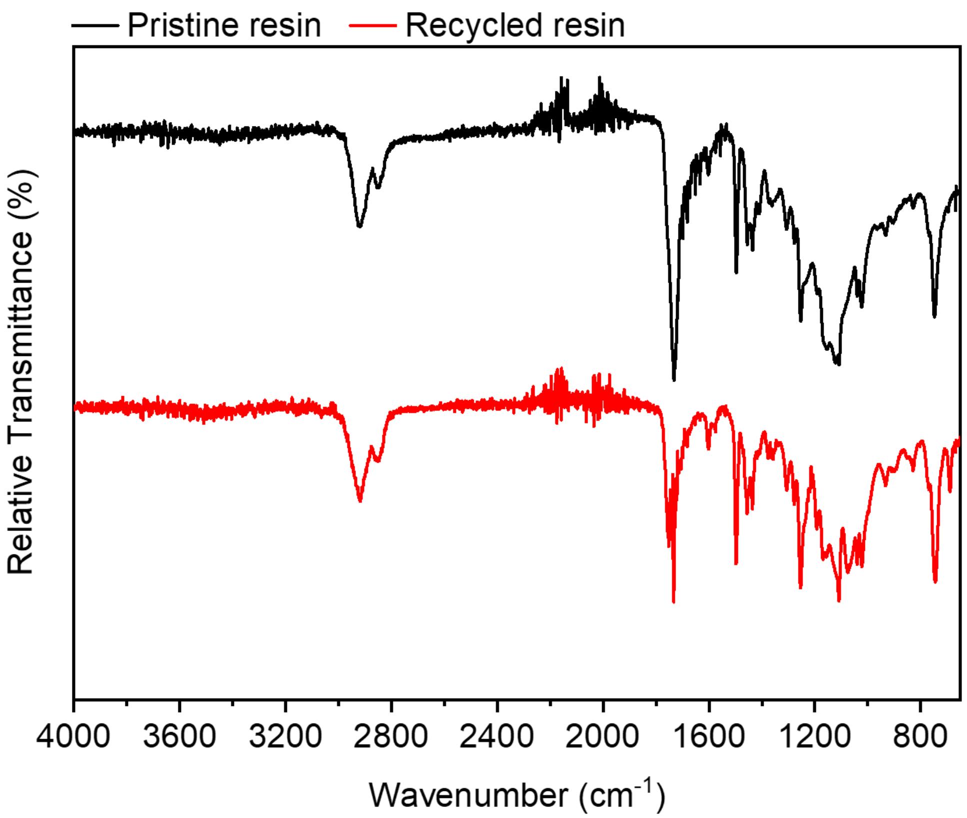

Fig. S70.

FT-IR spectra of GuaLp1:GlyLp3 (30:70 wt.%) Pristine & recycled using catalyzed depolymerization (phosphazene:thiophenol) method.

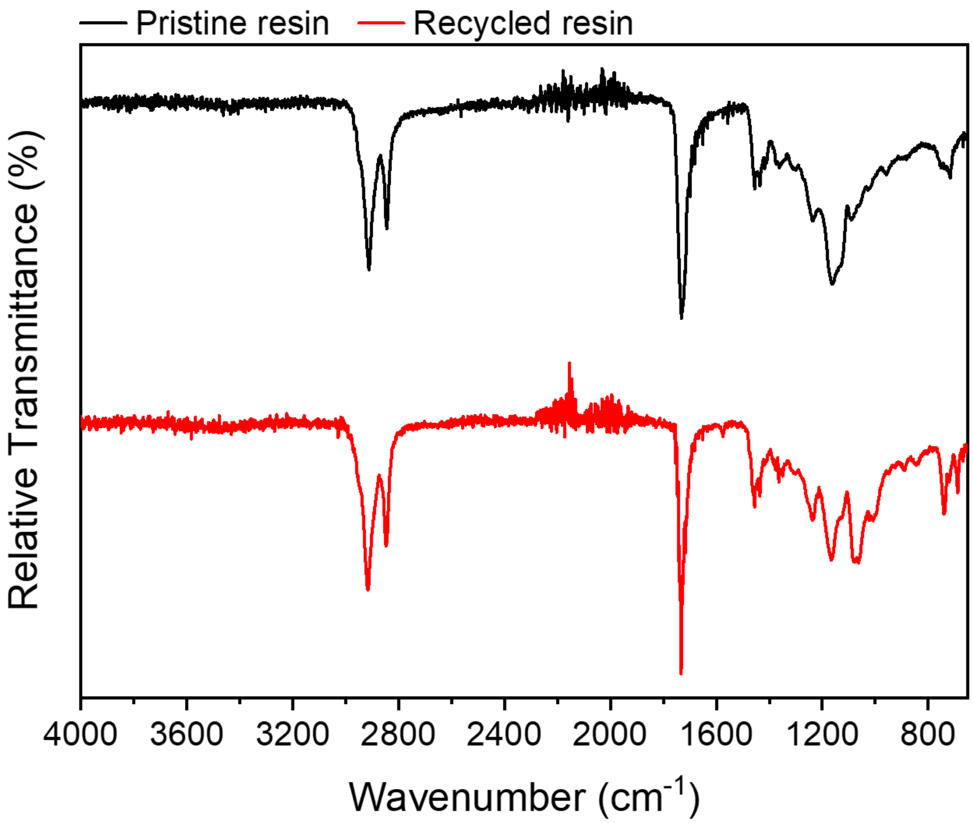

Fig. S71.

FT-IR spectra of SteaLp1:GlyLp3 (30:70 wt.%) Pristine & recycled using catalyzed depolymerization (phosphazene:thiophenol) method.

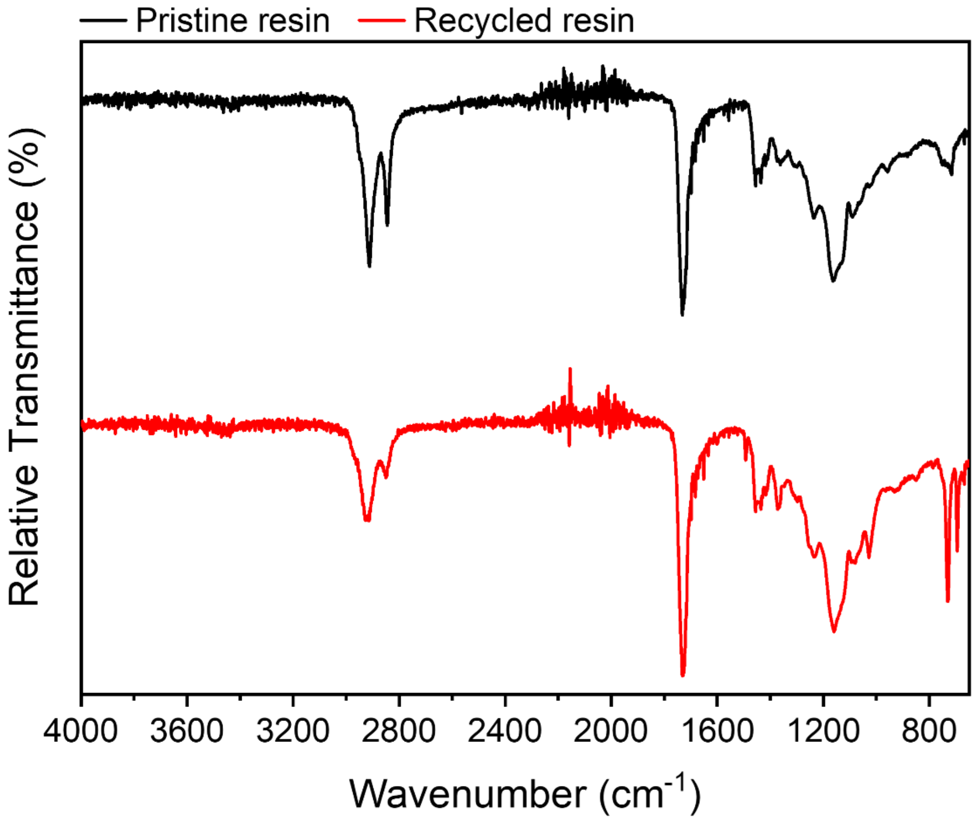

Fig. S72.
FT-IR spectra of $SteaLp_1$:$GlyLp_3$ (32:68 wt.%). Pristine & recycled using thermal depolymerization (DMF, 140 °C) method.

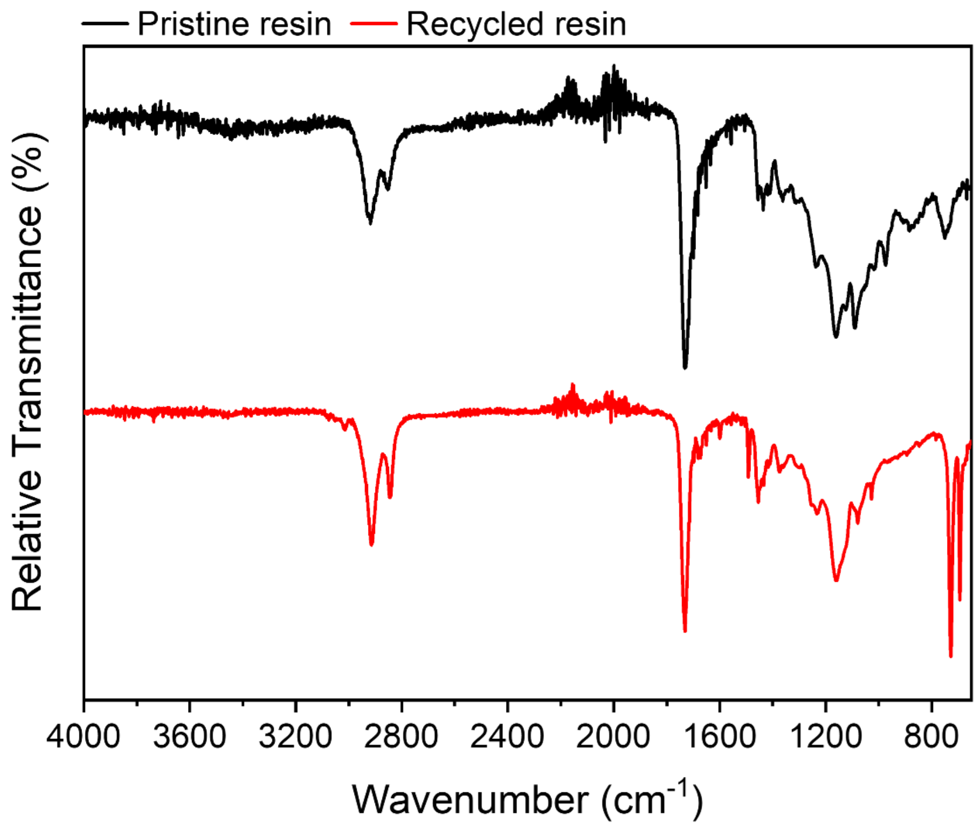

Fig. S73.
FT-IR spectra of IsoLp2:GlyLp3 (70:30 wt.%). Pristine & recycled using thermal depolymerization (DMF, 140 °C) method.

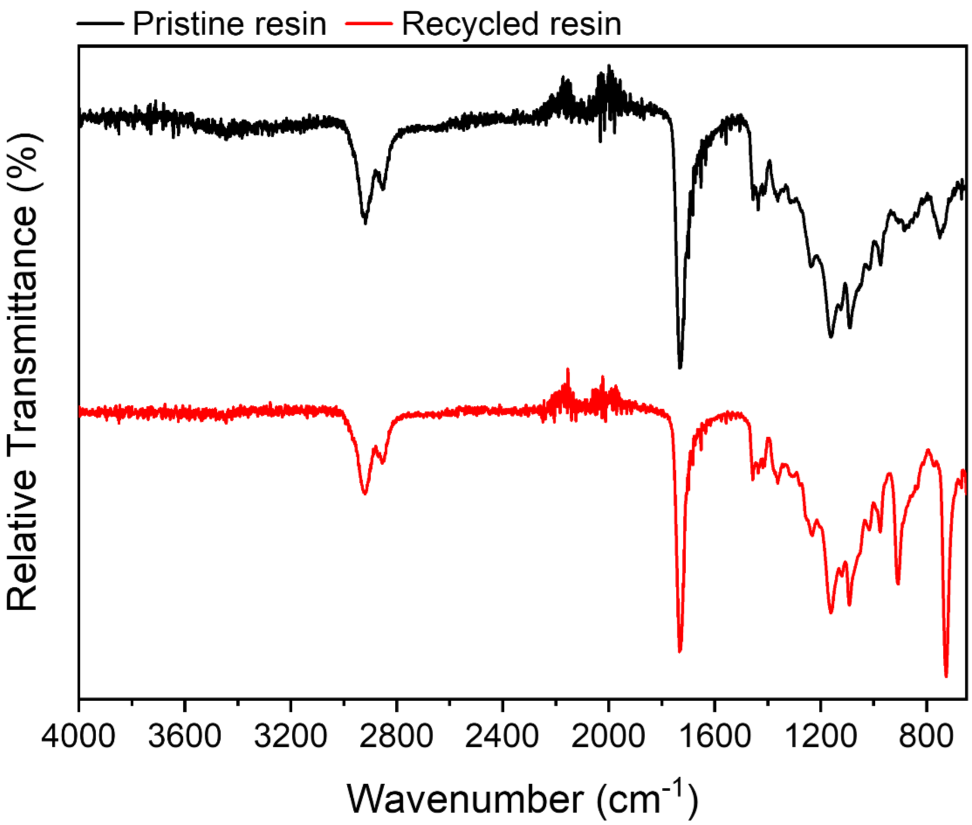

Fig. S74.
FT-IR spectra of IsoLp2:GlyLp3 (28:72 wt.%). Pristine & recycled using thermal depolymerization (DMF, 140 °C) method.

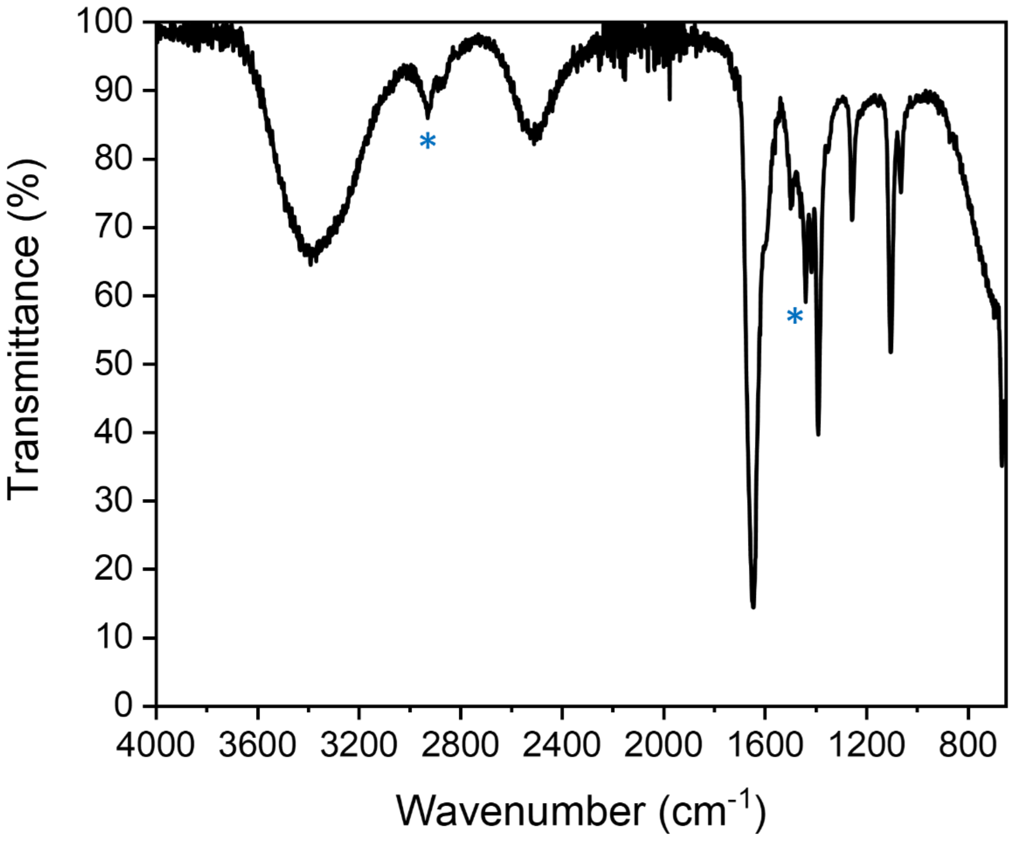

Fig. S75.
FT-IR spectra of recycled 3D-printed $MenLp_1$:$IsoLp_2$ (30:70 wt.%). Recycled using hydrolytic depolymerization method (Table S4). *DMF

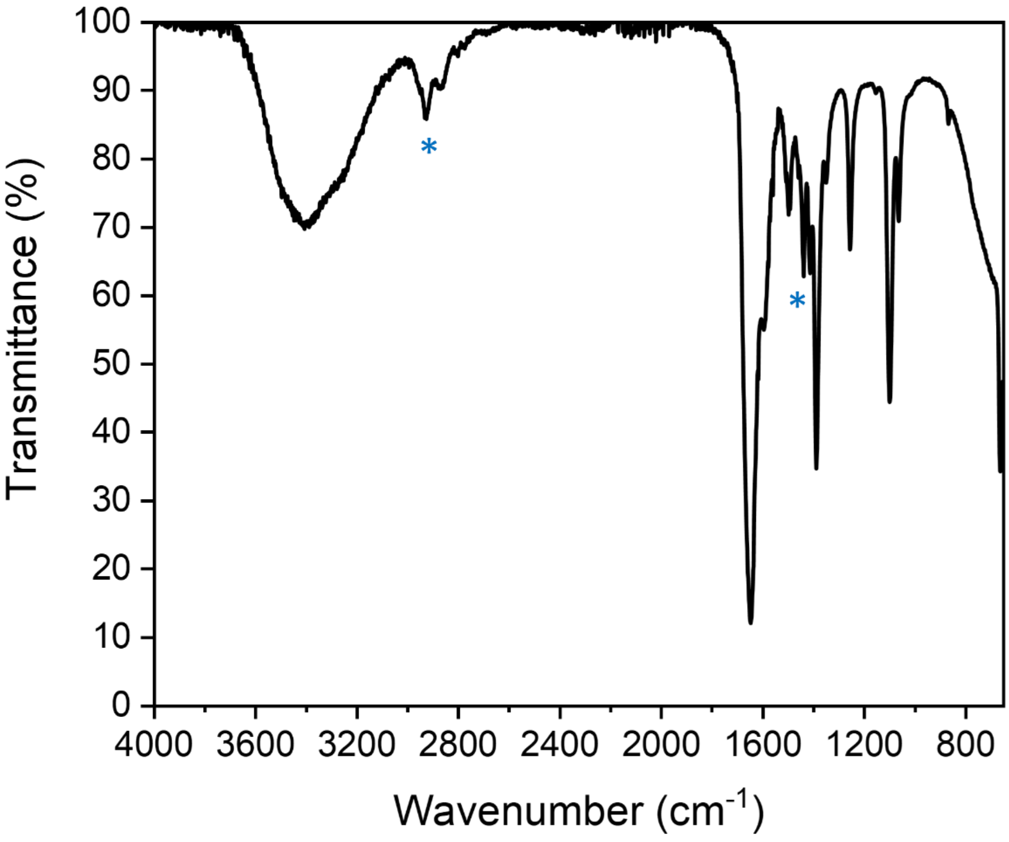

Fig. S76.
FT-IR spectra of recycled 3D-printed $EtLp_1$:$GlyLp_3$ (31:69 wt.%). Recycled using hydrolytic depolymerization method (Table S4). *DMF

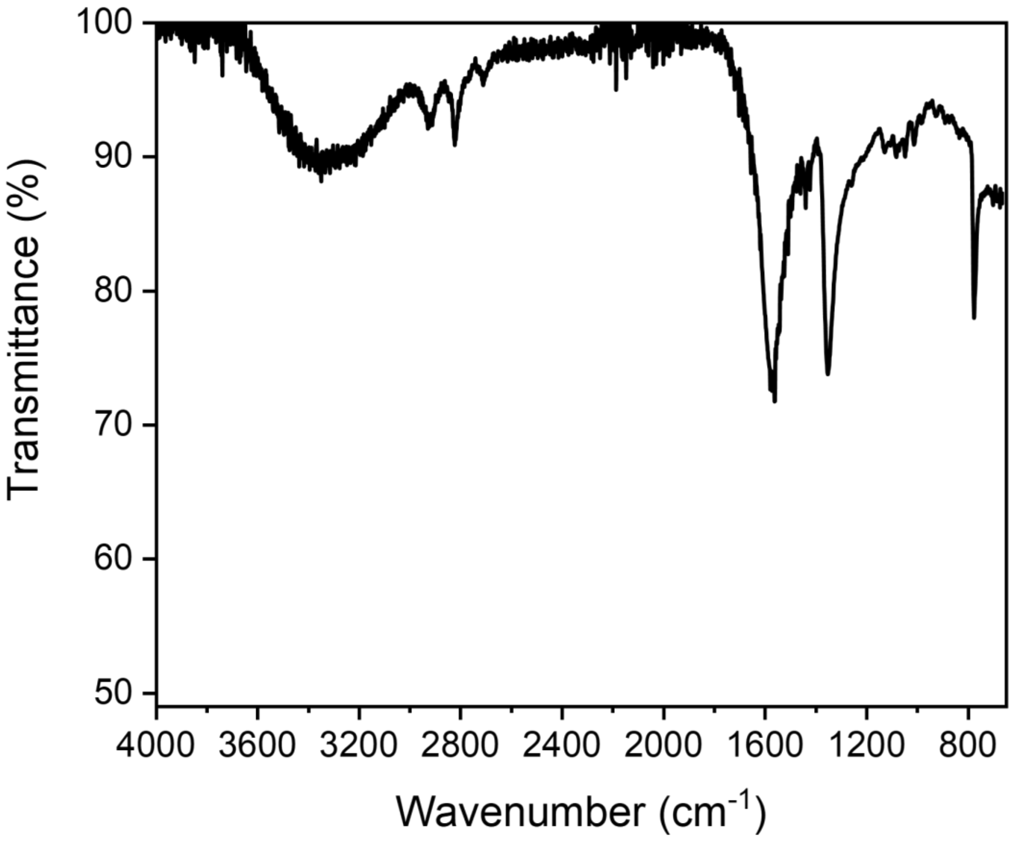

Fig. S77.
FT-IR spectra of recycled $Stea_1$:$IsoLp_2$ (30:70 wt.%) 2D photoset. Recycled using hydrolytic depolymerization method (Table S4).

3D-prints

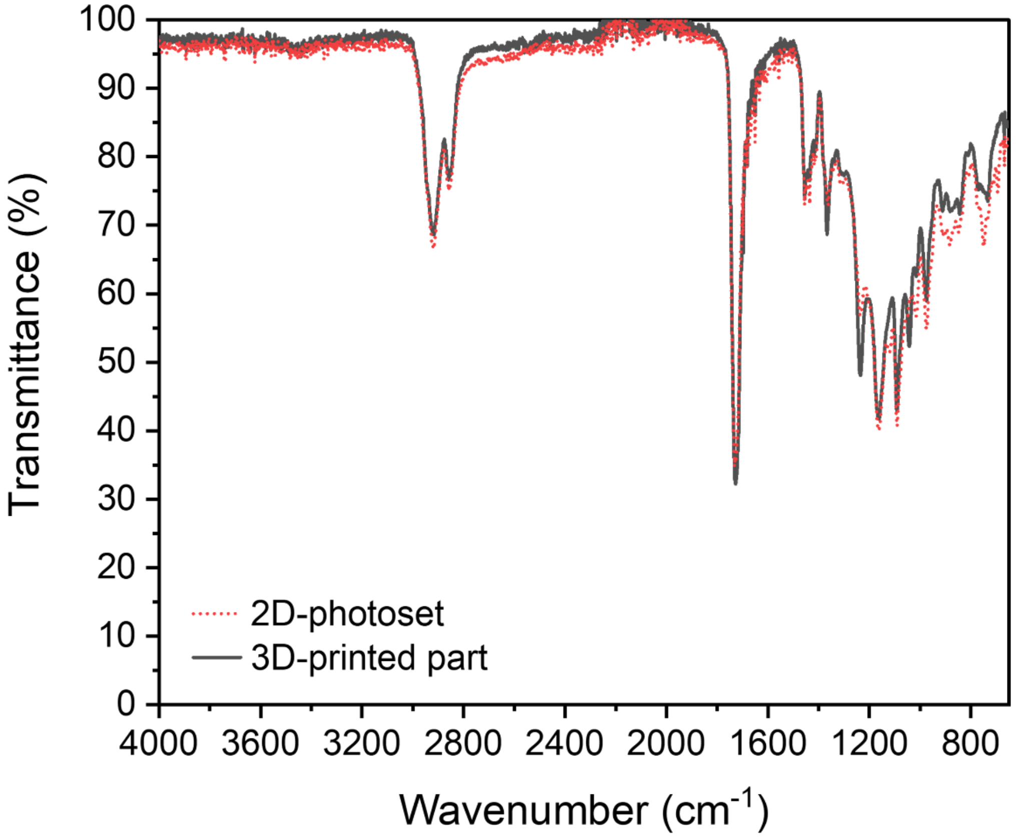

Fig. S78.
FT-IR spectra of MenLp1:IsoLp2 (30:70 wt.%) 2D-photoset *vs* 3D-printed part (both samples as-synthesized).

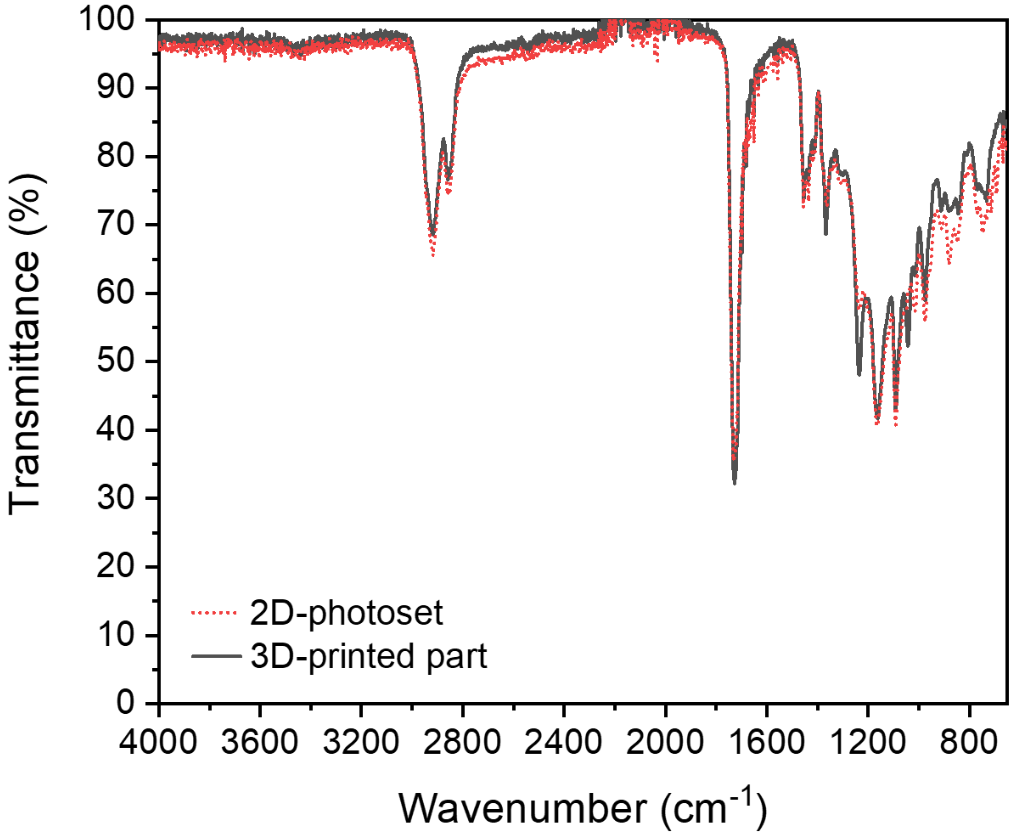

Fig. S79.
FT-IR spectra of MenLp1:IsoLp2 (30:70 wt.%) 2D-photoset *vs* 3D-printed part (both samples post-cured).

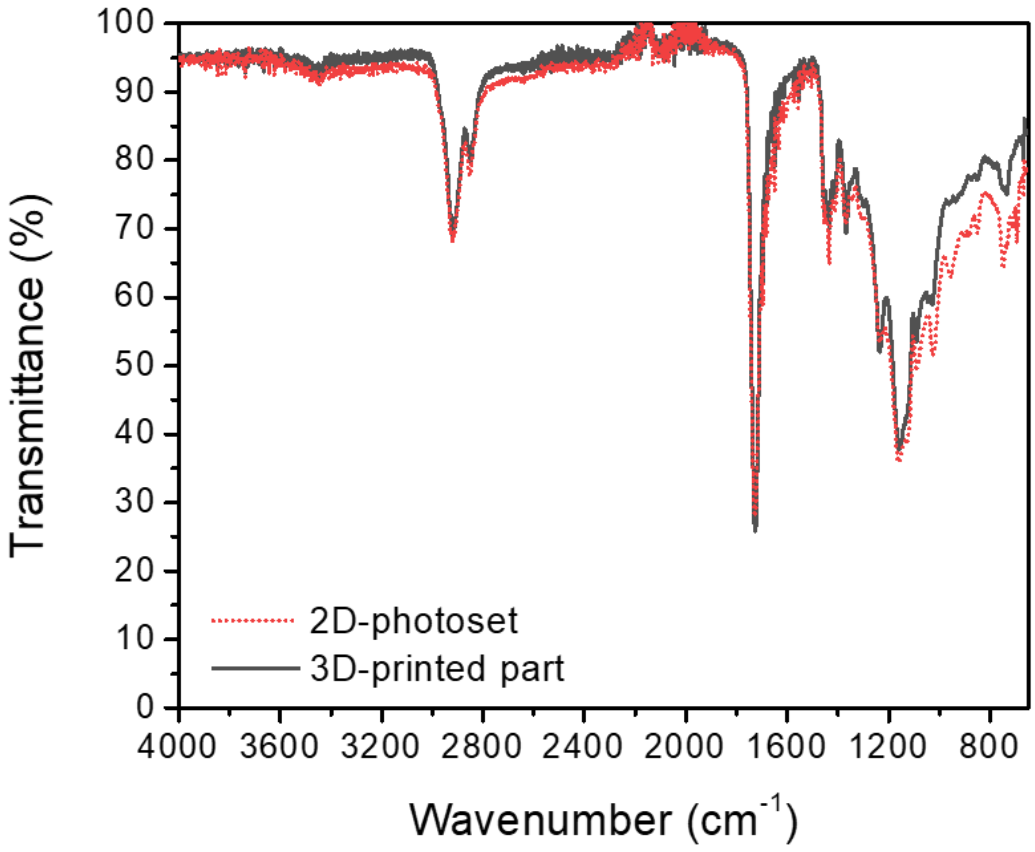

Fig. S80.

FT-IR spectra of $EtLp_1$:$GlyLp_3$ (30:70 wt.%) 2D-photoset *vs* 3D-printed part (both samples as-synthesized).

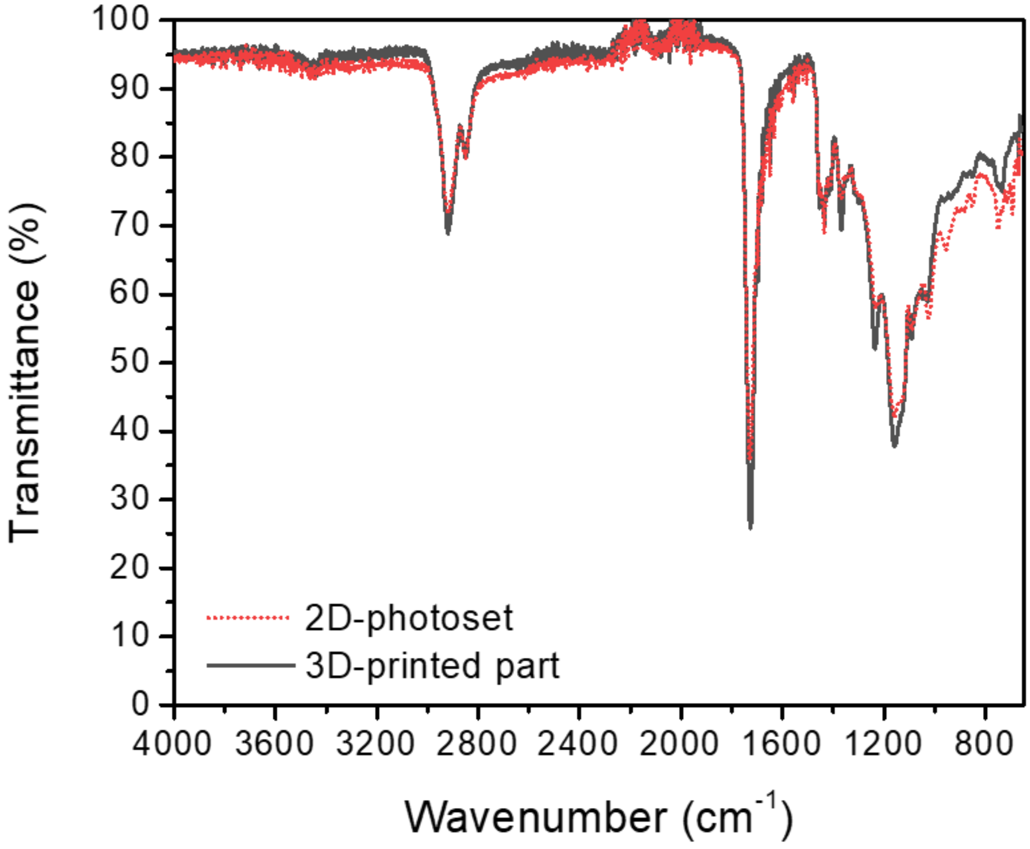

Fig. S81.

FT-IR spectra of $EtLp_1$:$GlyLp_3$ (30:70 wt.%) 2D-photoset *vs* 3D-printed part (both samples post-cured).

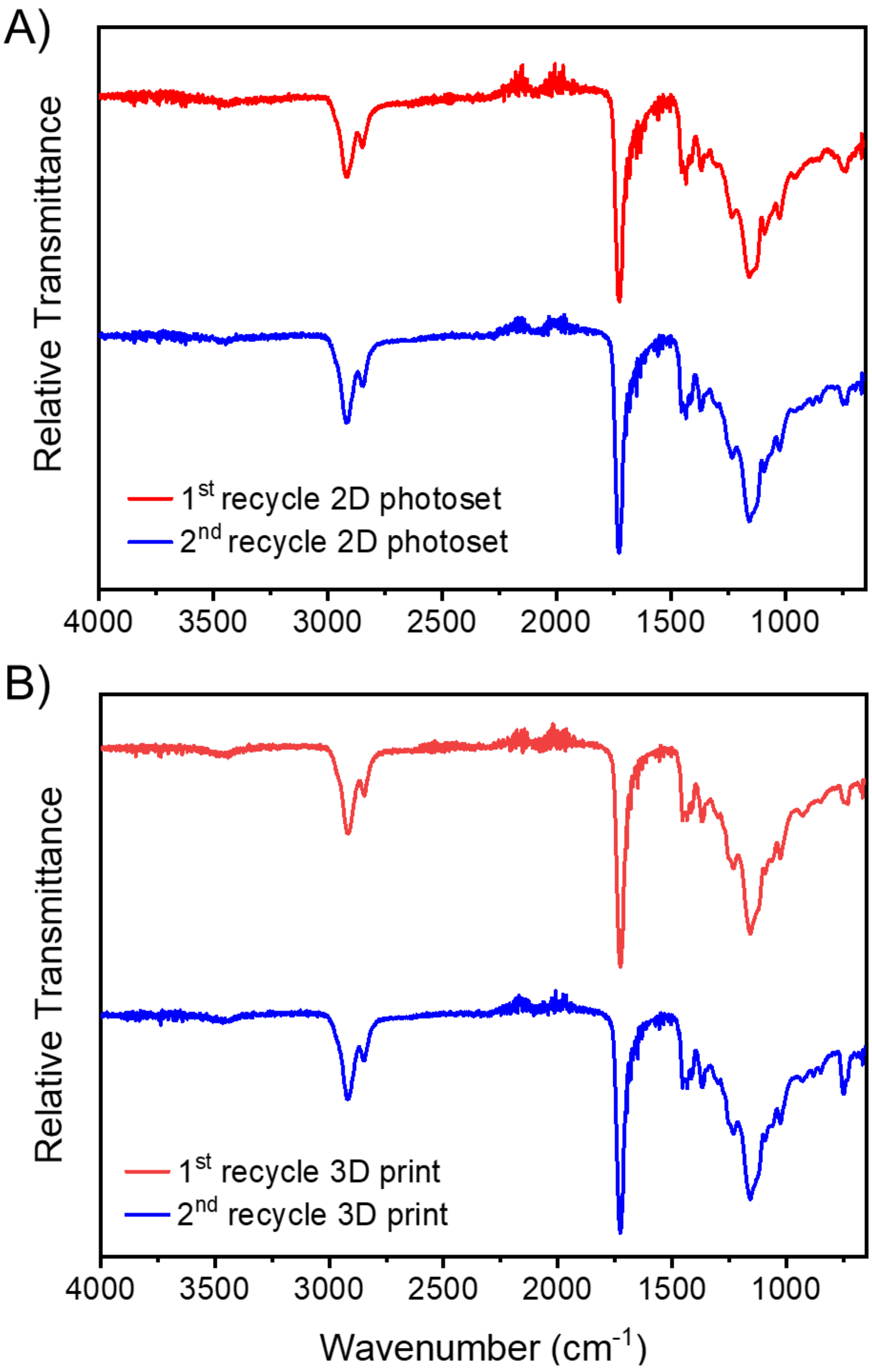

Fig. S82.
FT-IR spectra of $EtLp_1$:$GlyLp_3$ (30:70 wt.%) A) 2D-photoset *vs* B) 3D-printed part after multiple recycles.

Size-exclusion chromatography (SEC) data for depolymerization of 2D-photosets

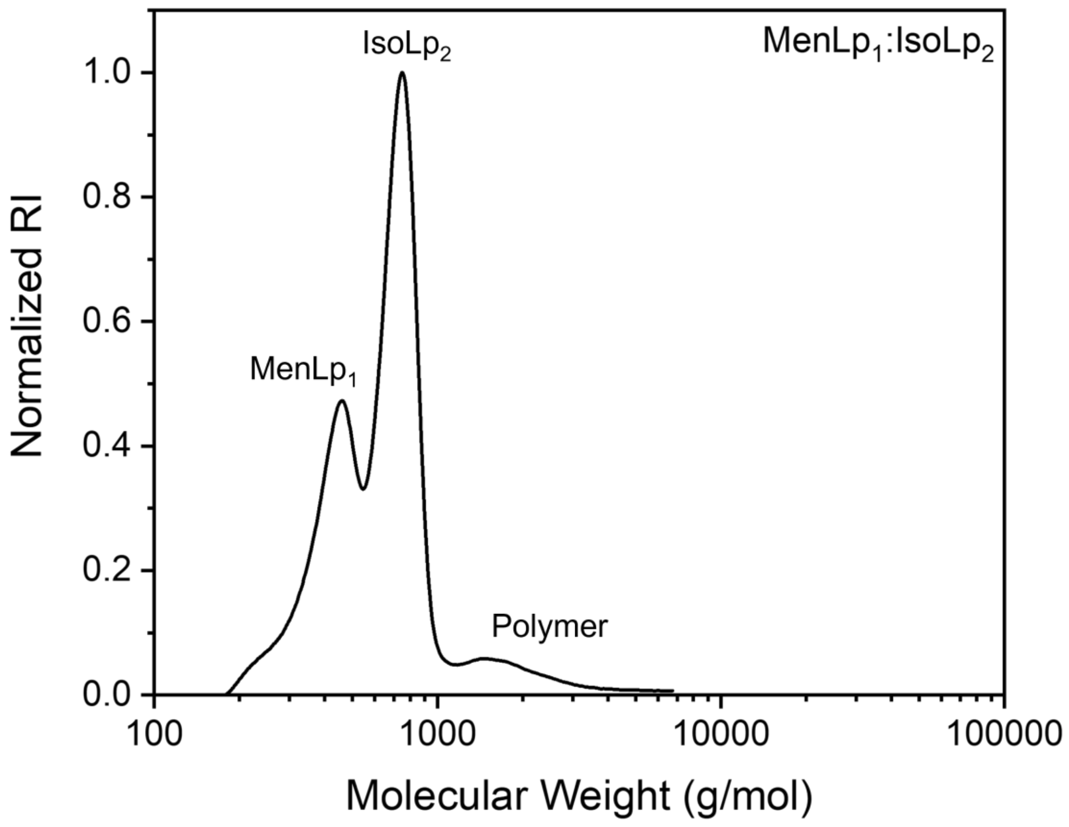

Fig. S83.
SEC chromatogram for 2D-photoset catalyzed depolymerization (phosphazene:thiophenol method) obtained from $MenLp_1$:$IsoLp_2$ (30:70 wt%) resin ($CHCl_3$, 0.5 % w/w NEt_3) determined against polystyrene (PS) standards.

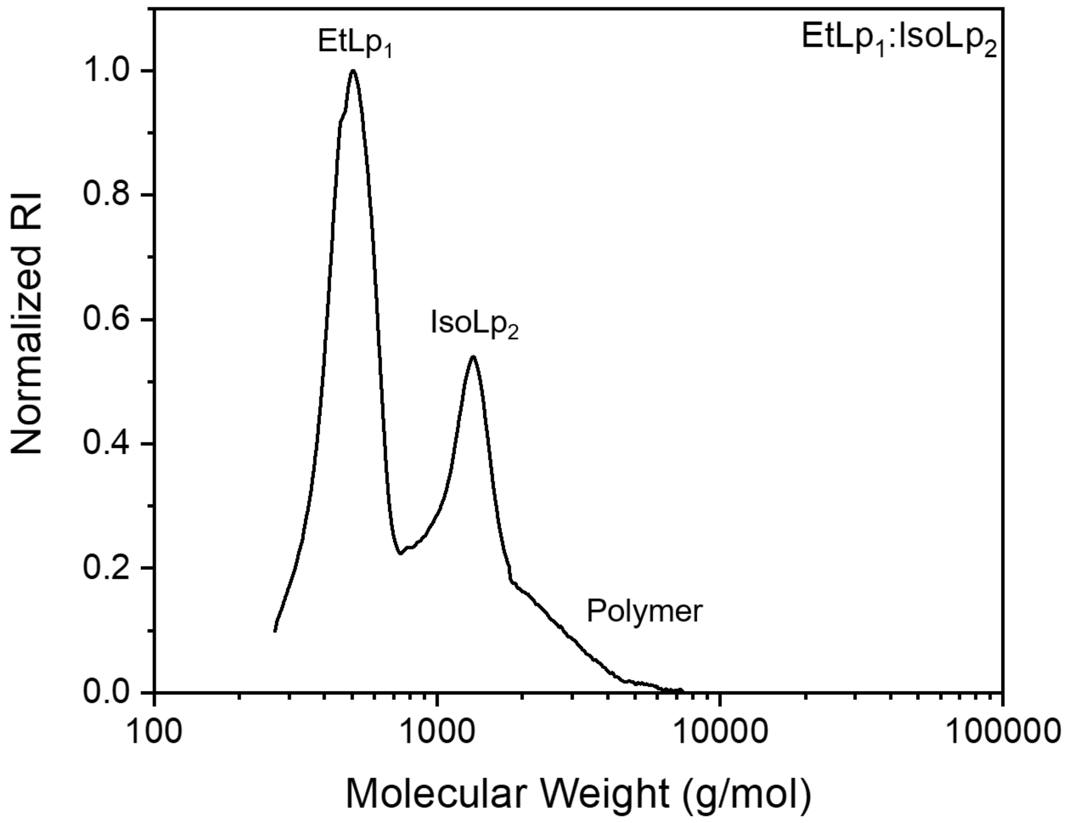

Fig. S84.
SEC chromatogram for 2D-photoset catalyzed depolymerization (phosphazene:thiophenol method) obtained from $EtLp_1$:$IsoLp_2$ (30:70 wt%) resin ($CHCl_3$, 0.5 % w/w NEt_3) determined against polystyrene (PS) standards.

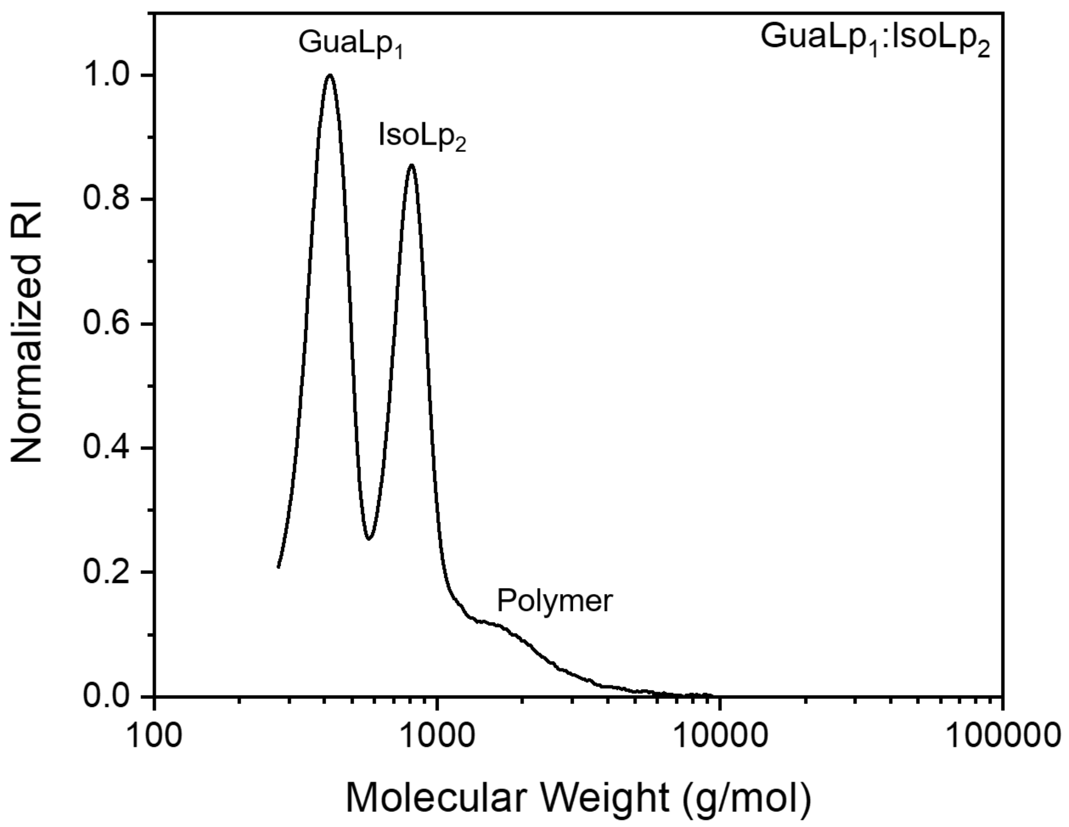

Fig. S85.
SEC chromatogram for 2D-photoset catalyzed depolymerization (phosphazene:thiophenol method) obtained from $GuaLp_1$:$IsoLp_2$ (30:70 wt%) resin ($CHCl_3$, 0.5 % w/w NEt_3) determined against polystyrene (PS) standards.

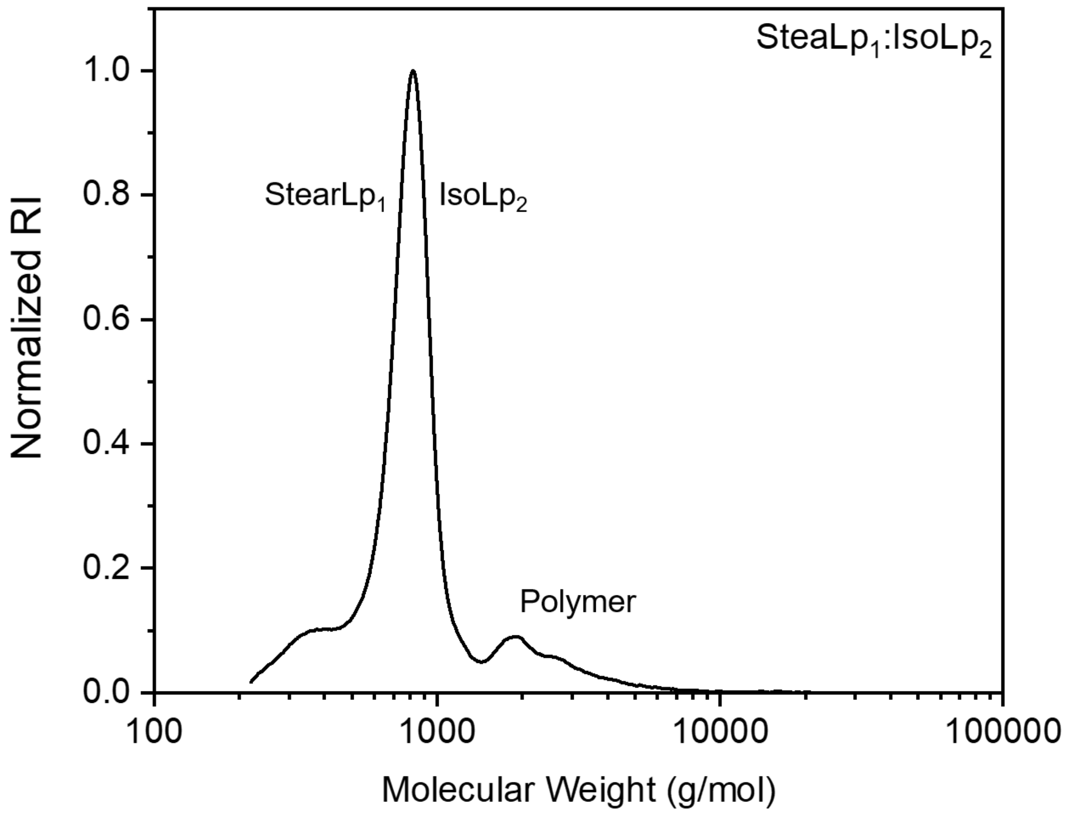

Fig. S86.
SEC chromatogram for 2D-photoset catalyzed depolymerization (phosphazene:thiophenol method) obtained from $SteaLp_1$:$IsoLp_2$ (30:70 wt%) resin ($CHCl_3$, 0.5 % w/w NEt_3) determined against polystyrene (PS) standards.

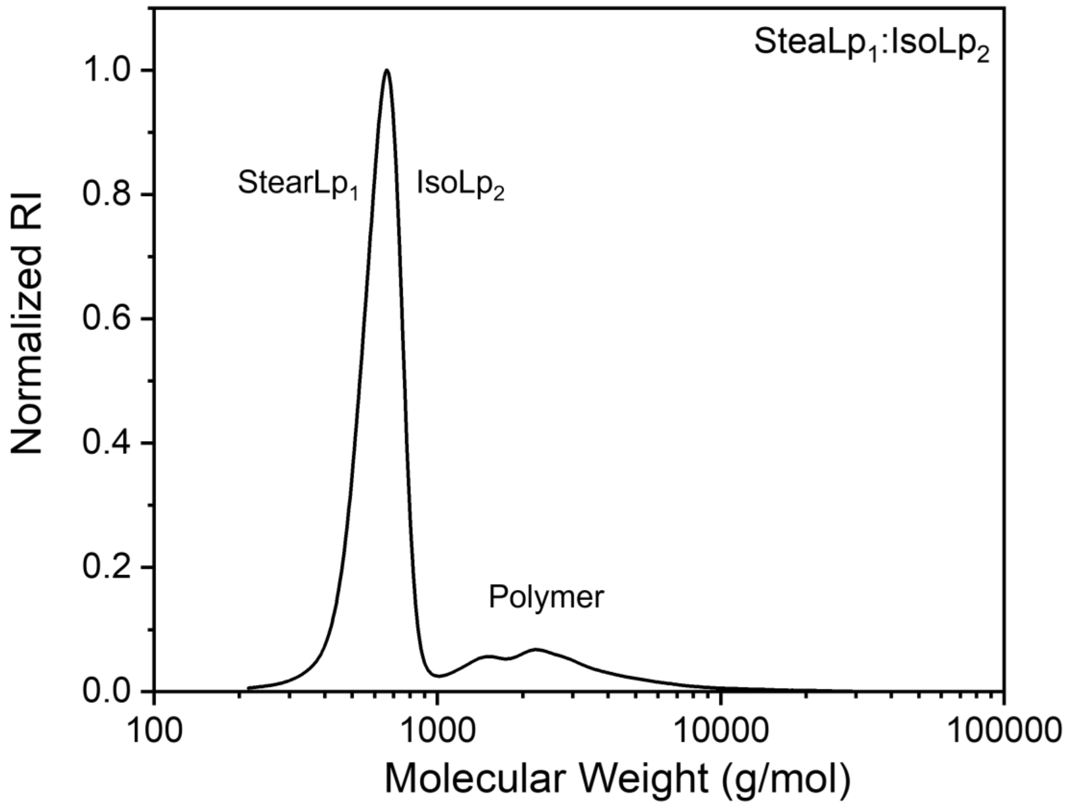

Fig. S87.
SEC chromatogram for 2D-photoset thermal depolymerization (DMF method) obtained from $SteaLp_1$:$IsoLp_2$ (30:70 wt%) resin ($CHCl_3$, 0.5 % w/w NEt_3) determined against polystyrene (PS) standards.

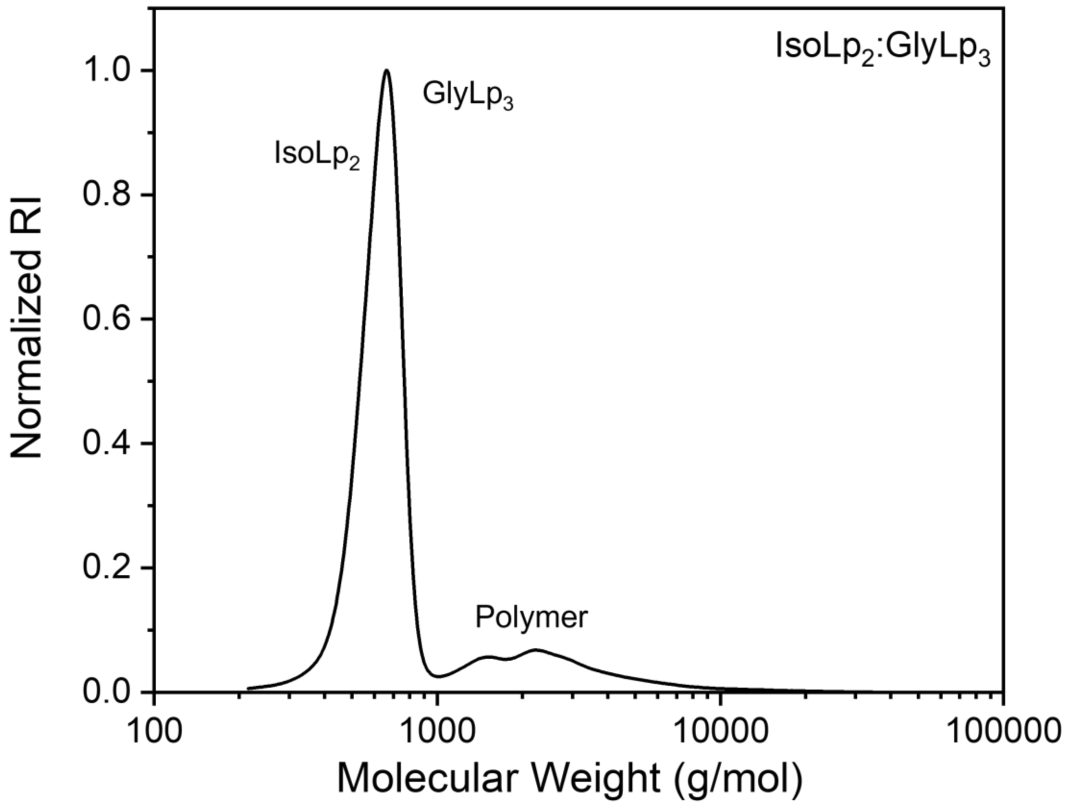

Fig. S88.
SEC chromatogram for 2D-photoset thermal depolymerization (DMF method) obtained from $IsoLp_2$:$GlyLp_3$ (70:30 wt%) resin ($CHCl_3$, 0.5 % w/w NEt_3) determined against polystyrene (PS) standards.

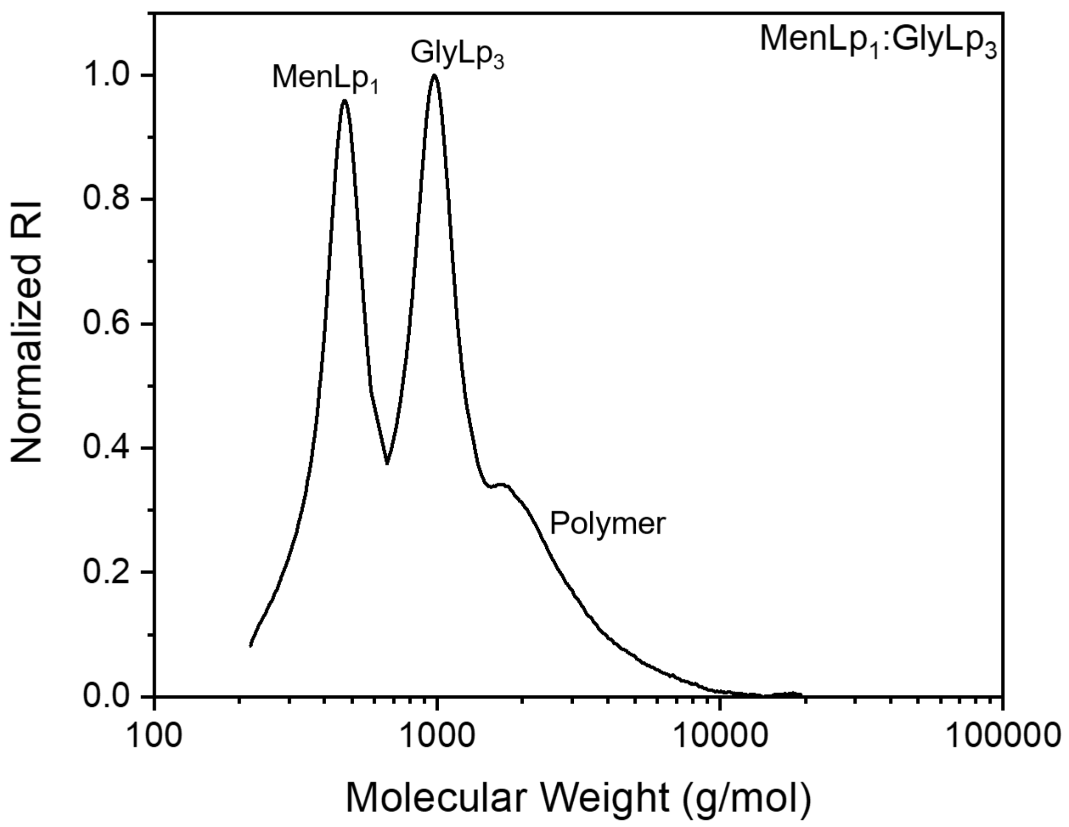

Fig. S89.
SEC chromatogram for 2D-photoset catalyzed depolymerization (phosphazene:thiophenol method) obtained from $MenLp_1$:$GlyLp_3$ (30:70 wt%) resin ($CHCl_3$, 0.5 % w/w NEt_3) determined against polystyrene (PS) standards.

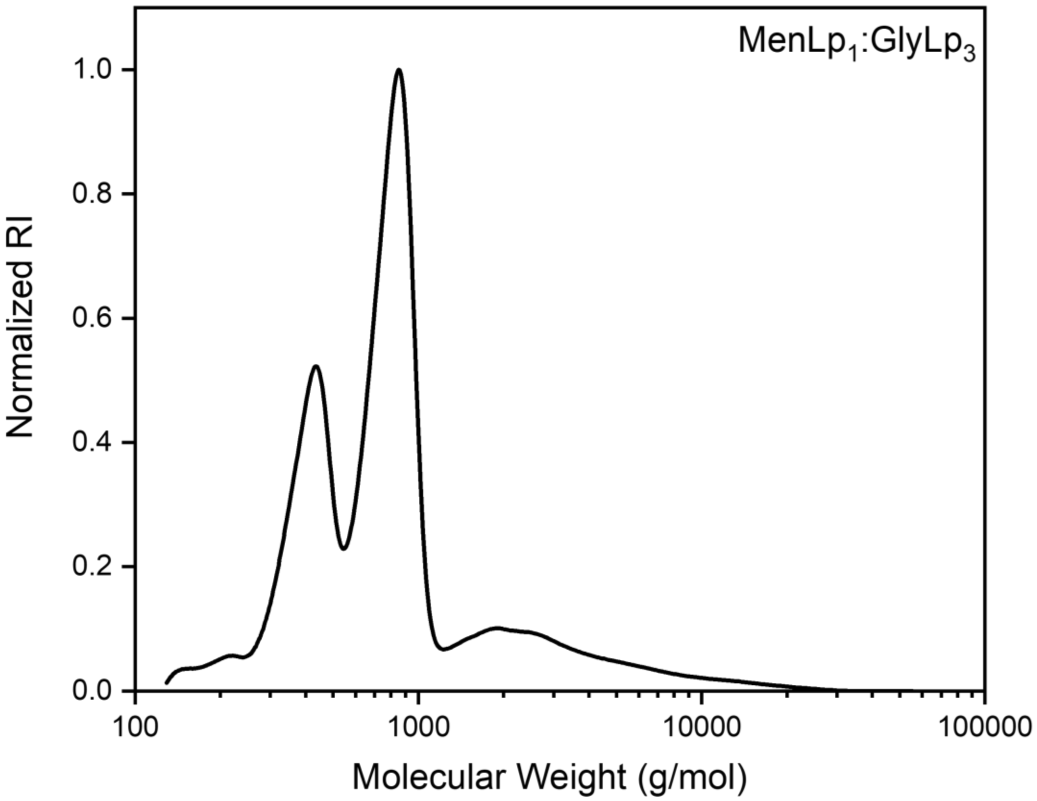

Fig. S90.
SEC chromatogram for 2D-photoset thermal depolymerization (DMF method) obtained from MenLp1:GlyLp3 (30:70 wt%) resin ($CHCl_3$, 0.5 % w/w NEt_3) determined against polystyrene (PS) standards.

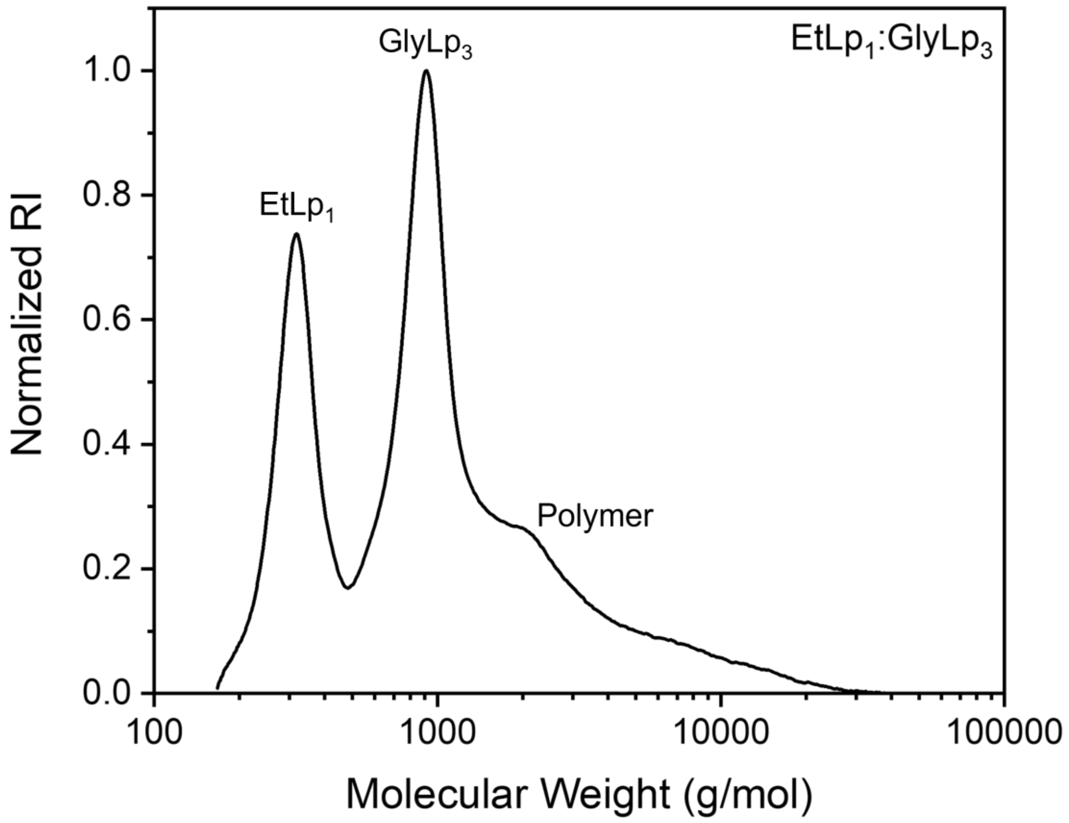

Fig. S91.
SEC chromatogram for 2D-photoset catalyzed depolymerization (phosphazene:thiophenol method) obtained from $EtLp_1$:$GlyLp_3$ (30:70 wt%) resin ($CHCl_3$, 0.5 % w/w NEt_3) determined against polystyrene (PS) standards.

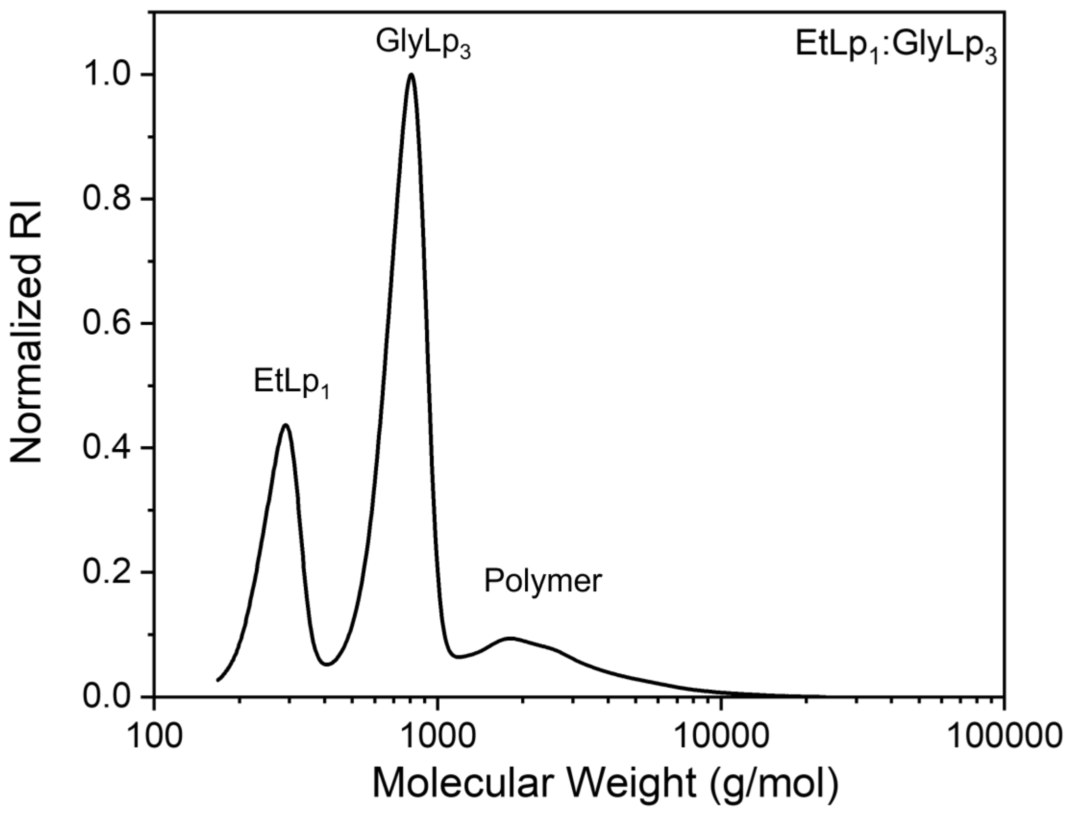

Fig. S92.
SEC chromatogram for 2D-photoset thermal depolymerization (DMF method) obtained from $EtLp_1$:$GlyLp_3$ (30:70 wt%) resin ($CHCl_3$, 0.5 % w/w NEt_3) determined against polystyrene (PS) standards.

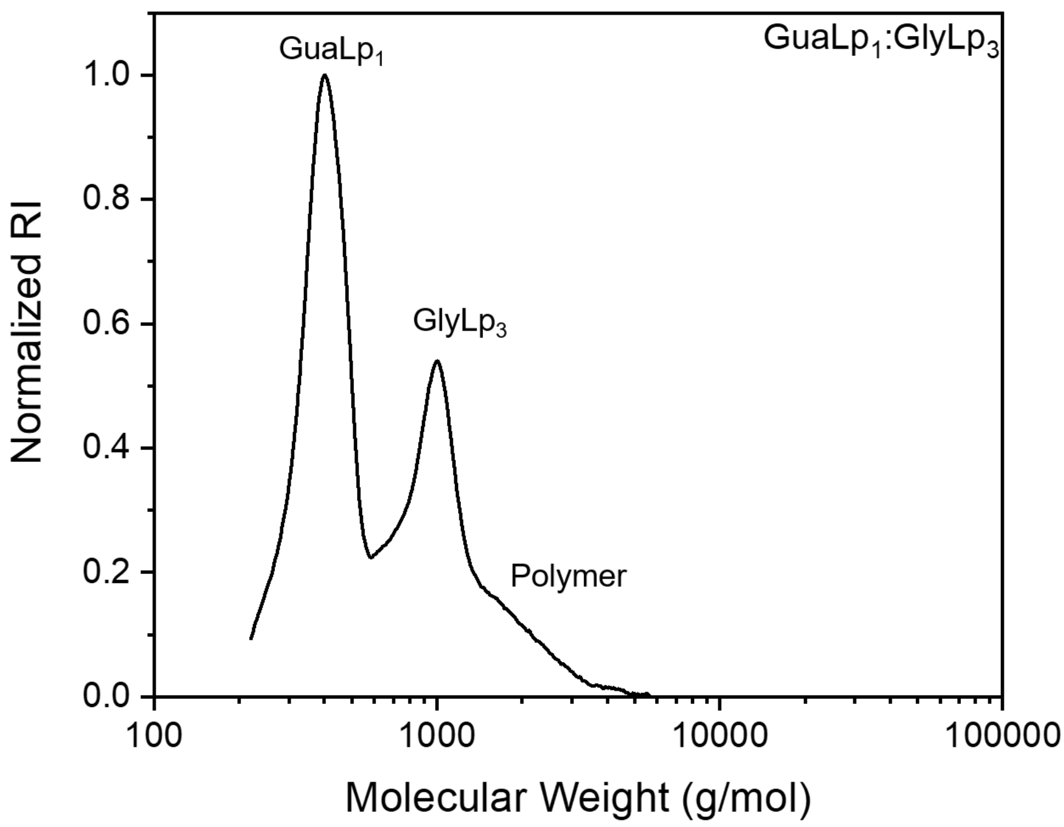

Fig. S93.
SEC chromatogram for 2D-photoset catalyzed depolymerization (phosphazene:thiophenol method) obtained from GuaLp1:GlyLp3 (30:70 wt%) resin ($CHCl_3$, 0.5 % w/w NEt_3) determined against polystyrene (PS) standards.

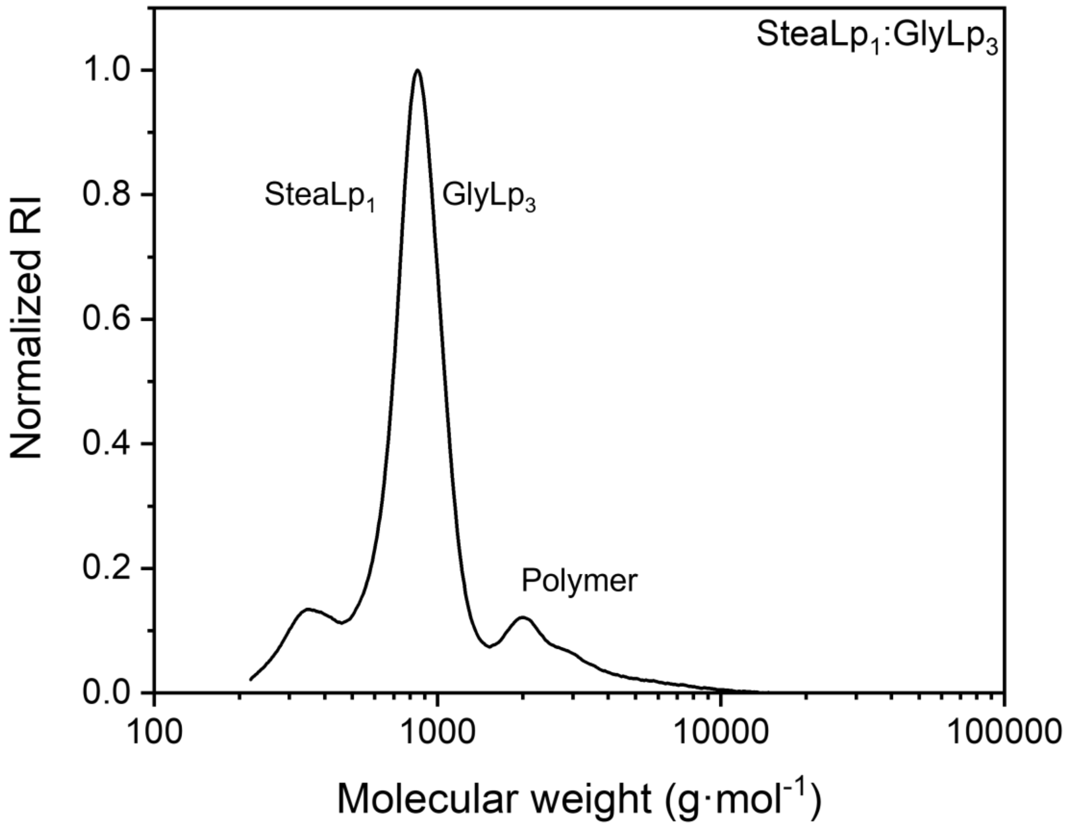

Fig. S94.
SEC chromatogram for 2D-photoset catalyzed depolymerization (phosphazene:thiophenol method) obtained from SteaLp1:GlyLp3 (30:70 wt%) resin ($CHCl_3$, 0.5 % w/w NEt_3) determined against polystyrene (PS) standards.

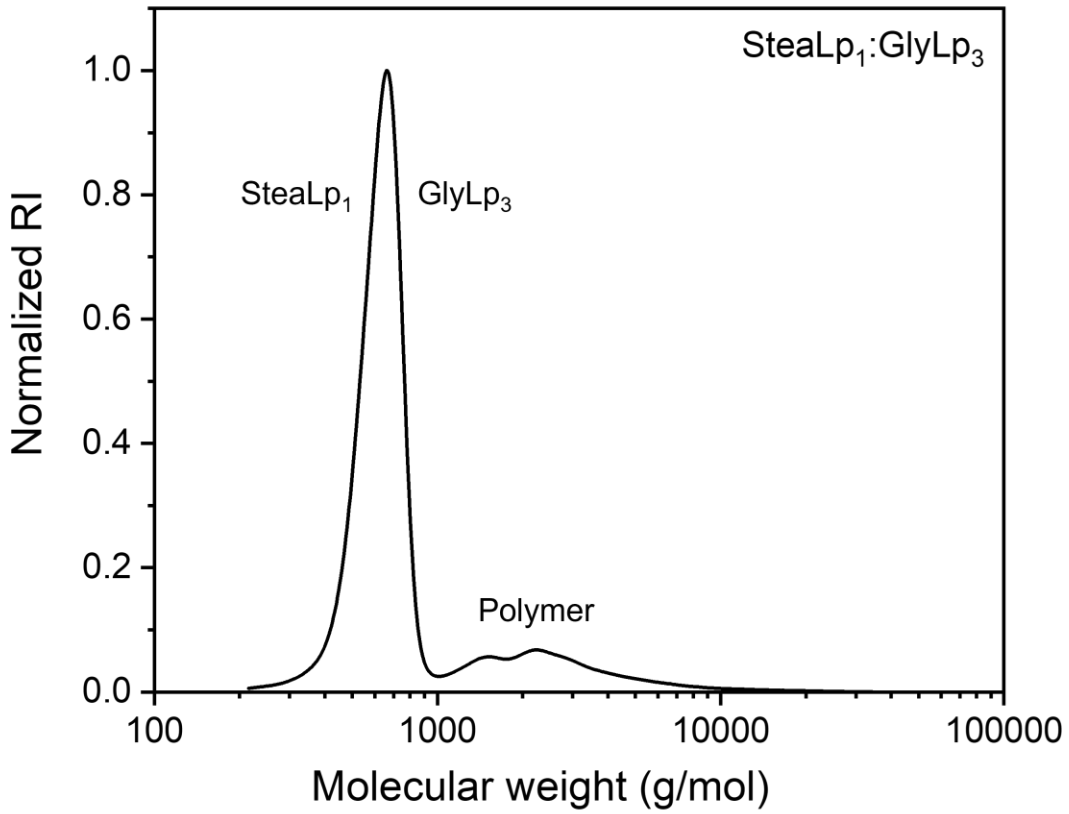

Fig. S95.
SEC chromatogram for 2D-photoset thermal depolymerization (DMF method) obtained from SteaLp1:GlyLp3 (30:70 wt%) resin ($CHCl_3$, 0.5 % w/w NEt_3) determined against polystyrene (PS) standards.

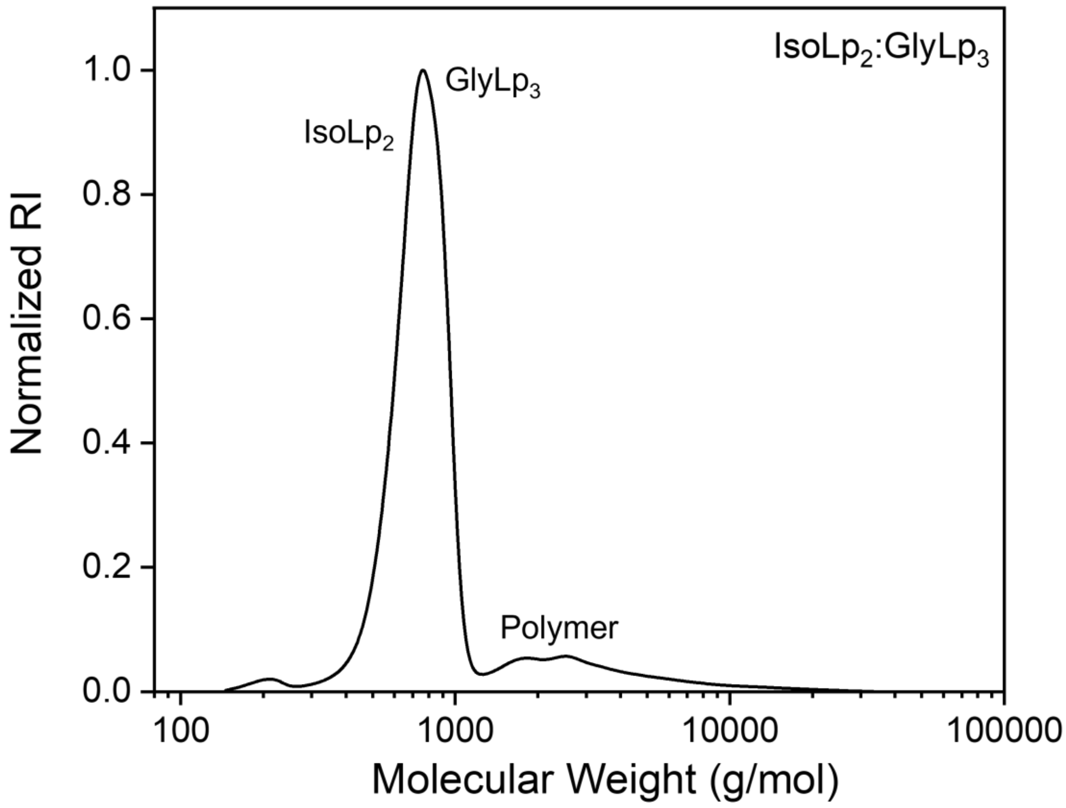

Fig. S96.
SEC chromatogram for 2D-photoset thermal depolymerization (DMF method) obtained from $IsoLp_2$:$GlyLp_3$ (30:80 wt%) resin ($CHCl_3$, 0.5 % w/w NEt_3) determined against polystyrene (PS) standards.

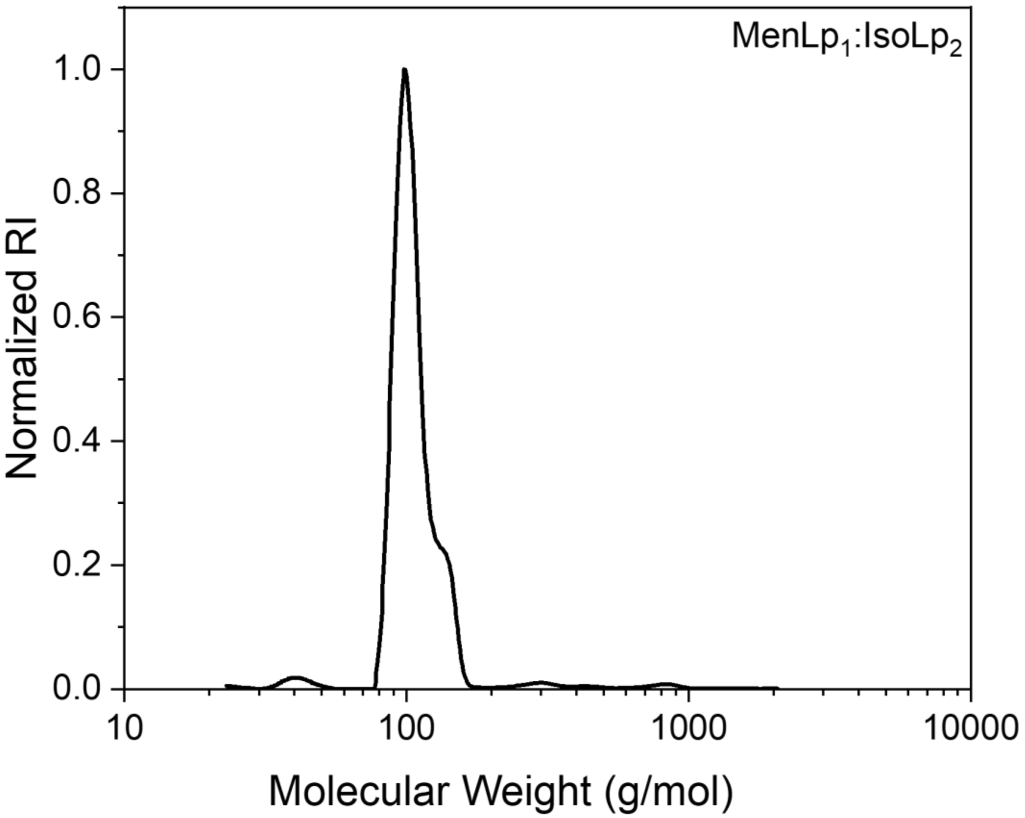

Fig. S97.
SEC chromatogram of recycled 3D-printed MenLp1:IsoLp2 (30:70 wt.%). Recycled using hydrolytic depolymerization method (Table S4).

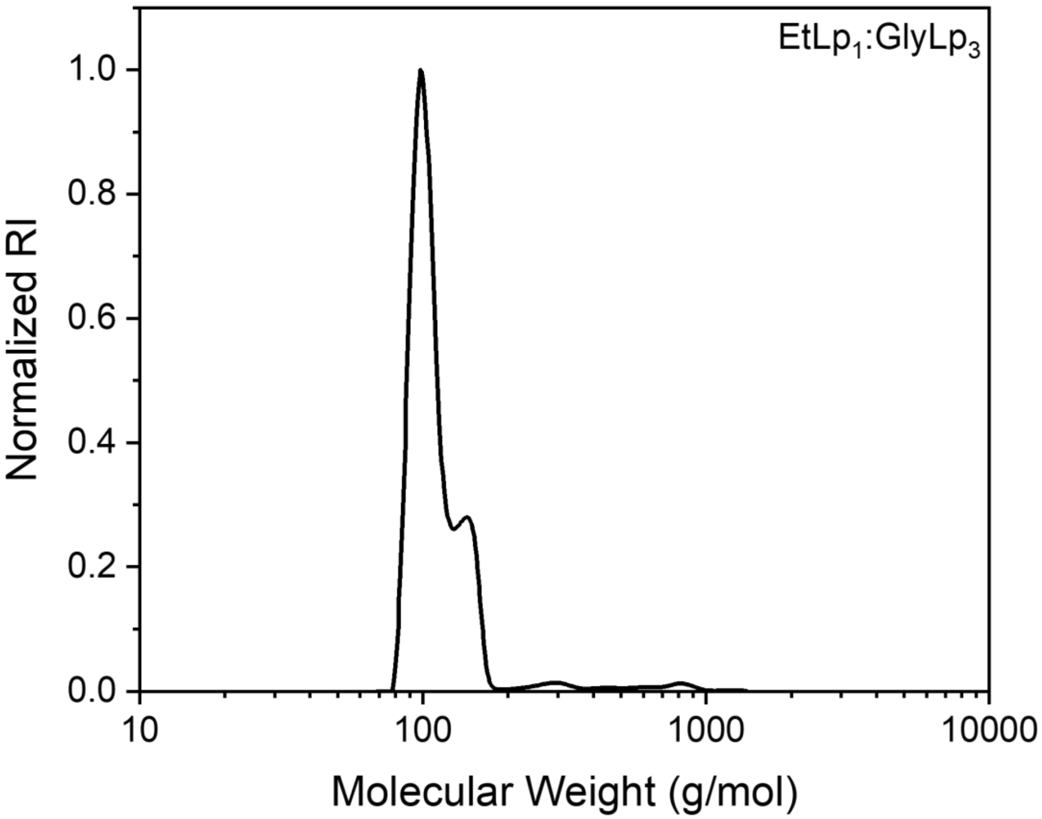

Fig. S98.
SEC chromatogram of recycled 3D-printed $EtLp_1$:$GlyLp_3$ (31:69 wt.%). Recycled using hydrolytic depolymerization method (Table S4).

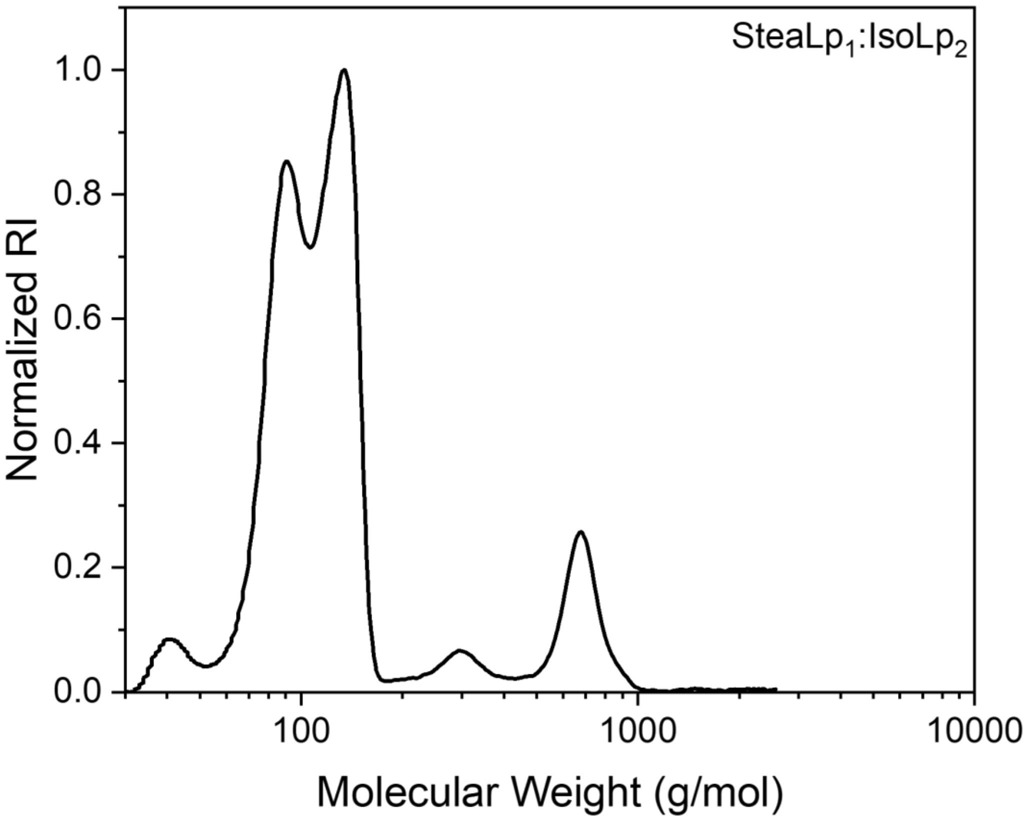

Fig. S99.
SEC chromatogram of recycled 3D-printed $SteaLp_1$:$IsoLp_2$ (30:70 wt.%). Recycled using hydrolytic depolymerization method (Table S4).

TGA thermograms

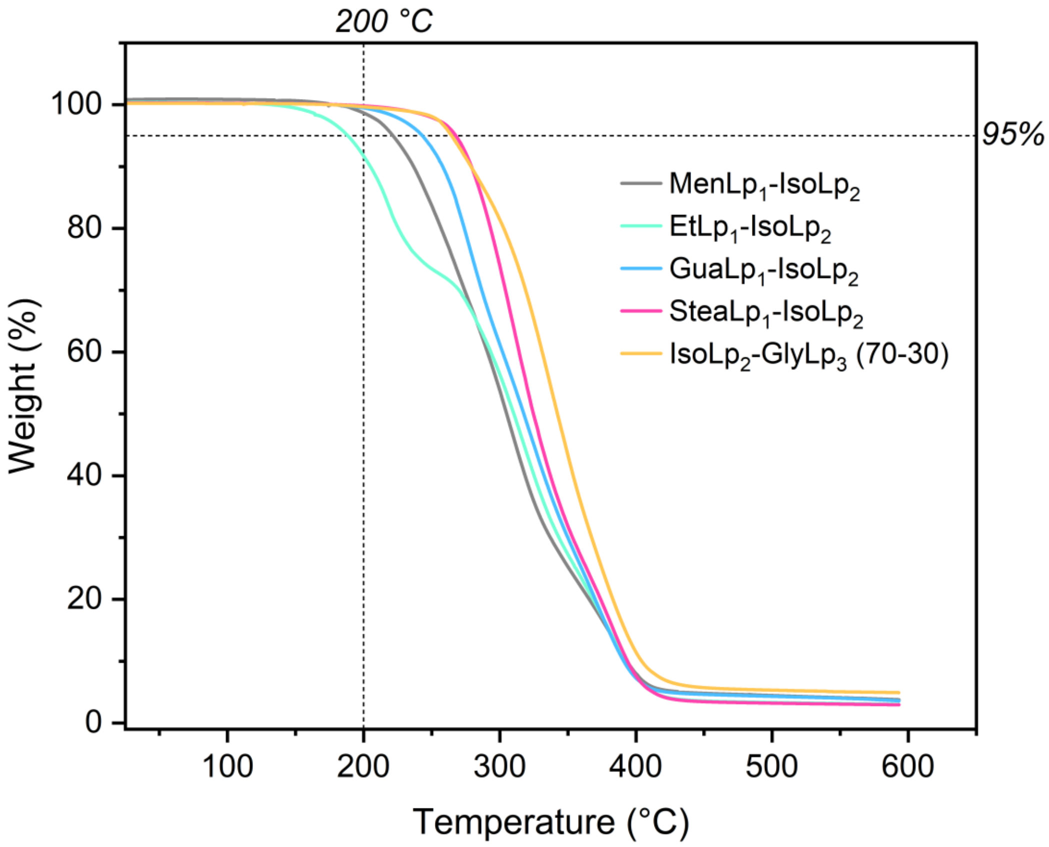

Fig. S100.
TGA thermograms of R-Lp_1:$IsoLp_2$ (~30:70 wt%) 2D-photoset post-cured samples. $T_{d, 95\%}$ is indicated by horizontal line, vertical line indicates 200 °C threshold.

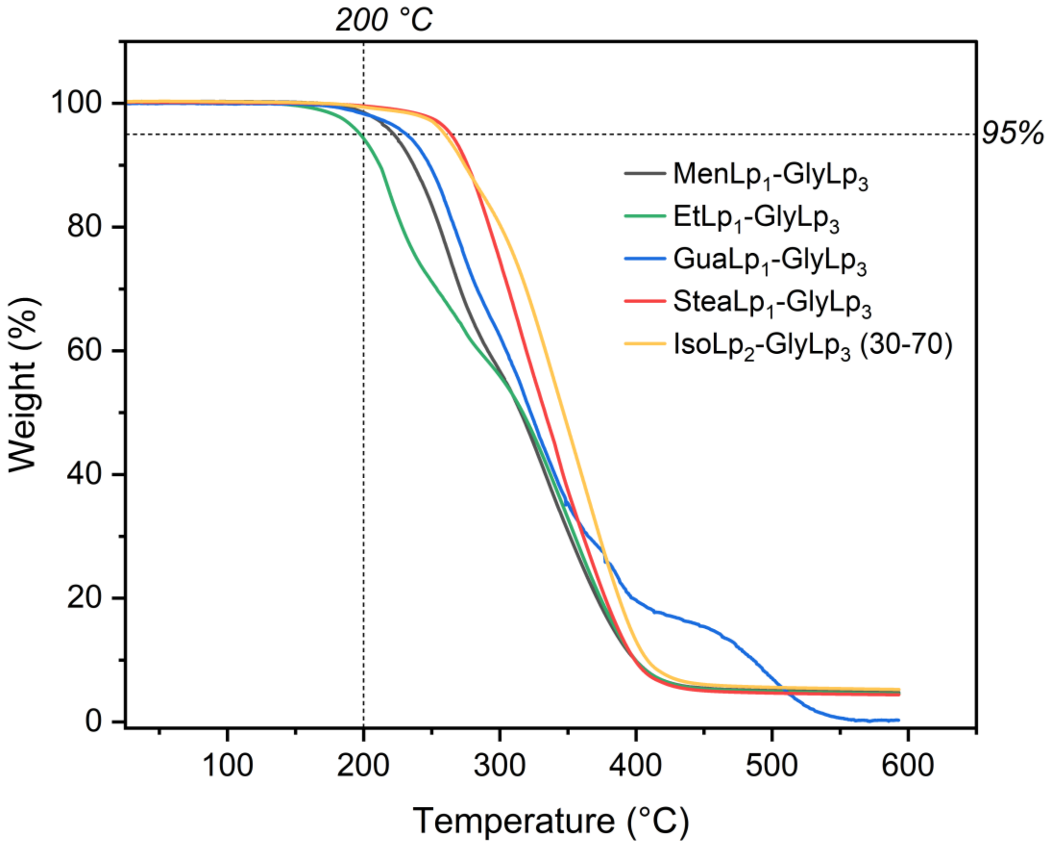

Fig. S101.
TGA thermograms of R-Lp_1:$GlyLp_3$ (~30:70 wt%) 2D-photoset post-cured samples. $T_{d,\,95\%}$ is indicated by horizontal line, vertical line indicates 200 °C threshold.

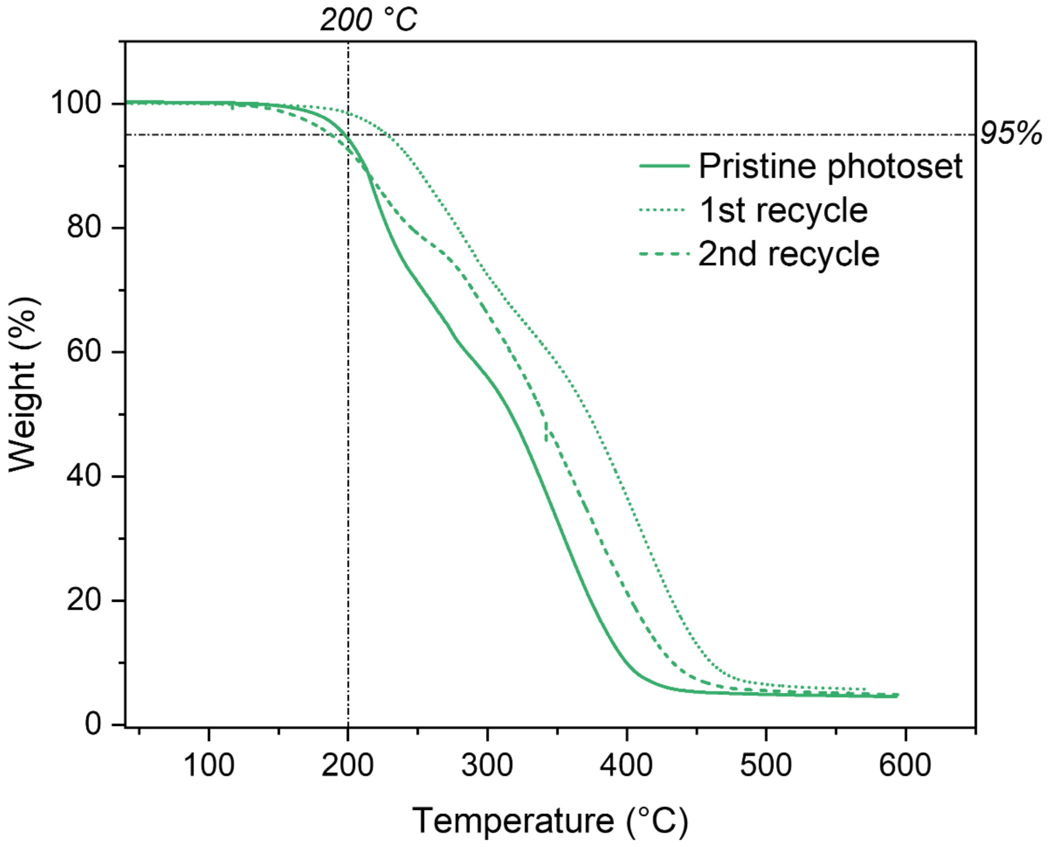

Fig. S102
TGA thermograms of 2D photosets from recycled $EtLp_1:GlyLp_3$ (30:70 wt%) resins. $T_{d,\,95\%}$ is indicated by horizontal line, vertical line indicates 200 °C threshold.

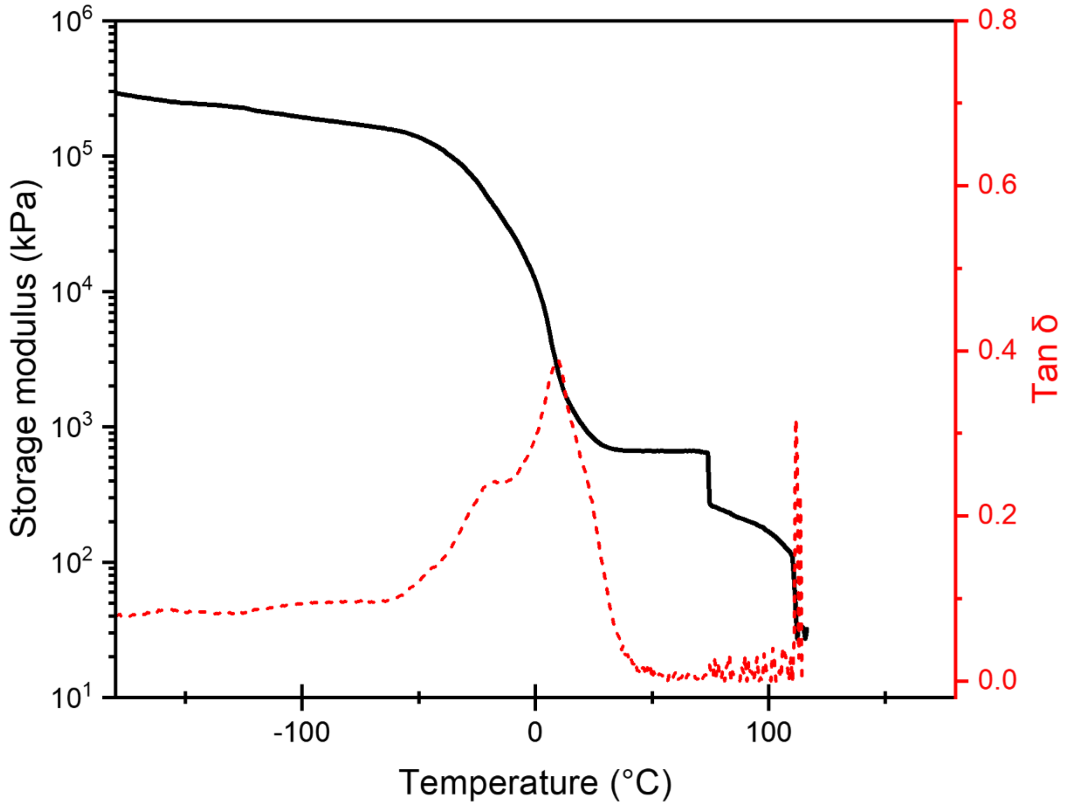

Fig. S103.
DMA thermogram plotting storage modulus, tan δ *vs* temperature for MenLp1:IsoLp2 (28:72 wt.%) 2D photoset.

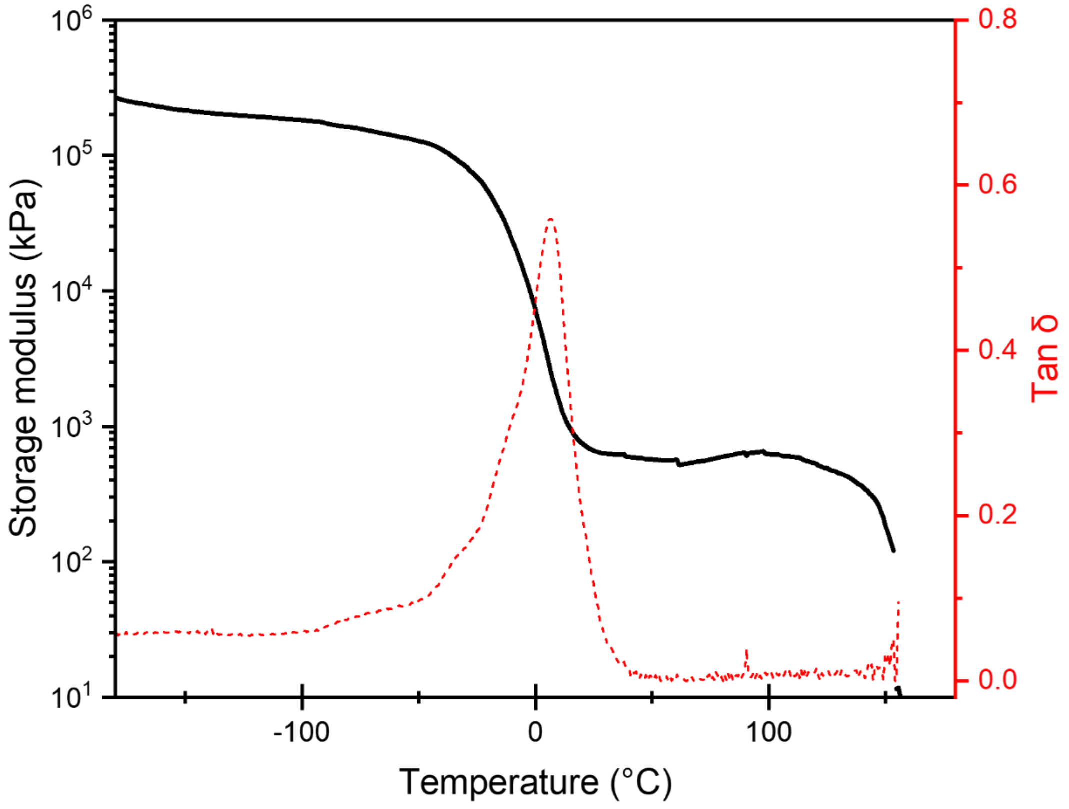

Fig. S104.
DMA thermogram plotting storage modulus, tan δ *vs* temperature for $EtLp_1$:$IsoLp_2$ (27:73 wt.%) 2D photoset.

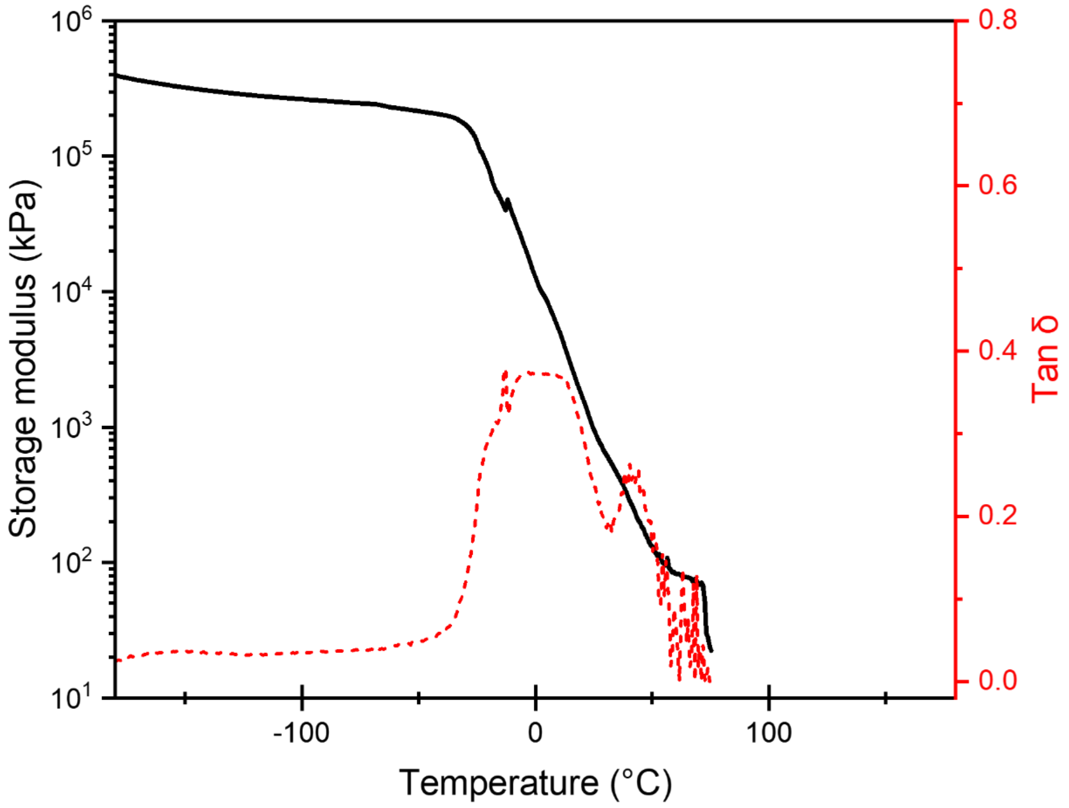

Fig. S105.
DMA thermogram plotting storage modulus, tan δ *vs* temperature for $GuaLp_1$:$IsoLp_2$ (34:66 wt.%) 2D photoset.

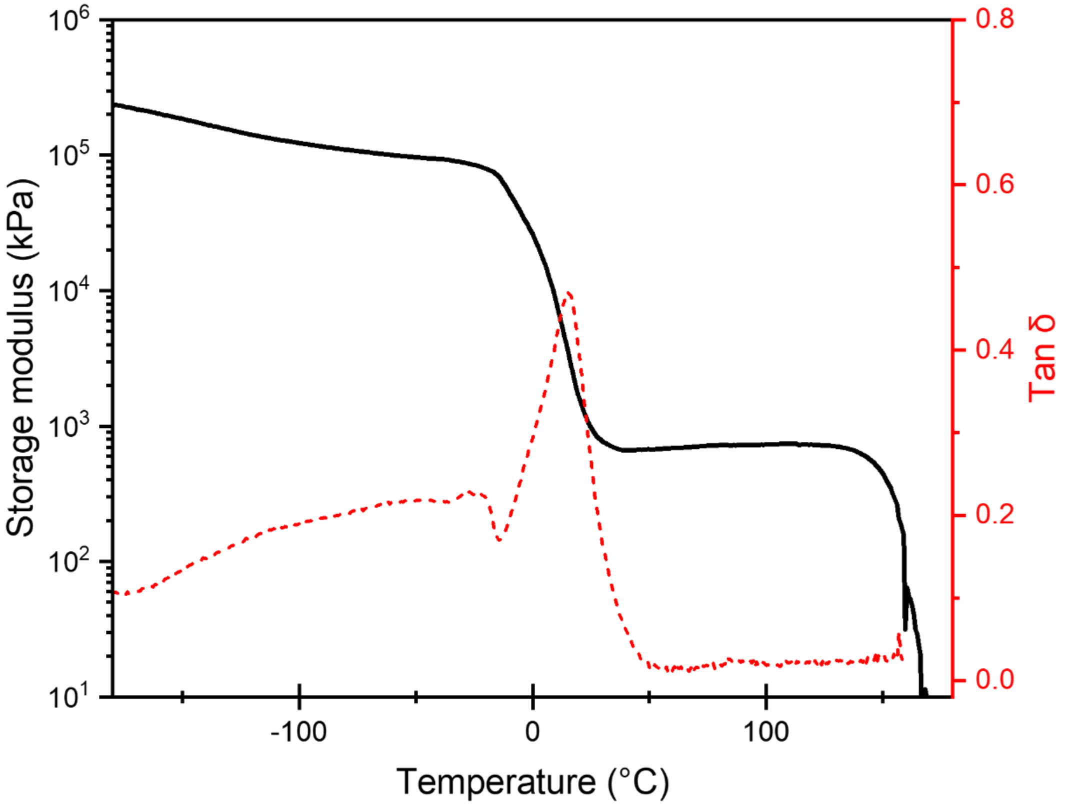

Fig. S106.
DMA thermogram plotting storage modulus, tan δ *vs* temperature for SteaLp1:IsoLp2 (30:70 wt.%) 2D photoset.

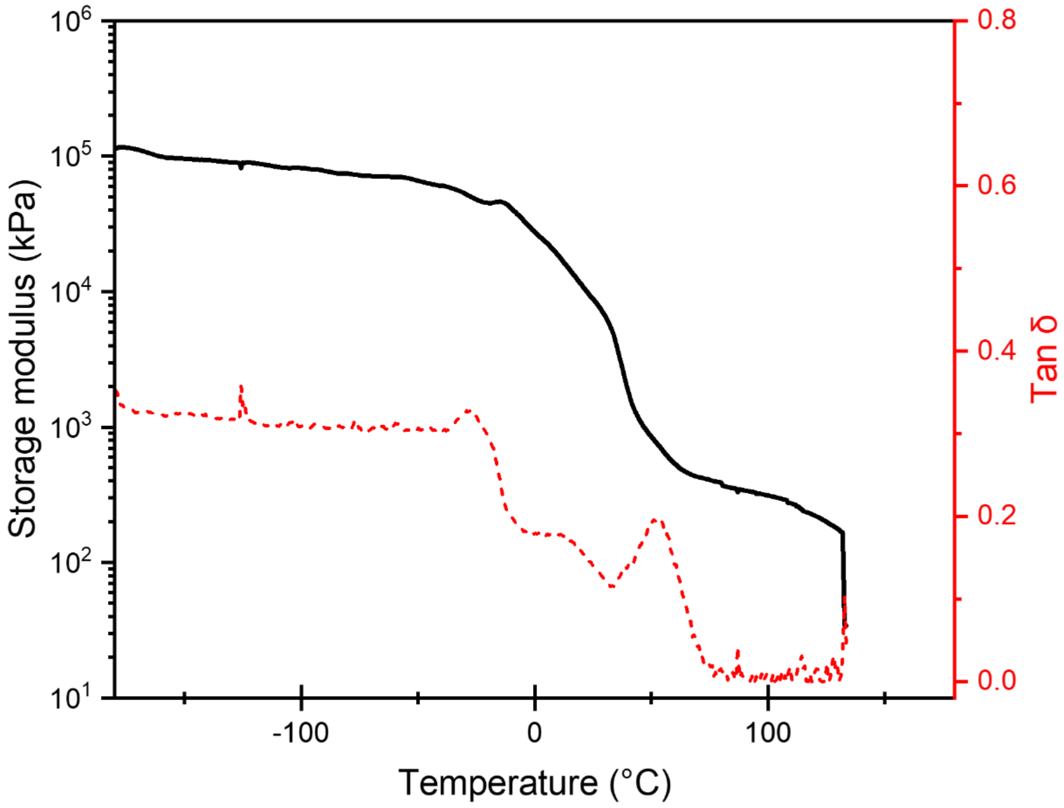

Fig. S107.
DMA thermogram plotting storage modulus, tan δ *vs* temperature for IsoLp2:GlyLp3: (70:30 wt.%) 2D photoset.

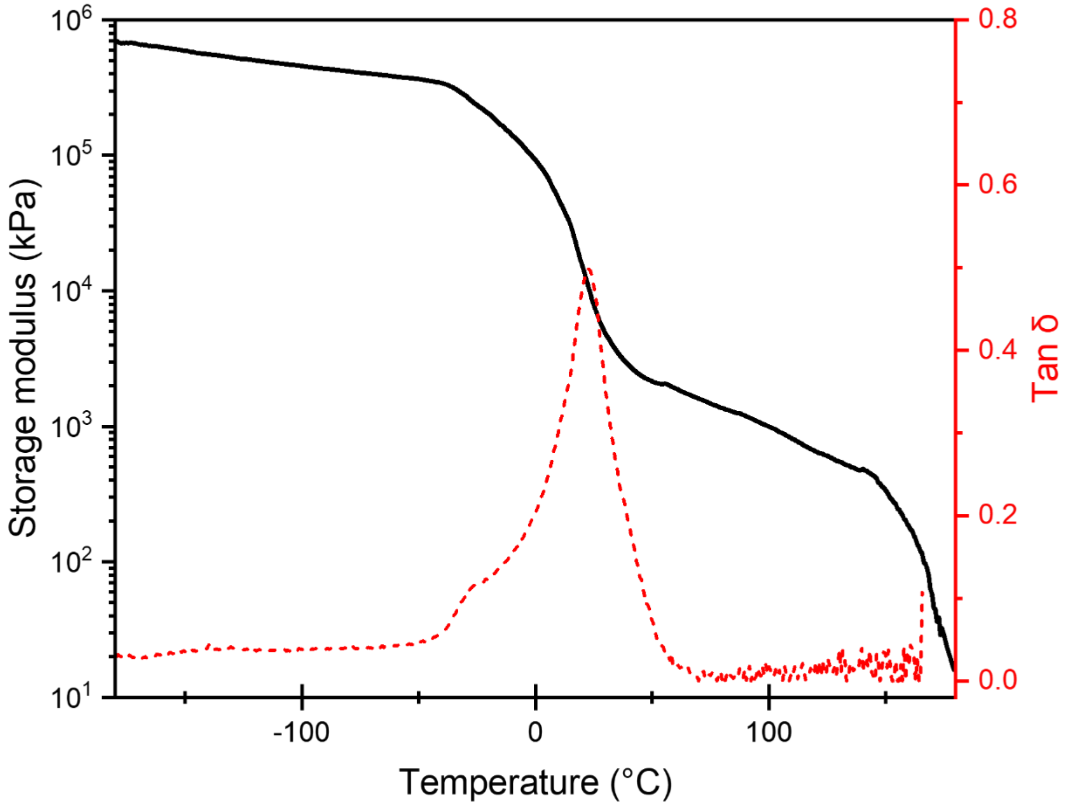

Fig. S108.
DMA thermogram plotting storage modulus, tan δ *vs* temperature for MenLp1:GlyLp3: (33:67 wt.%) 2D photoset.

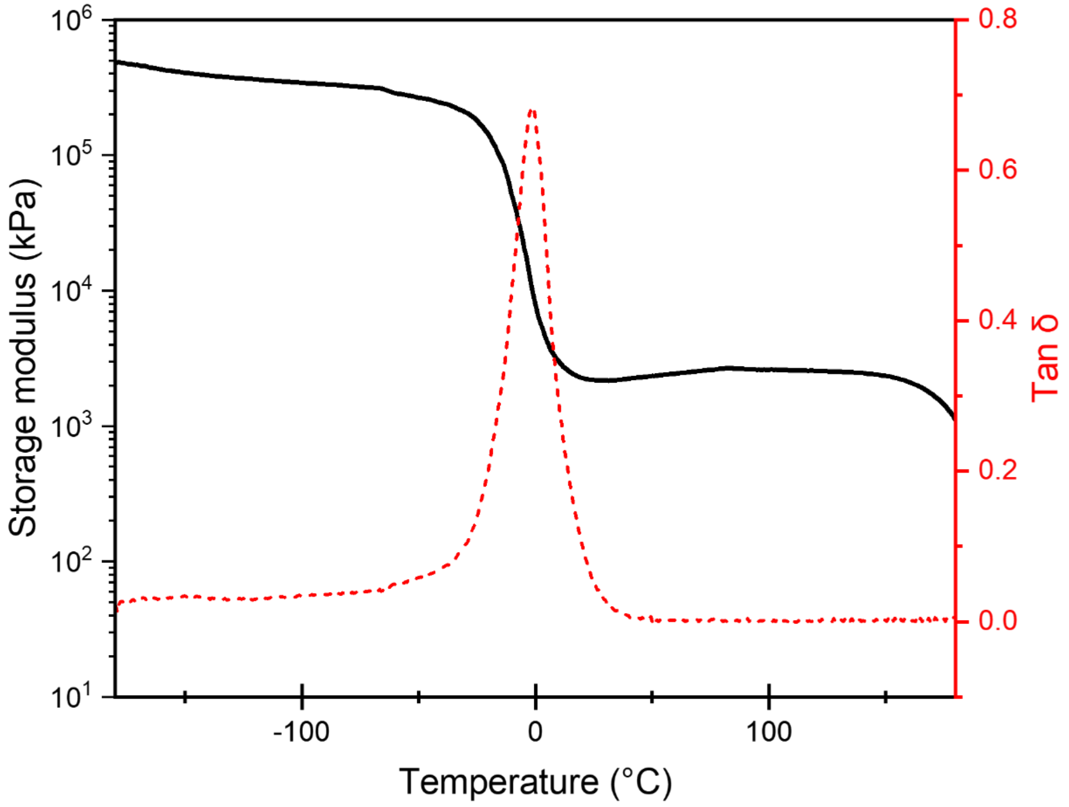

Fig. S109.
DMA thermogram plotting storage modulus, tan δ *vs* temperature for $EtLp_1$:$GlyLp_3$: (34:66 wt.%) 2D photoset.

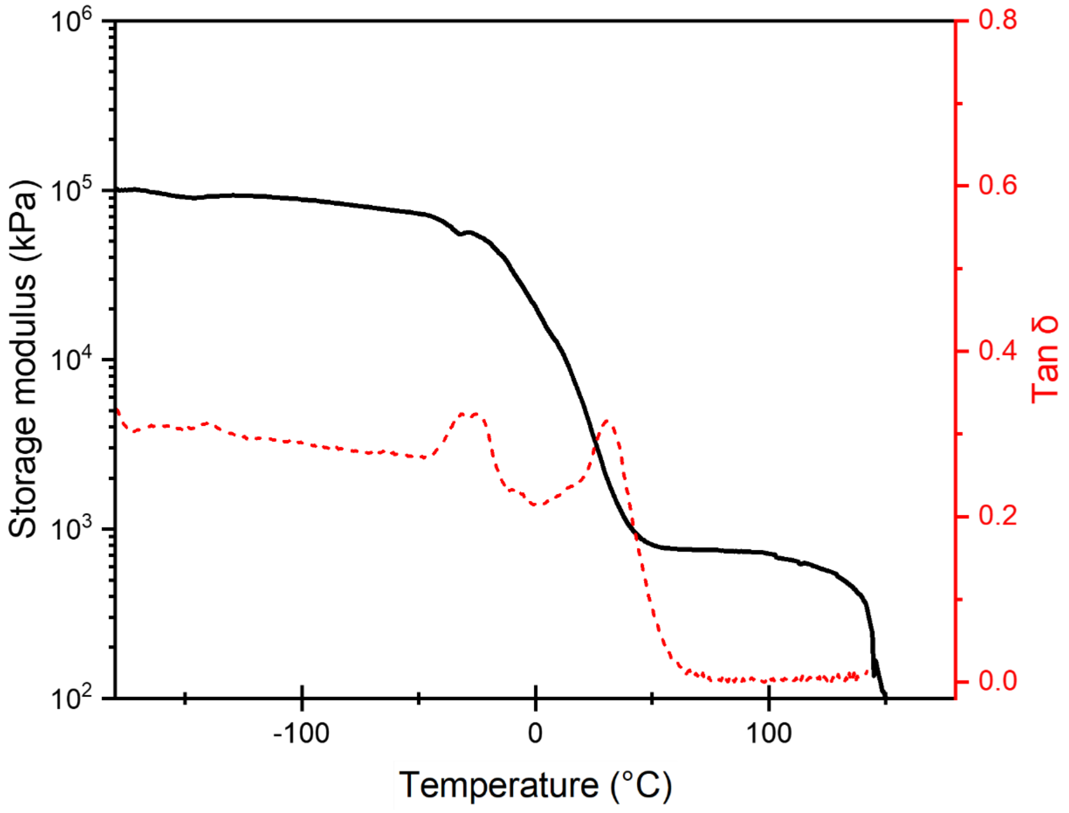

Fig. S110.
DMA thermogram plotting storage modulus, tan δ *vs* temperature for GuaLp1:GlyLp3: (31:69 wt.%) 2D photoset.

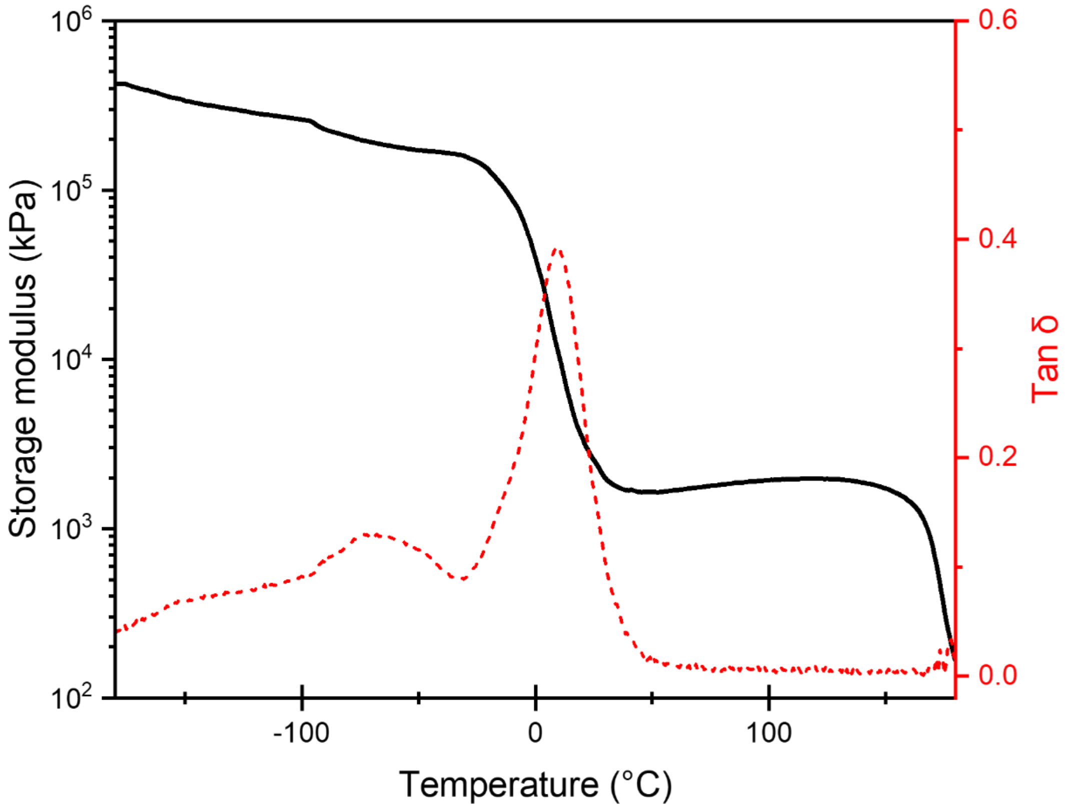

Fig. S111.
DMA thermogram plotting storage modulus, tan δ *vs* temperature for SteaLp1:GlyLp3: (32:68 wt.%) 2D photoset.

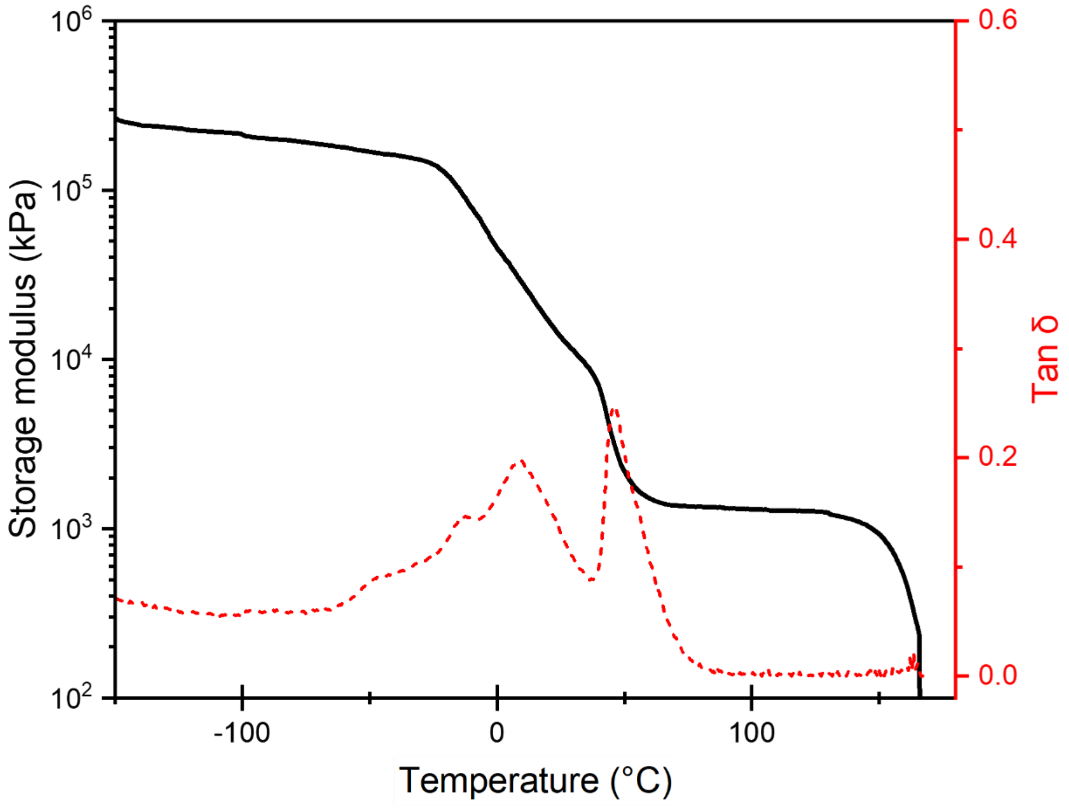

Fig. S112.
DMA thermogram plotting storage modulus, tan δ *vs* temperature for IsoLp2:GlyLp3: (28:72 wt.%) 2D photoset.

DSC thermograms

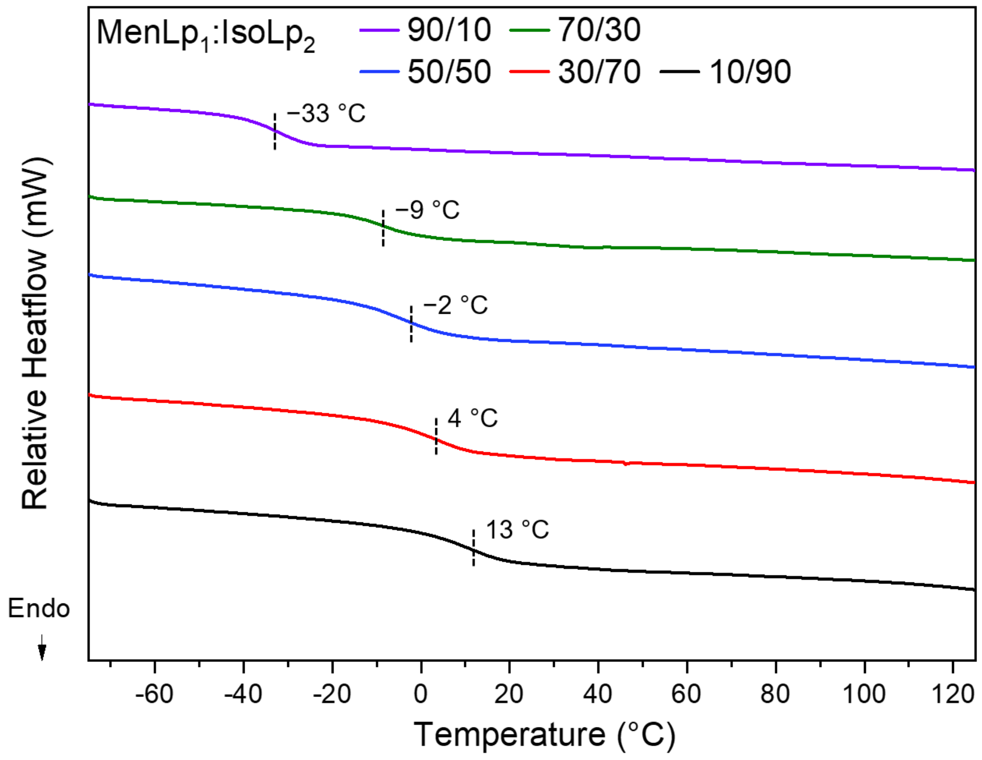

Fig. S113.
DSC thermograms of 2nd heating cycle for MenLp$_1$:IsoLp$_2$ (90:10 to 10:90 wt%) as-synthesized samples. Approximate position of T_g is indicated by vertical hashmark.

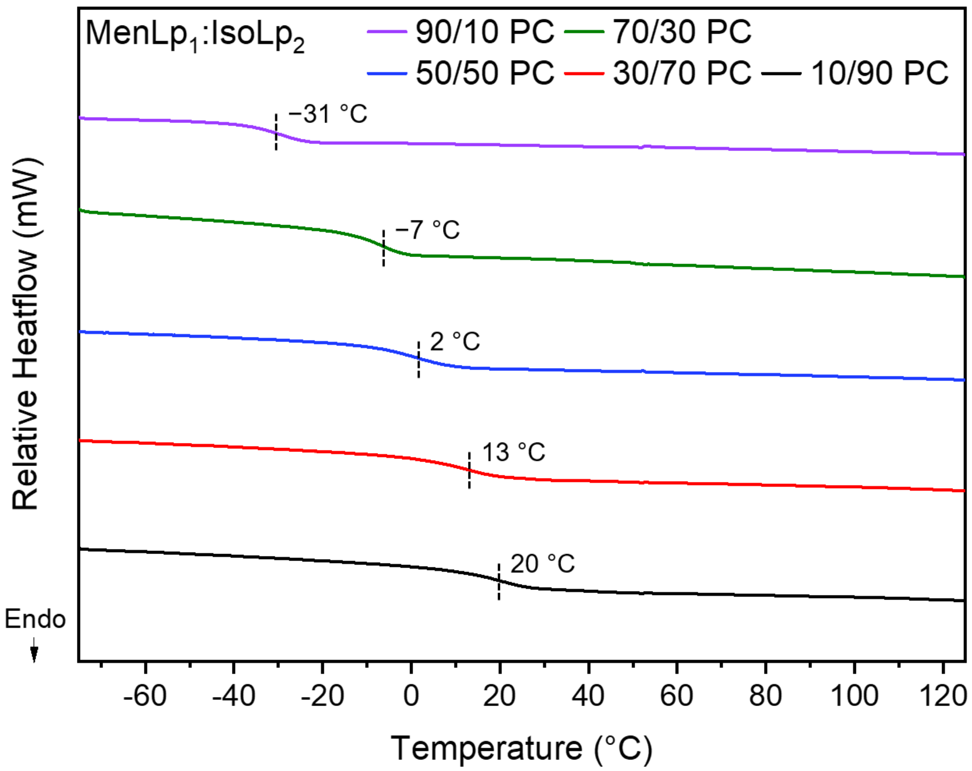

Fig. S114.
DSC thermograms of 2nd heating cycle for $MenLp_1$:$IsoLp_2$ (90:10 to 10:90 wt%) post-cured samples. Approximate position of T_g is indicated by vertical hashmark.

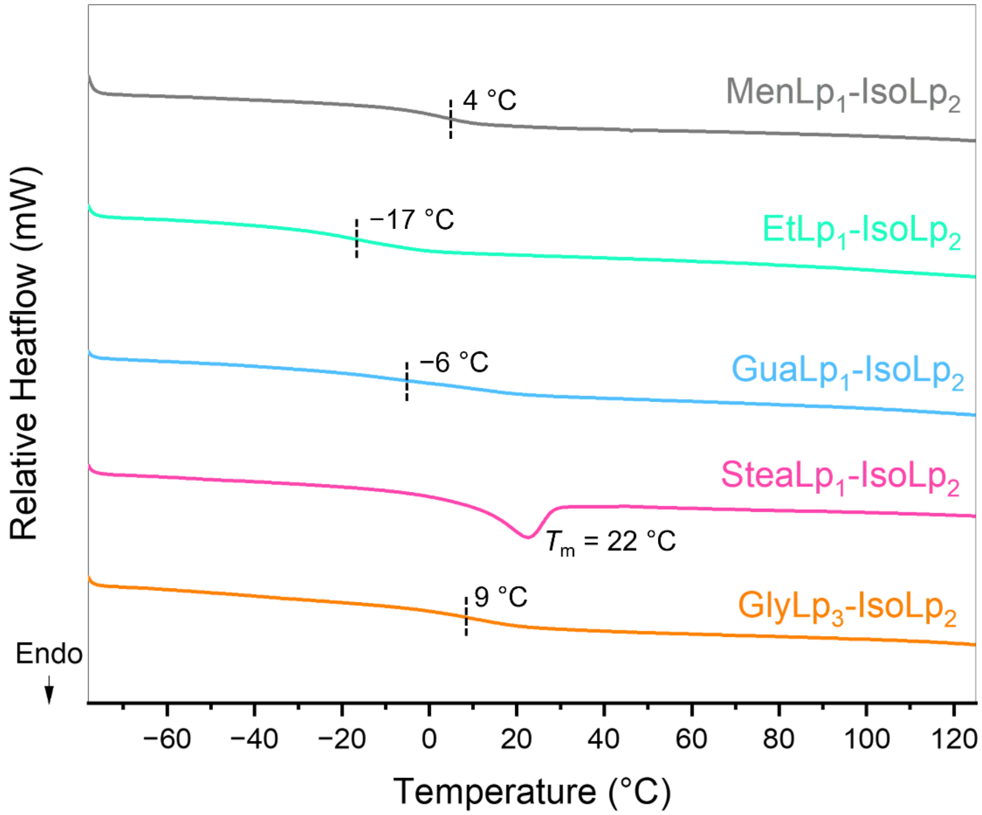

Fig. S115.
DSC thermograms of 2nd heating cycle for R-Lp_1:$IsoLp_2$ (30:70 wt%) as-synthesized samples. Approximate position of T_g is indicated by vertical hashmark.

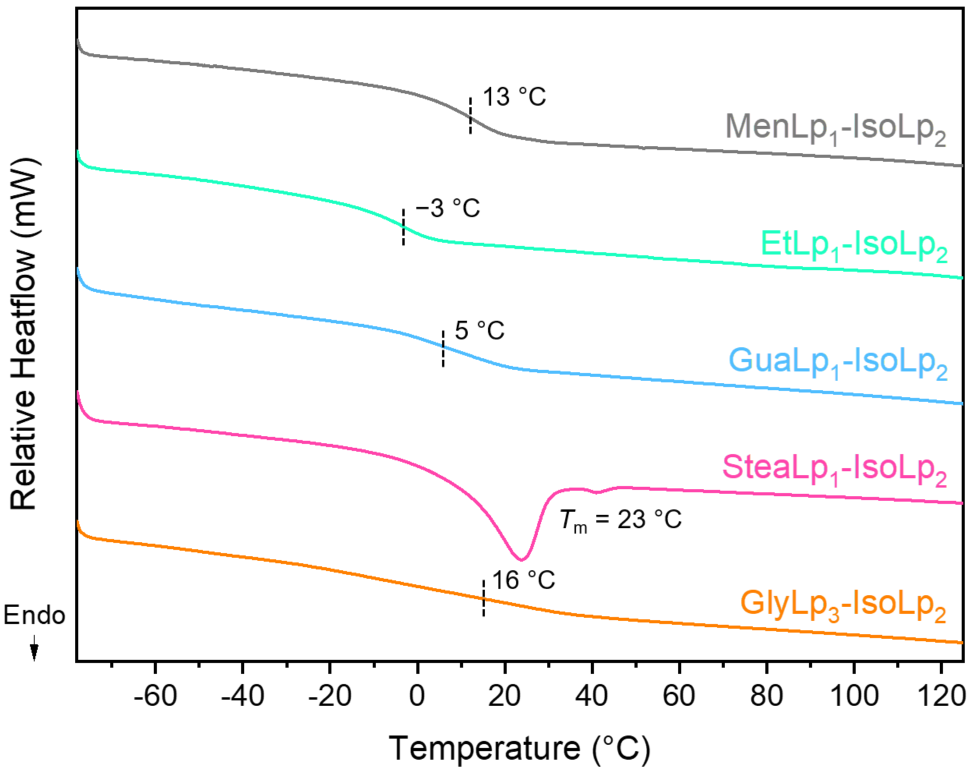

Fig. S116.
DSC thermograms of 2nd heating cycle for R-Lp_1:IsoLp_2 (30:70 wt%) post-cured samples. Approximate position of T_g is indicated by vertical hashmark.

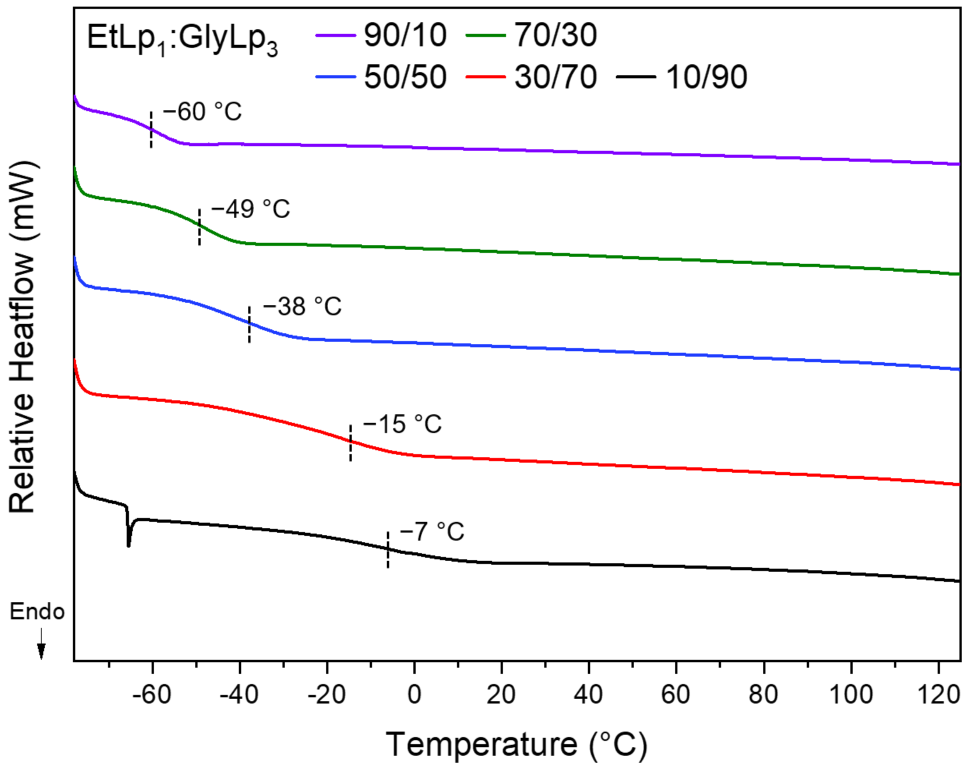

Fig. S117.
DSC thermograms of 2nd heating cycle for $EtLp_1$:$GlyLp_3$ (90:10 to 10:90 wt%) as-synthesized samples. Approximate position of T_g is indicated by vertical hashmark.

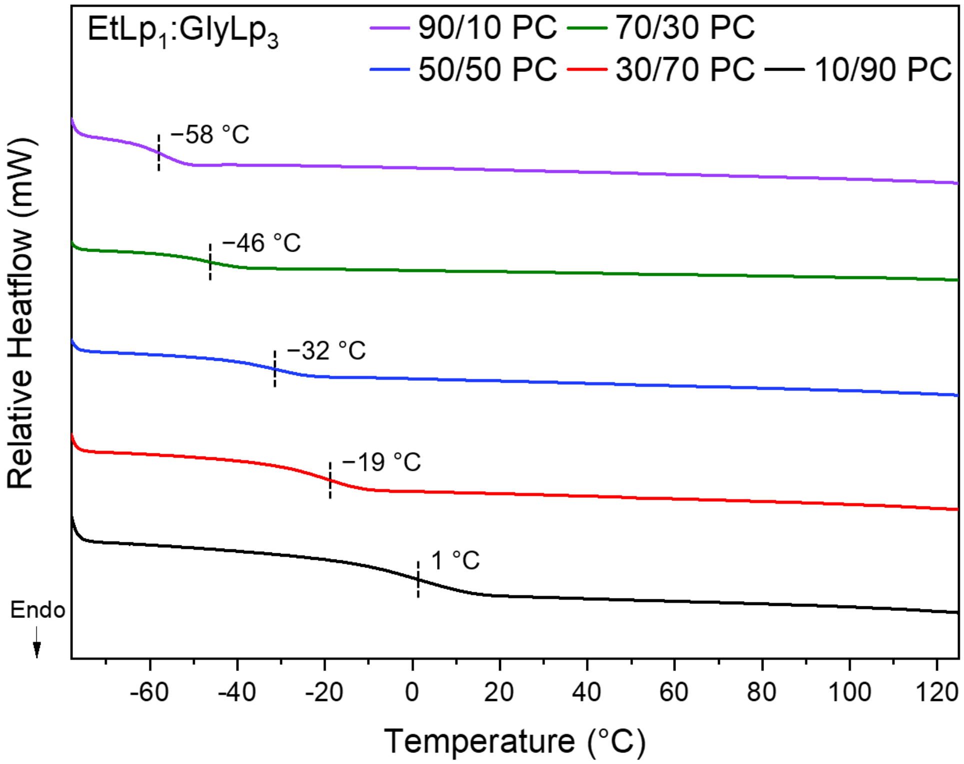

Fig. S118.
DSC thermograms of 2nd heating cycle for $EtLp_1$:$GlyLp_3$ (90:10 to 10:90 wt%) post-cured samples. Approximate position of T_g is indicated by vertical hashmark.

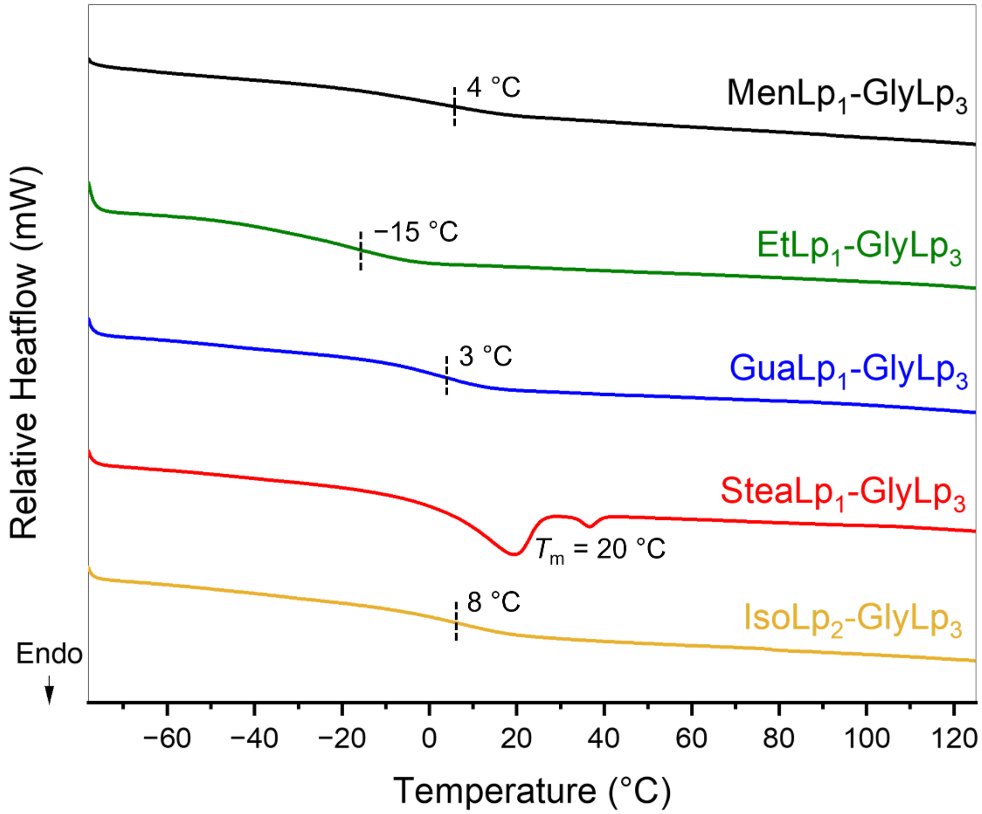

Fig. S119.
DSC thermograms of 2nd heating cycle for R-Lp_1:$GlyLp_3$ (30:70 wt%) as-synthesized samples. Approximate position of T_g is indicated by vertical hashmark.

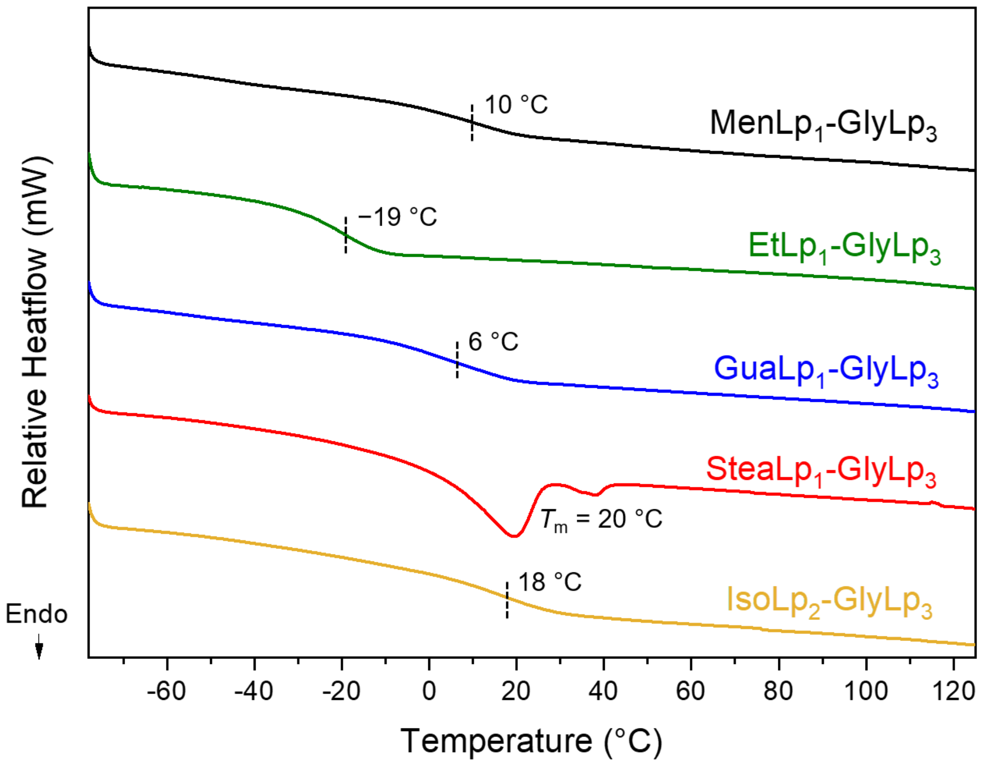

Fig. S120.
DSC thermograms of 2nd heating cycle for R-Lp_1:$GlyLp_3$ (30:70 wt%) post-cured samples. Approximate position of T_g is indicated by vertical hashmark.

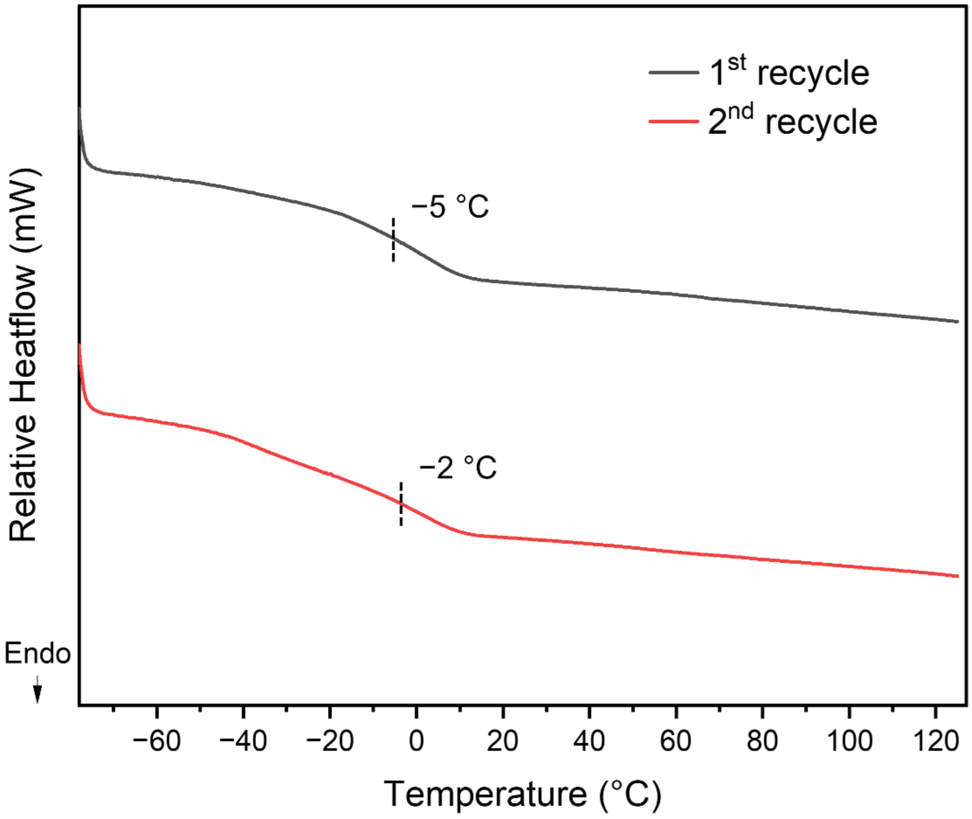

Fig. S121.
DSC thermograms of 2nd heating cycle for 2D photoset post-cured samples obtained from recycled $EtLp_1$:$GlyLp_3$ (30:70 wt%) resins. Approximate position of T_g is indicated by vertical hashmark.

Mechanical (tensile) properties

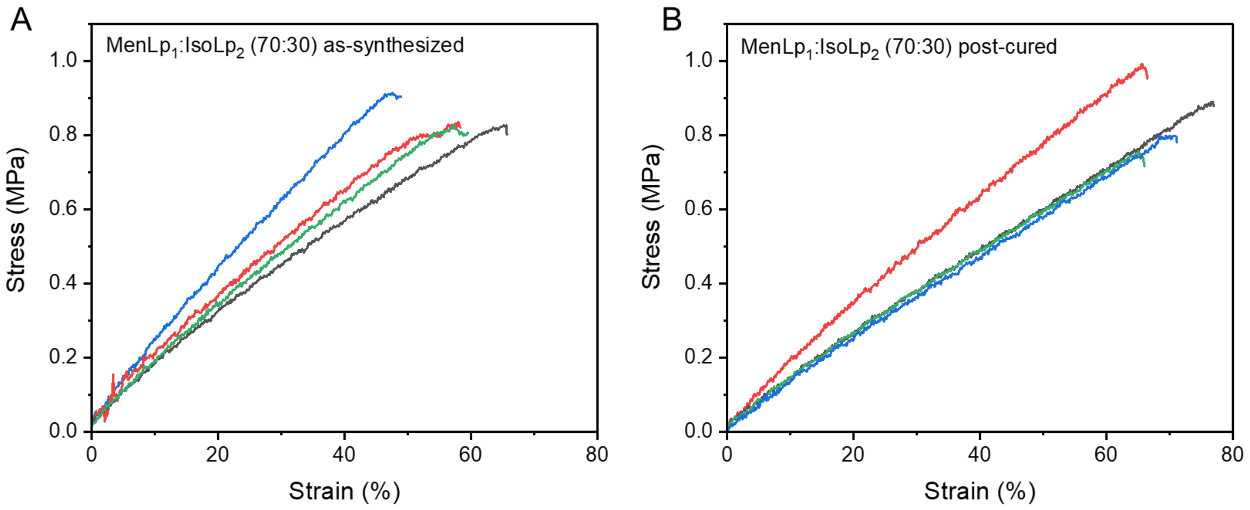

Fig. S122.
Stress *vs* strain curves of MenLp1:IsoLp2 (70:30 wt%) 2D-photoset tested at 10 mm min^{-1} strain rate (A) as-synthesized samples (B) post-cured samples.

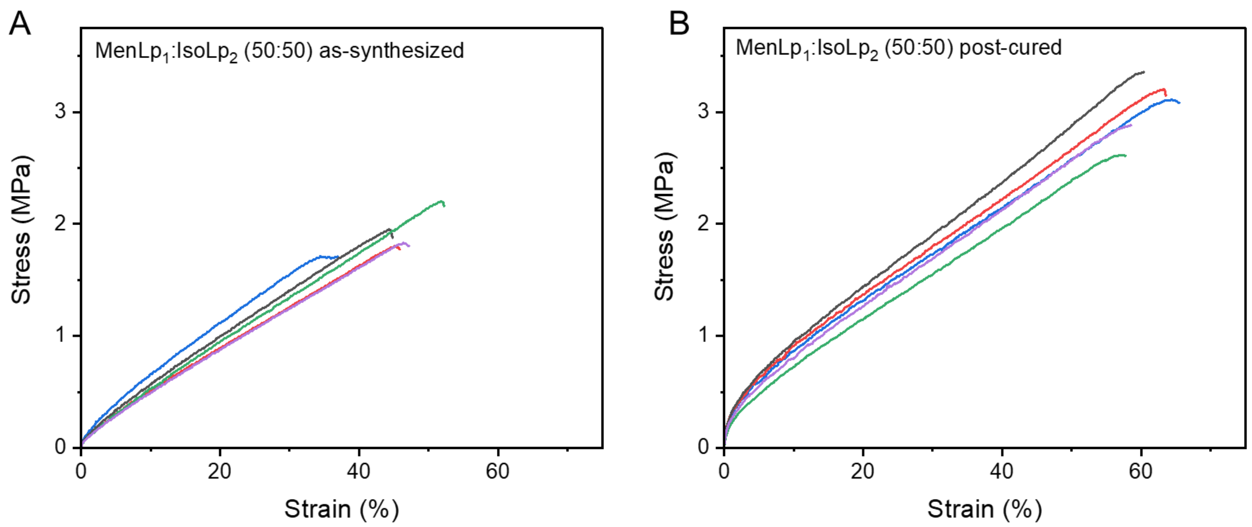

Fig. S123.
Stress *vs* strain curves of MenLp1:IsoLp2 (50:50 wt%) 2D-photoset tested at 10 mm min^{-1} strain rate (A) as-synthesized samples (B) post-cured samples.

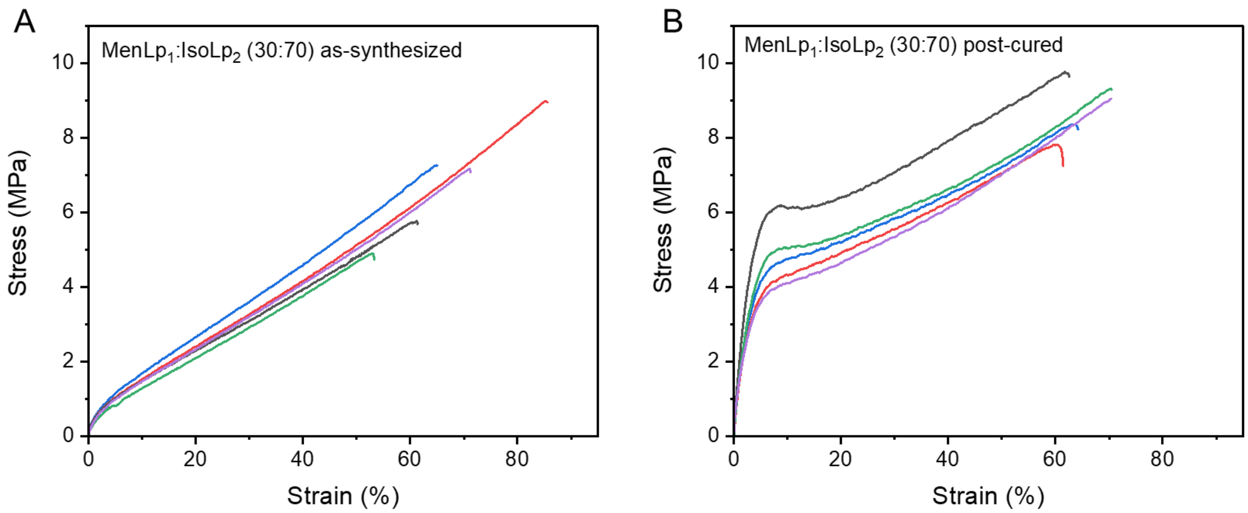

Fig. S124.
Stress *vs* strain curves of MenLp1:IsoLp2 (30:70 wt%) 2D-photoset tested at 10 mm min^{-1} strain rate (A) as-synthesized samples (B) post-cured samples.

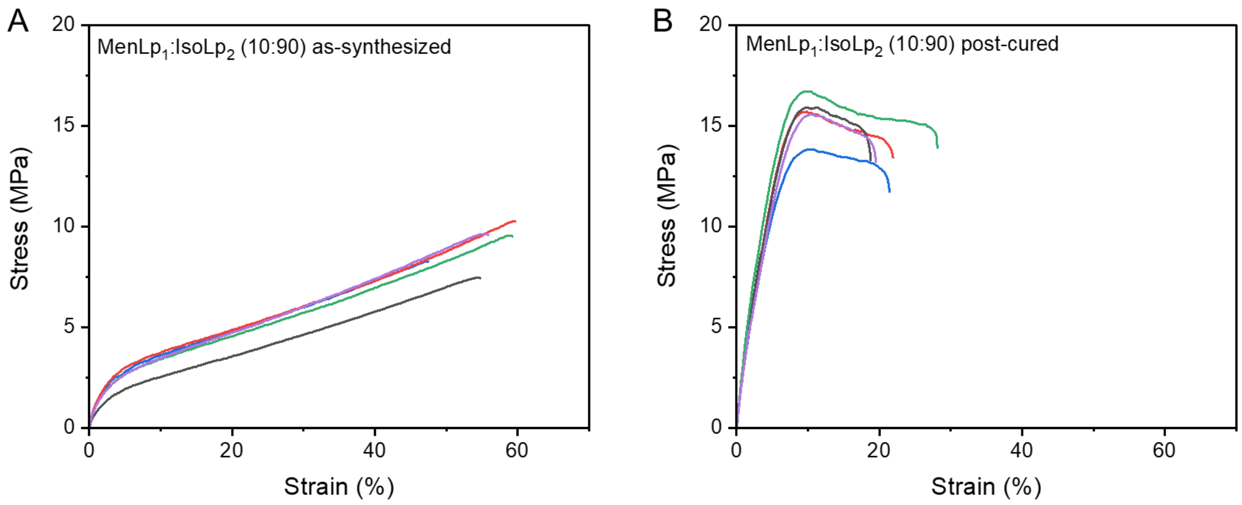

Fig. S125.
Stress *vs* strain curves of MenLp1:IsoLp2 (10:90 wt%) 2D-photoset tested at 10 mm min^{-1} strain rate (A) as-synthesized samples (B) post-cured samples.

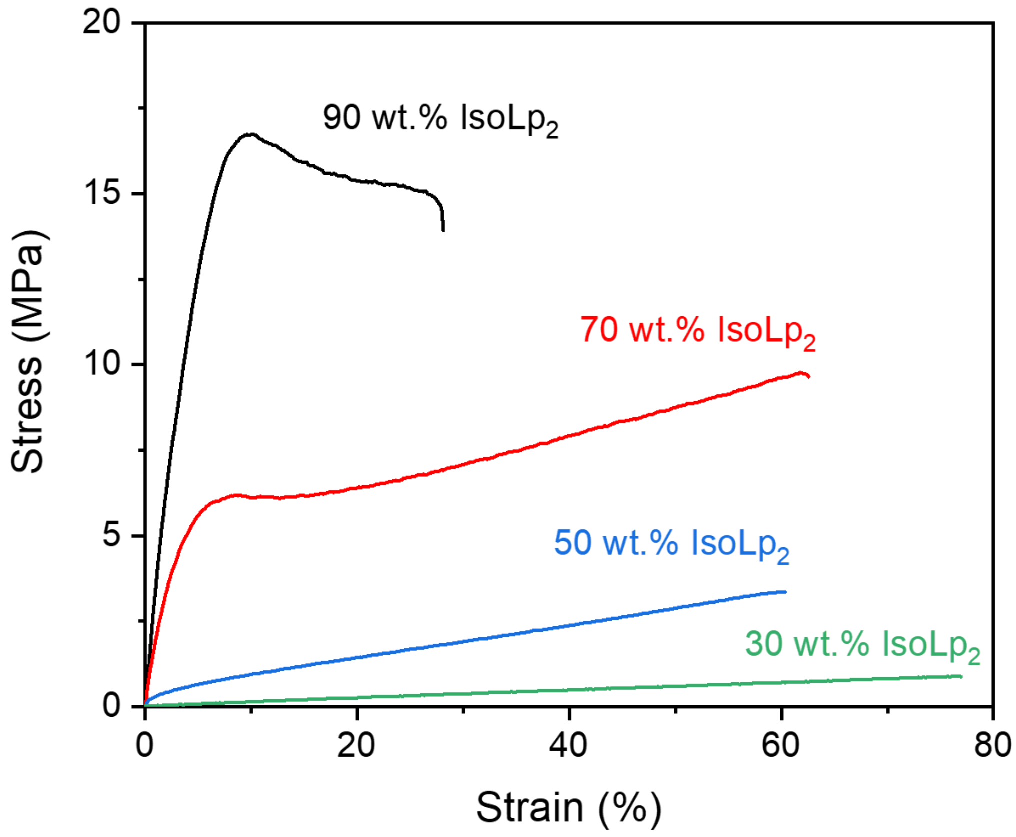

Fig. S126.
Representative stress *vs* strain curves of MenLp1:IsoLp2 (70:30 to 10:90 wt%) post-cured 2D-photoset tested at 10 mm min^{-1} strain rate.

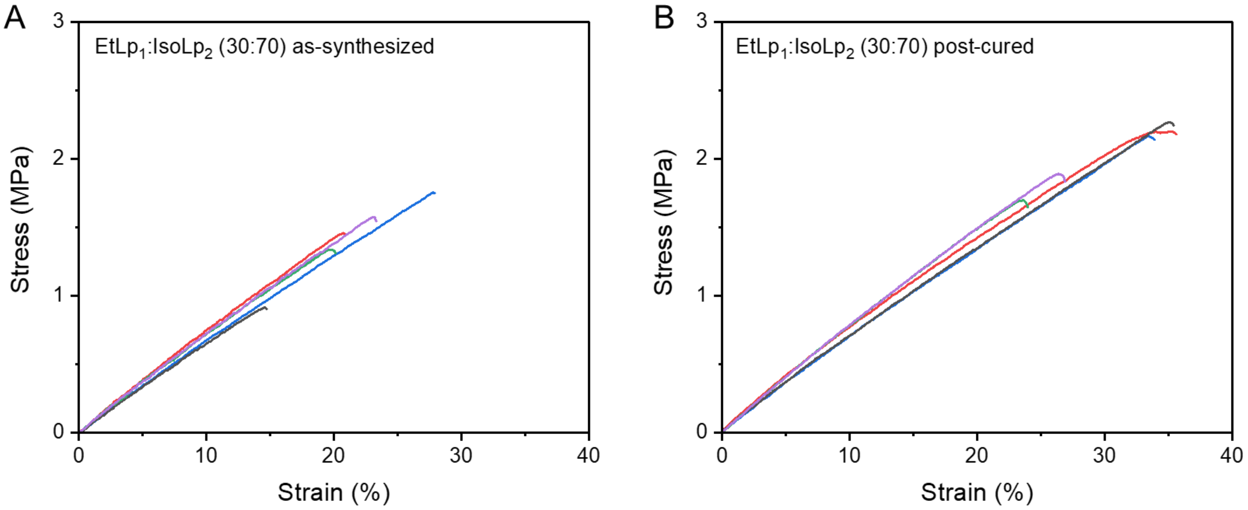

Fig. S127.
Stress *vs* strain curves of $EtLp_1$:$IsoLp_2$ (30:70 wt%) 2D-photoset tested at 10 mm min^{-1} strain rate (A) as-synthesized samples (B) post-cured samples.

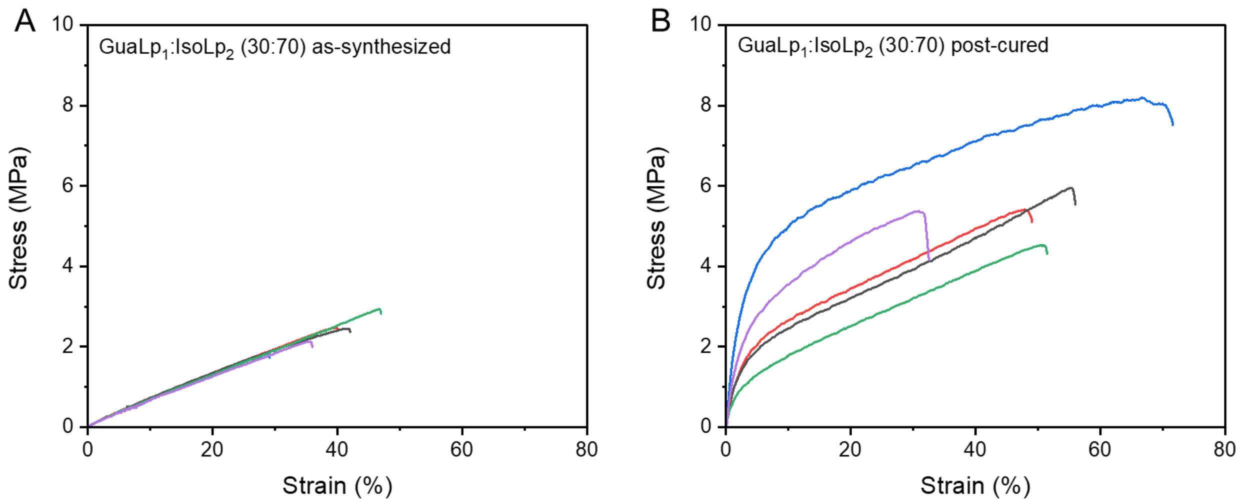

Fig. S128.
Stress *vs* strain curves of GuaLp1:IsoLp2 (30:70 wt%) 2D-photoset tested at 10 mm min^{-1} strain rate (A) as-synthesized samples (B) post-cured samples.

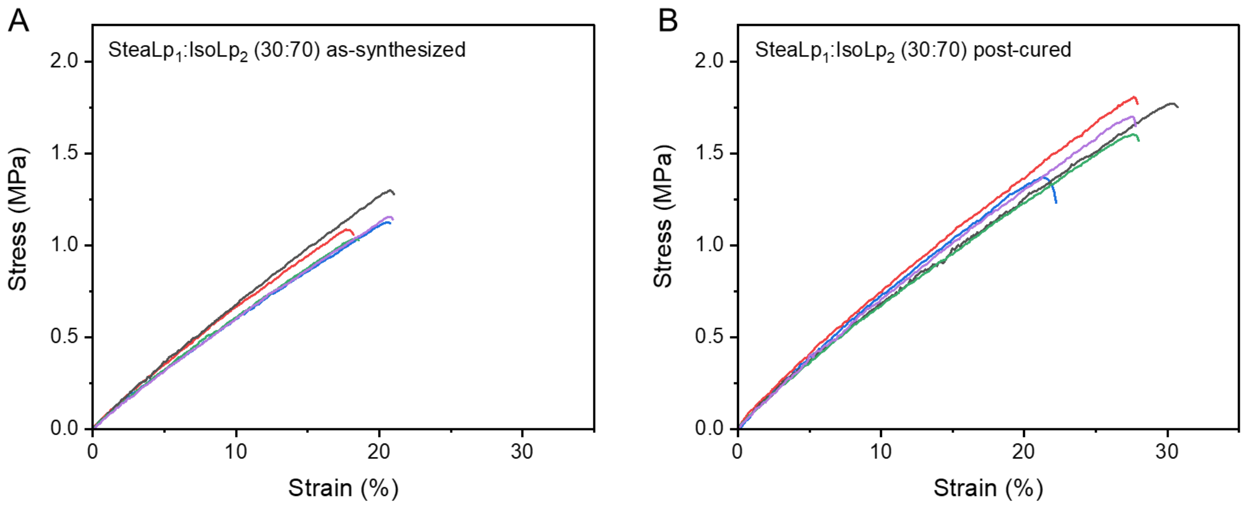

Fig. S129.
Stress *vs* strain curves of $SteaLp_1$:$IsoLp_2$ (30:70 wt%) 2D-photoset tested at 10 mm min^{-1} strain rate (A) as-synthesized samples (B) post-cured samples.

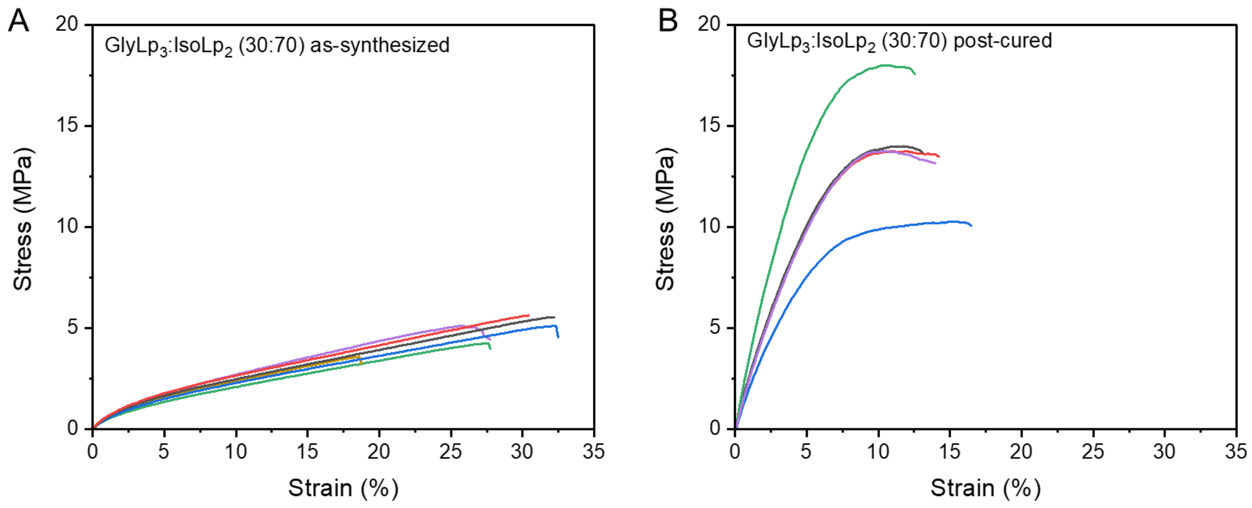

Fig. S130.
Stress *vs* strain curves of GlyLp3:IsoLp2 (30:70 wt%) 2D-photoset tested at 10 mm min^{-1} strain rate (A) as-synthesized samples (B) post-cured samples.

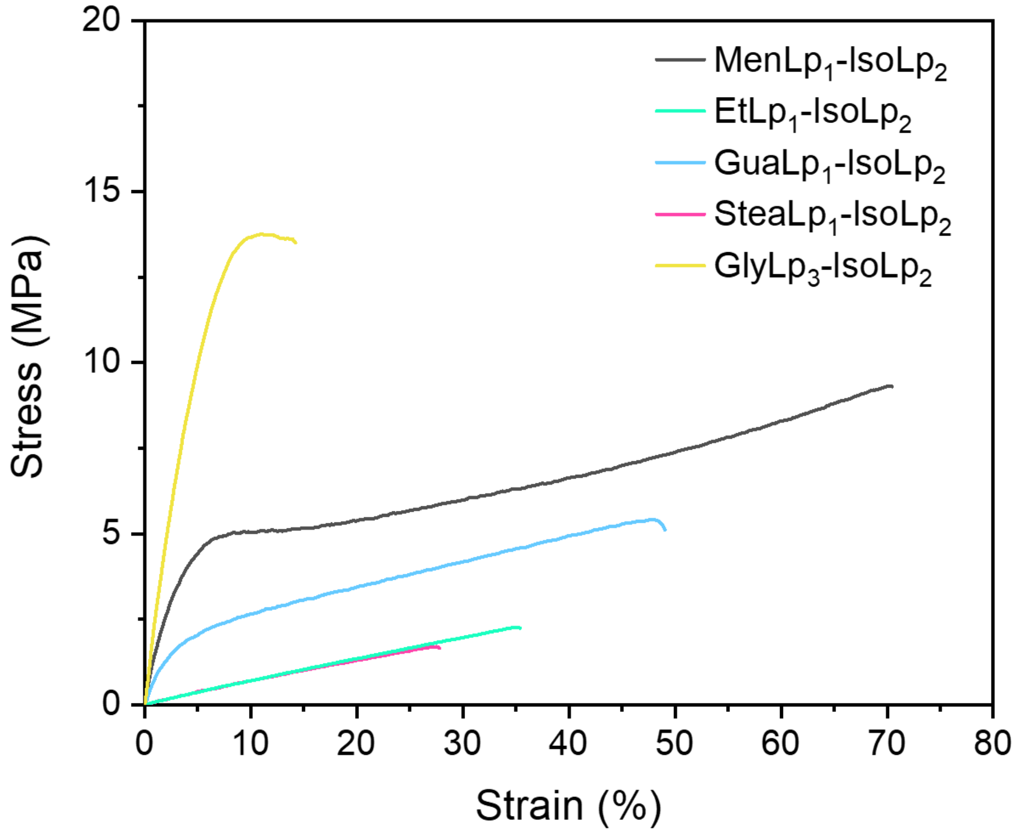

Fig. S131.
Representative stress *vs* strain curves of R-Lp_x:$IsoLp_2$ (30:70 wt%) post-cured 2D-photoset tested at 10 mm min^{-1} strain rate.

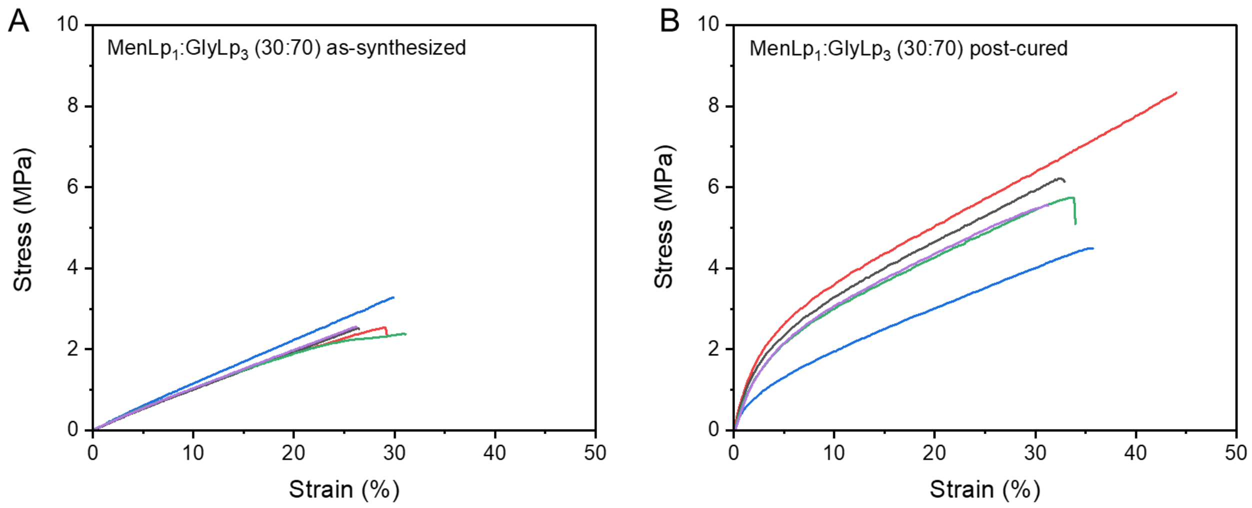

Fig. S132.
Stress *vs* strain curves of MenLp1:GlyLp3 (30:70 wt%) 2D-photoset tested at 10 mm min^{-1} strain rate (A) as-synthesized samples (B) post-cured samples.

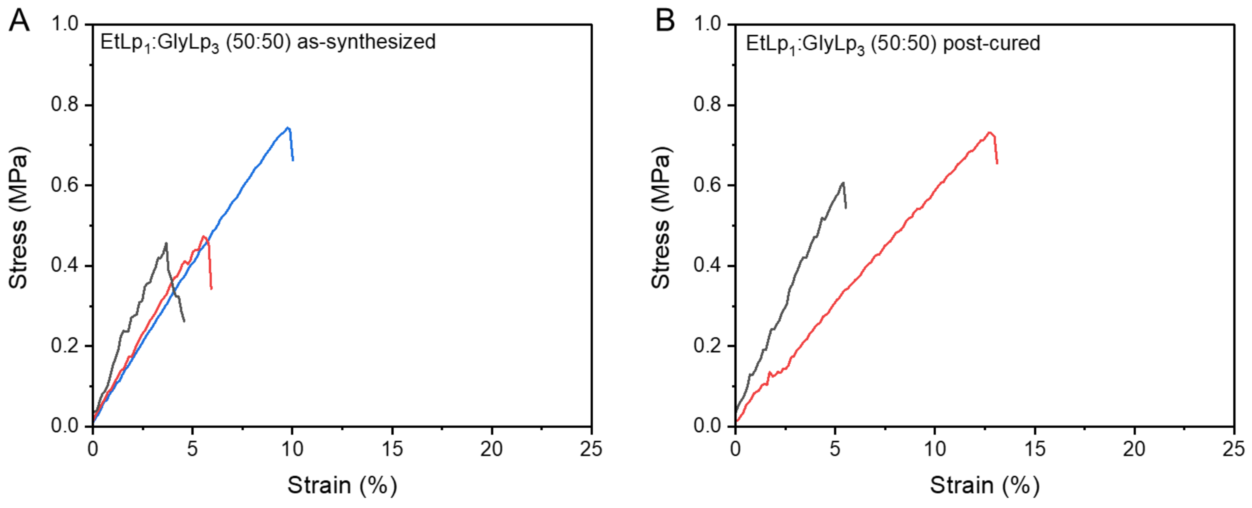

Fig. S133.
Stress *vs* strain curves of EtLp1:GlyLp3 (50:50 wt%) 2D-photoset tested at 10 mm min^{-1} strain rate (A) as-synthesized samples (B) post-cured samples.

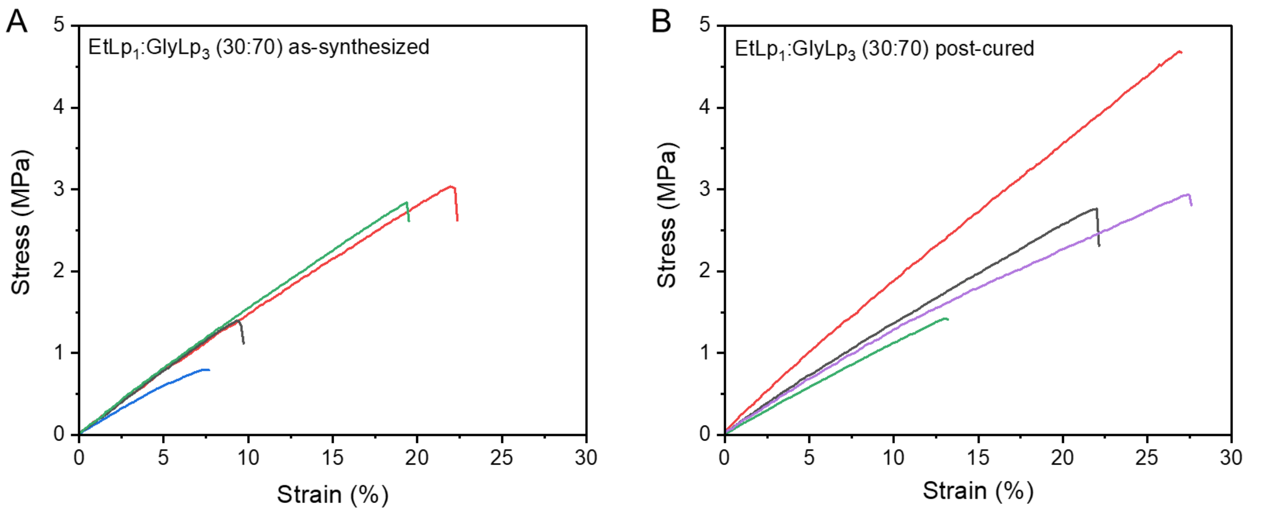

Fig. S134.
Stress *vs* strain curves of $EtLp_1$:$GlyLp_3$ (30:70 wt%) 2D-photoset tested at 10 mm min^{-1} strain rate (A) as-synthesized samples (B) post-cured samples.

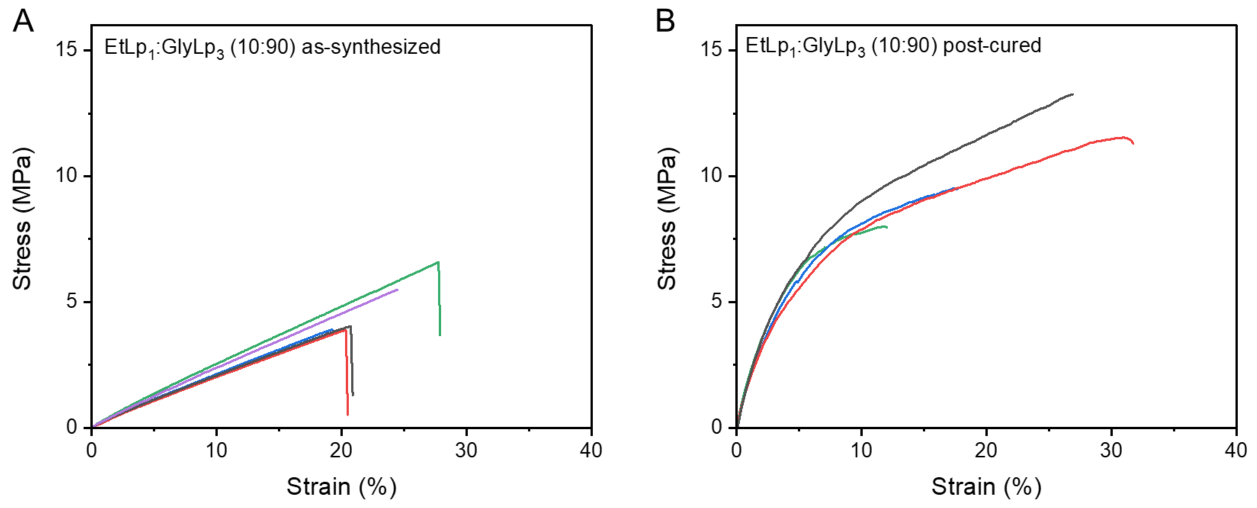

Fig. S135.
Stress *vs* strain curves of EtLp1:GlyLp3 (10:90 wt%) 2D-photoset tested at 10 mm min^{-1} strain rate (A) as-synthesized samples (B) post-cured samples.

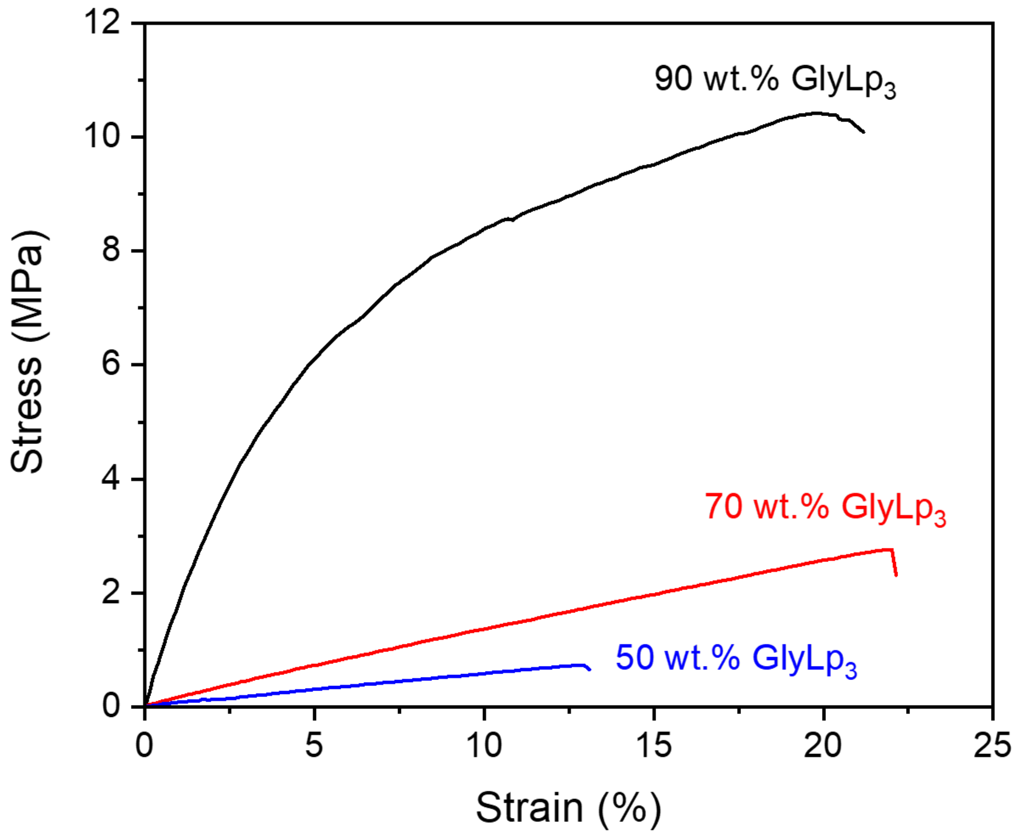

Fig. S136.
Representative stress *vs* strain curves of $EtLp_1$:$GlyLp_3$ (50:50 to 10:90 wt%) post-cured 2D-photoset tested at 10 mm min^{-1} strain rate.

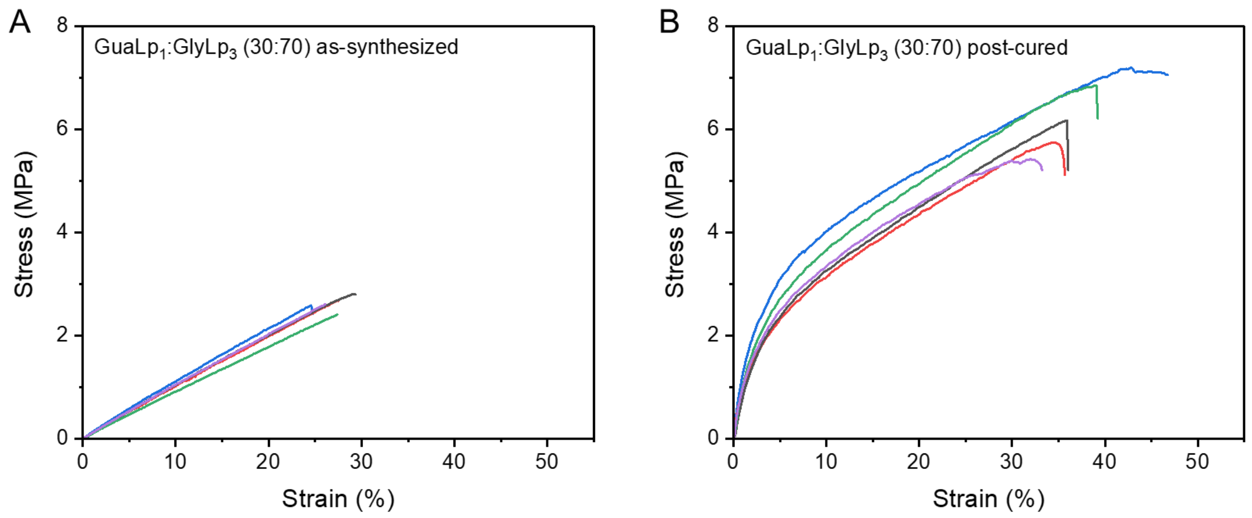

Fig. S137.
Stress *vs* strain curves of GuaLp$_1$:GlyLp$_3$ (30:70 wt%) 2D-photoset tested at 10 mm min^{-1} strain rate (A) as-synthesized samples (B) post-cured samples.

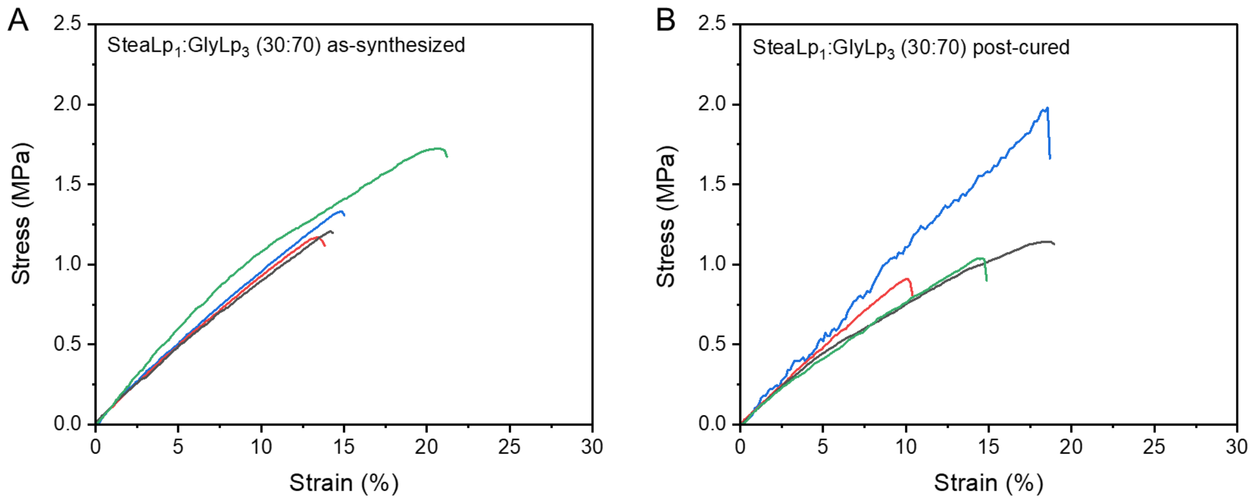

Fig. S138.
Stress *vs* strain curves of SteaLp1:GlyLp3 (30:70 wt%) 2D-photoset tested at 10 mm min^{-1} strain rate (A) as-synthesized samples (B) post-cured samples.

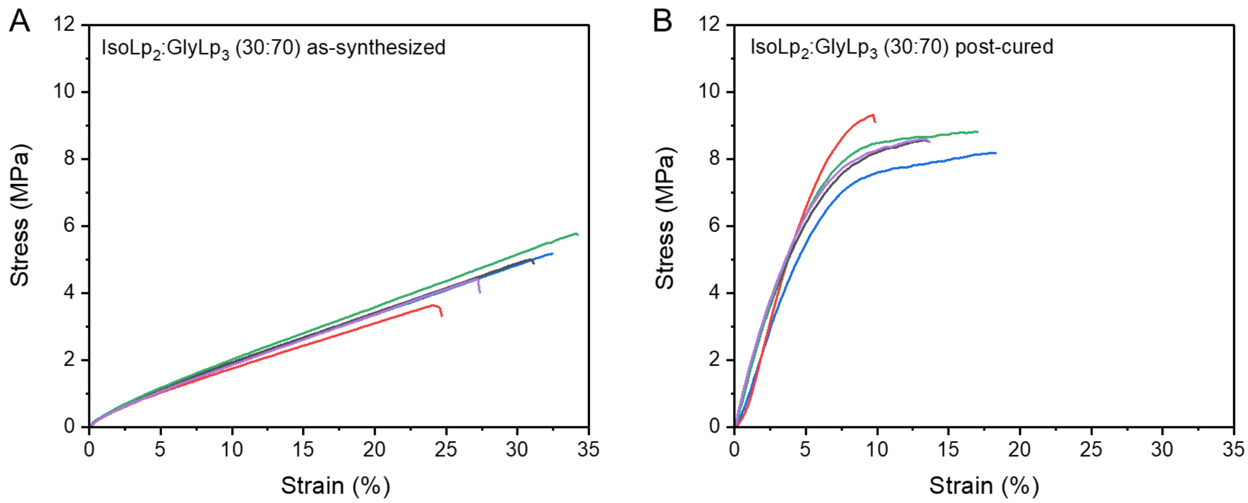

Fig. S139.
Stress *vs* strain curves of IsoLp2:GlyLp3 (30:70 wt%) 2D-photoset tested at 10 mm min^{-1} strain rate (A) as-synthesized samples (B) post-cured samples.

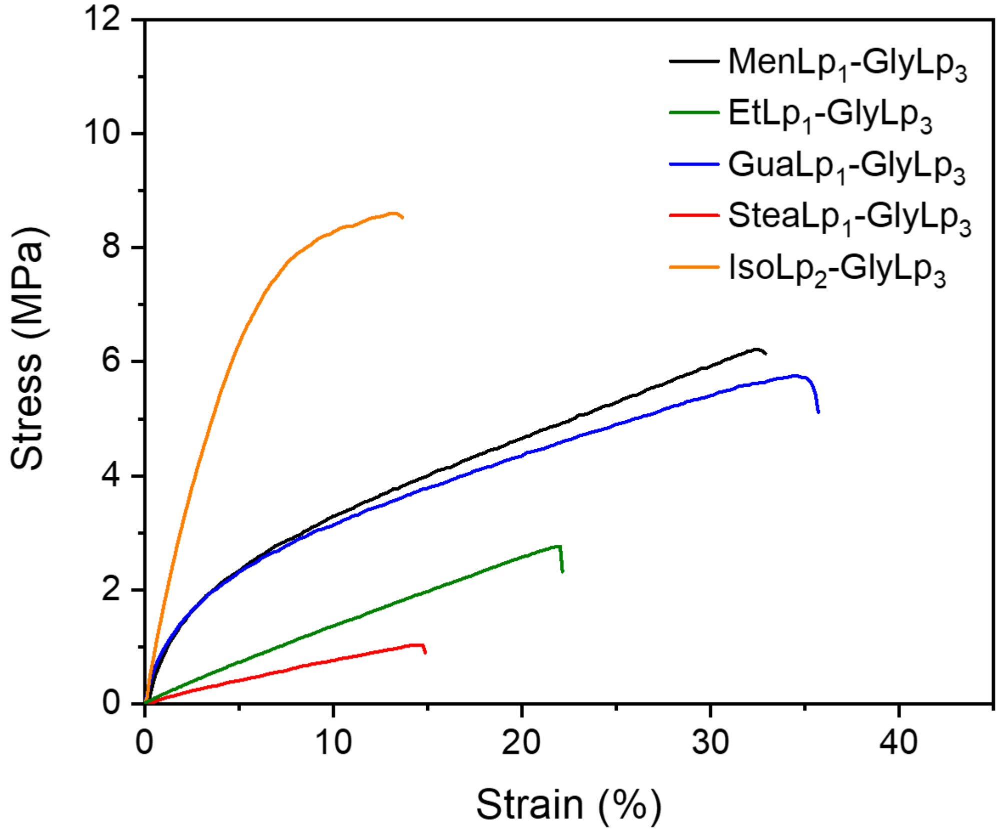

Fig. S140.
Representative stress *vs* strain curves of R-Lp_x:$GlyLp_3$ (30:70 wt%) post-cured 2D-photoset tested at 10 mm min^{-1} strain rate.

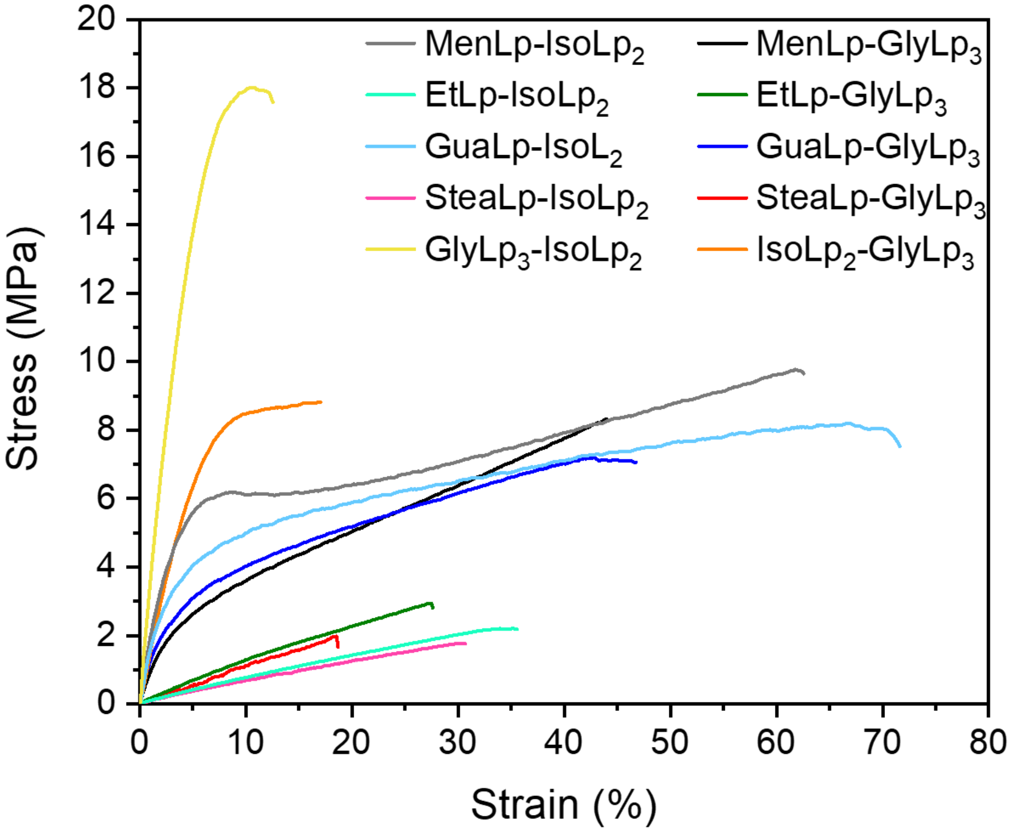

Fig. S141.
Representative stress *vs* strain curves of R-Lp_x:$GlyLp_3$ and R-Lp_x:$IsoLp_2$ (30:70 wt%) post-cured 2D-photoset at 10 mm min^{-1} strain rate.

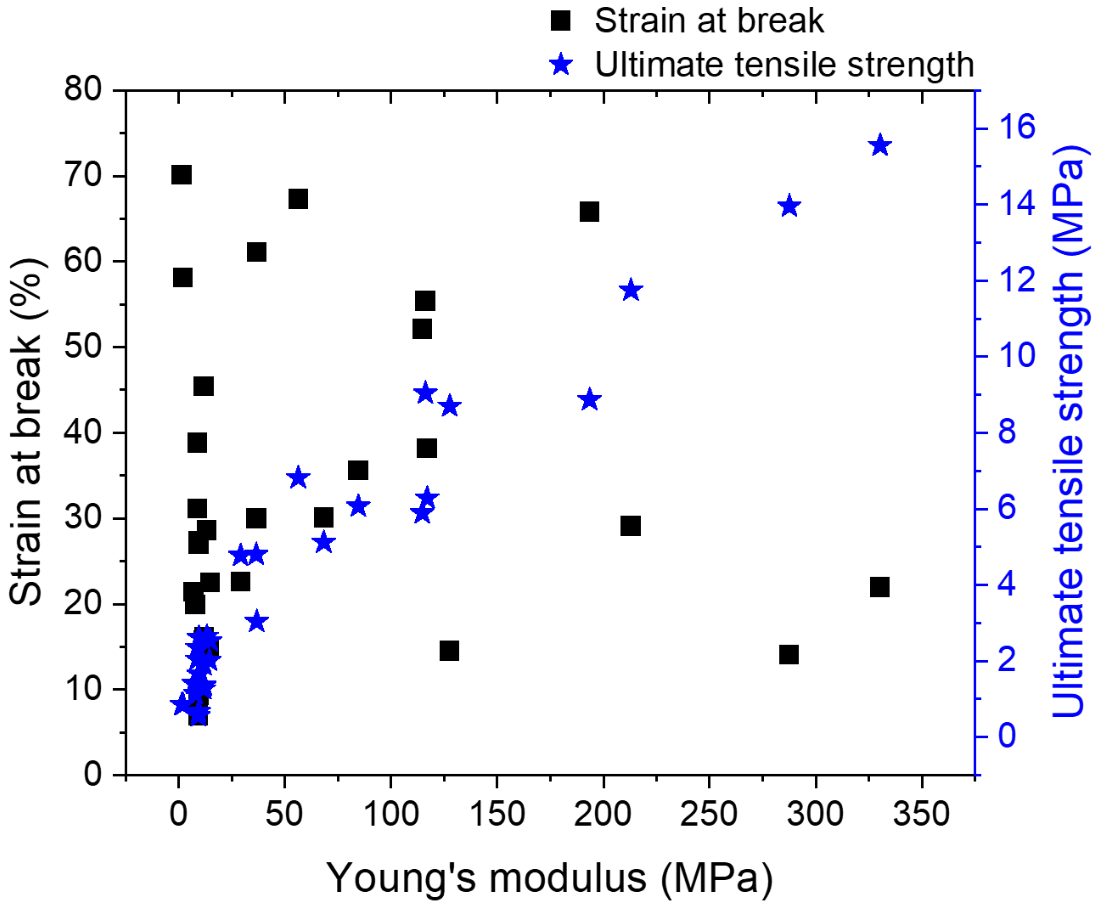

Fig. S142.
Mechanical property scope for all surveyed 2D-photosets (post-cured samples).

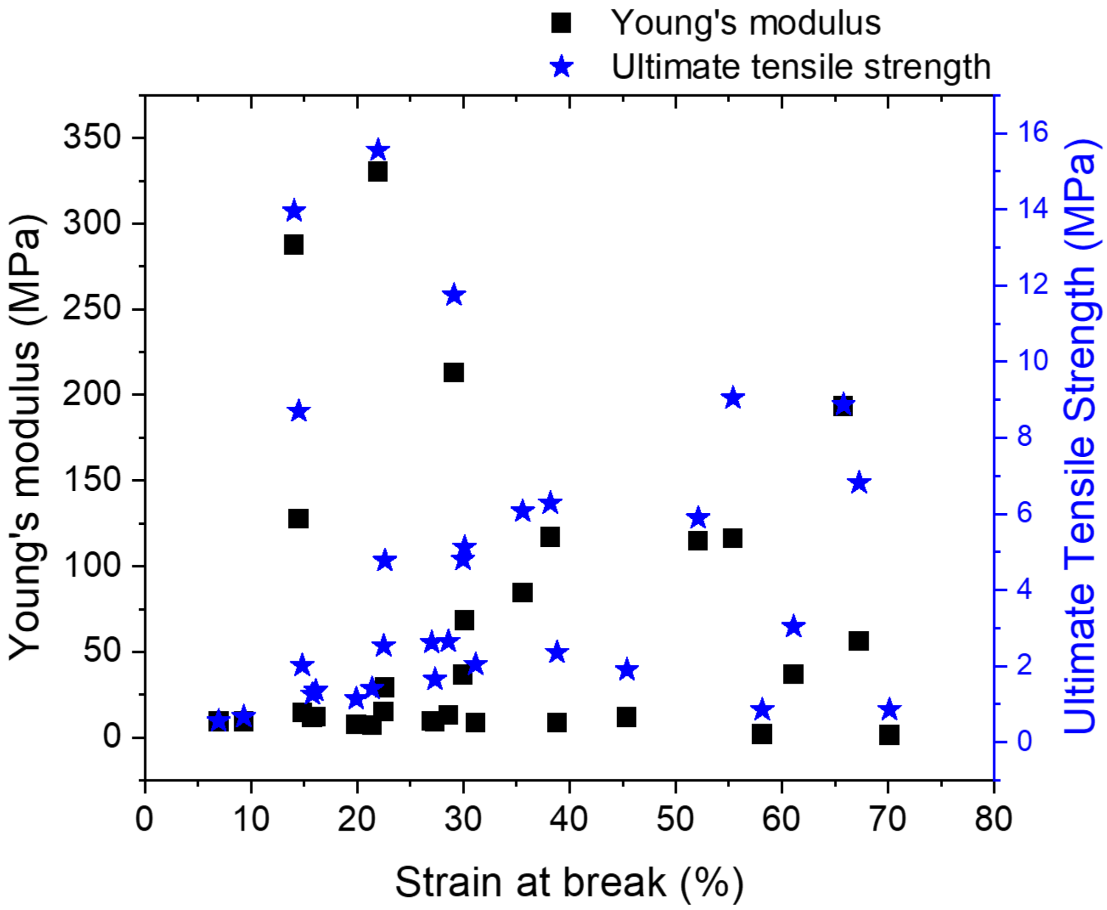

Fig. S143.
Mechanical property scope for all surveyed 2D-photosets (post-cured samples).

Recycling of 2D photosets with thermal depolymerization (DMF, 140 °C) method

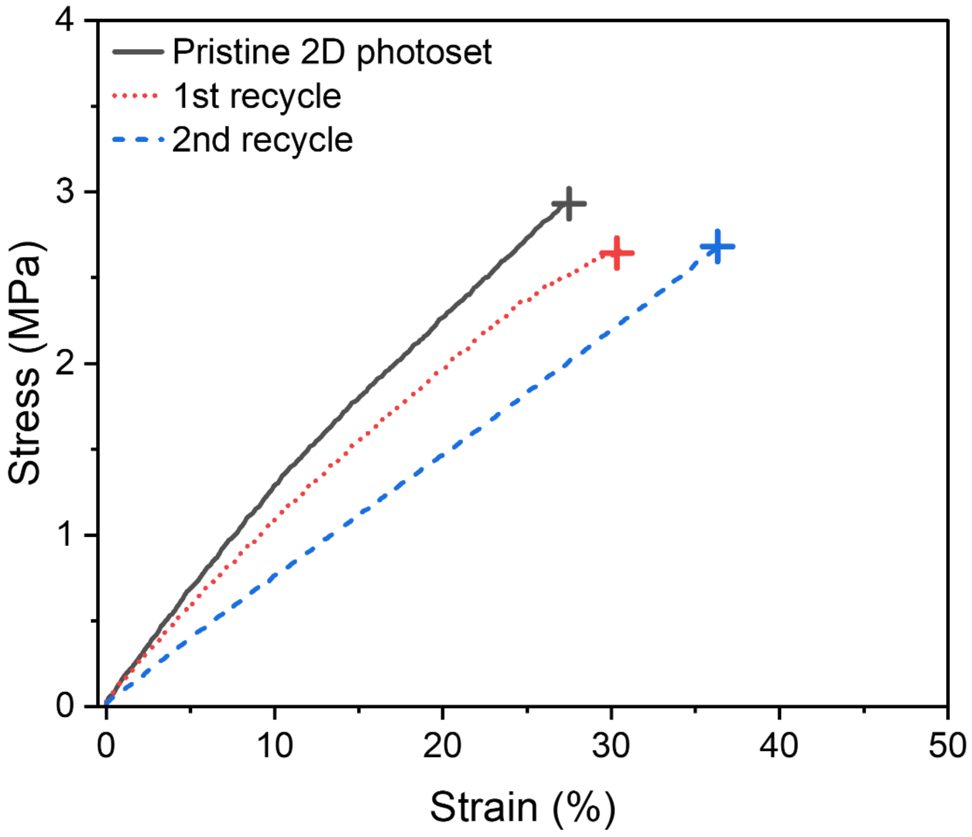

Fig. S144.
Representative stress *vs* strain curves of 2D-photoset for $EtLp_1$:$GlyLp_3$ (30:70 wt%) obtained from **pristine, 1st recycle, 2nd recycled** resins. Recycled resins obtained from thermal depolymerization (DMF, 140 °C) method. Tested at 10 mm min^{-1} strain rate.

Recycling and re-curing/printing resins using catalyzed depolymerization (phosphazene:thiophenol) method

Photorheology data

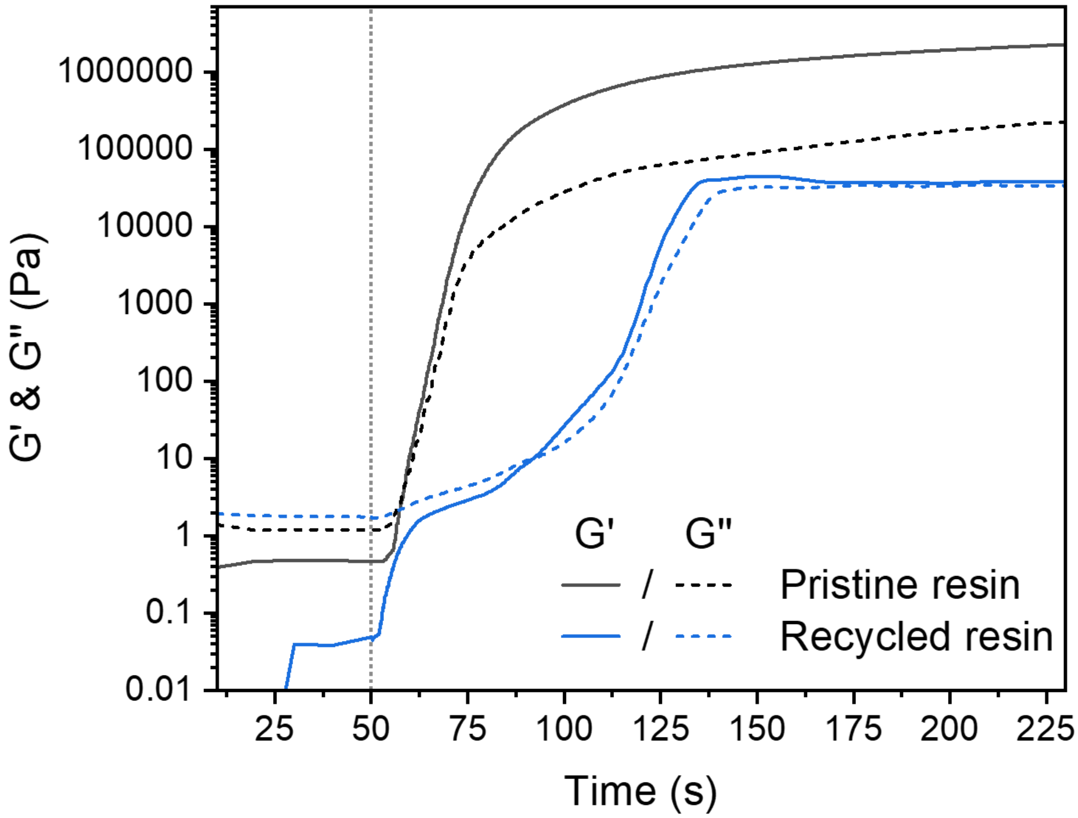

Fig. S145.
Photorheology of $MenLp_1:IsoLp_2$ (30:70 wt%) pristine resin (black) and recycled resin (blue) taken over 230 s under oscillatory shear at ambient temperature.

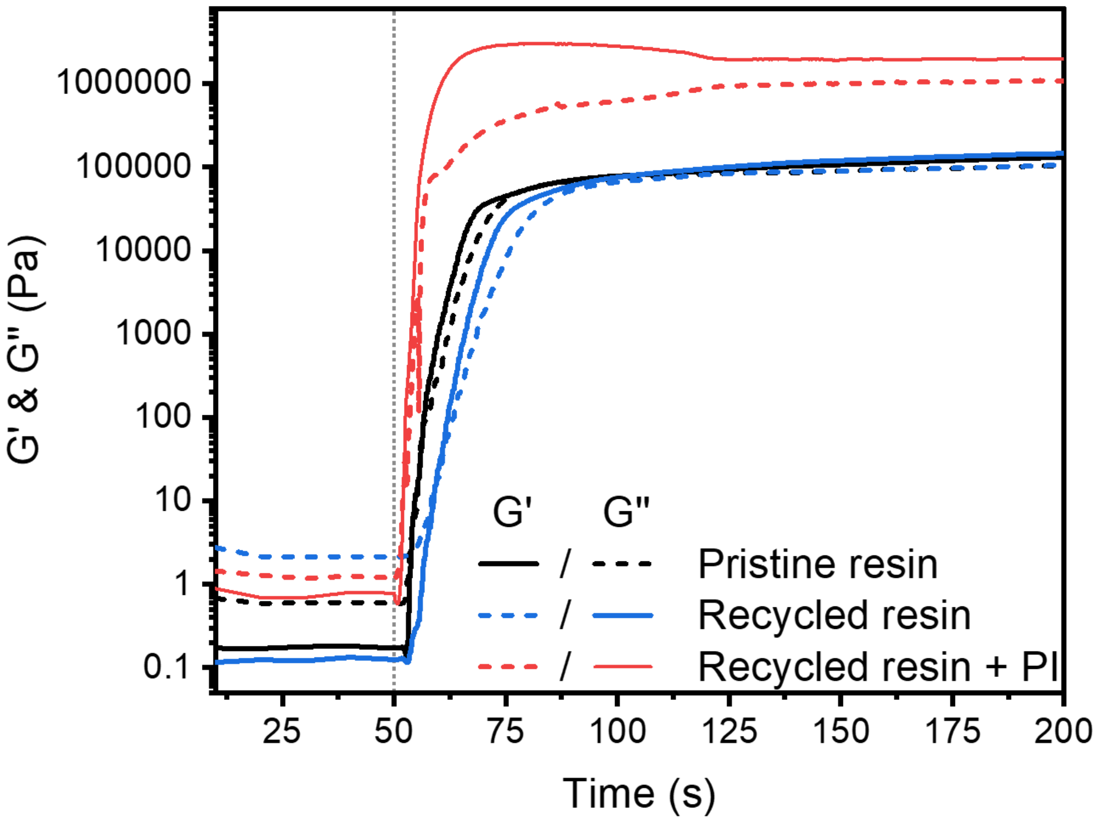

Fig. S146.
Photorheology of $EtLp_1$:$GlyLp_3$ (30:70 wt%) pristine resin (black), recycled resin (blue), and recycled resin containing 2 wt% photoinitiator (BAPO) taken over 230 s under oscillatory shear at ambient temperature.

3D-printing

Z-axis curing depth

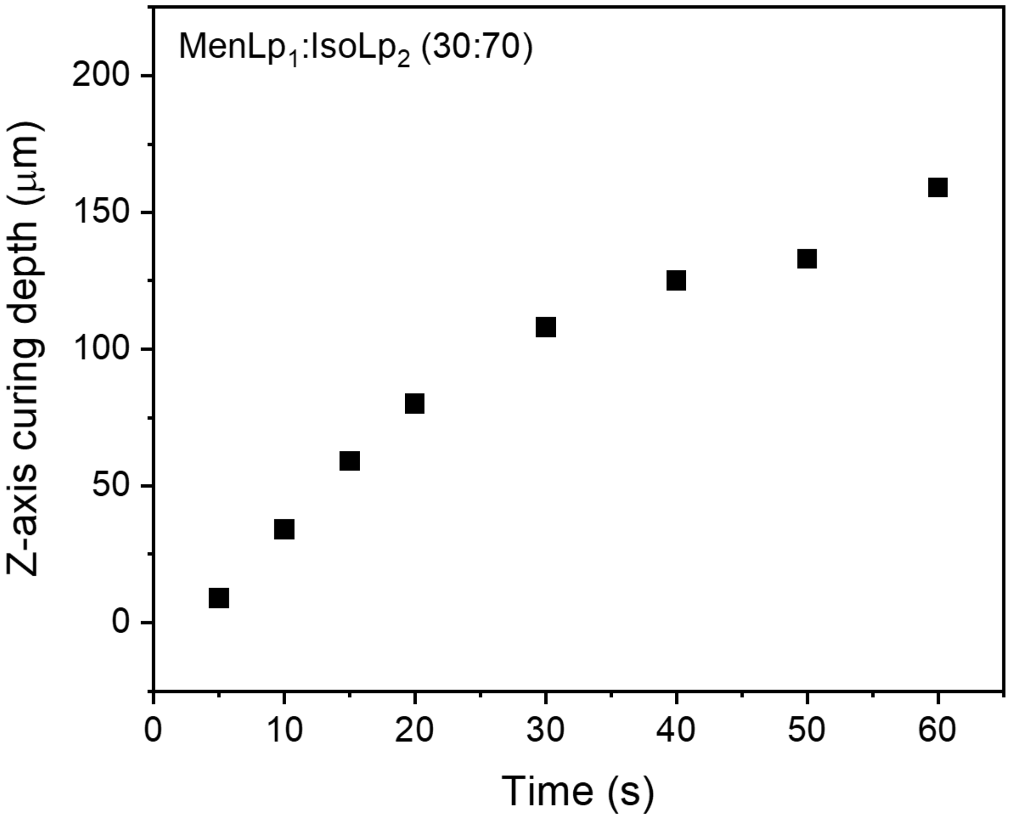

Fig. S147.

Z-depth cure screening for $MenLp_1$:$IsoLp_2$ (30:70 wt.%) **pristine** resin by irradiating a 2D-square and measuring sample thickness (Z-axis depth) vs irradiation time.

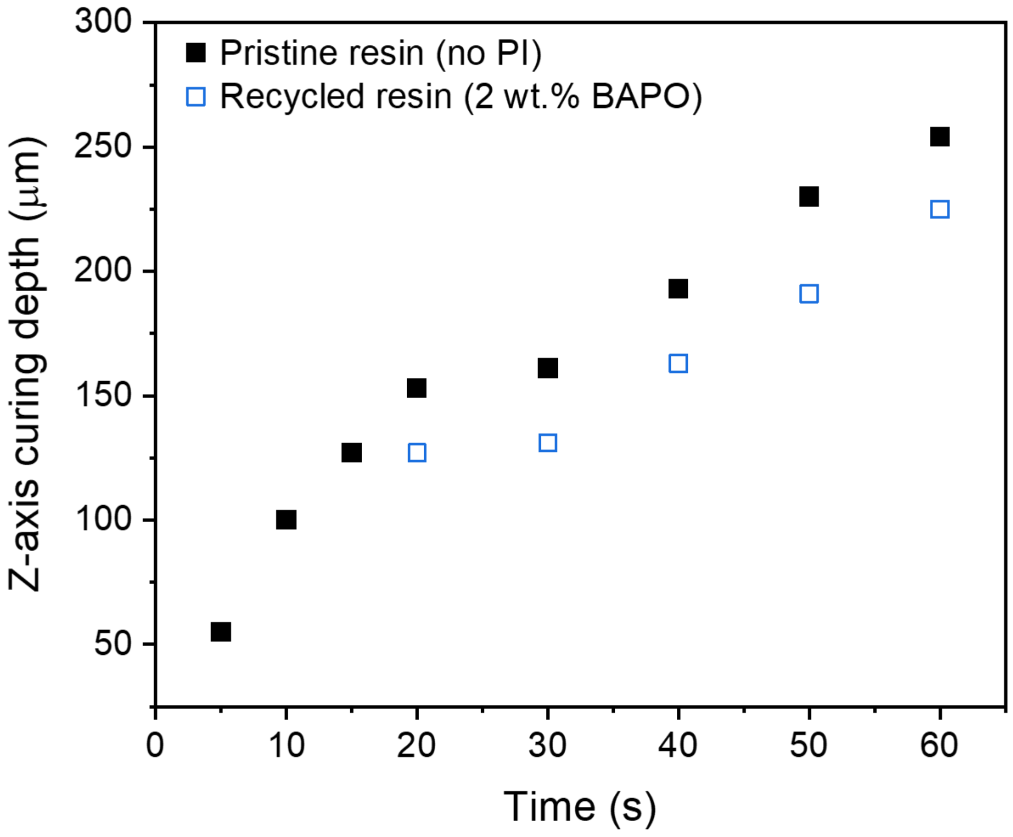

Fig. S148.
Z-depth cure screening for $EtLp_1$:$GlyLp_3$ (30:70 wt.%) resins (**pristine** and **recycled**) by irradiating a 2D-square and measuring sample thickness (Z-axis depth) vs irradiation time.

3D-printing of square arrays and bridges

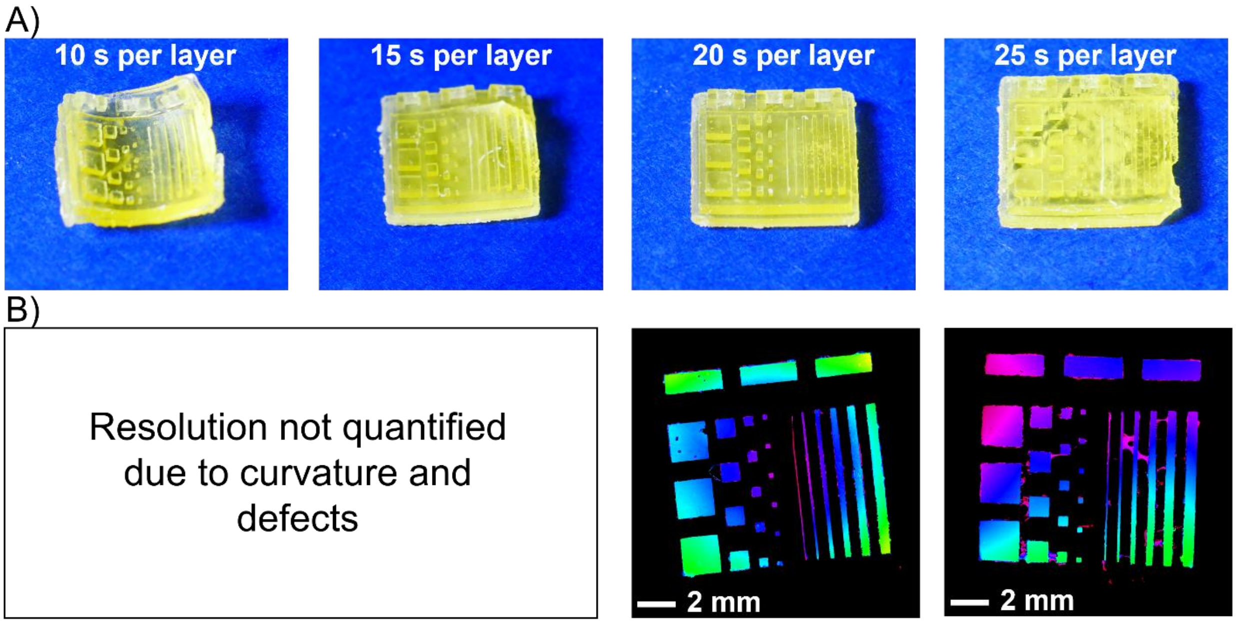

Fig. S149.
3D-printed bridges and square arrays at various cure times (s/layer) using MenLp1:IsoLp2 (30:70 wt.%) **pristine** resin (A) photographs of prints and (B) corresponding image analysis.

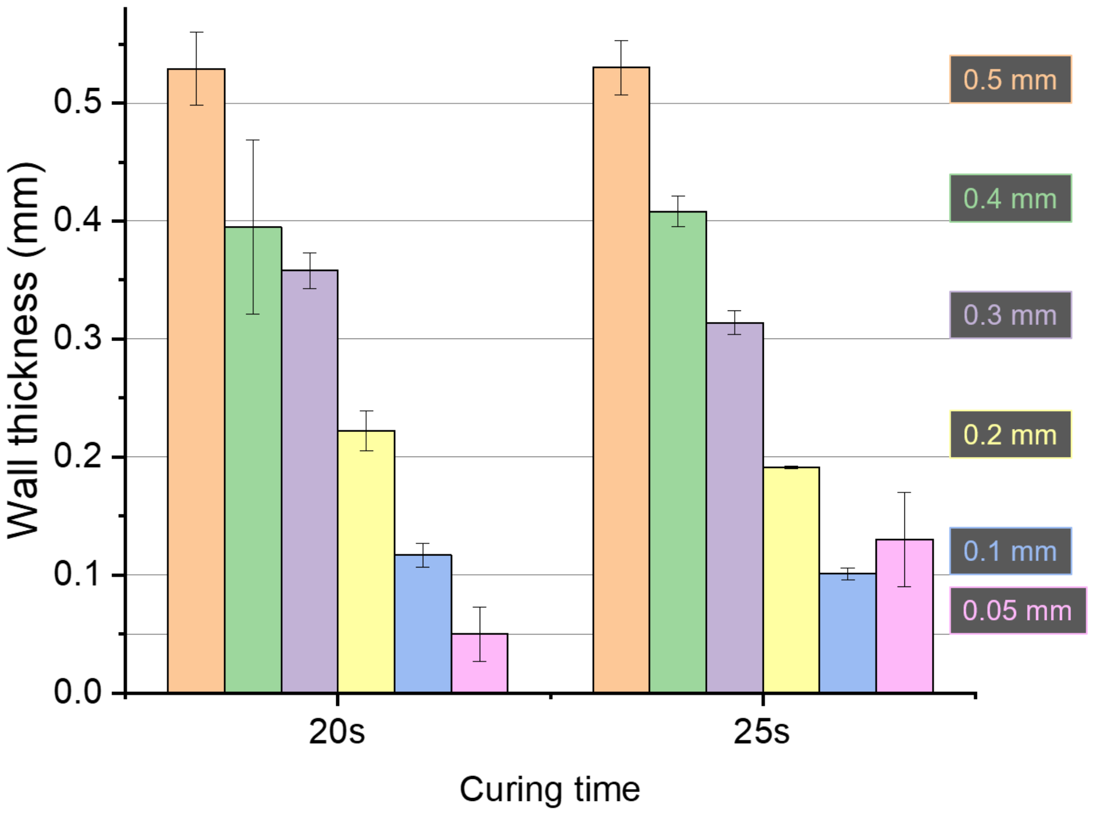

Fig. S150.
X-Y printing accuracy determined by comparing wall thickness (theoretical values indicated by color-coded labels) to curing time for 3D-printed bridges and square arrays using MenLp1:IsoLp2 (30:70 wt.%) **pristine** resin.

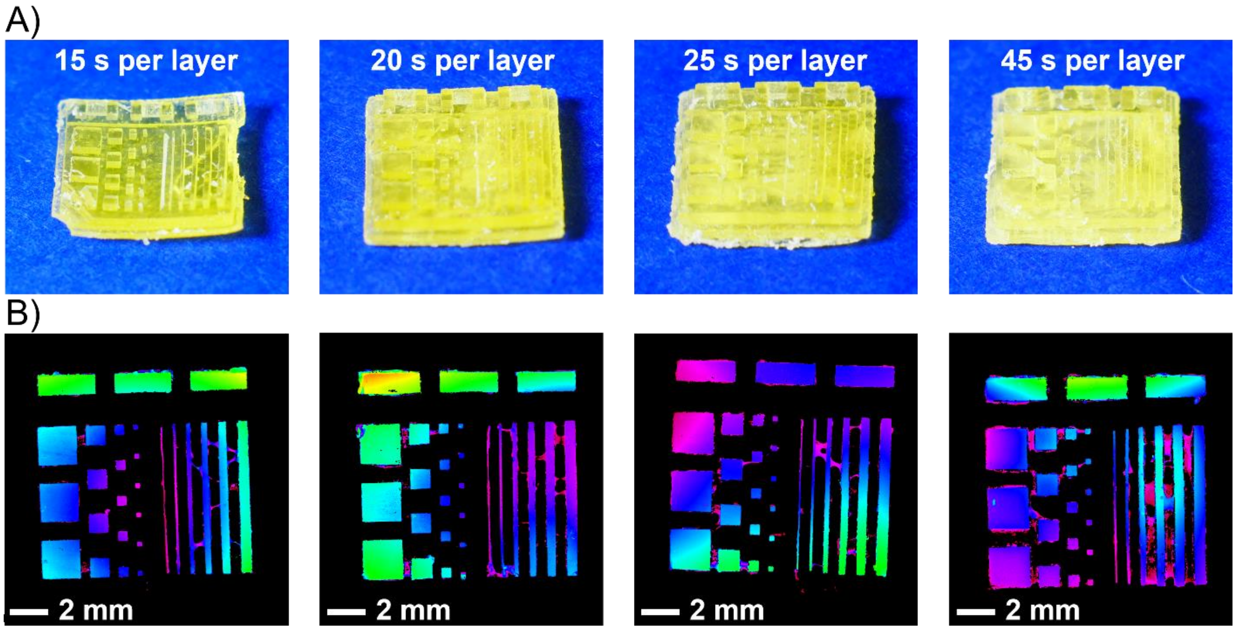

Fig. S151.
3D-printed bridges and square arrays at various cure times (s/layer) using $EtLp_1$:$GlyLp_3$ (30:70 wt.%) **pristine** resin (A) photographs of prints and (B) corresponding image analysis.

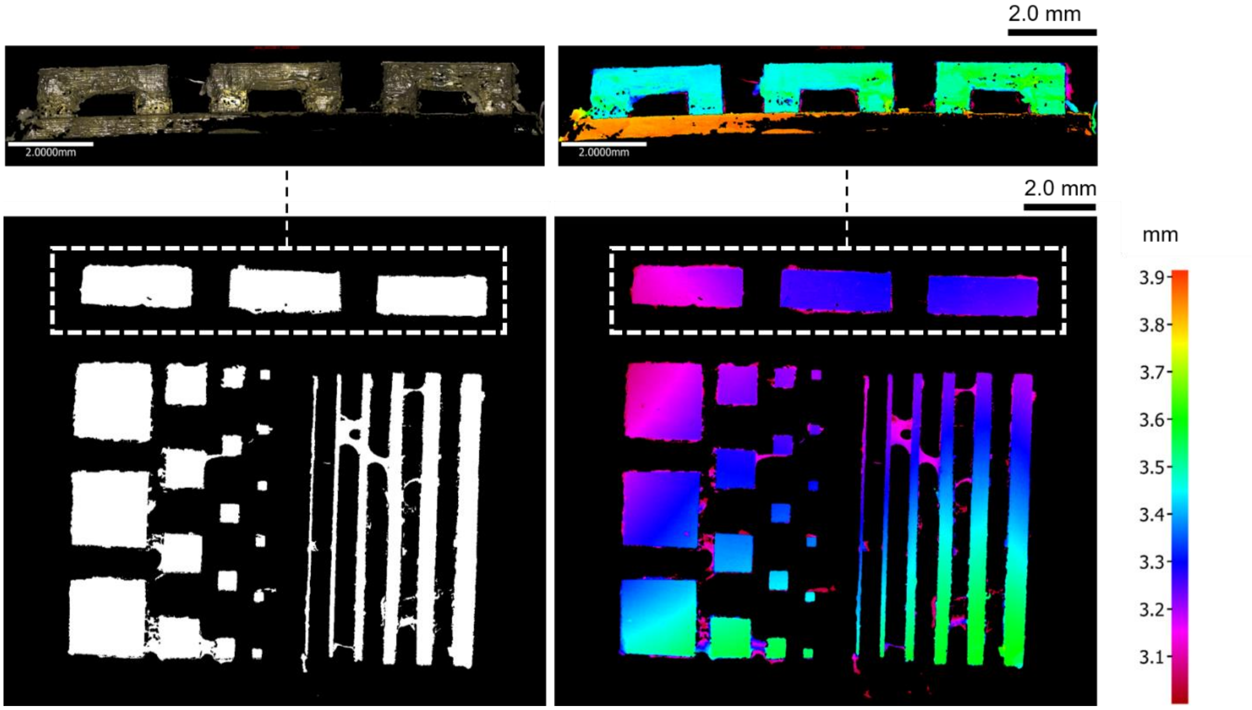

Fig. S152.
Representative image analysis of 3D-printed bridges and square arrays using $EtLp_1$:$GlyLp_3$ (30:70 wt.%) **pristine** resin. Cure time = 25 s/layer.

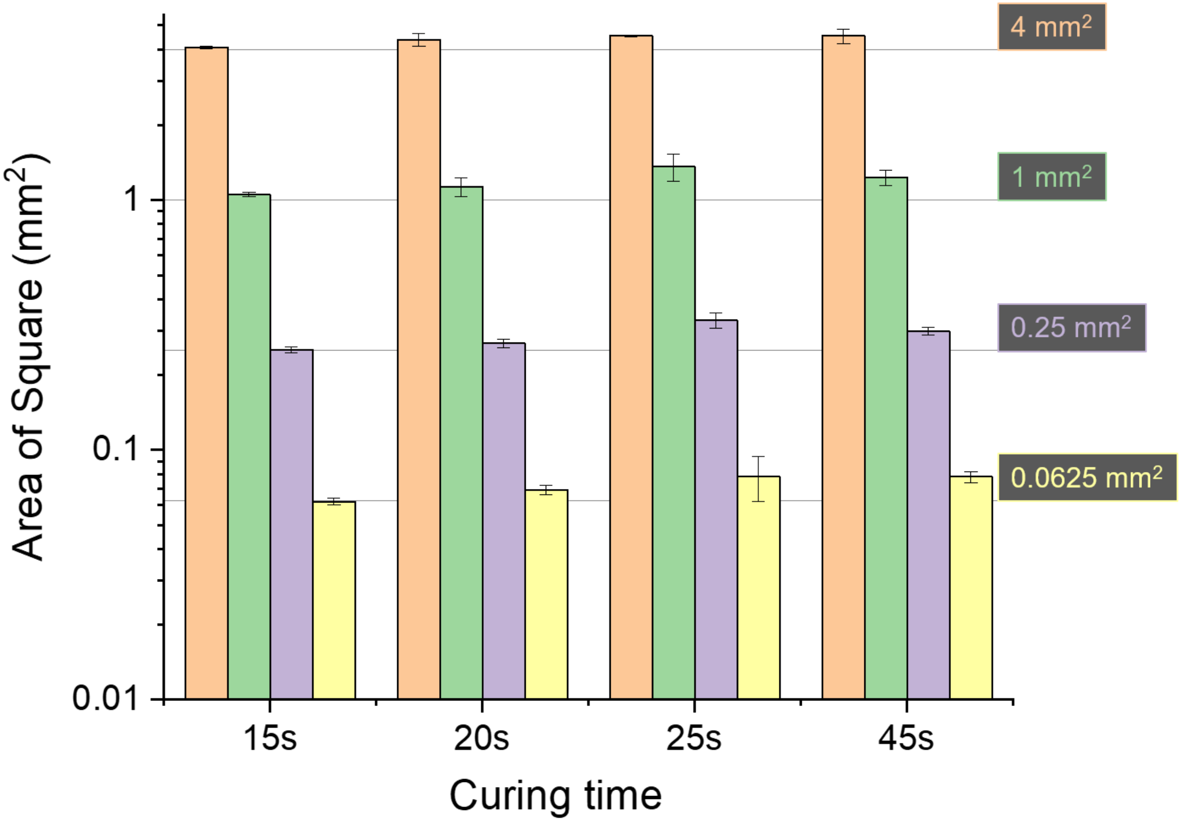

Fig. S153.
X-Y printing accuracy determined by comparing surface area of squares (theoretical values indicated by color-coded labels) to curing time for 3D-printed bridges and square arrays using $EtLp_1$:$GlyLp_3$ (30:70 wt.%) **pristine** resin.

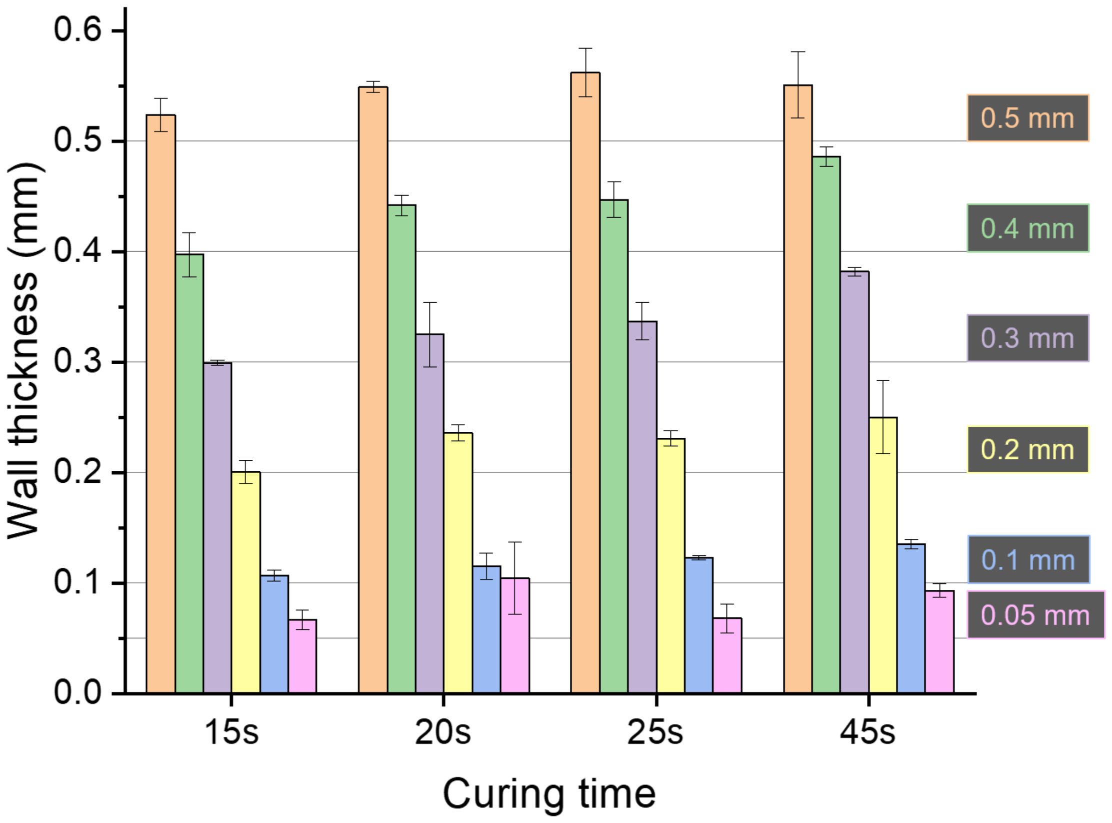

Fig. S154.
X-Y printing accuracy determined by comparing wall thickness (theoretical values indicated by color-coded labels) to curing time for 3D-printed bridges and square arrays using $EtLp_1$:$GlyLp_3$ (30:70 wt.%) **pristine** resin.

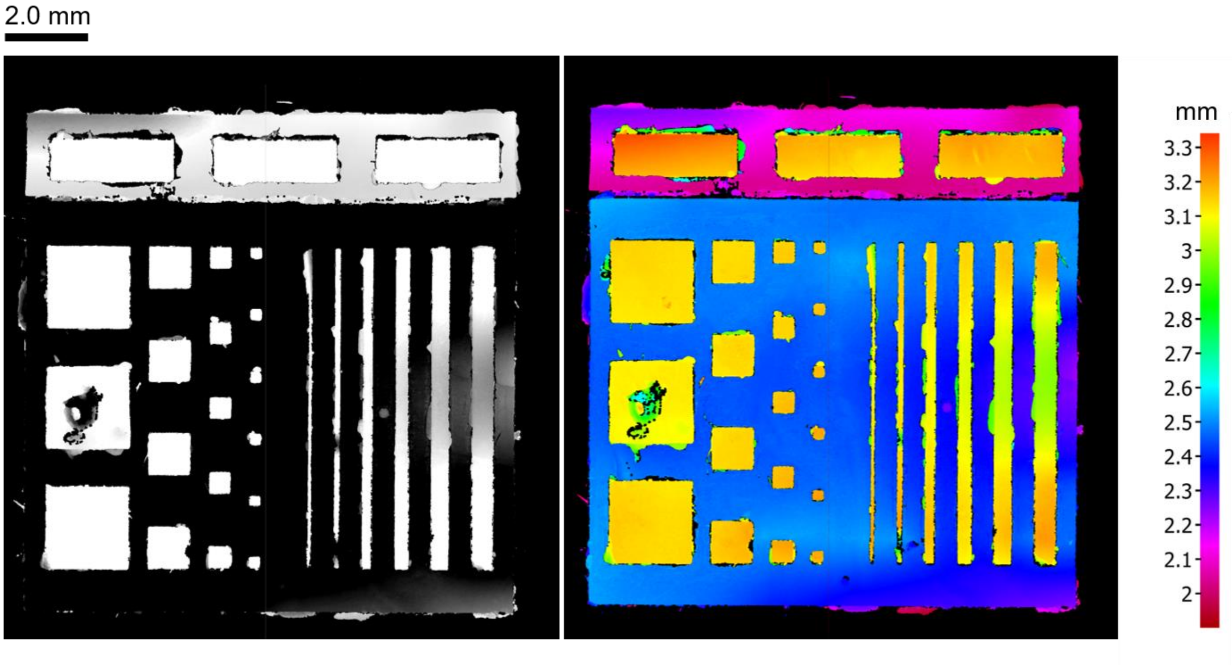

Fig. S155.
Representative image analysis of 3D-printed bridges and square arrays using $EtLp_1$:$GlyLp_3$ (30:70 wt.%) **recycled** resin. Cure time = 25 s/layer.

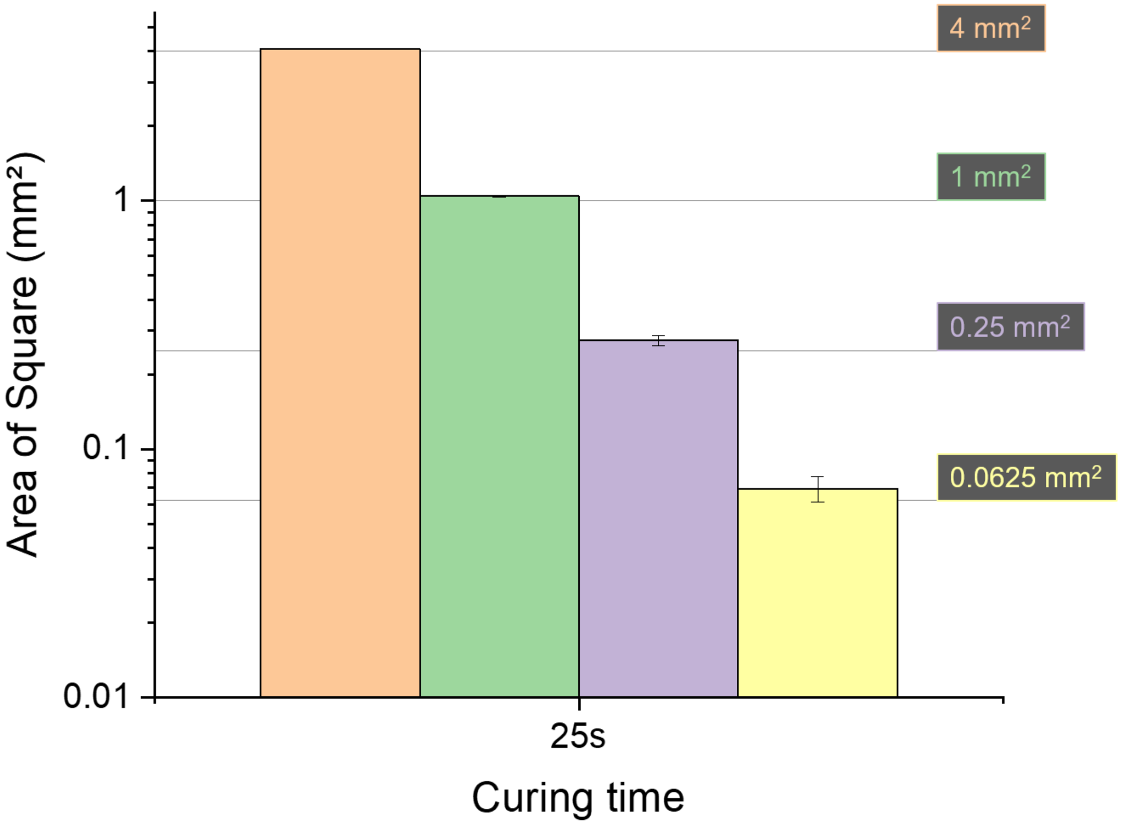

Fig. S156.
X-Y printing accuracy determined by comparing surface area of squares (theoretical values indicated by color-coded labels) to curing time for 3D-printed bridges and square arrays using $EtLp_1$:$GlyLp_3$ (30:70 wt.%) **recycled** resin.

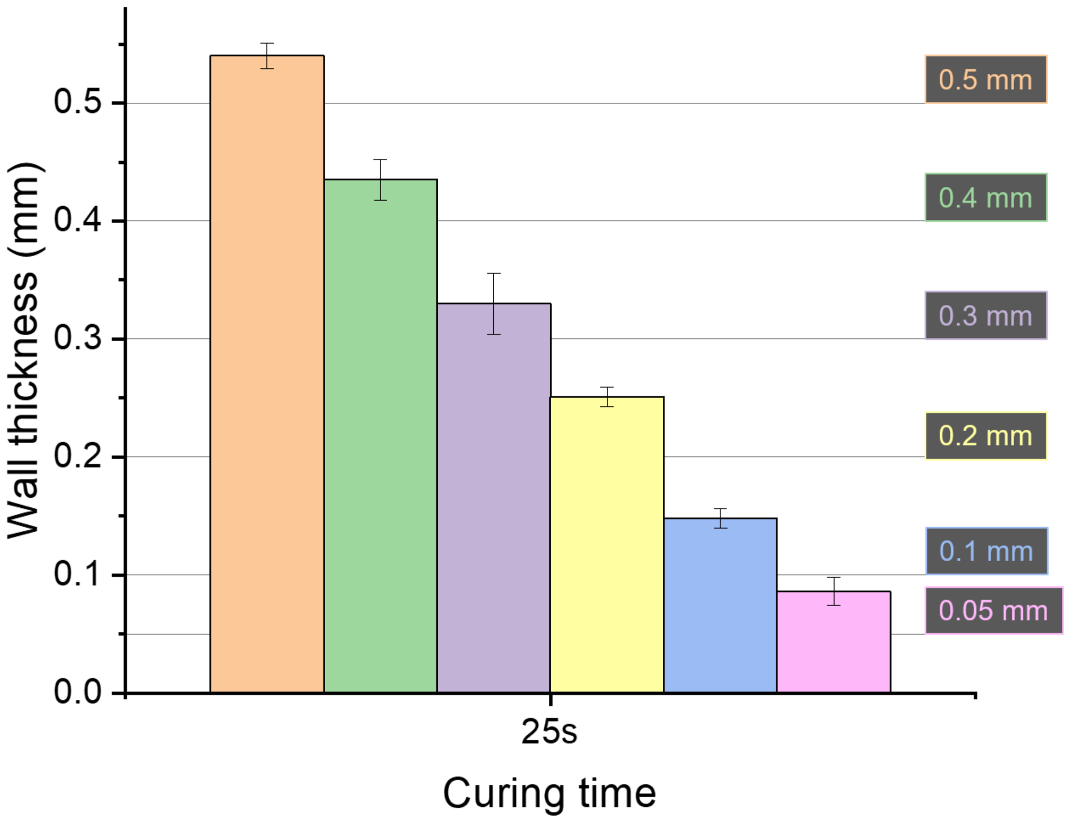

Fig. S157.
X-Y printing accuracy determined by comparing wall thickness (theoretical values indicated by color-coded labels) to curing time for 3D-printed bridges and square arrays using $EtLp_1$:$GlyLp_3$ (30:70 wt.%) **recycled** resin.

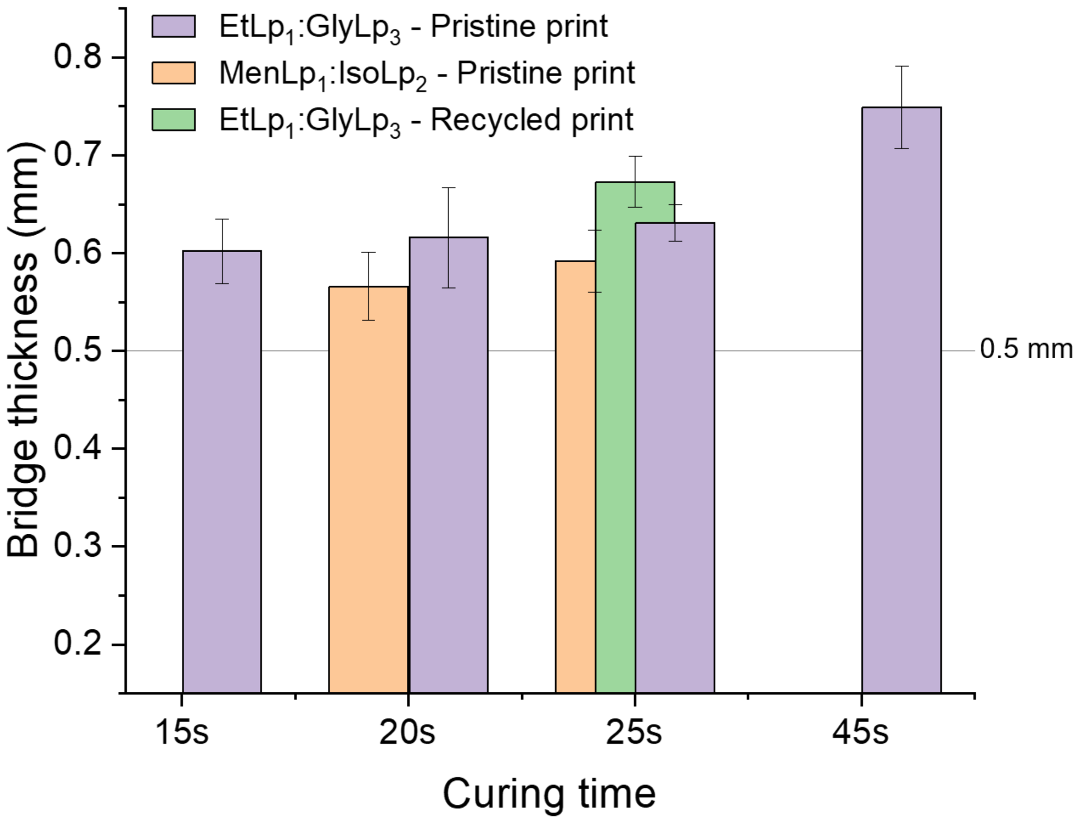

Fig. S158.
X-Y printing accuracy determined by comparing thickness of bridges (theoretical value indicated by horizontal line at 0.5 mm) to curing time for 3D-printed bridges and square arrays using $MenLp_1$:$IsoLp_2$ (30:70 wt.%) **pristine** resin and $EtLp_1$:$GlyLp_3$ (30:70 wt.%), **pristine** & **recycled** resins.

Renders and photographs of printed parts

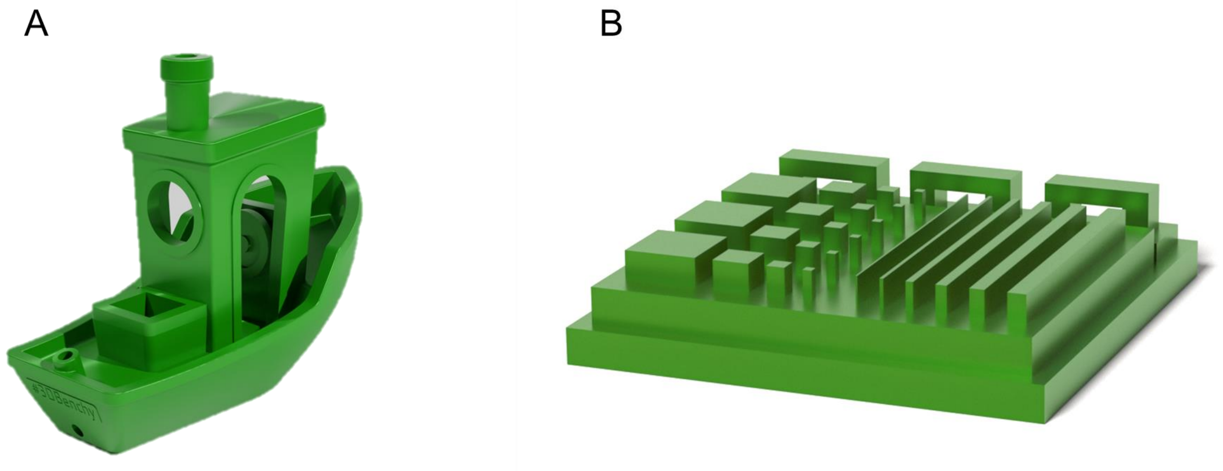

Fig. S159.
3D renders of (A) “3DBenchy” and (B) square arrays and bridges used in precision printing.

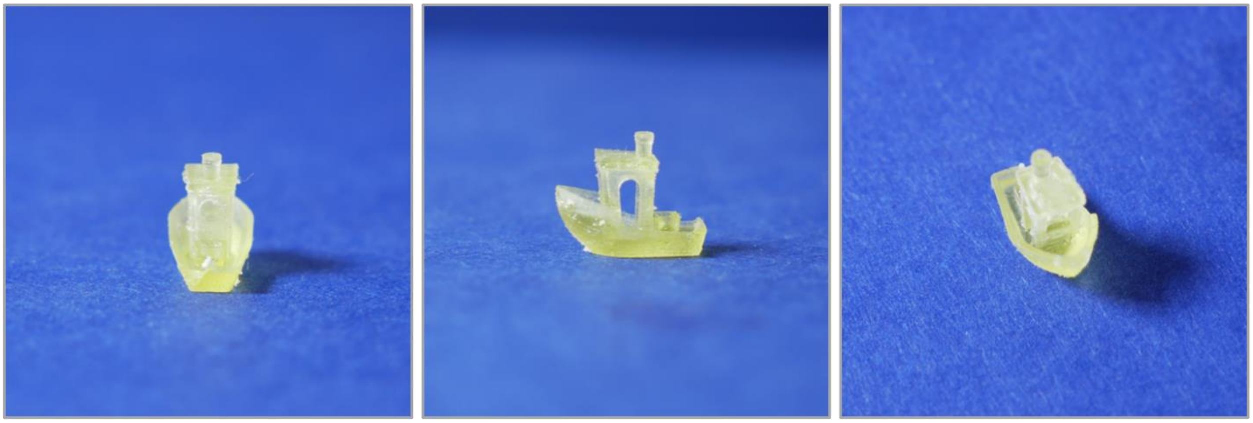

Fig. S160.
Photographs of 3D-printed “3DBenchy” from $MenLp_1$:$IsoLp_2$ (30:70 wt.%) **pristine** resin.

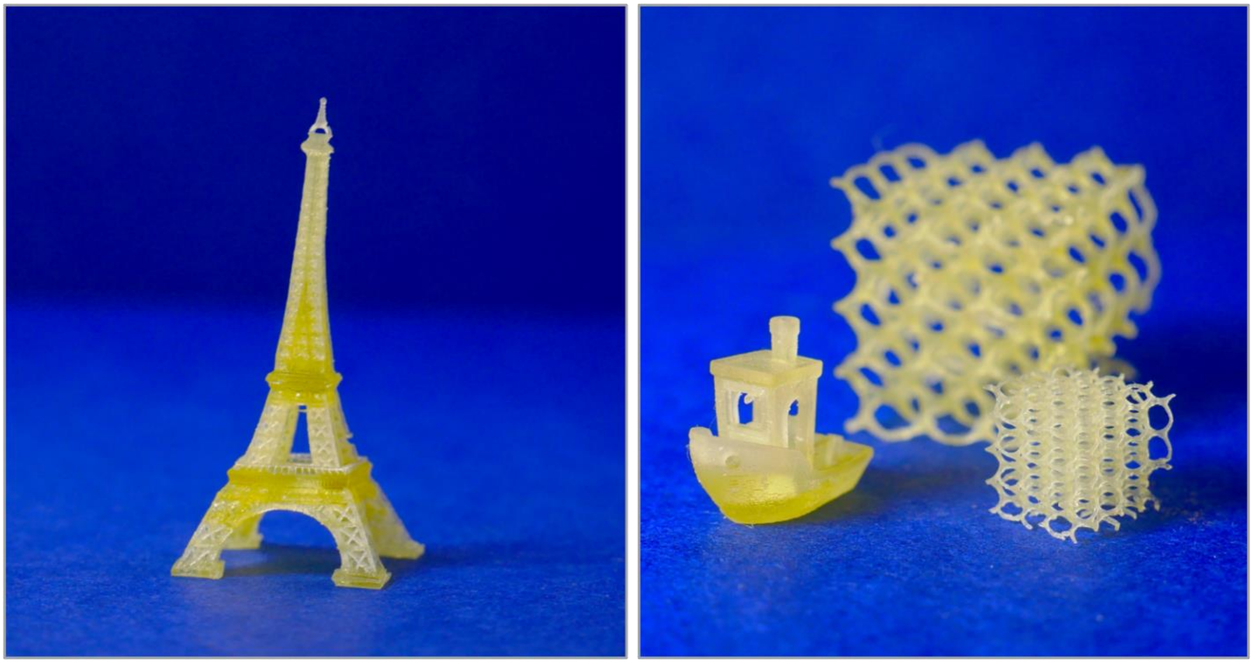

Fig. S161.
Photographs of 3D-printed complex parts from $EtLp_1$:$GlyLp_3$ (30:70 wt.%) **pristine** resin.

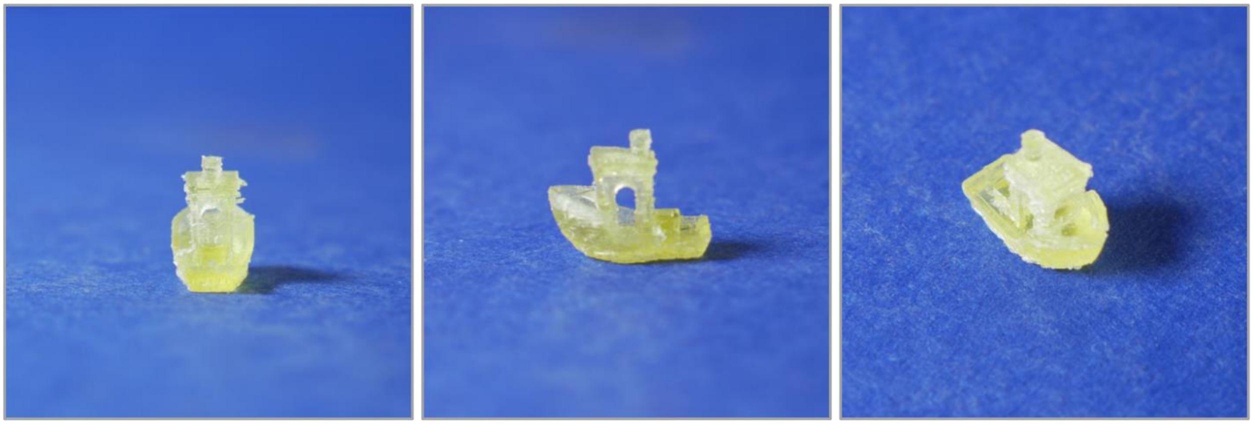

Fig. S162.
Photographs of 3D-printed “3DBenchy” from $EtLp_1$:$GlyLp_3$ (30:70 wt.%) **recycled** resin.

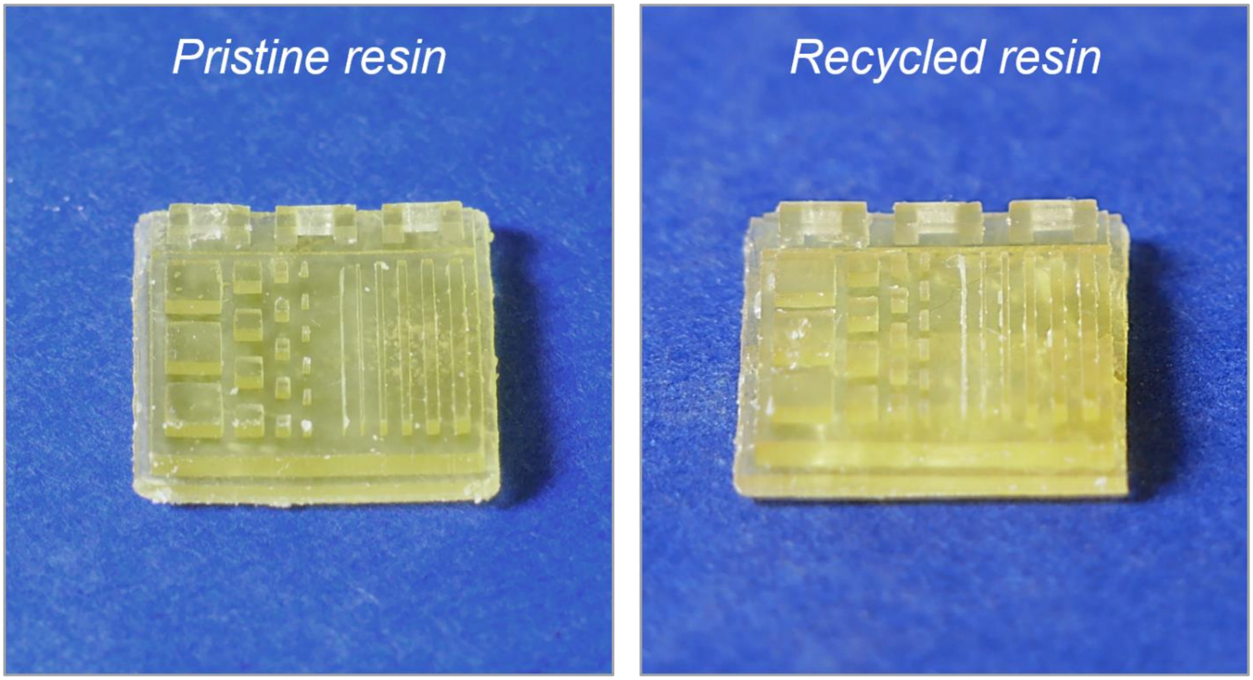

Fig. S163.
Photographs comparing 3D-prints of square arrays and bridges from $EtLp_1$:$GlyLp_3$ (30:70 wt.%) **pristine** vs **recycled** resins.

Mechanical properties of 3D prints

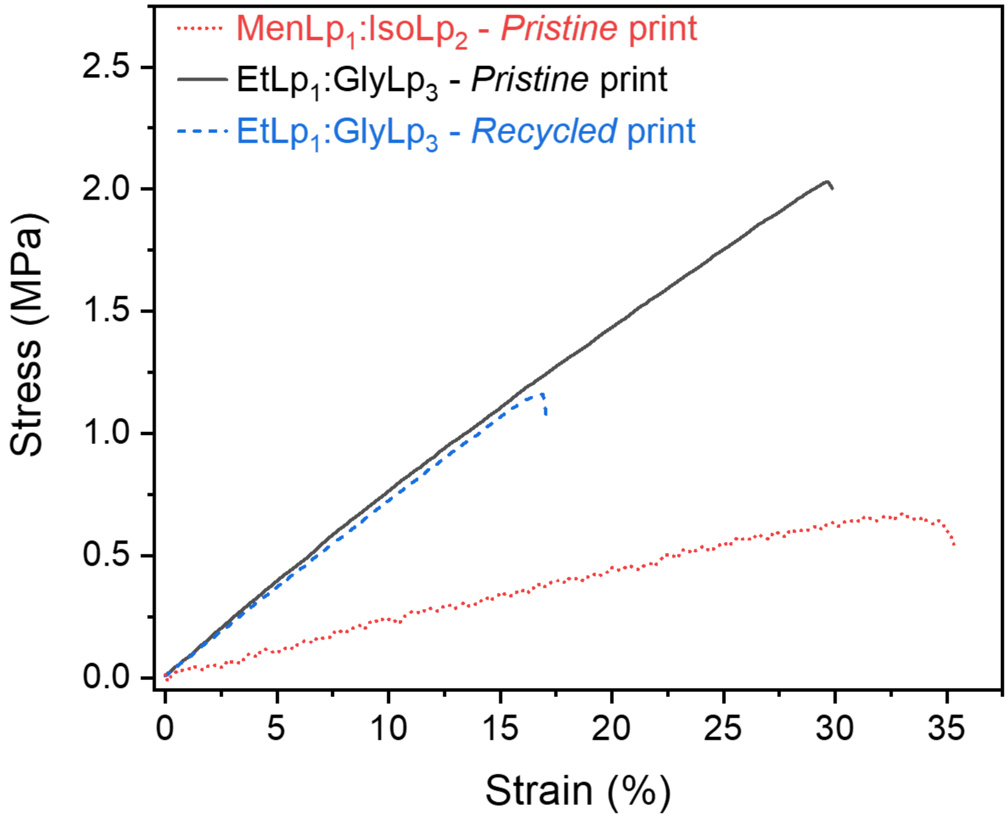

Fig. S164.
Representative stress *vs* strain curves of 3D-printed dog-bones for $MenLp_1$:$IsoLp_2$ (30:70 wt%) & $EtLp_1$:$GlyLp_3$ (30:70 wt%) as **pristine** prints and $EtLp_1$:$GlyLp_3$ (30:70 wt%) as a **recycled print** obtained from catalyzed depolymerization (phosphazene:thiophenol) method of 3D-printed pristine parts. Tested at 10 mm min^{-1} strain rate.

Recycling and re-curing/printing resins using thermal depolymerization (DMF, 140 °C) method

Photorheology data

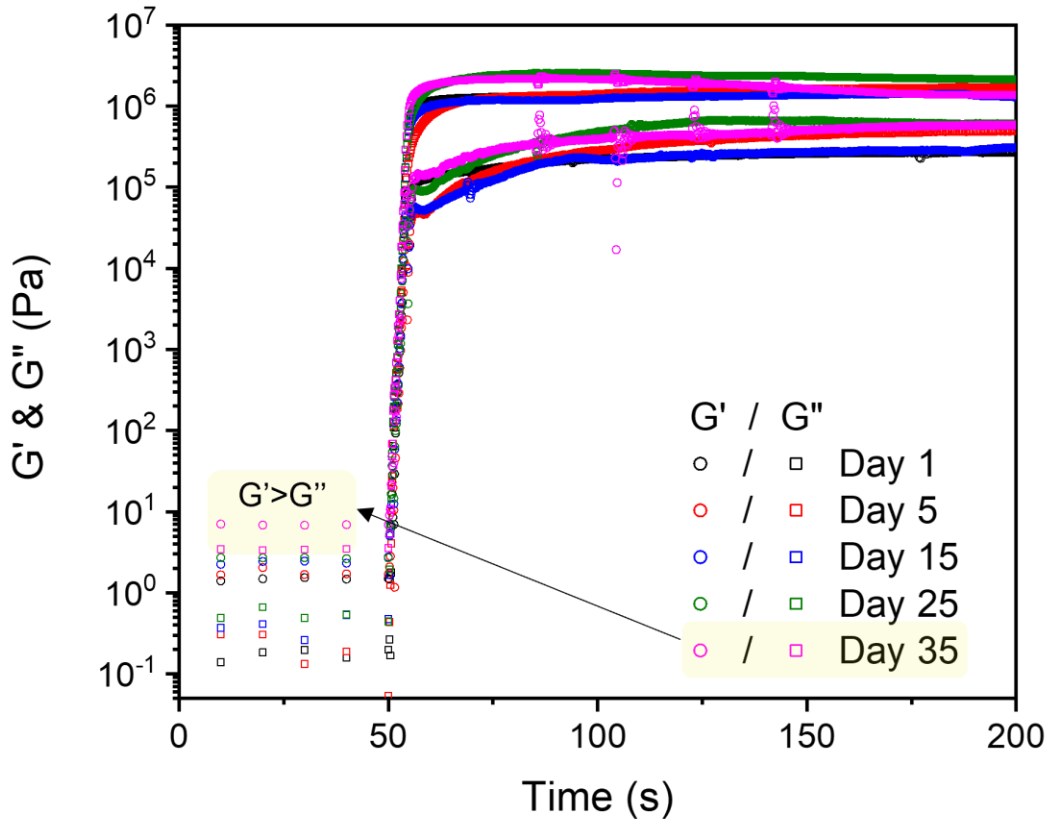

Fig. S165.
Photorheology of $EtLp_1$:$GlyLp_3$ (31:69 wt%) **pristine** resin assessed periodically over 35 days. Resin was kept at ambient temperature (21-23 °C) and protected from light for the duration of the study. Day 35 sample data (yellow highlight box) indicates that the resin was beyond the gel point (G '> G'') before photocuring commenced.

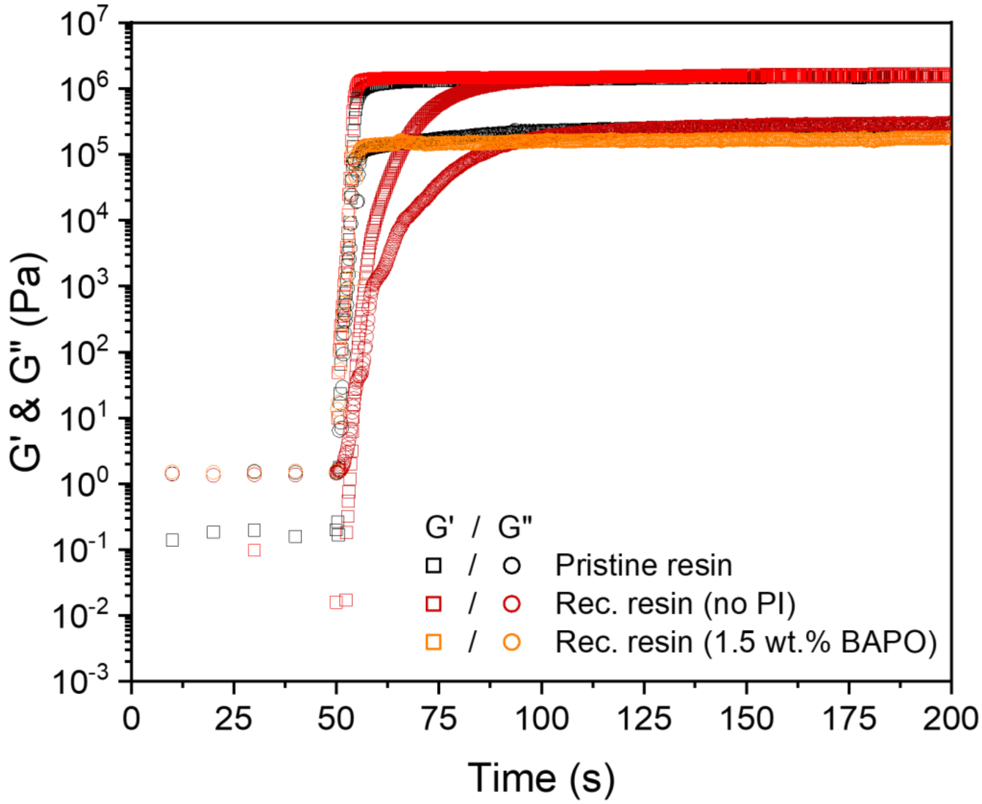

Fig. S166.
Photorheology of EtLp1:GlyLp3 (31:69 wt%) pristine resin (black) and **1st recycle** resin with and without photoinitiator (BAPO) taken over 230 s under oscillatory shear at ambient temperature.

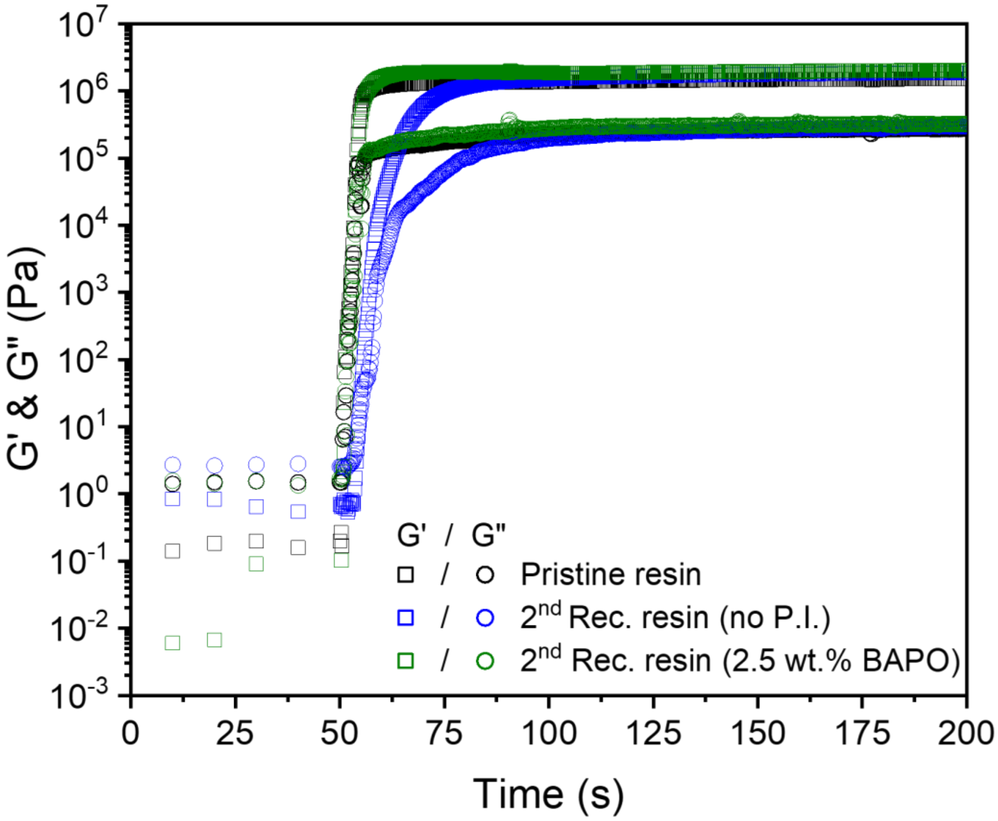

Fig. S167.
Photorheology of $EtLp_1$:$GlyLp_3$ (31:69 wt%) pristine resin (black) and **2nd recycle** resin with and without photoinitiator (BAPO) taken over 230 s under oscillatory shear at ambient temperature.

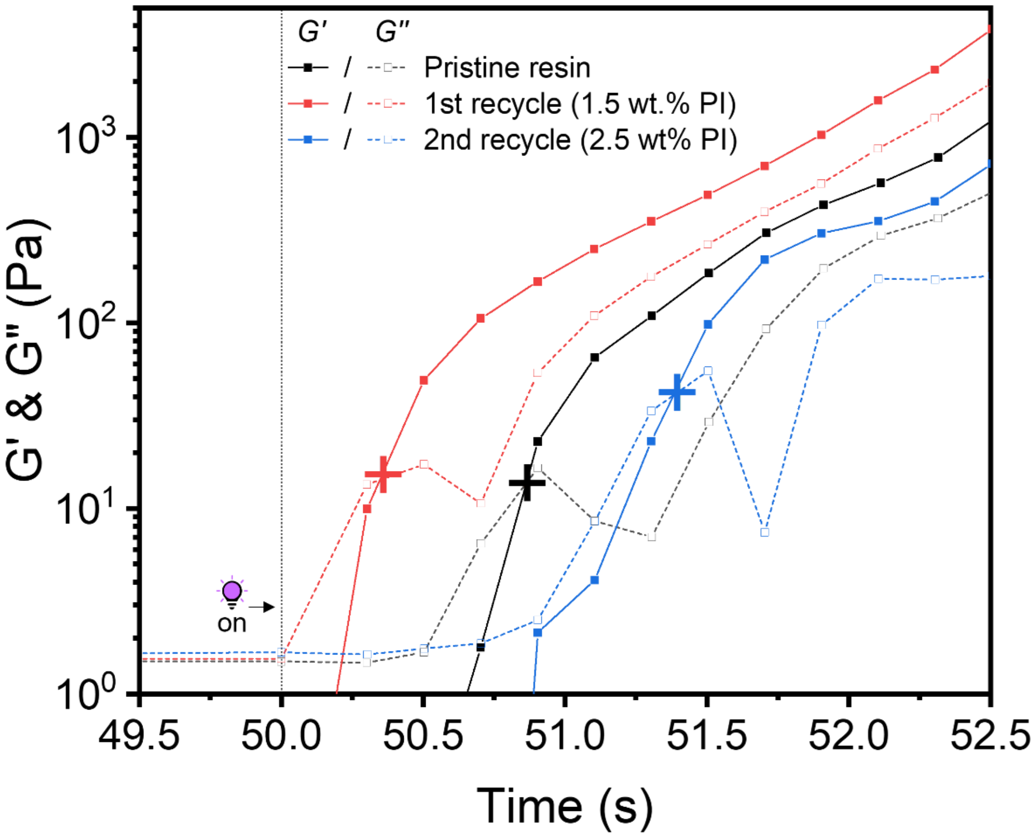

Fig. S168.
Photorheology of EtLp1:GlyLp3 (31:69 wt%) **pristine** (black), **1st recycle** (red) and **2nd recycle** (blue) resins taken over 230 s under oscillatory shear at ambient temperature. Inset zoom of plot from Fig. 4C to emphasize gel point (G’ > G’’) as indicated by “+” symbols.

3D-printing

Z-axis curing depth

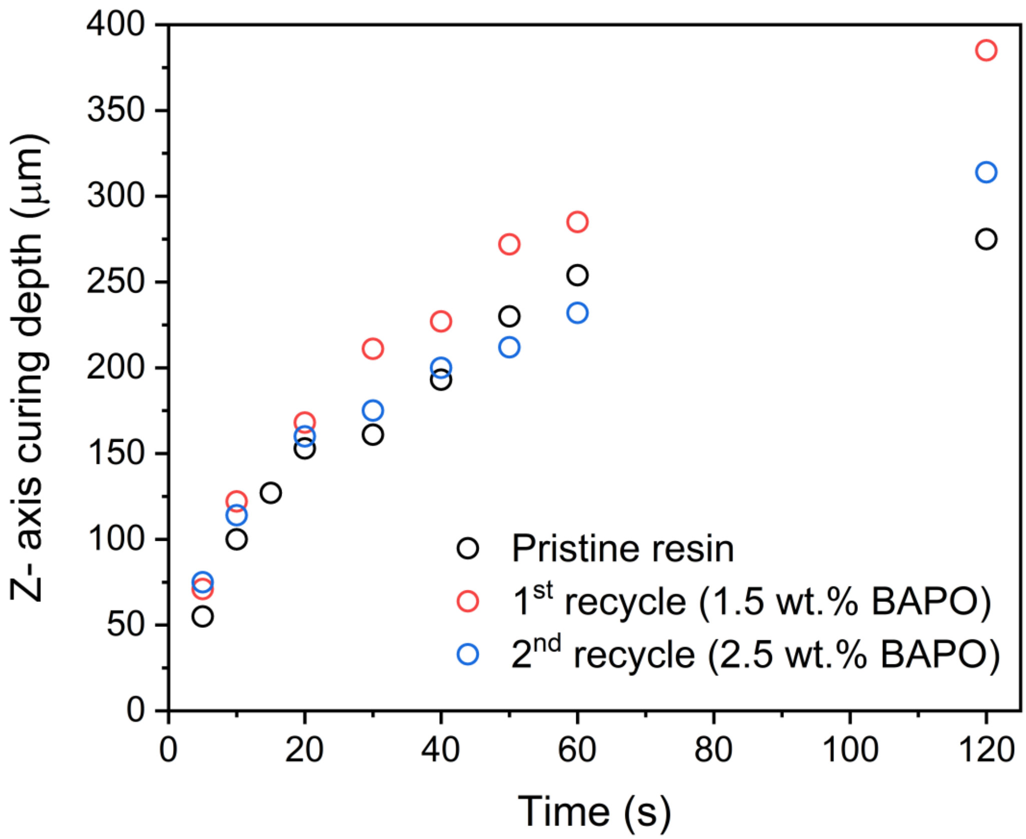

Fig. S169.
Z-depth cure screening for $EtLp_1$:$GlyLp_3$ (31:69 wt.%) comparing **pristine, 1st recycle, 2nd recycle** resins by irradiating a 2D-square and measuring sample thickness (Z-axis depth) vs irradiation time over 120 s.

3D-printing of square arrays and bridges

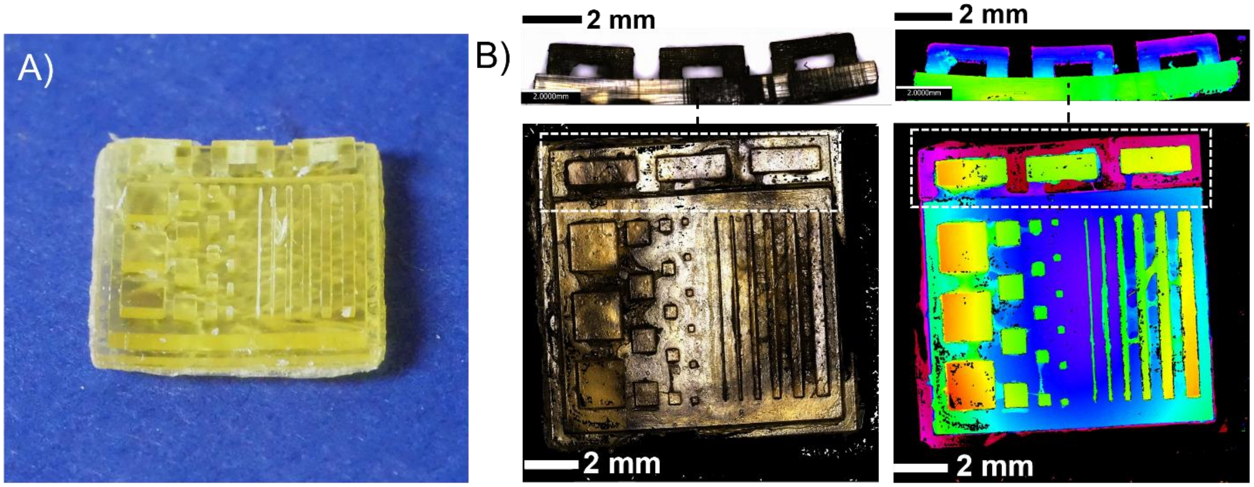

Fig. S170.
3D-printed bridges and square arrays at various cure times (s/layer) using $EtLp_1$:$GlyLp_3$ (31:69 wt.%) **1st recycle** resin (A) photographs of prints and (B) corresponding image analysis. Cure time = 20 s/layer.

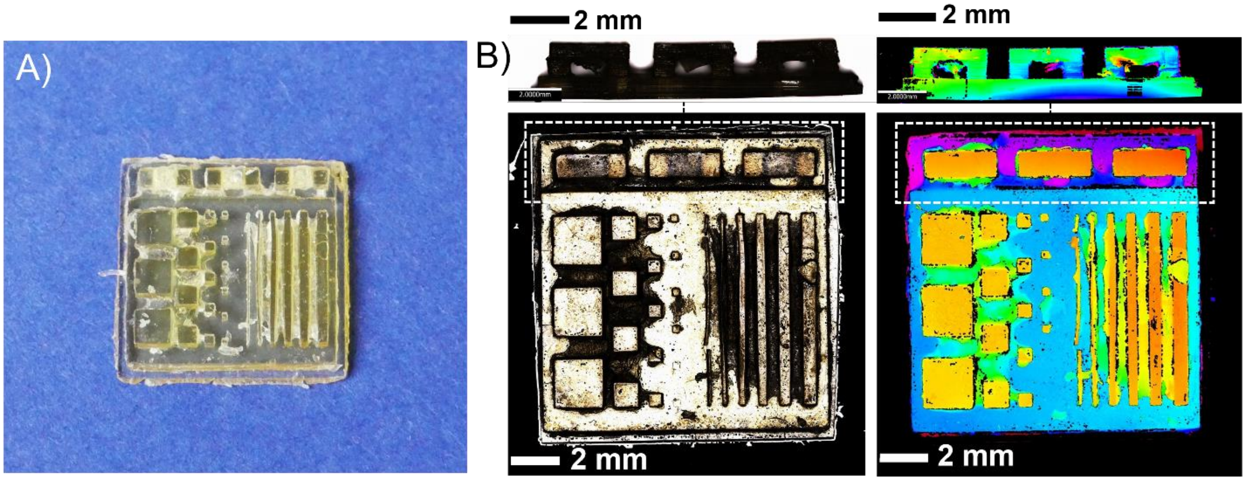

Fig. S171.
3D-printed bridges and square arrays at various cure times (s/layer) using $EtLp_1$:$GlyLp_3$ (31:69 wt.%) **2nd recycle** resin (A) photographs of prints and (B) corresponding image analysis. Cure time = 35 s/layer.

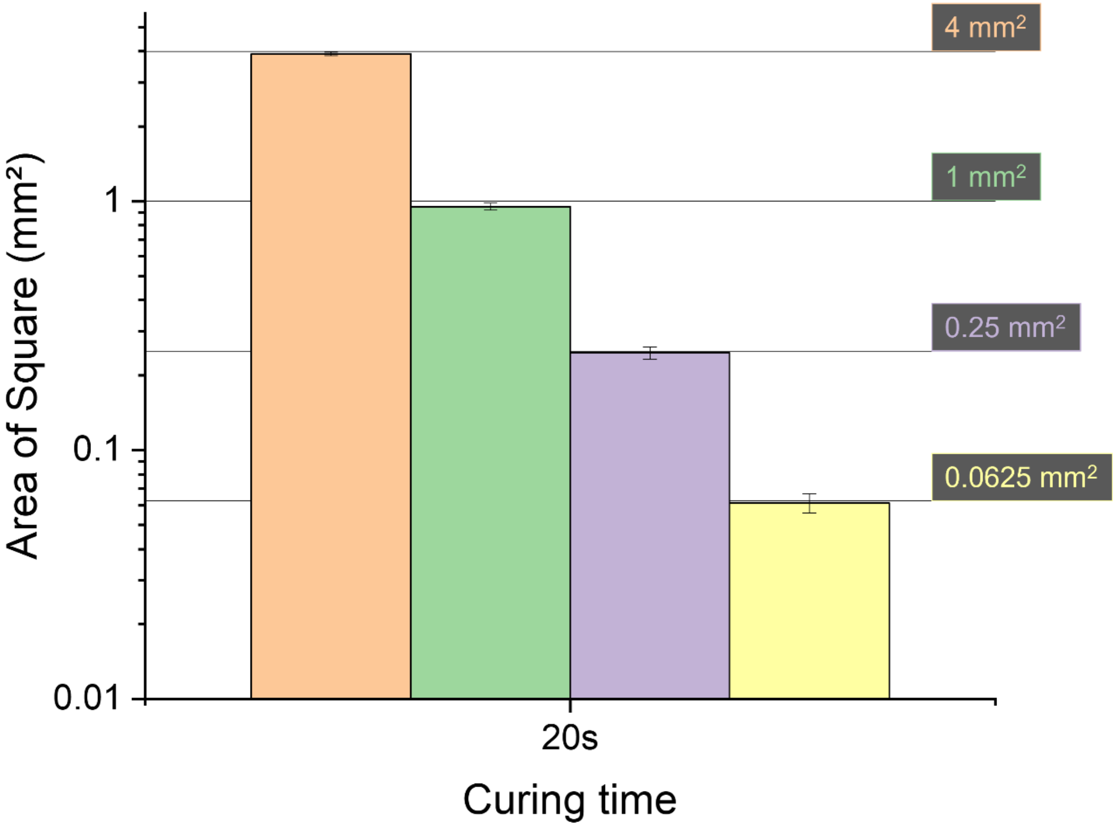

Fig. S172.
X-Y printing accuracy determined by comparing surface area of squares (theoretical values indicated by color-coded labels) to curing time for 3D-printed bridges and square arrays using $EtLp_1$:$GlyLp_3$ (31:69 wt.%) **1st recycle** resin. Cure time = 20 s/layer.

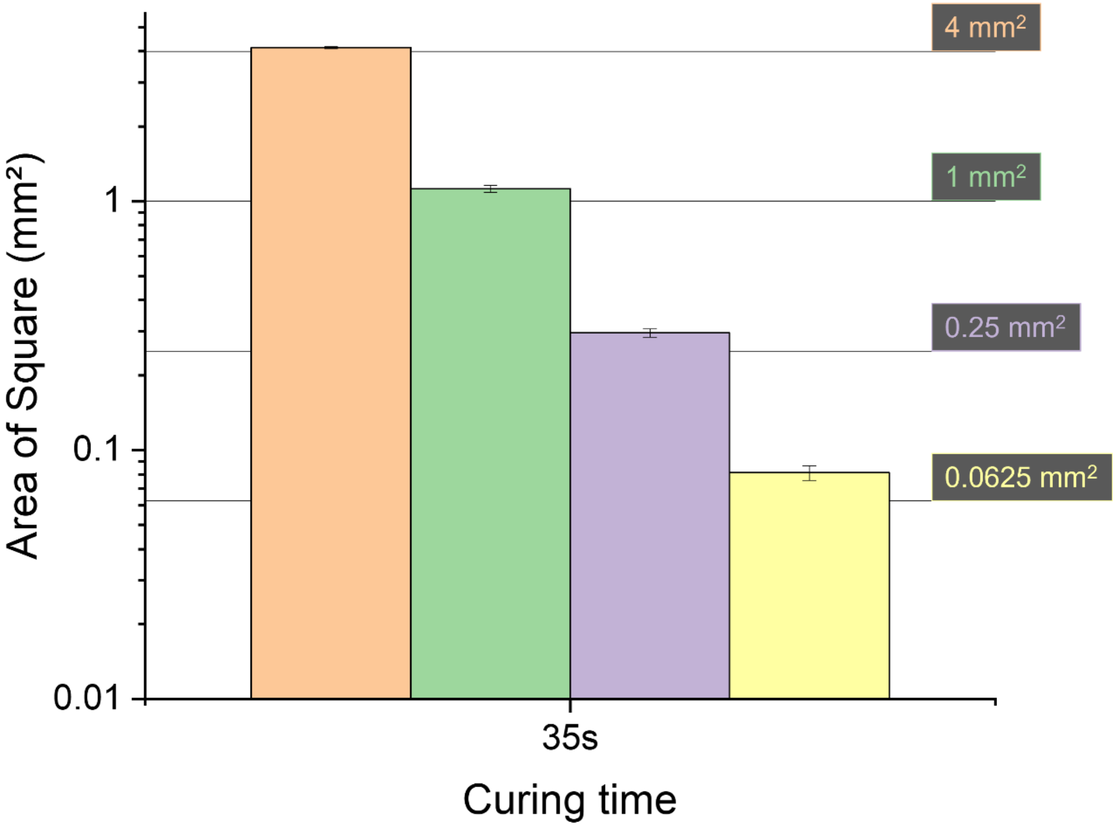

Fig. S173.
X-Y printing accuracy determined by comparing surface area of squares (theoretical values indicated by color-coded labels) to curing time for 3D-printed bridges and square arrays using $EtLp_1$:$GlyLp_3$ (31:69 wt.%) **2nd recycle** resin. Cure time = 35 s/layer.

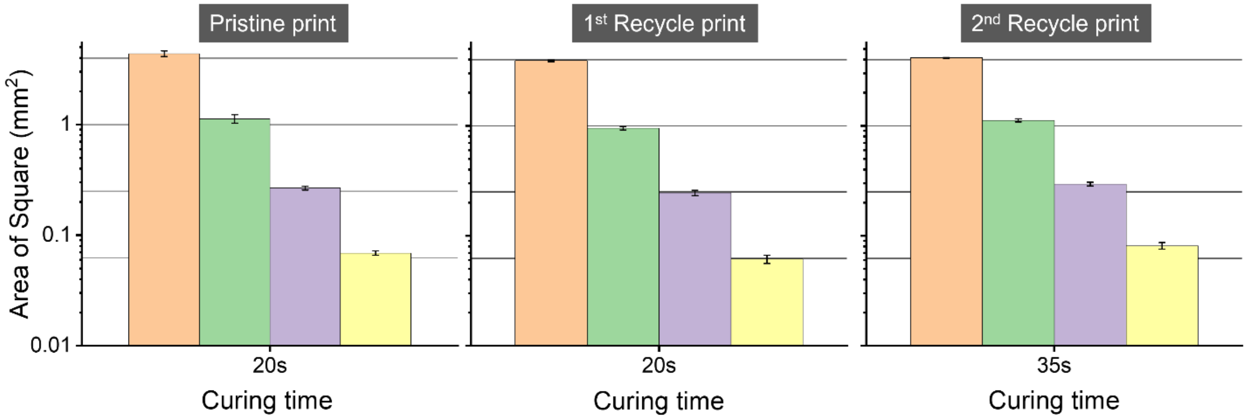

Fig. S174.
X-Y printing accuracy determined by comparing surface area of squares to curing time for 3D-printed bridges and square arrays using $EtLp_1$:$GlyLp_3$ (31:69 wt.%) comparing **pristine, 1st recycle, 2nd recycle** resins. Cure time dependent upon resin formulation (indicated in plot).

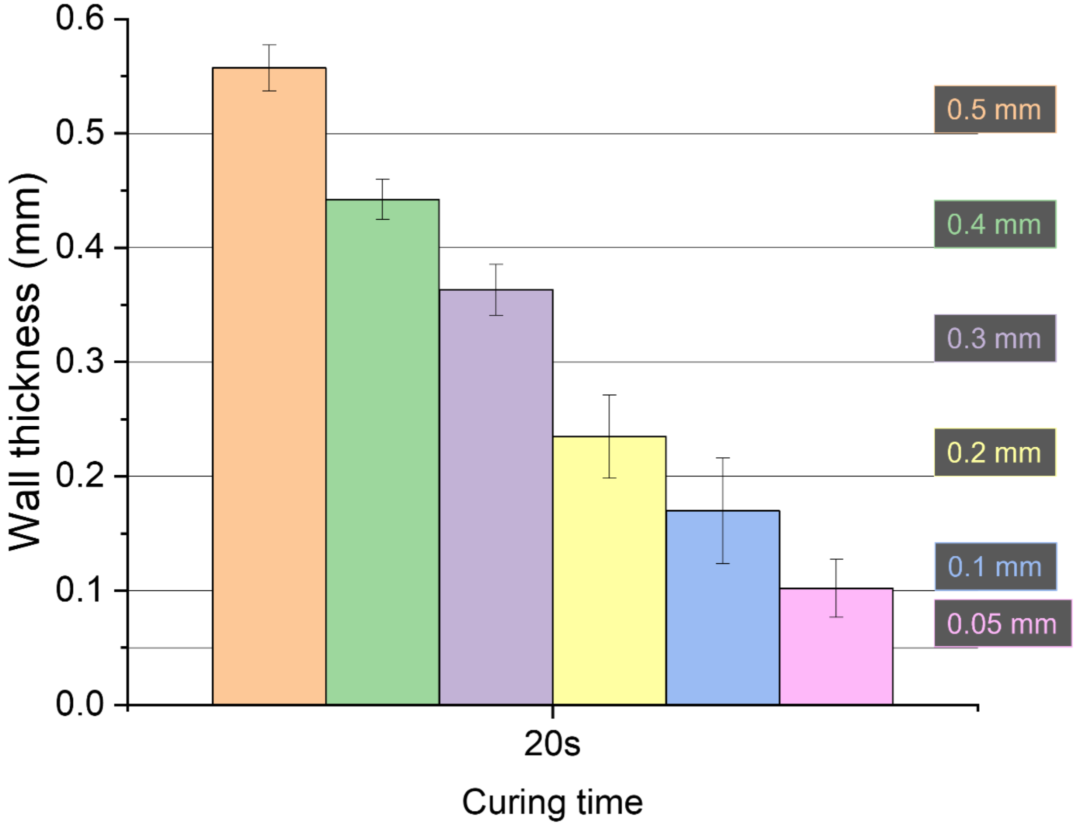

Fig. S175.
X-Y printing accuracy determined by comparing wall thickness (theoretical values indicated by color-coded labels) to curing time for 3D-printed bridges and square arrays using $EtLp_1:GlyLp_3$ (31:69 wt.%) **1st recycle** resin.

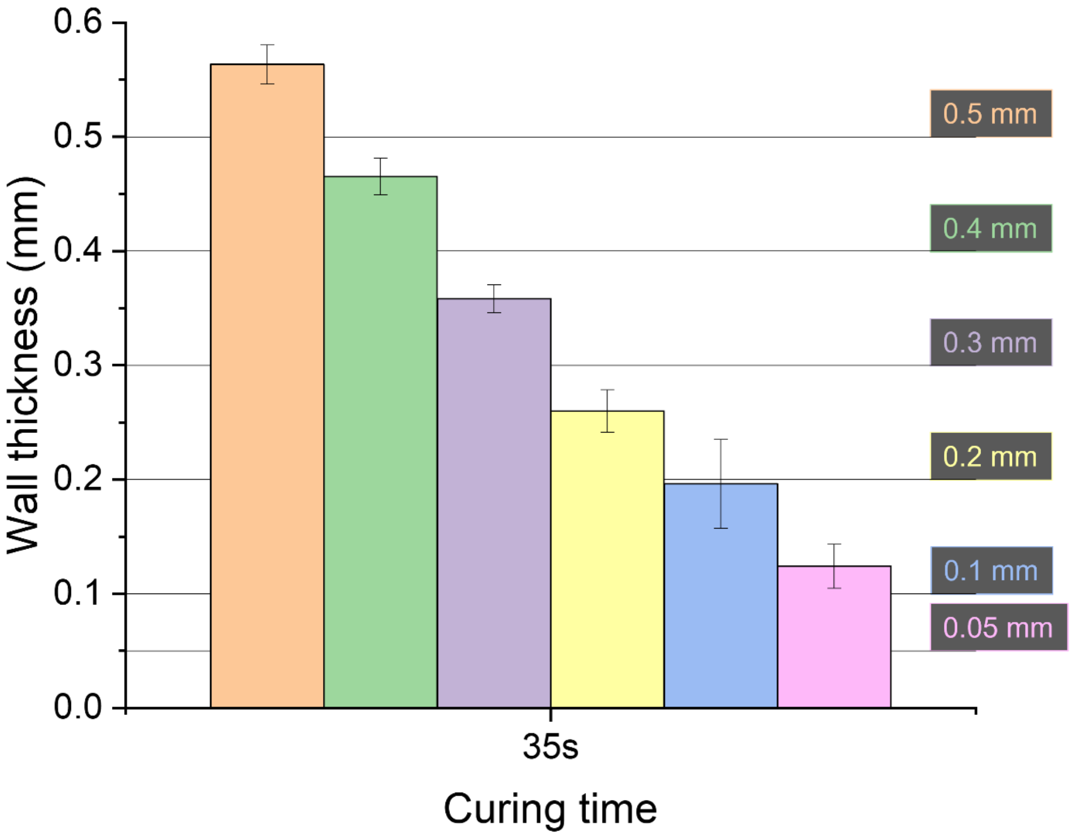

Fig. S176.
X-Y printing accuracy determined by comparing wall thickness (theoretical values indicated by color-coded labels) to curing time for 3D-printed bridges and square arrays using $EtLp_1:GlyLp_3$ (31:69 wt.%) **2nd recycle** resin.

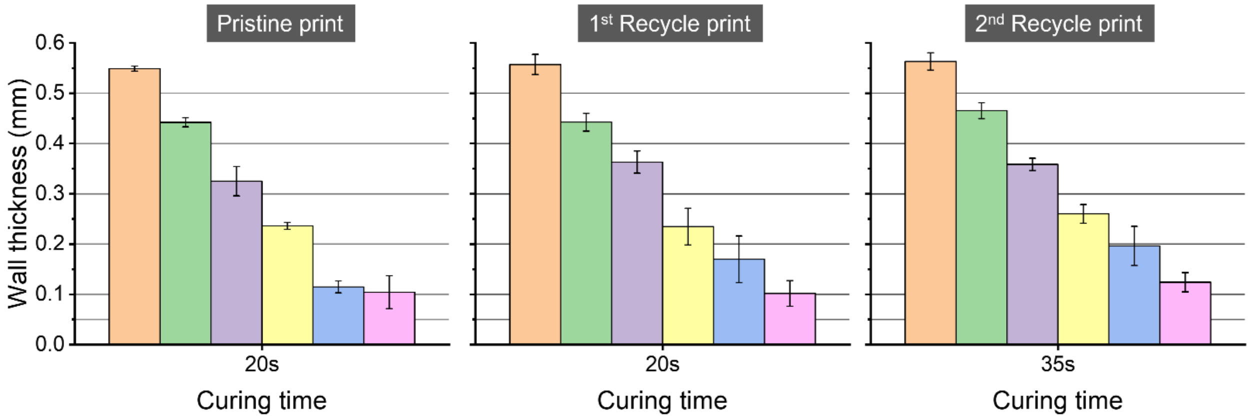

Fig. S177.
X-Y printing accuracy determined by comparing wall thickness to curing time for 3D-printed bridges and square arrays using $EtLp_1$:$GlyLp_3$ (31:69 wt.%) comparing **pristine, 1st recycle, 2nd recycle** resins. Cure time dependent upon resin formulation (indicated in plot).

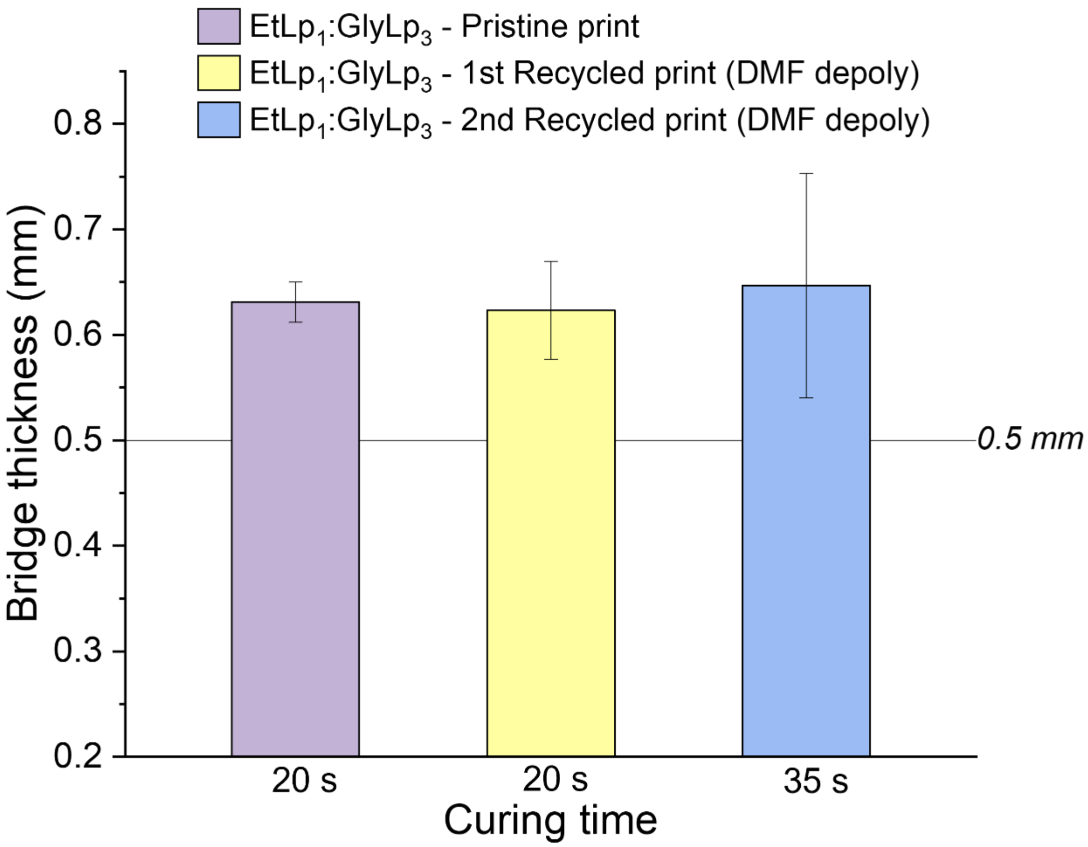

Fig. S178.
X-Y printing accuracy determined by comparing thickness of bridges (theoretical value indicated by horizontal line at 0.5 mm) to curing time for 3D-printed bridges and square arrays using $EtLp_1$:$GlyLp_3$ (31:69 wt.%) comparing **pristine, 1st recycle, 2nd recycle** resins. Cure time dependent upon resin formulation (indicated in plot).

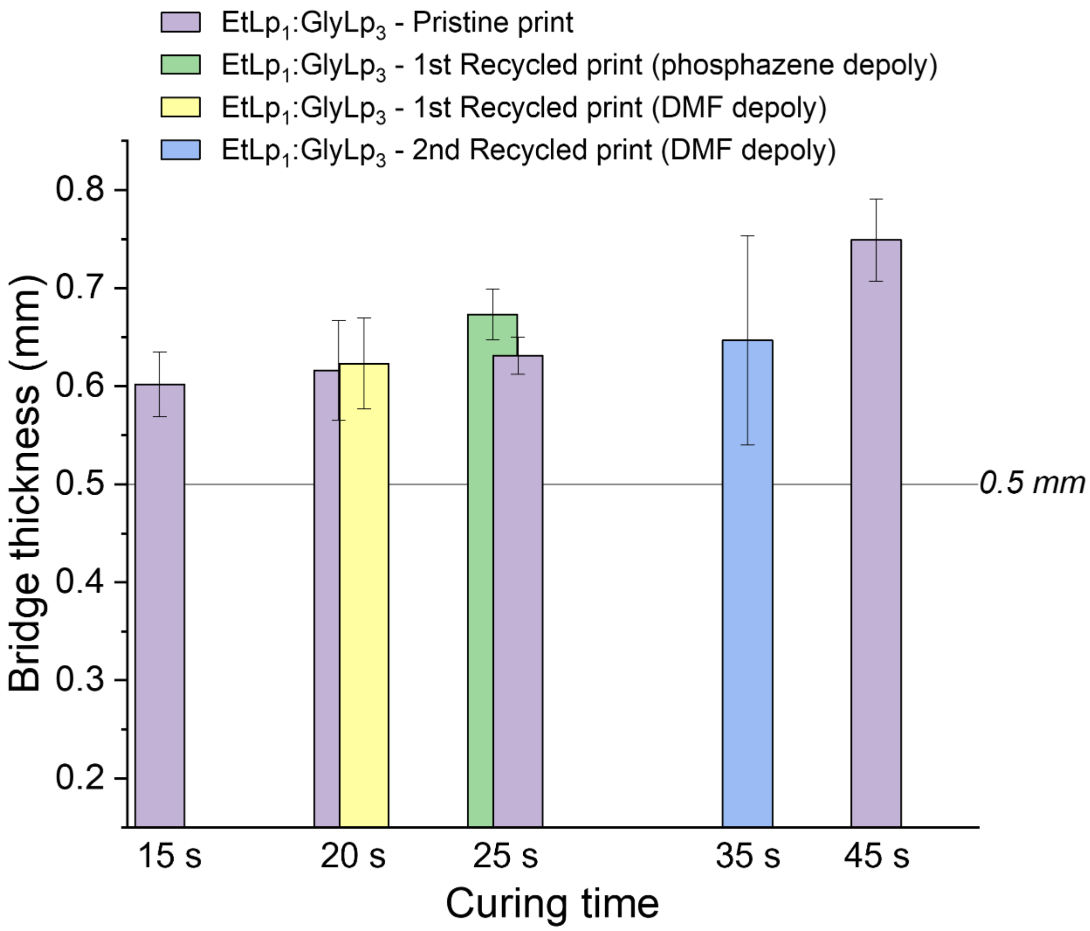

Fig. S179.
X-Y printing accuracy determined by comparing thickness of bridges (theoretical value indicated by horizontal line at 0.5 mm) to curing time for 3D-printed bridges and square arrays using $EtLp_1$:$GlyLp_3$ (31:69 wt.%) comparing **pristine, 1st recycle, 2nd recycle** resins (including 1st recycle from catalyzed depolymerization method). Cure time dependent upon resin formulation (indicated in plot).

Photographs of printed parts

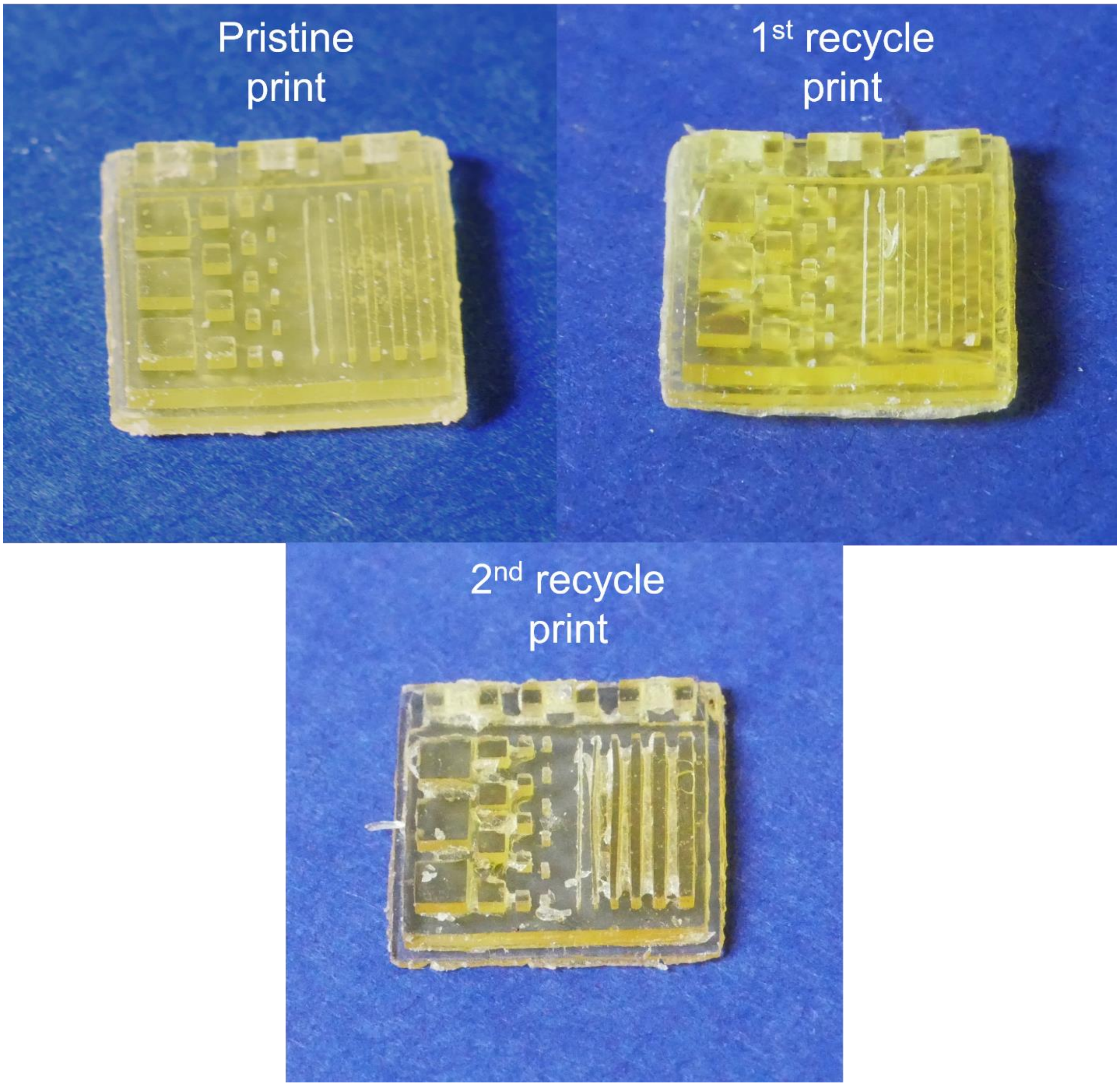

Fig. S180.
Photographs of 3D-printed square arrays and bridges from EtLp1:GlyLp3 (31:69 wt.%) comparing **pristine, 1st recycle, 2nd recycle** resins.

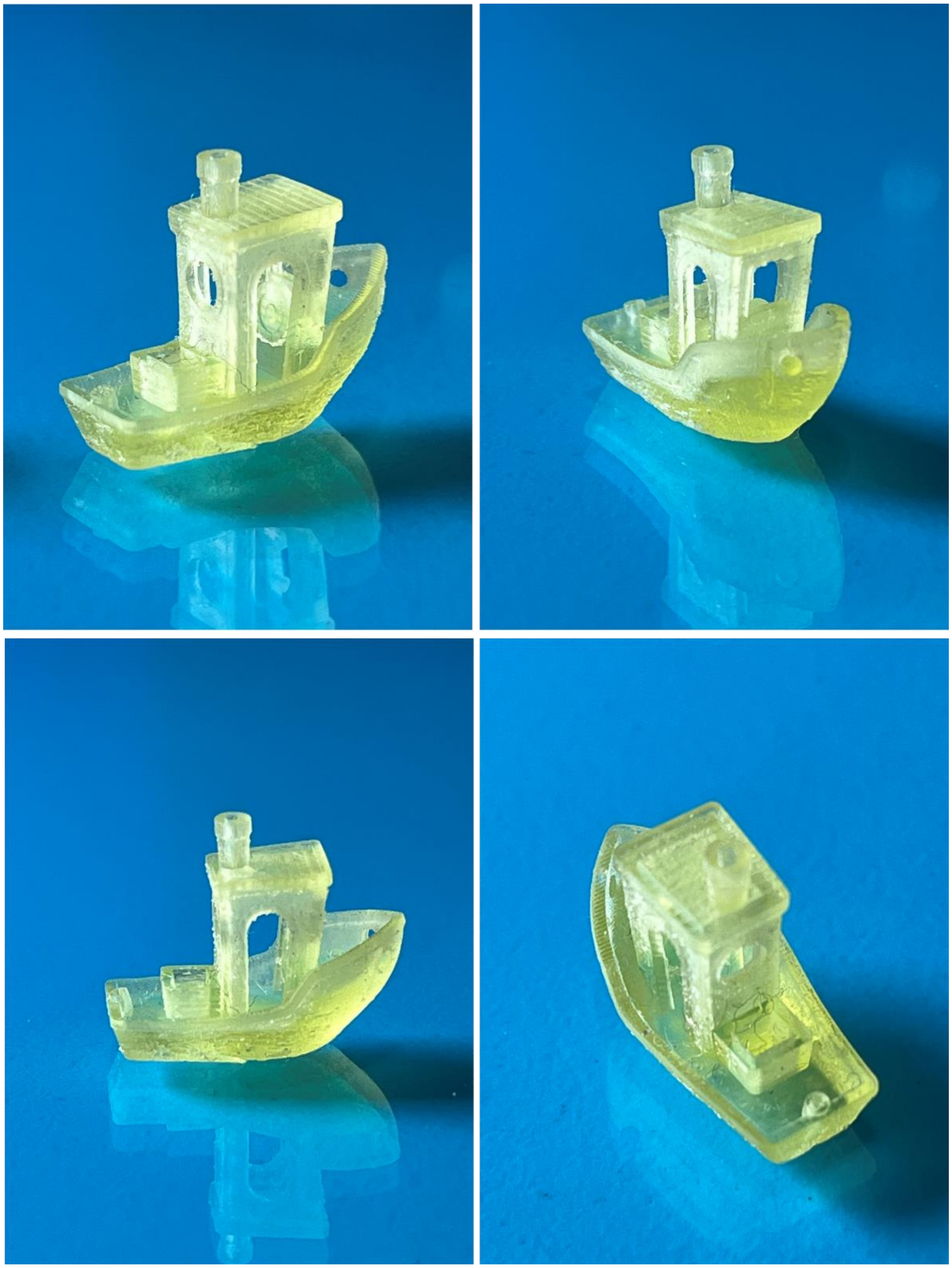

Fig. S181.
Photographs of large (10 mm width) 3D-printed “3DBenchy” from $EtLp_1$:$GlyLp_3$ (31:69 wt.%) **pristine** resin.

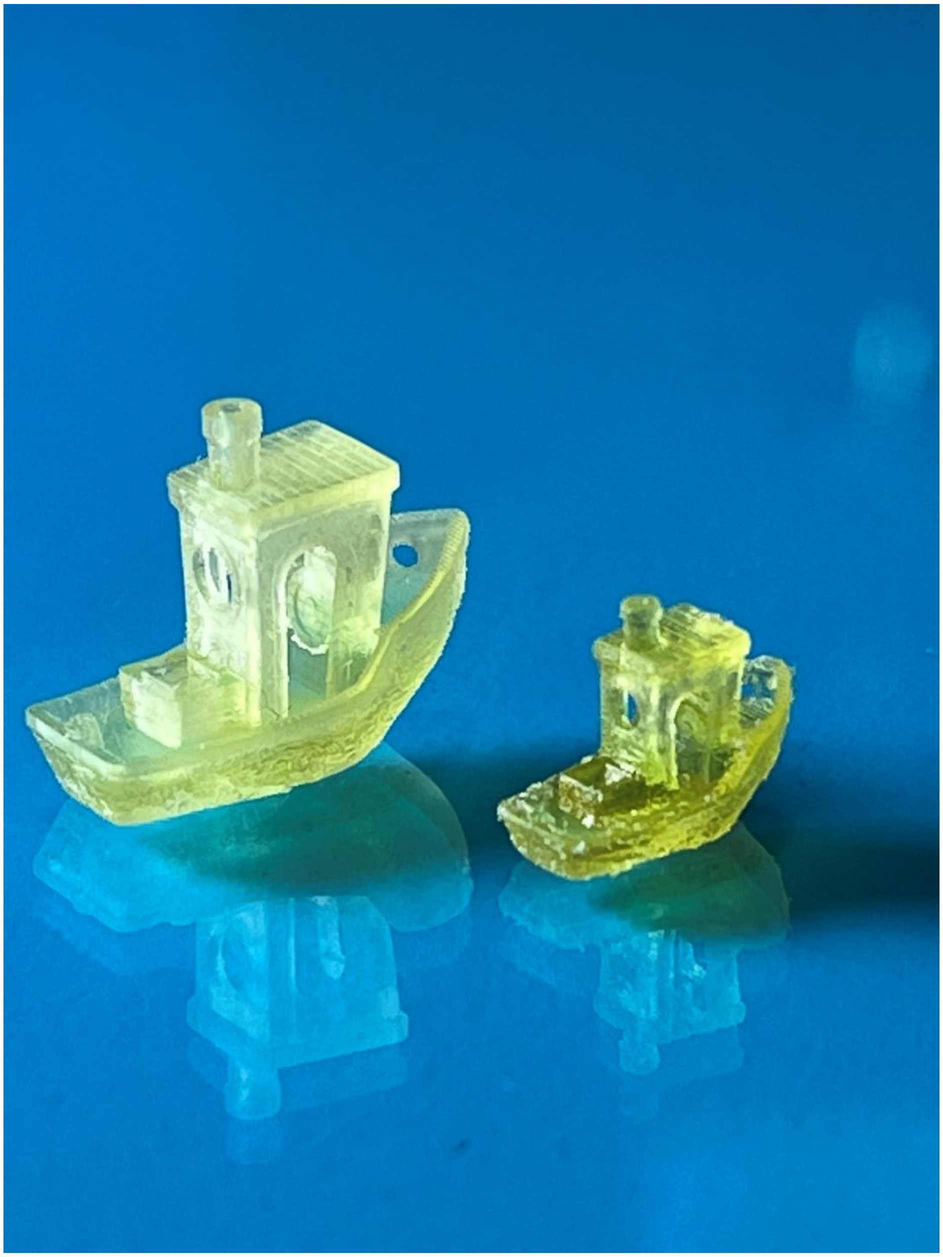

Fig. S182.
Photographs of large (10 mm width) and small (10 mm length) 3D-printed “3DBenchy” from $EtLp_1$:$GlyLp_3$ (31:69 wt.%) **pristine** resin.

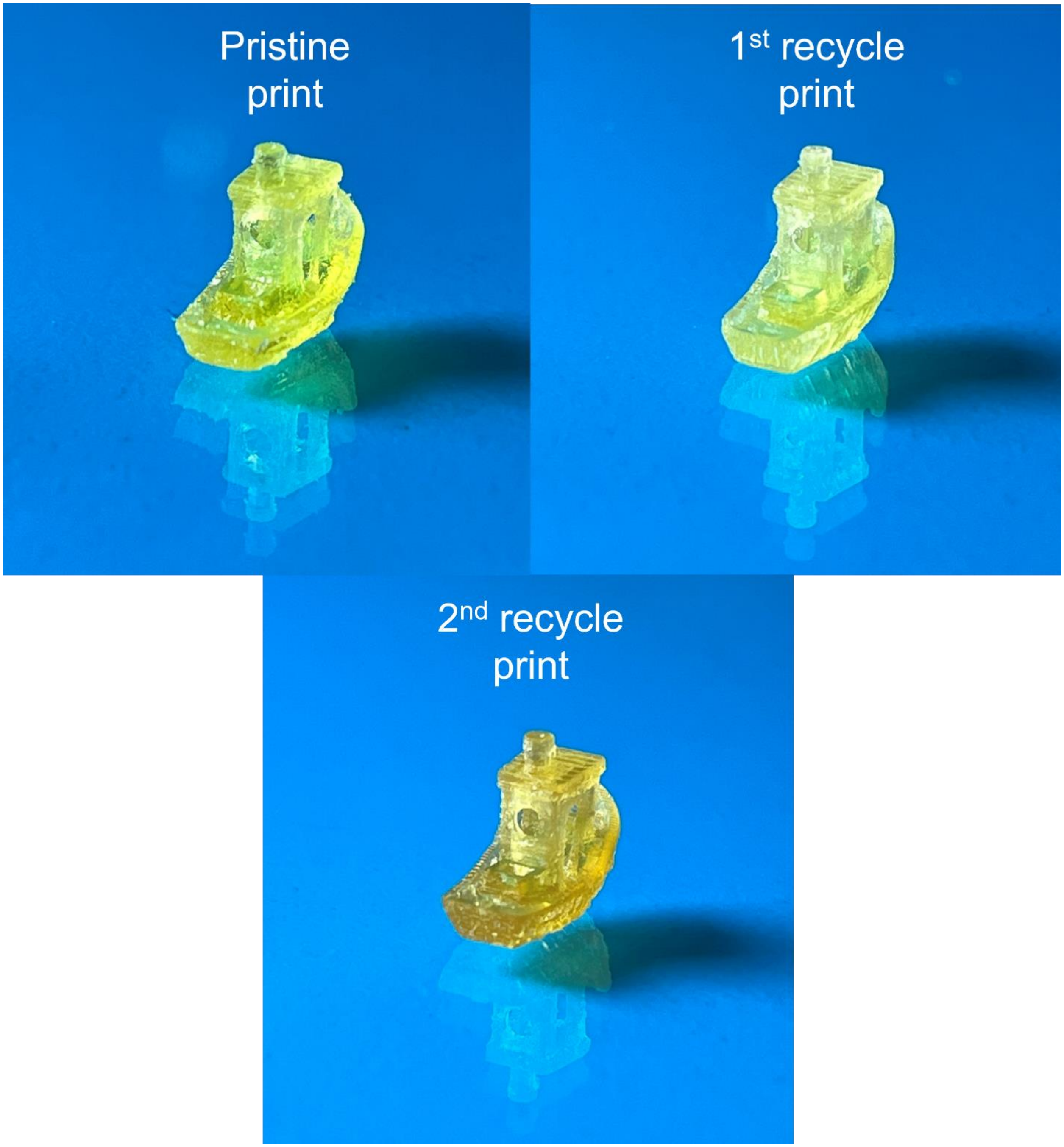

Fig. S183.
Photographs of small (10 mm width) 3D-printed "3DBenchy" from EtLp$_1$:GlyLp$_3$ (31:69 wt.%) comparing **pristine, 1st recycle, 2nd recycle** resins. Different angles of prints shown as compared to Fig. 4F.

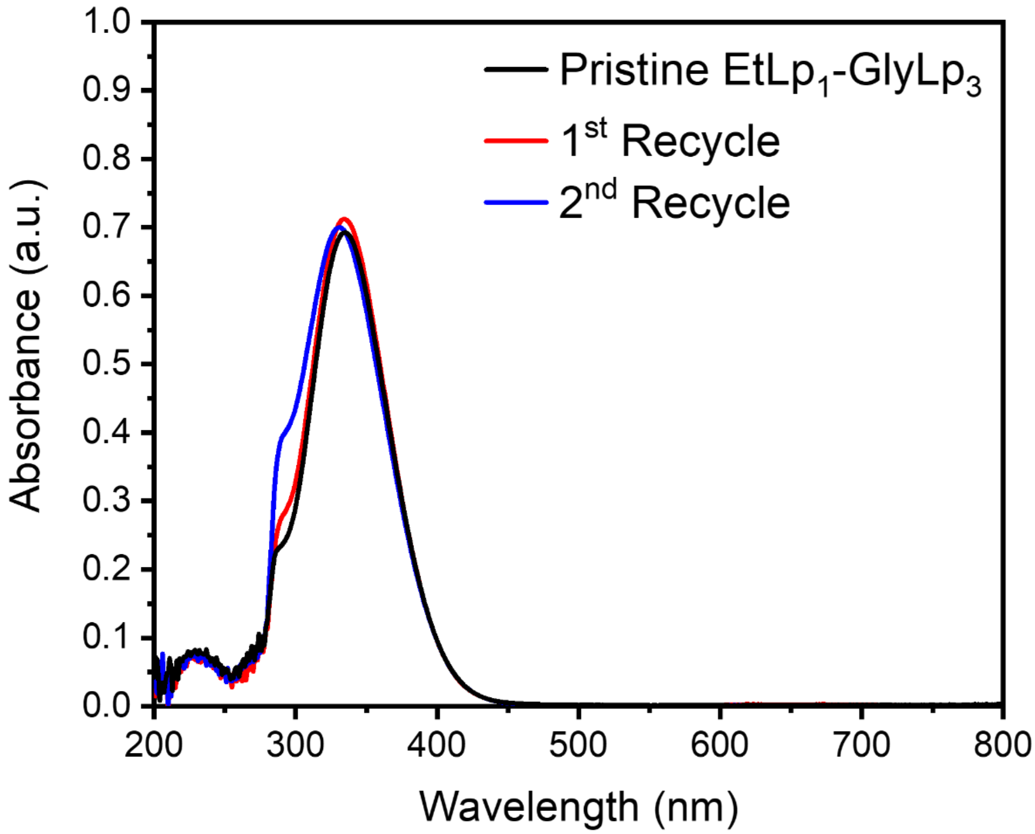

Fig. S184.
UV-Vis spectra of $EtLp_1$:$GlyLp_3$ resin (1 mg/mL, CH_2Cl_2) comparing **pristine, 1st recycle, 2nd recycle** resins.

Irradiance of light sources

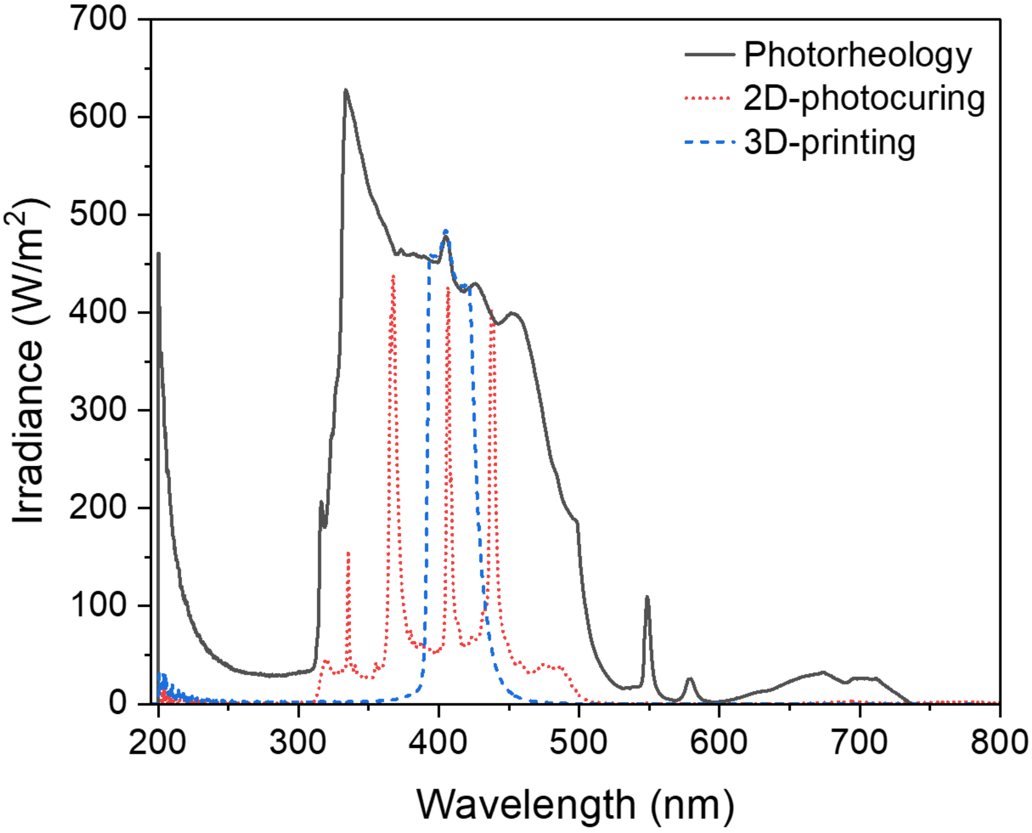

Fig. S185.
Irradiance *vs* wavelength for various light sources used for photopolymerization.

Table S5.
Summary of thermomechanical data for materials produced from all synthesized lipoate formulations.

	Resin	Formulation (wt:wt)	$T_{d,5\%}$ (°C)	T_g (°C)	E (MPa)	UTS (MPa)	ε_{break} (%)	Toughness (kJ/m^3)
Post-cured 2D-photosets	MenLp$_1$:IsoLp$_2$	90:10	-	− 31	-	-	-	-
		70:30	-	− 7	1.4 ± 0.2	0.9 ± 0.1	70 ± 4	321 ± 39
		50:50	-	2	37.6 ± 6.0	3.0 ± 0.3	61 ± 3	1070 ± 132
		30:70	221	13	194 ± 24	8.9 ± 0.7	66 ± 4	4076 ± 417
		10:90	-	20	330 ± 20	15.6 ± 0.9	22 ± 3	2811 ± 593
	EtLp$_1$:IsoLp$_2$	30:70	188	− 3	8.7 ± 0.6	2.0 ± 0.2	31 ± 5	348 ± 88
	GuaLp$_1$:IsoLp$_2$	30:70	243	5	115 ± 33	5.9 ± 1.2	52 ± 13	2264 ± 1232
	SteaLp$_1$:IsoLp$_2$	30:70	267	[a]23	9.4 ± 1.1	1.7 ± 0.2	27 ± 3	249 ± 43
	GlyLp$_3$:IsoLp$_2$	30:70	264	16	288 ± 56	14.0 ± 2.5	14 ± 1	1444 ± 126
	MenLp$_1$:GlyLp$_3$	30:70	222	10	84.6 ± 19.3	6.1 ± 1.3	36 ± 5	1393 ± 456
	EtLp$_1$:GlyLp$_3$	90:10	-	− 58	-	-	-	-
		70:30	-	− 46	-	-	-	-
		50:50	-	− 32	9.4 ± 1.7	0.7 ± 0.1	9 ± 4	35 ± 16
		30:70	197	− 19	14.9 ± 4.4	2.5 ± 1.3	23 ± 6	315 ± 228
		10:90		1	213 ± 10	11.8 ± 1.7	29 ± 7	2500 ± 813
	GuaLp$_1$:GlyLp$_3$	30:70	231	6	117 ± 29	6.3 ± 0.7	38 ± 5	1691 ± 422
	SteaLp$_1$:GlyLp$_3$	30:70	264	[a]20	11.8 ± 2.2	1.3 ± 0.4	16 ± 3	114 ± 51
	IsoLp$_2$:GlyLp$_3$	30:70	260	18	128 ± 46	8.7 ± 1.4	15 ± 3	916 ± 223
	[*]EtLp$_1$:GlyLp$_3$	30:70	197	-	11.5 ± 1.0	2.8 ± 0.1	25 ± 3	383 ± 60
	[b]EtLp$_1$:GlyLp$_3$	30:70	227	− 5	8.4 ± 0.9	2.5 ± 0.3	31 ± 5	428 ± 95
	[c]EtLp$_1$:GlyLp$_3$	30:70	188	− 2	7.0 ± 0.7	2.2 ± 0.4	31 ± 3	348 ± 85
As-synthesized 2D-photosets	MenLp$_1$:IsoLp$_2$	90:10		− 33	-	-	-	-
		70:30		− 9	1.9 ± 0.2	0.9 ± 0.1	58 ± 6	280 ± 20
		50:50		− 2	11.8 ± 1.5	1.9 ± 0.2	45 ± 5	478 ± 77
		30:70		4	56.3 ± 6.1	6.8 ± 1.4	67 ± 11	2493 ± 835
		10:90		13	116 ± 17	9.0 ± 1.0	55 ± 4	3016 ± 500
	EtLp$_1$:IsoLp$_2$	30:70		− 17	7.0 ± 1.0	1.4 ± 0.3	21 ± 4	164 ± 60
	GuaLp$_1$:IsoLp$_2$	30:70		− 6	8.7 ± 0.8	2.4 ± 0.4	39 ± 6	500 ± 145
	SteaLp$_1$:IsoLp$_2$	30:70		[a]22	7.7 ± 0.6	1.1 ± 0.1	20 ± 1	122 ± 16
	GlyLp$_3$:IsoLp$_2$	30:70		9	68.4 ± 7.6	5.1 ± 0.5	30 ± 2	930 ± 127
	MenLp$_1$:GlyLp$_3$	30:70		4	13.2 ± 1.2	2.7 ± 0.3	29 ± 2	409 ± 60
	EtLp$_1$:GlyLp$_3$	90:10		− 60	-	-	-	-
		70:30		− 49	-	-	-	-
		50:50		− 38	9.3 ± 0.4	0.6 ± 0.1	7 ± 2	23 ± 13
		30:70		− 15	14.5 ± 1.4	2.0 ± 0.9	15 ± 6	190 ± 139
		10:90		− 7	29.2 ± 3.6	4.8 ± 1.1	23 ± 3	581 ± 215
	GuaLp$_1$:GlyLp$_3$	30:70		− 6	9.8 ± 1.5	2.6 ± 0.1	27 ± 2	365 ± 38
	SteaLp$_1$:GlyLp$_3$	30:70		[a]22	12.1 ± 1.3	1.4 ± 0.2	16 ± 3	128 ± 54
	IsoLp$_2$:GlyLp$_3$	30:70		8	36.5 ± 4.2	4.8 ± 0.7	30 ± 4	792 ± 196
[d]3D prints	MenLp$_1$:IsoLp$_2$	30:70		-	2.0 ± 0.2	0.7 ± 0.2	38 ± 14	148 ± 80
	EtLp$_1$:GlyLp$_3$	30:70		-	7.8 ± 0.4	1.9 ± 0.6	26 ± 8	278 ± 142
	[e]EtLp$_1$:GlyLp$_3$	30:70		-	7.2 ± 0.3	1.3 ± 0.8	19 ± 10	163 ± 150

n ≥ 3 for all mechanical analyses unless specified ([*]2 samples tested); [a]melting temperature (T_m), no T_g detected; [b]1st recycle using thermal depolymerization (DMF) method, [c]2nd recycle using thermal depolymerization (DMF, 140 °C) method. [d]post-cured prints, [e]1st recycle using catalyzed depolymerization (phosphazene:thiophenol) method.

Table S6.

Summary of mechanical data for commercial 3D printing resins.

Supplier	Resin identifier	Suggested application	UTS (MPa)	*E* (MPa)	ε_{break} (%)	Standard	Source/website
Photocentric/BASF	Ultracur3D® EPD 1006	General resin for protypining	40	1500	25	ASTM D638	https://forward-am.com/material-portfolio/ultracur3d-photopolymers/daylight-line/ultracur3d-epd-1006/
Photocentric	DLP Hard	Rigid resin for prototyping	15	2060	4	ASTM D638	https://photocentricgroup.com/wp-content/uploads/2023/01/TDS-UV-Hard-Resin-RV1.pdf
Photocentric	Flexible UV160TR	Highly elastic resin	14.5	66.5	160	ASTM D412	https://photocentricgroup.com/wp-content/uploads/2023/01/TDS-Flexible-UV160TR-Resin-RV1.pdf
Photocentric	Durable UV80	General durable resin	46	1940	13	ASTM D638	https://photocentricgroup.com/wp-content/uploads/2023/01/TDS-Durable-UV80-Resin-RV1.pdf
Photocentric	Rigid DL240	General purpose 50% plant-based resin	64.5	2440	6.1	ASTM D638	https://photocentricgroup.com/wp-content/uploads/2023/01/TDS-Rigid-DL240-Plant-Based-Resin-RV1.pdf
Formlabs	FLGPCL04 Clear	General purpose clear resin	65	2800	12	ASTM D638-14	https://formlabs-media.formlabs.com/datasheets/1801089-TDS-ENUS-0P.pdf
Formlabs	Elastic 50A	Highly elastic resin	3.23	-	160	ASTM D 412-06 (A)	https://formlabs-media.formlabs.com/datasheets/2001420-TDS-ENUS-0.pdf
Formlabs	Flexible 80A	Strong flexible resin	8.9	-	120	ASTM D 412-06 (A)	https://formlabs-media.formlabs.com/datasheets/2001418-TDS-ENUS-0.pdf
Carbon Inc.	EPX 82	High strength resin	80	2800	5	ISO 527-2 Type IA	https://docs.carbon3d.com/files/technical-data-sheets/tds_carbon_epx-82.pdf?_ga=2.265295887.1094821087.1678719174-1623575212.1678719174
Carbon Inc.	EPU 40	Elastic polyurethane resin	19	8	400	ASTM D412	https://docs.carbon3d.com/files/technical-data-sheets/tds_carbon_epu-40.pdf?_ga=2.187039656.1094821087.1678719174-1623575212.1678719174
Carbon Inc.	EPU 43	Energy dampening elastomer	17	10	380	ASTM D412	https://docs.carbon3d.com/files/technical-data-sheets/tds_carbon_epu-43.pdf
3D systems	Accura® 60	Clear rigid plastic	58-68	690-3100	5-13	ASTM D 638	https://www.3dsystems.com/sites/default/files/2020-11/3d-systems-accura-60-datasheet-us-a4-2020-09-14-a-print.pdf
3D systems	Accura® 25	Flexible plastic	38	1590-1660	13-20	ASTM D 638	https://www.3dsystems.com/sites/default/files/2020-11/3d-systems-accura-25-datasheet-us-a4-2020-09-23-a-print.pdf
3D systems	Accura® AMX™ Durable Natural	High toughness plastic	32	1000	80	ASTM D638 Type IV	https://www.3dsystems.com/sites/default/files/2022-05/3d-systems-accura-amx-durable-natural-datasheet-usen-2022-05-12-a-print.pdf
3D systems	Accura Composite PIV	Rigid high perfomance composite	72	9300	1.2	ASTM D638 Type IV	https://www.3dsystems.com/sites/default/files/2021-06/3d-systems-accura-composite-piv-datasheet-usen-2021-06-10-a-print.pdf
Prusa	Prusament resin Flex80	Rubber-like resin	9	18	60	ISO 527-1	https://www.prusa3d.com/product/prusament-resin-flex80-black-1kg/#description
Prusa	Prusament resin Biobased60	60% Biobased resin	30	1090	5.3	ISO 527-1	https://prusament.com/resin-material/prusament-resin-biobased/
